# Measuring stillbirth and perinatal mortality rates through household surveys: a population-based analysis using an integrated approach to data quality assessment and adjustment with 157 surveys from 53 countries

**DOI:** 10.1016/S2214-109X(23)00125-0

**Published:** 2023-05-08

**Authors:** Mohamed M Ali, Saverio Bellizzi, Ties Boerma

**Affiliations:** a Department of Sexual and Reproductive Health and Research, WHO, Geneva, Switzerland; b WHO Jordan Country Office, Amman, Jordan; c Institute for Global Public Health, Max Rady Faculty of Health Sciences, University of Manitoba, Winnipeg, MB, Canada

## Abstract

**Background:**

In most low-income and middle-income countries (LMICs), national surveys are the main data source for stillbirths and perinatal mortality. Data quality issues such as under-reporting and misreporting have greatly limited the usefulness of such data. We aimed to enhance the use of mortality data in surveys by proposing data quality metrics and exploring adjustment procedures to obtain the best possible measure of perinatal mortality.

**Methods:**

We performed a population-based analysis of data from 157 demographic and health surveys (DHSs) from 1990 to 2020, with reproductive calendar and birth history data from 53 LMICs. Pregnancies terminated before 7 months' gestation were excluded. We examined data quality and compared survey values with reference values obtained from a literature review to assess misreporting of the age at early neonatal death, omission and transference of stillbirths, and very early neonatal deaths. Real cohort life-table rates of stillbirth, early neonatal, and perinatal mortality per 1000 births were calculated. The underlying risks of stillbirth and daily deaths were modelled using modified Gompertz-Makeham models.

**Findings:**

Data for 2 008 807 pregnancies of ≥7 months' gestational age were extracted from the reproductive calendar for the analysis period. Age heaping at day 7 occurred in most surveys. The median value for the heaping index of deaths at day 7 was 2·05 (IQR 1·36–2·87). The median ratio of stillbirths to deaths on days 0–1 was 1·15 (0·86–1·51). Of the 157 surveys, 23 (15%) were considered to have plausible ratios, 71 (45%) had probable ratios, and 63 (40%) had improbable ratios. The ratio of deaths on days 0–1 to deaths on days 2–6 varied considerably between surveys and 119 surveys (76%) had ratios of less than 2·4, indicative of under-reporting of very early neonatal deaths in most surveys. The fully adjusted model increased the median stillbirth rates from 12·2 (9·4–15·9) to 25·6 (18·0–33·4) per 1000 births, with a median relative increase of 95·0% (56·6–136·6). The median perinatal mortality rate also increased from 32·6 (23·6–38·3) to 44·8 (32·8–58·0) per 1000 births, with a median relative increase of 47·8% (6·9–61·0).

**Interpretation:**

A simultaneous focus on stillbirths and early neonatal mortality facilitates a comprehensive assessment of inaccurate reporting in household surveys and allows for better use of surveys in planning and monitoring of efforts to reduce stillbirths and early neonatal mortality.

**Funding:**

None.

## Introduction

Globally, an estimated 14 stillbirths per 1000 births and 17 neonatal deaths per 1000 livebirths occurred in 2019.^1^, ^2^ Mortality in low-income and middle-income countries (LMICs) was considerably higher, with estimates of 19 stillbirths per 1000 births and 25 neonatal deaths per 1000 livebirths. The UN Sustainable Development Goals include a target to reduce neonatal mortality to 12 per 1000 livebirths by 2030.^3^ The global Every Newborn Action Plan in 2014 set a target for stillbirths to be reduced to 12 per 1000 births by 2030.^4^ Although the world has increasingly turned its attention towards monitoring and reducing neonatal mortality, stillbirths have received much less attention and remain neglected by policy makers and funding agencies.^5^

In LMICs, household surveys, especially demographic and health surveys (DHSs),^6^ are the main source of population data on stillbirth and neonatal mortality. The quality of neonatal mortality data is generally considered good, although under-reporting and misreporting are issues in some surveys.^7^, ^8^ The collection of data on stillbirths is either based on questions regarding the outcome of pregnancy that are administered in addition to a livebirth history, or based on a full pregnancy history.^9^ The quality of survey data on stillbirths is considered poorer than that for neonatal mortality, mainly due to omission of deaths.^10^

**Research in context**

**Evidence before this study**

We reviewed the published literature over the past 15 years around stillbirths, neonatal deaths, and perinatal mortality. Stillbirths and early neonatal deaths can be considered together as deaths in the perinatal period because of several commonalities in terms of measurement and causes. In low-income and middle-income countries, household surveys are the main source of population data on stillbirth and neonatal mortality. Although the quality of neonatal mortality data is generally considered good, under-reporting and misreporting are issues in some surveys, and the quality of data on stillbirths is considered poor because of the omission of deaths.

**Added value of this study**

We showed that a comprehensive assessment of the quality of survey data on stillbirths and neonatal deaths provides better insights into data quality and can help produce adjusted measures. We used three data quality metrics with 157 surveys in 53 countries and showed that under-reporting of stillbirths (ie, under-reported or, less commonly, misreported as very early neonatal deaths) is a major issue in more than 95% of the surveys. Our statistical model that adjusted for possible omissions, transference, and under-reporting showed median relative increases of 95·0% for the stillbirth rate and 47·8% for the perinatal mortality rate.

**Implications of all the available evidence**

All surveys should be guided by a comprehensive data quality assessment that includes stillbirths and early neonatal deaths, which helps to interpret survey findings and might lead to adjusted mortality measures. Past survey results on data quality metrics should inform training and data quality monitoring of future surveys. A joint assessment of stillbirth and neonatal mortality data should also support the case for greater attention to stillbirths, often regarded as a neglected public health issue.

Perinatal mortality is a well established indicator that captures infant deaths between the end of the 28th week of pregnancy and the end of the first week of life.^11^ Intrapartum complications such as asphyxia are associated with as many as half of all stillbirths^10^, ^12^ and are also a leading cause of early neonatal death.^13^ A simultaneous focus on mortality in the late fetal and early neonatal periods provides an opportunity to assess the extent of incomplete or inaccurate reporting in surveys, such as omission and transference of stillbirths and neonatal deaths, and misreporting of the age at death, and lays a foundation for adjustments of such reporting biases.

This paper aims to enhance the appropriate use of data on stillbirth and neonatal mortality in surveys for the assessment of levels, trends, and inequalities. The paper proposes data quality metrics and explores adjustment procedures to obtain the best possible measure of perinatal mortality and its components from individual household surveys.

## Methods

### Data source

We performed a population-based analysis of 157 DHSs with reproductive calendar and birth history data from 53 LMICs that are publicly available,^14^ after excluding 15 surveys in which the number of deaths (unweighted) was less than 25 for stillbirths or deaths on days 0 and 1 (list of excluded surveys in appendix p 2).

The birth history collects information on the survival and age at death of the livebirths of babies born to respondents aged 15–49 years. The reproductive calendar is a separate instrument that includes a month-by-month history of women's reproduction such as pregnancy terminations and contraceptive use. In most surveys, the period covered by the calendar includes the months up to the month of interview in the year of interview, plus the 5 calendar years preceding the year of interview.

We extracted the gestational age (in months) and pregnancy outcomes (birth, termination, currently pregnant) from the calendar. Pregnancies terminated before 7 months' gestation were excluded, in line with WHO's definition of stillbirths and perinatal mortality. Livebirths were linked with the corresponding birth (including multiple births) in the birth history to obtain the survival status, current age, and age at death (day, month, and year). Current pregnancies of 7 or more months were also included, as censored on the survey month. We retained data for pregnancies that were conceived between 7 and 66 months before the survey date. Livebirths that occurred in the month of the interview were assumed to be more than 1 week old.

Because stillbirths and the current age of living children are reported in months, whereas the age at death of neonates is reported in days, the data were considered interval censored and restructured to compute life tables and fit models for interval-censored data. Ethics approval was not required for this secondary data analysis.

### Data quality assessment

In our analysis we examined three concerning data quality issues: misreporting of the age at early neonatal death, omission and transference of stillbirths, and very early neonatal deaths. Building on previous work,^7^, ^8^, ^15^ we used four indicators to examine different aspects of data quality, and compared survey values with reference values obtained from a review including vital registration data, historical data, perinatal mortality surveillance in higher mortality settings, and prospective studies of the outcome of pregnancy (table 1; appendix p 3).

**Table 1:** Data quality issues and adjustment metrics

	**Indicator**	**Reference values**	**Adjustment metric**
Misreporting of age at death in first week	Age heaping index: the number of deaths on day 7 divided by a fifth of the number of deaths on days 5–9	Improbable: <0·6; probable: 0·6–0·8; plausible: 0·8–1·3; probable: 1·3–1·5; improbable: ≥1·5	Smoothing via the Gompertz-Makeham model linear predictor
Under-reporting stillbirths or misclassification on days 0–1	Ratio of stillbirths (gestational age ≥7 months) to deaths on days 0–1	Improbable: <1·0; probable: 1·0–1·7; plausible: 1·7–3·0; probable: 3·0–4·0; improbable: ≥4·0	If the ratio is less than the median of 1·89, then: STB/(D0–1)=1·89
Under-reporting of stillbirths or misclassification	Ratio of stillbirths to deaths in the first week (days 0–6)	Improbable: <0·5; probable: 0·5–1·0; plausible: 1·0–1·9; probable: 1·9–2·4; improbable: ≥24	Not used for adjustment
Under-reporting of very early neonatal deaths	Ratio of deaths on day 0–1 to deaths on day 2–6	Plausible: ≥2·4	If the ratio is less than 2·4, then weight D0–1 by: (D6–2)/(D0–1) × 2·4

Reference values were compared with observed survey-specific value of each indicator. STB=stillbirths. D=deaths.

### Misreporting of age at death

Heaping of the age (the tendency to systematically misreport age at death onto age 7 days at the expense of neighbouring days) at neonatal death at 7 days commonly occurs in surveys. Such deaths would fall outside the perinatal period that includes up to the end of day 6. An age heaping index is calculated as five times the number of deaths reported on day 7 divided by the sum of all deaths on days 5–9. A modified Gompertz-Makeham model was used to redistribute the deaths heaped on day 7 (table 1).

Misreporting of the age at death in surveys is also common for deaths in the first 2 days of life. Strictly, deaths within the first 24 h of life should be classified as first-day deaths (which we refer to as day 0), and those occurring 24–47 h after birth as second-day deaths (ie, day 1). In practice, the respondent or interviewer may use day 0 to report or record deaths on the calendar date of the birth and use day 1 for deaths occurring on the following calendar date, even though these deaths might still occur within 24 h. This commonly occurs in DHSs and varies between surveys.^8^ Highly variable reporting of deaths on days 0 and 1 is also observed in prospective studies of the outcome of pregnancy. For example, the proportion of deaths in the first 2 days of life recorded as first-day deaths varied from 21% to 81% in the ten prospective AMANHI studies.^16^ Therefore, in our analysis we combined deaths on day 0 and day 1.

### Omission and transference

Previous studies have concluded that there is no widespread evidence of omission of neonatal deaths in DHSs in most countries.^7^, ^17^ The greatest concern is under-reporting of very early neonatal deaths in surveys, which was considered to occur in about one in seven DHSs.^8^ In general, stillbirths are more likely to be under-reported than neonatal deaths in surveys.^10^, ^18^, ^19^ If neonatal death reporting is accurate, the ratio of stillbirths to neonatal deaths can be used to assess under-reporting of stillbirths and adjust for this bias, or exclude the survey values, as is mostly done in global analyses.^20^

Several small-scale studies have shown transference of stillbirths and neonatal deaths in surveys.^21^, ^22^ Registration practices and sociocultural factors might compound the reporting of stillbirths and perhaps very early neonatal deaths in surveys, and might lead to a combination of under-reporting and transference.^9^, ^23^ For example, a newborn baby who is alive but not breathing is reported as a stillbirth. This would lead to under-reporting of neonatal mortality rates and increase the ratio of stillbirths to early neonatal deaths. But because omission is concurrently present, the magnitude of transference is difficult to assess. The opposite is also true; ie, assessment of the extent to which omission occurs is affected by transference. We have no systematic method to disentangle the two effects.

We assessed two ratios: stillbirths (gestational age ≥7 months) to early neonatal mortality (ie, occurring in the first week [days 0–6]) and stillbirths to deaths in the first 2 days (days 0–1). The ratio of stillbirths to deaths in the first 2 days has the advantage of a stronger aetiological cohesion but includes the problem of greater uncertainty in the neonatal mortality measures. In addition, we used the ratio of neonatal mortality on days 0–1 to days 2–6 to assess under-reporting of very early neonatal deaths (ie, in the first 2 days). On the basis of our review of published data from various sources for LMICs and historical populations, we selected ranges for plausible, probable, and improbable ratio values for each of the four metrics (table 1). We opted for constant reference values because the selected data quality metrics did not show a clear pattern by level of early neonatal mortality, except for very low levels of mortality that are below the range of mortality rates in countries with DHSs.

The choice of the reference value and the range to identify plausible, probable, and improbable values for the indicators of data quality is arbitrary.^24^ True ratios are likely to vary between populations and over time in accordance with demographic factors (such as fertility and parity distribution), epidemiology, and home and health facility delivery practices. In addition, sampling error contributes to variation of the ratios.^25^ The summary of the selection of indicators and proposed reference values and adjustment metrics is shown in table 1.

Data quality might be associated with a range of factors related to the survey or respondents' characteristics. We examined the extent to which the data quality metrics were influenced by geographical region (sub-Saharan Africa; north Africa, west Asia, or Europe; rest of Asia; and Latin America and the Caribbean), household wealth (poor, middle, and rich), urban-rural residence, level of education (none, primary, and secondary or more), age of the respondent at birth in years (<25, 25–29, and ≥30 years), calendar period of birth (<2000, 2000–04, 2005–09, 2010–14, and ≥2015), and number of living children at birth (0, 1, 2, 3, or ≥4).

### Statistical analysis

Age-specific risks of death and the cumulative probabilities of death from 7-month gestation to day 10 were computed using life-table methods for real cohorts; currently alive or born in the month of the survey, or died after day 10 were censored at day 10. Day 10 was chosen to calculate the heaping index between days 5 and 9. Life-table rates of stillbirth, early neonatal mortality, and perinatal mortality per 1000 births were calculated. The data were restructured to fit a piece-wise exponential, with complementary log–log transformation link function log{–log(1–π_ij_)}=γ_1_ + γ_2_ + γ_3_ + ···γ_k_, which is equivalent to a life table (appendix p 11). Then the underlying risks of stillbirth and daily deaths were modelled using a modified Gompertz-Makeham model:

$$\gamma_{j}=\{\begin{array}{cc} \gamma_{1} & ifj=1\\ \gamma_{2} & ifj=2\\ \gamma_{0}+\gamma_{j} & ifj\geq 3 \end{array}$$

The flexibility of the modified Gompertz-Makeham model allows for specific linear predictors and model coefficients' adjustment.

To account for under-reporting and transference between stillbirth to death on days 0 and 1 in both directions, the Gompertz-Makeham model included a constrain equation (equation 3) for the first two coefficients of stillbirth and death on days 0 and 1 to be [γ_1_ – γ_2_=log(1·89)] if the observed ratio was less than a median value of 1·89. If the observed ratio of deaths on days 0 and 1 to deaths on days 2 to 6 was lower than 2·4, then deaths on days 0–1 were weighted by 2·40 divided by the observed ratio and weighted by 1·89 to simultaneously adjust for under-reporting and transference. The functional form of equation 2's linear predictor smooths the heaping on day 7.

We assessed the correlations of the data quality indicators with early neonatal mortality using the Pearson correlation coefficient, and the variations with maternal characteristics using ANOVA.

The piece-wise exponential model was used to measure the observed age-specific risk of death and the cumulative probabilities of stillbirth, early neonatal mortality, and perinatal mortality (model 1). The modified Gompertz-Makeham model was used to adjust for possible omission, transference, and under-reporting, as well as smoothing. We adjusted for stillbirth transference and omission (model 2), the under-reporting of very early neonatal mortality (model 3), and finally we adjusted for both simultaneously (model 4).

### Role of the funding source

There was no funding source for this study.

## Results

We retained 157 surveys, covering the period from 1990 to 2020 (appendix p 14). Data for 2 008 807 pregnancies of 7 or more months' gestational age were extracted from the reproductive calendar for the analysis period. Survey-specific numbers ranged from 2342 in Azerbaijan in 2006 to 256 211 India in 2015–16. The number of stillbirths ranged from 26 in Timor-Leste in 2009–10 to 3147 in India in 2015–16 (appendix p 14).

A summary of the data quality indicators that include ratios and proportions of stillbirths and early neonatal mortality and heaping at day 7 is presented in table 2 (survey-specific values are in the appendix p 18). The level of associations and degree of variations of the three data quality indicators with the characteristics of the surveys and the respondents, and early neonatal mortality are further examined and summarised in the appendix (pp 30–31).

**Table 2:** Summary statistics of all assessed data quality indicators

	**Range**	**Median (IQR)**	**Mean (SD)**
Heaping index at day 7	0·00–4·28	2·05 (1·36–2·87)	2·10 (0·99)
Stillbirths to D0–1 ratio	0·22–4·72	1·15 (0·86–1·51)	1·26 (0·63)
Ratio of stillbirths to the number of deaths in the first week	0·14–2·22	0·73 (0·58–0·95)	0·78 (0·31)
D0–1 to D2–6 ratio	0·43–5·30	1·92 (1·48–2·38)	2·05 (0·90)

D0–1=deaths on days 0–1. D2–6=deaths on days 2–6.

Age heaping at day 7 occurred in most surveys. Of 157 surveys, 121 (77%) surveys had improbable ranges, 19 (12%) had probable ranges, and 17 (11%) had plausible ranges. The median heaping index of deaths at day 7 was 2·05 (IQR 1·36–2·87). Survey-specific heaping index results are in the appendix (pp 30–31). The extent of early neonatal mortality was not correlated with the heaping index, but there was a significant correlation between the heaping index and other two data quality measures: a significant negative correlation with the ratio of stillbirth to deaths on days 0–1 (*r*[157]=–0·2686, p=0·0007), and a positive correlation with the ratio of deaths on days 0–1 to deaths on days 2–6 (*r*[157]=0·2219, p=0·0052).

There was substantial variation in the heaping index between regions only (appendix p 30). A nested ANOVA for a subset of countries with three or more surveys showed that there was significantly more variation between countries than within countries.

The median ratio of stillbirths to deaths on days 0–1 was 1·15 (IQR 0·86–1·51). Of the 157 surveys, 23 surveys (15%) were considered to have plausible ratios, 71 surveys (45%) probable ratios, and 63 surveys (40%) improbable ratios; and 141 surveys (90%) reported ratios of less than the median of 1·89. The correlation between the ratios and early neonatal mortality was moderate (*r*[157]=–0·4245, p<0·0001), indicating that the higher the ratio, the lower the mortality rate. The ratio varied significantly (as measured by the mean square error from the ANOVA) by region (significantly high in sub-Saharan Africa and Asia) and increased with maternal age (appendix p 30). The within-country variance was significant (p<0·0001), and the between-country variance was not significant.

The ratio of deaths on days 0–1 to deaths on days 2–6 varied considerably between surveys, with a median of 1·92 (IQR 1·48–2·38), and 119 surveys (75·8%) had ratios of less than 2·4, indicative of under-reporting of very early neonatal deaths in most surveys. We found an association between the ratio and early neonatal mortality, and geographical region, place of residence, level of education, and maternal age. Variances between and within countries were not significant, suggesting that underlying age-specific mortality patterns or consistent reporting patterns of deaths by age are key drivers of the ratio.

**Figure 1:**
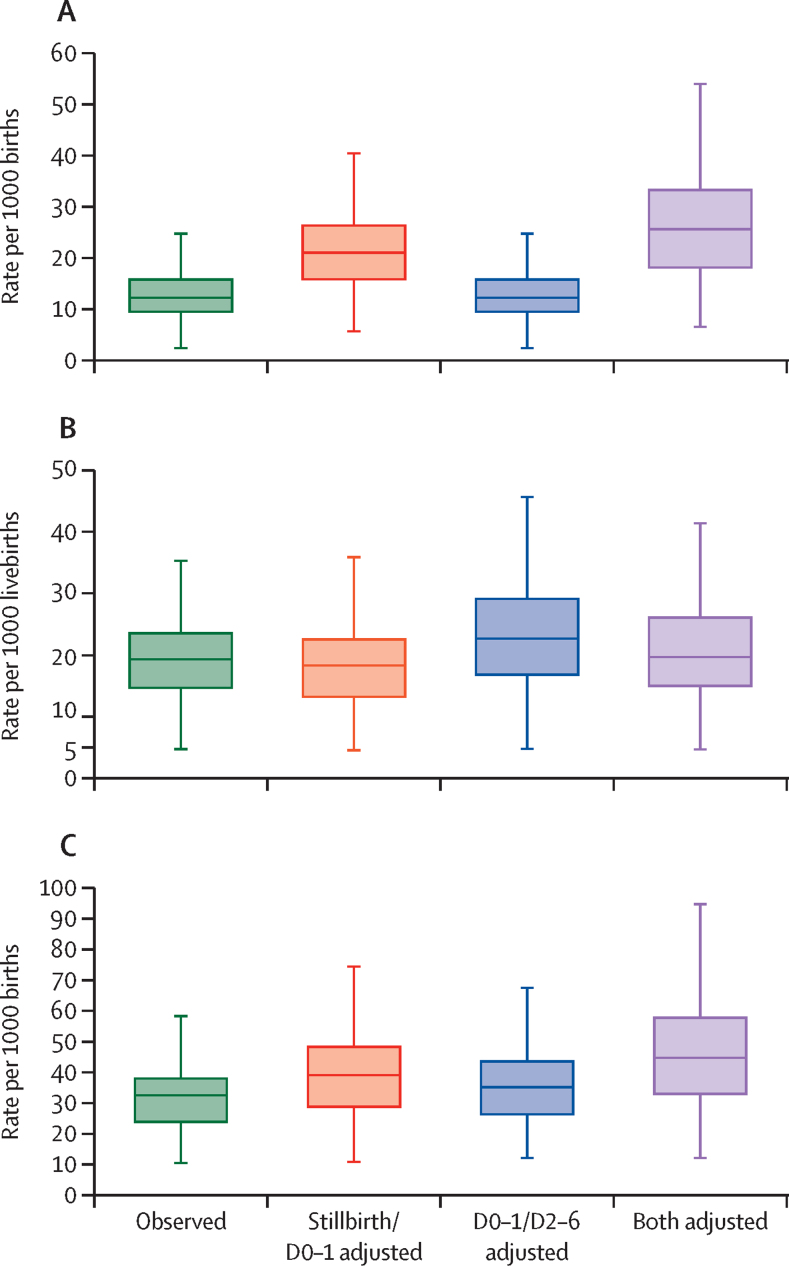
Observed and adjusted rates of (A) stillbirths, (B) early neonatal mortality, and (C) perinatal mortality Data exclude outside values. D0–1=deaths on days 0–1. D2–6=deaths on days 2–6.

**Figure 2:**
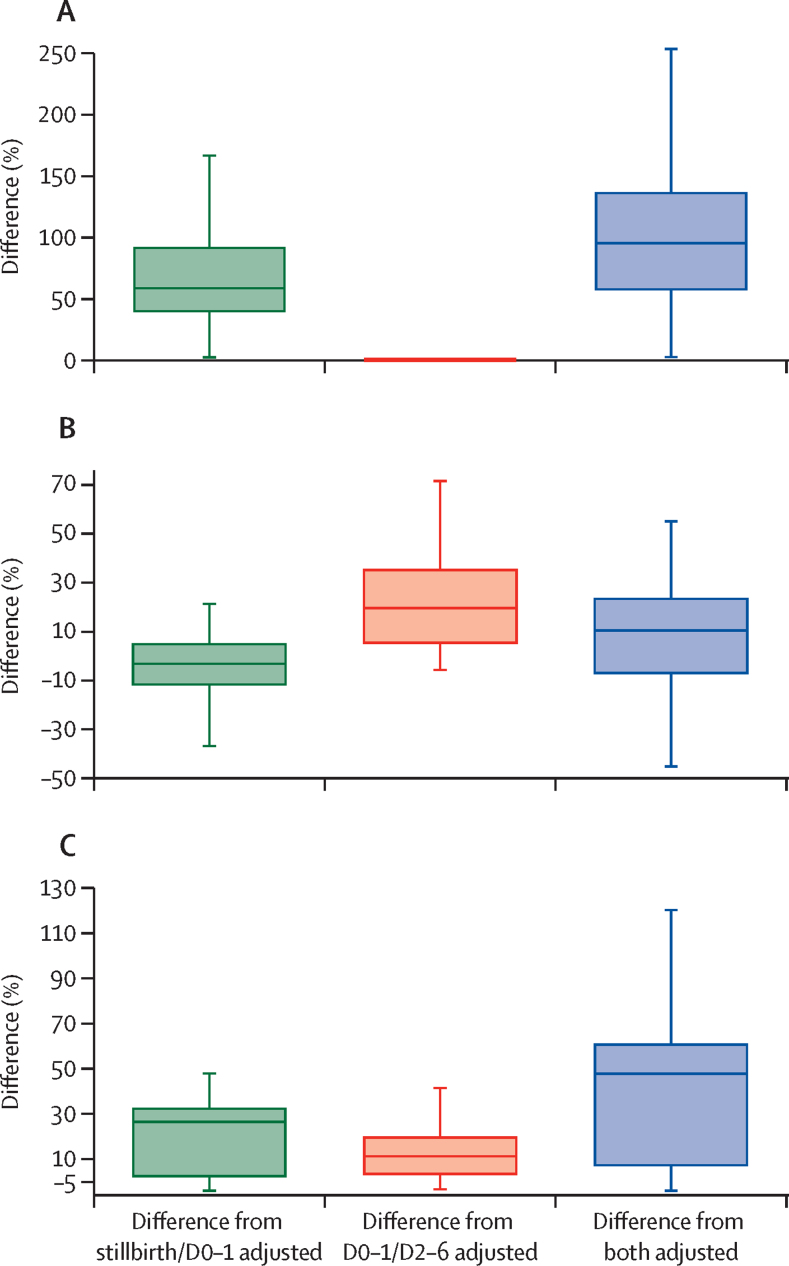
Differences between observed and adjusted rates of (A) stillbirths, (B) early neonatal mortality, and (C) perinatal mortality Data exclude outside values. D0–1=deaths on days 0–1. D2–6=deaths on days 2–6.

Table 3 summarises the observed and adjusted measures of perinatal mortality and its components, with relative differences showed in box-whiskers plots (Figure 1, Figure 2). The survey-specific observed and adjusted rates, and age-specific risk of death and cumulative probabilities are in the appendix (pp 37, 41).

**Table 3:** Observed and adjusted stillbirth, early neonatal, and perinatal mortality rates, and relative differences

	**Stillbirth rates per 1000 births**				**Early neonatal mortality per 1000 livebirths**				**Perinatal mortality per 1000 births**			
	Model 1	Model 2	Model 3	Model 4	Model 1	Model 2	Model 3	Model 4	Model 1	Model 2	Model 3	Model 4
Rates per 1000 births or livebirths	12·2 (9·4 to 15·9)	21·0 (15·7 to 26·5)	12·2 (9·4 to 15·9)	25·6 (18·0 to 33·4)	19·2 (14·4 to 23·6)	18·2 (13·0 to 22·5)	22·6 (16·5 to 29·1)	19·5 (14·7 to 26·1)	32·6 (23·6 to 38·3)	39·1 (28·6 to 48·6)	35·2 (26·1 to 43·8)	44·8 (32·8 to 58·0)
Relative differences from model 1 (% median)	NA	58·3% (38·8 to 91·8)	0	95·0% (56·6 to 136·6)	NA	3·4% (−12·3 to 5·0)	19·2% (4·9 to 35·2)	10·3% (−7·5 to 23·4)	NA	26·5% (2·1 to 32·7)	11·3% (3·1 to 32·7)	47·8% (6·9 to 61·0)

Data are median (IQR). NA=not applicable. Model 1=piece-wise exponential model. Model 2=Gompertz-Makeham model adjusted for stillbirth transference and omission only. Model 3=Gompertz-Makeham model adjusted for the under-reporting of very early neonatal mortality. Model 4=Gompertz-Makeham model adjusted for stillbirth transference, omission, and the under-reporting of very early neonatal mortality.

The fully adjusted model (model 4) increased the median stillbirth rate from 12·2 (IQR 9·4–15·9) to 25·6 (18·0–33·4) per 1000 births, with a median relative increase from model 1 to model 4 of 95·0% (56·6–136·6). Similarly, the perinatal mortality rate increased from a median 32·6 (23·6–38·3) per 1000 births with model 1 to 44·8 (32·8–58·0) with model 4, with a median relative increase of around 47·8% (6·9–61·0). Adjusting for the stillbirth transference increased the stillbirth rate but slightly decreased the early neonatal rate, whereas adjusting for very early neonatal death under-reporting had no effect on the median stillbirth rate but moderately increased the median early neonatal mortality rate from 19·2 (14·4–23·6; model 1) to 22·6 (16·5–29·1) per 1000 livebirths (model 3).

## Discussion

We developed a flexible statistical model to adjust for data quality when measuring stillbirth and early neonatal mortality rates. Our analysis showed that it is possible to make more use of stillbirth and age-specific neonatal mortality data in household surveys by adding data quality metrics and adjusting for under-reporting and transference of deaths. A simultaneous focus on stillbirths and early neonatal mortality facilitates a comprehensive assessment of the effect of incomplete or inaccurate reporting in surveys, and enables countries to make full use of household surveys in the planning and monitoring of efforts to reduce late fetal and early neonatal mortality.

The UN Inter Agency Group on child mortality estimation runs separate modelling exercises for stillbirths and neonate deaths. Although global estimates are informative and provide a useful benchmark, country planning and monitoring are generally based on adjusted or non-adjusted statistics from household surveys. Ideally, such methods are available and can be applied to a single country survey. Country survey reports, such as the DHS, give much attention to neonatal mortality but stillbirths and deaths in the first week are usually presented in just a short section on perinatal mortality. Therefore, country planning and monitoring rarely use stillbirth-related statistics from surveys.

Our analysis shows that a combined assessment of data quality and adjustment of mortality rates is possible and should be used regularly as part of survey analysis and reporting. This principle also applies to the new round of DHSs, which have included pregnancy histories instead of birth histories with a reproductive calendar as the standard survey instrument. A previous study^26^ showed that the full pregnancy history might lead to an improvement in stillbirth reporting, although under-reporting of stillbirths seems to persist to varying degrees.

Another argument for the joint assessment of stillbirth and neonatal mortality data is that this approach would support the case for greater attention to stillbirths, which has often been flagged as a neglected public health issue.^1^ Although there are important arguments for separate analysis in terms of interventions, there are also reasons for combined analysis from the aetiological perspective. Our intention is not to recommend only perinatal mortality statistics, but to show that perinatal mortality rates are useful in addition to stillbirth and neonatal mortality rates.

Looking across the three indicators, we can highlight five findings that show the value of a comprehensive approach. First, the under-reporting of stillbirths was the most serious data quality issue in most of the surveys, as shown by the much lower than expected ratios of stillbirths to neonatal deaths on days 0–1. The age heaping index was also often implausibly high but its effect on early neonatal mortality or perinatal mortality rates was small because of the lower number of deaths after the first few days of life. Second, the data quality metrics in the same survey were significantly correlated, meaning that having an improbable value for one indicator was a predictor of a similarly improbable value for another indicator. Third, we found some evidence that past surveys within the same country can predict data quality metrics for age heaping and the early neonatal mortality ratio for deaths on days 0–1 to deaths on days 2–6, but not the ratio of stillbirths to deaths on days 0–1. Also, for both mortality ratios it was possible to identify countries in which less plausible values were more common. Both findings can help with training and data quality monitoring in future surveys. Fourth, studies of inequalities need to take into account differential reporting biases. Fifth, the approach of adjustment of mortality rates allowed us to provide adjusted mortality measures, which for stillbirth and perinatal mortality differed greatly from the unadjusted rates.

Our study had several limitations. The sex of fetuses was not recorded, and ethnicity was only inconsistently recorded. Additionally, our analysis did not solve the fundamental data quality problem, which is primarily related to severe under-reporting of stillbirths in surveys; the data quality metrics are useful as they show the magnitude of this problem. The model-based adjustment provided improved mortality measures, but in several surveys stillbirth rates were lower than expected and uncertainty remained high for the adjusted measures. The reference values for the data quality metrics were based on a review of the literature, which included only four studies. Further work to refine these values is needed. We also assumed constant values for the data quality metrics, which helped to simplify the assessment and adjustments. We found that the ratio of deaths in days 0–1 to days 2–6 and the heaping index were independent of early neonatal mortality levels in the same survey, but that the ratio of stillbirths to deaths in days 0–1 increased as early neonatal mortality decreased. Additional efforts are needed to determine a formalised approach, validated through a series of models with different cutoff values, to further enhance the perinatal mortality measures. Surveys are only one source of mortality data; other sources include more longitudinal data collections occurring in multiple settings. Combining the survey results with health facility data (which would be especially useful if a high proportion of women deliver in health facilities) and research studies might improve the measures of stillbirth mortality, but would still require regional and global parameters to fill data gaps.^1^ Neonatal mortality rates are, however, not available from routine health facility reports, as newborn babies are discharged soon after delivery. Finally, we used the DHS cutoff of 7 months' gestational age (based on the WHO definition^27^ of 28 weeks of pregnancy) for our analysis. Reporting of gestational age in weeks in the full pregnancy history might allow further refinement of our model approach in the future.

Our proposed approach contributes to better use of survey data to obtain more reliable stillbirth, neonatal, and perinatal mortality values. Such information is crucial to improve programming and monitoring of perinatal mortality in LMICs that rely on household surveys.

## Data sharing

All data are available at https://www.dhsprogram.com/data/availabledatasets.cfm. The demographic and health surveys (DHSs) program is authorised to distribute, at no cost, unrestricted survey data files for legitimate academic research. Registration is required to be able to download the data. Researchers are required to provide their contact information, the research title, and a description of the proposed analysis. Approval to access datasets is usually granted and communicated via email. The data are third-party and are not owned or collected by the authors, and the authors do not have any special access privileges. The created analysis files from the reproductive calendar are available upon request to the corresponding author (MMA).

## Declaration of interests

We declare no competing interests. MMA and SB are staff members of WHO. TB is a professor at the Institute for Global Public Health, Max Rady Faculty of Health Sciences, University of Manitoba. The authors alone are responsible for the views expressed in this publication, and they do not necessarily represent the decisions, policy, or views of WHO or University of Manitoba. The country names used do not imply the expression of any opinion whatsoever on the part of WHO or University of Manitoba concerning the legal status of any country, territory, city, or area, or of its authorities.
