# Correction to *Lancet Glob Health* 2023; 11: e854–61

**DOI:** 10.1016/S2214-109X(23)00242-5

**Published:** 2023-05-17

*Ali MM, Bellizzi S, Boerma T. Measuring stillbirth and perinatal mortality rates through household surveys: a population-based analysis using an integrated approach to data quality assessment and adjustment with 157 surveys from 53 countries.* Lancet Glob Health *2023; **11:** e854–61*—For this Article, the supplementary appendix has been corrected. This correction has been made as of May 17, 2023.

