# Supplementary material for: Measuring stillbirth and perinatal mortality rates through household surveys: a population-based analysis using an integrated approach to data quality assessment and adjustment with 157 surveys from 53 countries
Source: Lancet Glob Health. 2023 May 8;11(6):e854–61. doi: 10.1016/S2214-109X(23)00125-0 (PMC10188368; doi:10.1016/S2214-109X(23)00125-0)

THE LANCET
Global Health

Supplementary appendix

This appendix formed part of the original submission and has been peer reviewed. We post it as supplied by the authors.

Supplement to: Ali MM, Bellizzi S, Boerma T. Measuring stillbirth and perinatal mortality rates through household surveys: a population-based analysis using an integrated approach to data quality assessment and adjustment with 157 surveys from 53 countries. *Lancet Glob Health* 2023; published online May 8. https://doi.org/10.1016/S2214-109X(23)00125-0.

Measuring stillbirth and perinatal mortality rates through household surveys: a population-based analysis using an integrated approach to data quality assessment and adjustment with 157 surveys from 53 countries

Mohamed M Ali, Saverio Bellizzi, Ties Boerma

Table of Contents

Appendix 1- Table A1: Excluded survey 2
Appendix 2: Derivation of the adjustment ratios 3
Appendix 3: The DHS surveys and the statistical model used 11
Appendix 4: Number of pregnancies 14
Appendix 5: Data quality indicators 18
Appendix 6: data quality correlations and associations 30
Appendix 7: Scatter plots of data quality indicators 31
Appendix 8: Country-specific results 37
Appendix 9: country-specific observed and adjusted risk and cumulative risk of death 41

Appendix 1- Table A1: Excluded survey

**Country**	**DHS survey round**	Stillbirth	Death D0-1
Comoros	2012	13	41
Albania	2008/9	8	10
Albania	2017/18	7	1
Armenia	2000	28	17
Armenia	2005	12	4
Armenia	2010	10	5
Armenia	2015/16	10	3
Moldova	2005	14	3
Ukraine	2007	6	2
Kazakhstan	1999	16	18
Kyrgyz Republic	2012	22	25
Vietnam	1997	21	24
Vietnam	2002	8	16
Colombia	1990	47	22
Guyana	2009	25	22

Appendix 2: Derivation of the adjustment ratios

Our main purpose is to obtain reference values for the ratios of stillbirths to neonatal deaths and ratios within the neonatal period to assess the quality of data on stillbirths and neonatal deaths in surveys and adjust for under- or misclassification of deaths using a statistical model. There are a limited number of publications based on longitudinal studies that are of sufficient quality and clarity to assess ratios of deaths between stillbirths and neonatal deaths and within the neonatal period. We reviewed vital registration data, historical data, perinatal mortality surveillance in higher mortality settings, and longitudinal studies.

Vital registration data: UN database

The registration of stillbirths in the context of civil registration and vital statistics systems is mandatory in many but not all countries and has been introduced at different points in time. The UN Statistical Division maintains a database with stillbirths and neonatal mortality by age, based on reported data from countries on an annual basis.[1] Table A.1. presents the summary of an analysis 176 countries and territories for the period 1970-2016 The country medians of the ratios for four indicators of the age pattern of mortality are shown by level of early neonatal mortality (first week).

Table A2.1. Selected ratios of stillbirths and early neonatal mortality by level of early neonatal mortality (day 0-6), vital registration database, UN Population Division**.**

	**Median values**				
**Early neonatal mortality (per 1,000 live births)**	**Stillbirth: Day 0-1**	**Stillbirth: Day 0-6**		**Day 0: Day 1**	**Day 0-1: Day 2-6**
**< 3**	2.62	1.65		2.96	2.03
**3-4**	1.54	1.04		2.83	2.02
**5-7**	1.19	0.80		1.68	1.46
**8+**	1.08	0.65		1.40	1.79
All	2.00	1.29		2.50	1.91

		STB/D0-1	STB/D0-6	D0/D1	D0-1/D2-6
**World Bank classification**					
	Developed	2.10	1.36	2.43	1.86
	LMICs	1.68	1.09	2.57	2.00
**Average**		1.89	1.23	2.50	1.93

[1] https://unstats.un.org/unsd/demographic-social/sconcerns/birth_death/

There is a clear association with the level of mortality. The higher the early neonatal mortality, the lower the ratio of stillbirths to deaths on day 0-1, or stillbirths to day 0-6. The day of deaths on days 0-1 to days 2-6 also declines but much less so. Finally, the predominance of deaths on day 0 compared to day 1 also declines strongly with mortality levels.

There are too few country years with early neonatal mortality levels of 10 or higher which limits the ability to examine the ratios at higher levels of mortality. The most relevant statistics for our study in low- and middle-income countries are for the group of countries with the highest levels of early neonatal mortality (8 per 1,000 and over): the stillbirth to days 0-1 ratio is 1.08 and the days 0-1 to days 2-6 ratio is 1.79.

Vital registration data: PAHO

During 1968-1970 PAHO coordinated a mortality surveillance project in 15 sites in 13 countries in Latin America and the Caribbean.[2] Stillbirths were not included. The early neonatal mortality rates ranged from 16-25 per 1,000 live births. The median range of mortality on day 0 to days 1-6 was 1.07, ranging from 0.76 to 1.34. Mortality in the first week was 2.3 times higher in week 1 than during weeks 2-4.

Table A2.2 Early neonatal mortality rate (per 1,000 live births), ratios mortality on day 0 to day 1-6 and week 1 to week 2-4, PAHO 15 site study, 1968-1970.

	**Early neonatal mortality rate**	**Ratio day 0 / day 1-6**	**Ratio week 1 / week 2-4**
**Medellin, Colombia**	16.2	0.76	4.6
**Cartagena, Colombia**	17.3	0.90	3.3
**Kingston-St Andrew, Jamaica**	17.8	0.80	2.8
**Cali, Colombia**	20.3	1.05	4.0
**Monterrey, Mexico**	18.5	1.34	2.5
**Chile project**	19.4	1.02	2.7
**Ribeirao Preto, Brazil**	21.5	1.15	3.2
**Bolivia project**	17.0	0.98	1.4
**El Salvador project**	19.5	1.01	1.9
**Chaco province, Argentine**	22.7	0.88	2.3
**Sao Paulo, Brazil**	20.3	1.11	1.5
**Recife, Brazil**	25.3	0.86	2.5
**San Juan province, Argentina**	24.7	0.80	1.8
**Median**	20.1	1.07	2.7
Without Medellin	20.3	1.07	2.3

[2] Puffer RR, Serrano CV. Patterns of mortality in childhood. Pan American Health Organization. 1973. Washington DC.

If we assume that 11% of deaths in the first week occur on the second day of life (as found in the Global Network Study, ref 7), the mortality ratio day 0-1 to day 2-6 is 1.4 for all sites combined.

Historical data

A WHO review of historical data in Europe from 1900 showed that the ratio stillbirth to early neonatal deaths was about 1.2 or higher.[3] The ratio stillbirth rate to early neonatal mortality rate in long-term data series, from 1900 where available, is shown by level of mortality in the Table A.3, copied from the report, based on 10 countries and Hong Kong. It was concluded that ratio of 1.2 at early neonatal mortality levels of 20 or above presented a good approximation. This ratio was used by WHO to estimate stillbirth rates from early neonatal mortality rates for higher mortality countries without reliable stillbirth data.

Woods reviewed the WHO estimates in detail and conducted further analyses of historical population data from England and northwest Europe, suggesting that somewhat lower ratios are possible, especially if one assumes that stillbirth rates rarely go over 70 per 1,000 live births.[4] Yet, a ratio of 1.2 was generally considered a good approximation, even at higher levels of mortality.

Table A2.3: Historical data, from WHO, 2006.

Table A6.2 SBR/ENMR ratio at different early neonatal mortality levels from different sources and long-term data series

Early neonatal mortality rate	All long-term data in ENMR range, all countries, *all years*[i] Average; median; (range)	1900-50 long-term data series, *all*[ii] countries. Average; median; (range)	1900-50 long-term data series, *selected*[iii] countries. Average; median; (range)	France (1853 and 1913)
25-	-	-	-	1.4
20-24	1.4; 1.2; (0.9-1.9)	1.4; 1.2; (0.9-1.9)	1.5; 1.5; (1.2-1.9)	-
15-19	1.3; 1.4; (0.8-1.6)	1.4; 1.4; (0.9-1.6)	1.4; 1.4; (1.0-1.6)	2.7
10-14	1.3; 1.3; (0.7-2.6)	1.5; 1.5; (1.4-1.7)	1.4; 1.5; (1.2-1.6)	-

i Most recent entries: Chile 1974, Mexico 1982.
ii Denmark, England, Hong Kong, the Netherlands, Norway, Scotland, Singapore, Sweden and USA.
iii Denmark, England, Netherlands, Norway, Scotland and Sweden.

[3] World Health Organization. 2006.Neonatal and perinatal mortality: country, region and global estimates. Geneva. 2006.
[4] Woods R. Late-Fetal Mortality: Historical Perspectives on Continuing Problems of Estimation and Interpretation. Population (English Edition) 2008, 63 (4): 591-614

South Africa perinatal mortality surveillance

The South Africa perinatal mortality surveillance system includes the numbers of stillbirths and neonatal deaths for 1997-2014 in a 2015 report by Statistics South Africa. The perinatal mortality rate hovered between 18 and 22 per 1,000 births during 2002-2014 with no clear decline. The SB/ENN ratio of the numbers was 1.6 in the most recent year and ranged between 1.3 and 1.9 in the decade preceding 2014. Underreporting of both stillbirths and early NN deaths is possible but the degree cannot be established from the published report. We did not use the South African data to determine a cut-off value for the ratios.

Figure A.1 SB / ENN ratio, South Africa perinatal mortality surveillance system, 1997-2014 (PNM 18-22 per 1,000 births)

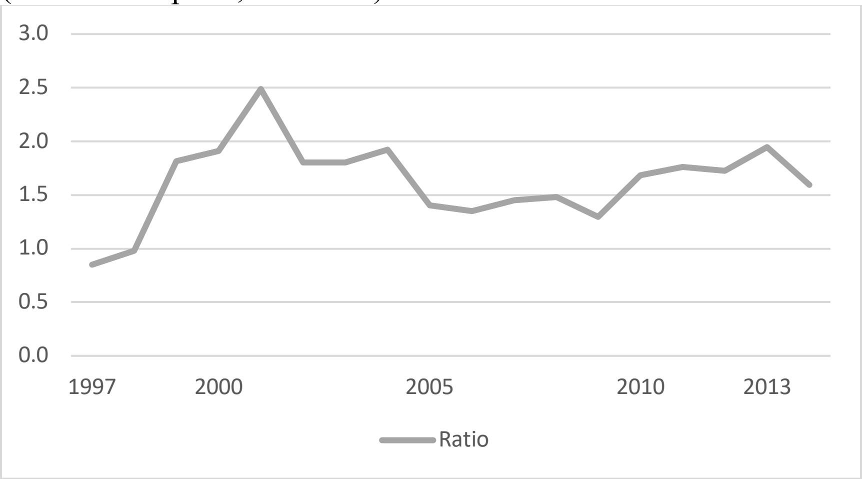

Longitudinal studies: AMANHI

The AMANHI multi-country study included 11 study sites in South Asia (5) and sub-Saharan Africa (6). Pregnant women were followed through birth and until 42 days after birth.[5] The South Asia studies (2 in India, 2 in Pakistan and 1 in Bangladesh) recorded the highest levels of stillbirth rates (overall 35 per 1,000 births (range (22-43)) and neonatal mortality (overall 43 per 1,000 live births (range 38-50)). The six sites in Africa were located in Kenya, Tanzania (2), DR Congo, Ghana and Zambia with an overall stillbirth date of 17 (range 8-27) and neonatal mortality of 20 (range 13-29). The Kenya site was an outlier with a stillbirth rate of 7.5 per 1,000 births and neonatal mortality of 12.8 per 1,000 live births.

[5] Alliance for Maternal and Newborn Health Improvement (AMANHI) mortality study group. Population-based rates, timing, and causes of maternal deaths, stillbirths, and neonatal deaths in south Asia and sub-Saharan Africa: a multi-country prospective cohort study. Lancet Glob Health. 2018 Dec;6(12):e1297-e1308. doi: 10.1016/S2214-109X(18)30385-1. Epub 2018 Oct 22. PMID: 30361107; PMCID: PMC6227247.

Table A2.4: Ratios of the age-specific mortality rates (median of the sites - range)

Ratio	Median (range)
Stillbirths to neonatal mortality	0.9 (0.5 – 1.6)
Stillbirth rate to first week mortality	1.2 (0.7 – 2.2)
Stillbirth rate to mortality day 0-1	1.6 (1.0 – 2.8)
Mortality 0-1 days / 2-6 days	2.4 (1.6 – 3.7)
Mortality 0-6 days / 7-27 days	3.7 (2.8 – 6.4)

There were several outliers but no clear association between the level of mortality and the ratios emerged. There were no systematic differences in the ratios between the South Asian and sub-Saharan Africa studies.

Global Network for Women's and Children's Health Research Maternal and Newborn Health Registry

This first part of this study was conducted 2010 through 2013. The seven Global Network sites were located in Argentina, Guatemala, Belgaum and Nagpur, India, Pakistan, Kenya, and Zambia. Pregnant women were enrolled if they resided in or receive healthcare in designated groups of communities within sites in the Global Network. Data were recorded at enrollment, the time of delivery and at 42 days postpartum. Several assessments showed that the data were of good quality.[6]

Perinatal mortality was 51 per 1,000 births and total late fetal and neonatal mortality was 56 per 1,000 births. Seven of the nine sites recorded perinatal mortality rates in the range of 34-46 per 1,000 births. Pakistan (99) and Argentina were outliers (22 per 1,000).[7] [8] [9]

Also, in these studies no clear association between the level of mortality and the ratios emerged. No systematic differences were observed in the ratios between the South Asian and sub-Saharan Africa studies.

Table A2.5: Ratios of the age-specific mortality rates (median - range):

---

[6] Goudar SS, Stolka KB, Koso-Thomas M, et al. Data quality monitoring and performance metrics of a prospective, population-based observational study of maternal and newborn health in low resource settings. Reprod Health. 2015;12 Suppl 2(Suppl 2):S2. doi: 10.1186/1742-4755-12-S2-S2. Epub 2015 Jun 8. PMID: 26062714; PMCID: PMC4464020.

[7] Saleem S, McClure EM, Goudar SS, et al. Global Network Maternal Newborn Health Registry Study Investigators. A prospective study of maternal, fetal and neonatal deaths in low- and middle-income countries. Bull World Health Organ. 2014 Aug 1;92(8):605-12. doi: 10.2471/BLT.13.127464. Epub 2014 Jun 5. PMID: 25177075; PMCID:

[8] Belizán JM, McClure EM, Goudar SS, Pasha O, Esamai F, Patel A et al. Neonataldeath in low- to middle-income countries: a global network study. Am J Perinatol 2012; 29: 649–656.

[9] Patel AB, Simmons EM, Rao SR, et al. Evaluating the effect of care around labor and delivery practices on early neonatal mortality in the Global Network's Maternal and Newborn Health Registry. Reprod Health. 2020 Nov 30;17(Suppl 2):156.

Ratio	All sites (range)
Stillbirths to neonatal mortality	1.1 (0.9 – 1.4)
Stillbirth rate to first week mortality	1.4 (1.3 – 1.8)
Stillbirth rate to mortality day 0-1[7]	2.3 (not available)
Mortality 0-1 days / 2-6 days	1.7 (0.7 – 2.9)
Mortality 0-6 days / 7-27 days	4.2 (2.6 – 5.1)

Data collection continued in six sites during 2013-2018 (excluding Argentina) and results were used to compute stillbirth / neonatal mortality ratios for 2013-2018 based on published data: the median was 1.0, with a range from 0.8 – 1.6 in the six studies.[10]

Other prospective studies

We screened the literature for prospective studies with stillbirth and neonatal mortality data broken down by age within the neonatal period. We included studies that met or were close to the following criteria: (1) initiation of follow up before 7 months of pregnancy (2) follow up until the end of the first week or ideally end of the first month of life (3) intensive follow up (weekly in most instances, monthly during pregnancy) (4) large sample size (more than 100 perinatal deaths) (5) community component included (6) no intervention trial.

Only few studies met these criteria. The community-based Health Demographic Surveillance Systems (HDSS) could be a major source of stillbirth and neonatal deaths but in many settings the frequency of follow-up is not high enough to ascertain high quality data. Furthermore, the use of proxy respondents (mostly the head of the households) may further compromise the completeness of reporting in HDSS settings. A review of HDSS results in 13 HDSS sites showed the wide variety in methods and results with often implausible results[11]. The results were not included here.

Conclusion

For the purpose of this paper, we opted for constant reference values for the data quality metrics and adjustment model. We used vital registration data for the STB/D01 and took the midpoint between the ratio of the developed countries (2.10) and low ad middle income countries (1.68), which is 1.89. For the ratio days 0-1 to days 2-6, we took the AMANHI median value of 2.4 as reference value. These are considered the cut-off values for plausible ratio values. The cut-offs for probably and improbable values were based on the general presented in this review.

[10] Goudar SS, Goco N, Somannavar MS, et al. Institutional deliveries and stillbirth and neonatal mortality in the Global Network's Maternal and Newborn Health Registry. Reprod Health. 2020 Dec 17;17(Suppl 3):179.

[11] Waiswa P, Akuze J, Moyer C, Kwesiga D, Arthur S, Sankoh O, Welaga P, Bangha M, Eminas J, Muuo S, Ziraba A, Kerber K; INDEPTH Network MNCH team. Status of birth and pregnancy outcome capture in Health Demographic Surveillance Sites in 13 countries. Int J Public Health. 2019 Jul;64(6):909-920. doi: 10.1007/s00038-019-01241-0. Epub 2019 Jun 26. PMID: 31240333; PMCID: PMC6614155.

Table A2.6: Ratios of the age-specific mortality rates (median - range):

Ratio	B'adesh Matlab[12]	B'desh, gov.	India[13]	Pakistan[14]	Malawi[15]	DR Congo[16]	6-country study[17]
Stillbirths to neonatal mortality			0.8	0.8	0.9		
Stillbirth rate to 1st week mortality	1.4	1.2	1.0	1.1	1.1	1.0	1.4
Stillbirth rate to day 0-1			1.7				
Mortality 0-1 days / 2-6 days			1.5				
Mortality 0-6 days / 7-27 days			3.0	3.0	5.5		

Conclusion

There is no large volume of studies upon which ratios can be based for LMIC with higher levels of perinatal and neonatal mortality. In general, there appears to be a lack of an association of the stillbirth neonatal death ratios with the levels of mortality as long as perinatal mortality is above 10 per 1,000 births. For deaths within the neonatal period there is a greater drop in mortality in the later neonatal period (2-4 weeks) compared to the earlier neonatal period (first week) by level of mortality.

There is substantive variation between studies which is likely a reflection of variation in relation to demographic factors (fertility and parity distribution, incidence of multiple pregnancies, consanguinity and marriage), epidemiology (such as malaria), home and health facility delivery practices, etc.

---

[12] Rahman A, Moran A, Pervin J, Rahman A, Rahman M, Yeasmin S, Begum H, Rashid H, Yunus M, Hruschka D, Arifeen SE, Streatfield PK, Sibley L, Bhuiya A, Koblinsky M. Effectiveness of an integrated approach to reduce perinatal mortality: recent experiences from Matlab, Bangladesh. BMC Public Health. 2011 Dec 10;11:914. doi: 10.1186/1471-2458-11-914. PMID: 22151276; PMCID: PMC3257323.

[13] Bapat U, Alcock G, More NS, Das S, Joshi W, Osrin D. Stillbirths and newborn deaths in slum settlements in Mumbai, India: a prospective verbal autopsy study. BMC Pregnancy Childbirth 2012; 12: 39.

[14] Anwar J, Torvaldsen S, Sheikh M, Taylor R. Under-estimation of maternal and perinatal mortality revealed by an enhanced surveillance system: enumerating all births and deaths in Pakistan. BMC Public Health. 2018 Apr 2;18(1):428. doi: 10.1186/s12889-018-5363-3. PMID: 29609571; PMCID: PMC5880001.

[15] Colbourn T, Nambiar B, Bondo A, Makwenda C, Tsetekani E, Makonda-Ridley A, Msukwa M, Barker P, Kotagal U, Williams C, Davies R, Webb D, Flatman D, Lewycka S, Rosato M, Kachale F, Mwansambo C, Costello A. Effects of quality improvement in health facilities and community mobilization through women's groups on maternal, neonatal and perinatal mortality in three districts of Malawi: MaiKhanda, a cluster randomized controlled effectiveness trial. Int Health. 2013 Sep;5(3):180-95. doi: 10.1093/inthealth/iht011. Epub 2013 Jun 26. PMID: 24030269; PMCID: PMC5102328.

[16] Engmann C, Matendo R, Kinoshita R, Ditekemena J, Moore J, Goldenberg RL, Tshefu A, Carlo WA, McClure EM, Bose C, Wright LL. Stillbirth and early neonatal mortality in rural Central Africa. Int J Gynaecol Obstet. 2009 May;105(2):112-7. doi: 10.1016/j.ijgo.2008.12.012. Epub 2009 Feb 7. PMID: 19201402; PMCID: PMC3972762.

[17] Ngoc NT, Merialdi M, Abdel-Aleem H, Carroli G, Purwar M, Zavaleta N et al. Causes of stillbirths and early neonatal deaths: data from 7993 pregnancies in six developing countries. Bull World Health Organ 2006; 84: 699–705.

There is however some uniformity in the results that allows the selection of reference values. The median ratio stillbirth to neonatal deaths was 0.8 (IQR: 0.6-1.0) based on 25 data points. The median ratio stillbirths to first week deaths was 1.2 (1.1-1.4) based on 26 data points.

The median was 1.6 (1.2-1.8) for stillbirths to deaths on day 0-1 was 1.7 (1.5-2.3) and for neonatal mortality on day 0-1 to day 2-6 2.1 (1.7-2.4). Our primary concern is omission of stillbirths and very early neonatal deaths (0-1 days). We consider surveys with SB to day 0-1 ratios below 1.7 as having moderate evidence of omission and those with ratios below 1.2 as severe omission of stillbirths. For neonatal deaths we consider surveys with ratios below 2.1 as having moderate omission of very early neonatal deaths, while those with ratios below 1.7 as having severe omission, all based on the median and first quartile of the distribution of the studies reviewed.

Appendix 3: The DHS surveys and the statistical model used

The DHS Survey

DHS is the major data source for pregnancy outcomes worldwide. From 1984 to date, the DHS programme had seven phases (DHS-I to DHS-VIII) collecting data from more than 400 surveys in more than 90 countries. Throughout all DHS phases, the model questionnaire included a Full birth history (FBH), capturing a woman's lifetime live births and survival status, which most countries have implemented to calculate early childhood mortality. FBH has undergone minor changes during the last three decades. Stillbirths were initially not captured or reported in DHS-I. In DHS-II to DHS VIII, reproductive calendars were used to collect data on pregnancy outcomes including abortion and stillbirth. Since DHS-III, stillbirth rates have been shown in the standard national DHS tabulation. DHS-VII introduced a reverse truncated history for non-live births, and in DHS-VIII, the full birth history module is replaced by a full pregnancy history module.

The DHS reproductive calendar

The reproductive calendar includes a "month-by-month" complete history of women's reproduction and contraceptive use for a period of between 5 and 7 years prior to the survey. The exact length of the period covered by the calendar varies depending on the defined recall period and duration of the survey data collection. In most surveys, the period covered by the calendar includes the months up to the month of interview in the year of interview, plus the five calendar years preceding the year of interview.

Piece-wise exponential (PWE) and modified Gompertz-Makeham models

The DHS reproductive calendar records the pregnancy loss in months and the birth history module records the child death in days, months or years, therefore the exact gestational age at pregnancy loss or age at death are not reported, which gives rise to grouped or interval censored data.

If p_{ij} is the probability of pregnancy loss or child death reported for the ith pregnancy/child, where $i = 1, 2, \ldots, n$, at j^{th} time interval, $j = 1, 2, \ldots, k$. Also let π_{ij} be the probability that the ith of n pregnancies/children is still alive at time interval t_{j-1} and has experienced the loss or death in the jth time interval, $j = 1, 2, \ldots, k$. This is therefore, the conditional probability of pregnancy loss or child death in the jth time interval, given that loss/death occurs after t_{j-1}.

Using T_i to denote the random variable associated with the event time of the ith of pregnancy/child, we therefore have

$$p_{ij} = P(t_{j-1} \leq T_i < t_j)$$

and

$$\pi_{ij} = P(t_{j-1} \leq T_i < t_j | T_i \geq t_{j-1})$$

For $j = 1, 2, \ldots, k$

$$1 - \pi_{ij} = P\left(T_i \geq t_j \middle| T_i \geq t_{j-1}\right) = \frac{S_i(t_j)}{S_i\left(t_{j-1}\right)}$$

Adopting a proportional hazards model for the event times, the hazard of loss or death being reported at time t_j for the ith pregnancy/child can be expressed

$$h_i\left(t_j\right) = \exp\left(\eta_i\right) h_0(t_j)$$

$$1 - \pi_{ij} = \left[\frac{S_0(t_j)}{S_0\left(t_{j-1}\right)}\right]^{\exp(\eta_i)}$$

$$\begin{aligned} log\{-\log(1 - \pi_{ij})\} &= \eta_i + log\left[-log\{S_0(t_j)/S_0(t_{j-1})\}\right] \\ &= \eta_i + \gamma_j \\ &= \beta X + \gamma_1 + \gamma_2 + \gamma_3 + \cdots + \gamma_k \end{aligned}$$

This is a linear model for the complementary log-log transformation of π_{ij}, in which the parameters γ_j , $j = 1, 2, \ldots, k$, are associated with the k time intervals. Since no covariate is included, the right-hand side is PWE (i.e., fitting a parameter for each time interval) (1)

$$= \gamma_1 + \gamma_2 + \gamma_3 + \cdots + \gamma_k \tag{1}$$

Then the modified Gompertz-Makeham model parametrization is (2)

$$\gamma_j = \begin{cases} \gamma_1 & if \quad j = 1 \\ \gamma_2 & if \quad j = 2 \\ \gamma_0 + \gamma_j & if \quad j \geq 3 \end{cases} \tag{2}$$

Reference

1. Collett, D. (2014). *Modelling survival data in medical research (3rd ed.)*: Chapter 9: Interval-censored Survival data p319-344. Chapman & Hall/CRC.

2. Albert W. Marshall Ingram Olkin (2007). *Life Distributions: Structure of Nonparametric, Semiparametric, and Parametric Families.* Chapter 10: Gompertz and Gompertz–Makeham Distributions p363-398. Springer Series in Statistics.

Appendix 4: Number of pregnancies

Table A4: Number of pregnancies with 7+ months gestation, pregnancy outcome (unweighted), by country and survey round

Country name	Survey round	Births and pregnancies of 7+ months gestation	Stillbirth	Currently pregnant	Live births
Angola	2015/16	14,392	101	494	13,797
Benin	2006	16,419	202	605	15,612
Benin	2011/12	13,358	79	424	12,855
Benin	2017/18	13,514	112	487	12,915
Burkina Faso	2003	10,909	158	405	10,346
Burkina Faso	2010	15,398	176	561	14,661
Burundi	2010/11	8,022	141	318	7,563
Ethiopia	2005	10,197	97	444	9,656
Ethiopia	2010/11	11,857	165	458	11,234
Ethiopia	2016	10,855	113	442	10,300
Gambia	2013	7,972	95	322	7,555
Gambia	2019/20	8,533	144	370	8,019
Ghana	2003	3,967	39	164	3,764
Ghana	2008	3,074	41	112	2,921
Ghana	2014	6,004	73	220	5,711
Guinea	2005	6,219	88	306	5,825
Guinea	2018	7,975	107	270	7,598
Kenya	1998	5,586	53	194	5,339
Kenya	2003	6,020	86	241	5,693
Kenya	2008/9	6,149	57	203	5,889
Kenya	2014	10,232	131	330	9,771
Lesotho	2009/10	4,033	66	122	3,845
Lesotho	2014	3,188	57	111	3,020
Liberia	2013	7,404	84	320	7,000
Liberia	2019/20	5,777	76	226	5,475
Madagascar	2003/4	5,550	62	219	5,269
Madagascar	2008/9	12,577	166	459	11,952
Malawi	2000	12,372	156	555	11,661
Malawi	2004/5	11,214	169	489	10,556
Malawi	2010	20,104	302	703	19,099
Malawi	2015/16	17,387	203	658	16,526
Mali	2001	12,755	173	594	11,988
Mali	2006	14,644	169	615	13,860
Mali	2012/13	10,398	67	337	9,994
Mali	2018	10,150	130	360	9,660
Mozambique	2003/4	10,702	202	406	10,094
Mozambique	2011	11,448	130	515	10,803
Namibia	2006/7	5,295	45	232	5,018
Namibia	2013	5,112	33	202	4,877
Niger	2006	9,089	114	417	8,558
Niger	2012	12,743	208	530	12,005

Nigeria	2008	29,416	233	1,228	27,955
Nigeria	2013	31,486	401	1,597	29,488
Nigeria	2018	34,904	571	1,446	32,887
Rwanda	2000	8,149	138	330	7,681
Rwanda	2005	8,725	153	341	8,231
Rwanda	2010/11	9,147	147	292	8,708
Rwanda	2014/15	8,046	122	293	7,631
Rwanda	2019/20	8,180	111	278	7,791
Senegal	2005	10,980	220	408	10,352
Senegal	2010/11	12,414	212	468	11,734
Senegal	2015	6,998	163	256	6,579
Senegal	2016	6,824	129	252	6,443
Senegal	2018	6,839	143	296	6,400
Senegal	2019	6,271	126	273	5,872
Sierra Leone	2008	5,747	46	239	5,462
Sierra Leone	2013	12,245	95	538	11,612
South Africa	2016	3,509	42	128	3,339
Eswatini	2006/7	2,891	39	108	2,744
Tanzania	2004/5	8,875	185	383	8,307
Tanzania	2009/10	7,804	137	334	7,333
Tanzania	2015/16	10,415	213	354	9,848
Uganda	2000/1	7,327	128	290	6,909
Uganda	2006	8,441	123	348	7,970
Uganda	2011	8,117	144	333	7,640
Zambia	2007	6,538	86	267	6,185
Zambia	2013/14	13,697	179	480	13,038
Zambia	2018/19	10,108	133	392	9,583
Zimbabwe	1994	4,169	57	166	3,946
Zimbabwe	1999	3,702	50	143	3,509
Zimbabwe	2005/6	5,305	42	197	5,066
Zimbabwe	2010/11	5,715	72	265	5,378
Zimbabwe	2015	6,125	78	203	5,844
Azerbaijan	2006	2,342	28	97	2,217
Egypt	1992/93	8,968	138	394	8,436
Egypt	1995/96	12,420	201	514	11,705
Egypt	2000	11,182	164	359	10,659
Egypt	2003	6,725	80	227	6,418
Egypt	2005	13,788	134	469	13,185
Egypt	2008	10,719	89	398	10,232
Egypt	2014	15,817	95	585	15,137
Jordan	1990	8,571	98	388	8,085
Jordan	1997	6,578	57	252	6,269
Jordan	2002	6,213	59	270	5,884
Jordan	2007	10,704	64	506	10,134
Jordan	2009	9,847	94	404	9,349
Jordan	2012	10,416	70	363	9,983
Jordan	2017/18	10,731	52	548	10,131
Morocco	1992	5,339	110	217	5,012
Morocco	2003/4	6,322	99	247	5,976
Türkiye	1993	3,834	64	155	3,615

Türkiye	1998	3,670	60	157	3,453
Türkiye	2003/4	4,594	53	160	4,381
Yemen	2013	16,713	253	715	15,745
Tajikistan	2012	5,196	40	286	4,870
Tajikistan	2017	6,286	46	299	5,941
Bangladesh	1993/94	7,344	194	369	6,781
Bangladesh	1996/97	6,472	196	303	5,973
Bangladesh	1999/0	7,125	197	289	6,639
Bangladesh	2004	7,216	258	232	6,726
Bangladesh	2007	6,262	166	224	5,872
Bangladesh	2011	9,006	225	373	8,408
Bangladesh	2014	8,180	178	371	7,631
Bangladesh	2017/18	8,981	215	363	8,403
Cambodia	2010/11	8,393	68	347	7,978
Cambodia	2014	7,282	36	341	6,905
India	2005/6	52,058	847	2,150	49,061
India	2015/16	256,211	3,147	9,806	243,258
India	2019/21	230,166	2,542	8,984	218,640
Indonesia	1991	15,982	119	651	15,212
Indonesia	1994	18,625	170	787	17,668
Indonesia	1997	17,780	154	688	16,938
Indonesia	2002/3	16,544	157	637	15,750
Indonesia	2007	18,927	176	694	18,057
Indonesia	2012	18,180	204	744	17,232
Indonesia	2017	18,235	176	770	17,289
Maldives	2009	3,887	29	156	3,702
Myanmar	2015/16	4,899	50	182	4,667
Nepal	2006	5,917	139	187	5,591
Nepal	2011	5,356	69	174	5,113
Nepal	2016	4,992	71	187	4,734
Pakistan	2012/13	12,220	341	504	11,375
Pakistan	2017/18	10,790	248	444	10,098
Philippines	1993	9,199	88	348	8,763
Philippines	1998	7,911	101	336	7,474
Philippines	2003	7,250	78	276	6,896
Timor-Leste	2009/10	9,905	26	330	9,549
Bolivia	1993/94	5,870	65	245	5,560
Bolivia	2003/4	10,535	113	356	10,066
Bolivia	2008	8,465	91	327	8,047
Brazil	1991/92	3,213	50	110	3,053
Brazil	1996	4,969	45	177	4,747
Colombia	1995	5,138	47	191	4,900
Colombia	2000	4,658	54	195	4,409
Colombia	2004/5	14,650	103	501	14,046
Colombia	2009/10	17,606	109	634	16,863
Colombia	2015/16	11,410	83	407	10,920
Dominican Republic	1991	4,263	72	162	4,029
Dominican Republic	1996	4,797	112	201	4,484

Dominican Republic	2002	11,502	123	432	10,947
Guatemala	1995	10,165	122	410	9,633
Guatemala	1998/99	5,085	100	208	4,777
Guatemala	2014/15	12,660	126	475	12,059
Honduras	2005/6	10,933	143	392	10,398
Honduras	2011/12	11,060	118	439	10,503
Nicaragua	1997/98	8,133	61	289	7,783
Nicaragua	2001	7,099	72	268	6,759
Paraguay	1990	4,353	78	170	4,105
Peru	1991/92	9,472	93	334	9,045
Peru	1996	17,789	186	585	17,018
Peru	2000	13,812	129	490	13,193
Peru	2004/6	7,830	69	328	7,433
Peru	2007/8	9,226	109	397	8,720
Peru	2009	10,249	89	374	9,786
Peru	2010	9,180	70	335	8,775
Peru	2011	9102	92	323	8687
Peru	2012	9610	73	361	9176

Appendix 5: Data quality indicators

Table A5: Number of deaths by time of death, data quality indicators and thresholds, by country and survey

Country Name	Survey fieldwork	Stillbirth	D0-1	D0-2	D2-6	D3-6	ENM	LNM	NNM
Angola	2015/16	101	235	257	48	27	284	25	309
Benin	2006	202	234	270	147	111	381	92	472
Benin	2011/12	80	158	178	59	39	217	47	264
Benin	2017/18	115	212	238	93	67	305	57	362
Burkina Faso	2003	160	119	148	89	60	208	94	302
Burkina Faso	2010	172	157	210	127	74	284	117	401
Burundi	2010/11	155	104	134	65	35	169	69	238
Ethiopia	2005	118	198	242	101	58	299	127	426
Ethiopia	2010/11	204	236	257	102	82	339	86	425
Ethiopia	2016	127	185	195	48	37	233	68	301
Gambia	2013	89	86	96	53	43	139	17	156
Gambia	2019/20	146	97	120	61	38	158	43	201
Ghana	2003	40	80	86	46	40	126	26	152
Ghana	2008	40	49	52	22	19	71	13	84
Ghana	2014	80	109	117	26	18	135	19	154
Guinea	2005	98	81	118	82	45	163	58	221
Guinea	2018	106	134	153	56	37	190	46	236
Kenya	1998	53	80	93	34	21	114	28	142
Kenya	2003	84	104	123	52	33	156	35	191
Kenya	2008/9	68	93	110	43	25	135	32	168
Kenya	2014	125	94	122	50	22	144	63	207
Lesotho	2009/10	61	91	103	29	17	120	30	150
Lesotho	2014	69	47	56	23	14	70	15	86
Liberia	2013	70	74	94	46	25	119	40	159
Liberia	2019/20	61	124	143	34	15	158	30	188
Madagascar	2003/4	85	96	107	34	24	131	57	188
Madagascar	2008/9	167	158	182	75	51	233	59	291
Malawi	2000	158	201	245	133	89	334	146	480
Malawi	2004/5	163	136	161	63	38	199	76	275
Malawi	2010	312	328	363	114	79	442	136	578
Malawi	2015/16	233	241	277	86	49	326	74	400
Mali	2001	162	303	351	158	110	461	208	669
Mali	2006	193	298	358	186	126	484	134	618
Mali	2012/13	64	221	239	60	43	282	45	327
Mali	2018	123	157	188	105	74	262	52	315
Mozambique	2003/4	206	105	136	130	99	235	130	365
Mozambique	2011	126	244	279	66	30	310	35	345
Namibia	2006/7	46	70	79	23	14	93	16	109
Namibia	2013	38	56	63	15	8	72	16	88
Niger	2006	123	100	131	90	59	190	102	293
Niger	2012	225	142	176	82	48	224	83	307
Nigeria	2008	227	560	646	285	199	845	230	1075

Nigeria	2013	396	524	644	330	211	854	236	1091
Nigeria	2018	595	772	881	300	191	1071	225	1297
Rwanda	2000	148	165	190	76	50	241	93	333
Rwanda	2005	160	148	167	62	43	210	78	289
Rwanda	2010/11	153	109	127	52	34	161	77	238
Rwanda	2014/15	122	65	82	44	28	110	41	150
Rwanda	2019/20	108	79	94	34	20	113	27	140
Senegal	2005	215	135	157	105	83	240	83	323
Senegal	2010/11	190	164	181	70	54	234	60	295
Senegal	2015	126	42	51	53	44	95	39	134
Senegal	2016	133	37	53	61	46	98	26	124
Senegal	2018	108	63	78	51	36	114	20	134
Senegal	2019	120	26	45	60	40	85	26	111
Sierra Leone	2008	49	92	111	51	33	144	58	202
Sierra Leone	2013	98	215	262	149	102	364	85	449
South Africa	2016	37	41	42	8	6	49	8	57
Eswatini	2006/7	35	30	37	18	11	48	12	60
Tanzania	2004/5	163	142	169	56	28	198	61	259
Tanzania	2009/10	143	99	115	48	31	146	48	195
Tanzania	2015/16	180	133	157	75	51	208	34	242
Uganda	2000/1	125	132	150	45	27	177	66	243
Uganda	2006	135	105	115	49	39	154	47	201
Uganda	2011	162	114	136	45	23	159	50	208
Zambia	2007	91	93	108	54	38	147	61	208
Zambia	2013/14	175	161	200	83	45	244	72	317
Zambia	2018/19	117	153	174	51	30	204	60	265
Zimbabwe	1994	57	36	43	26	18	62	25	86
Zimbabwe	1999	53	58	66	15	6	73	23	96
Zimbabwe	2005/6	40	64	69	22	17	86	31	117
Zimbabwe	2010/11	85	87	99	31	19	118	28	146
Zimbabwe	2015	77	78	98	40	19	118	43	161
Azerbaijan	2006	37	37	44	16	9	53	14	67
Egypt	1992/93	139	92	113	78	57	170	107	278
Egypt	1995/96	167	109	139	103	74	212	126	338
Egypt	2000	166	93	116	75	52	168	77	245
Egypt	2003	67	55	65	22	13	77	59	137
Egypt	2005	129	96	120	78	54	174	75	249
Egypt	2008	87	57	69	49	37	106	49	155
Egypt	2014	104	66	82	60	44	126	74	200
Jordan	1990	98	73	87	48	34	121	46	167
Jordan	1997	51	54	61	28	22	82	36	118
Jordan	2002	56	27	42	42	27	69	22	91
Jordan	2007	57	44	54	37	28	82	45	126
Jordan	2009	81	63	66	22	19	84	50	134
Jordan	2012	49	55	77	51	29	106	21	127
Jordan	2017/18	46	37	51	43	30	80	23	103
Morocco	1992	110	50	57	42	35	92	65	157
Morocco	2003/4	99	69	74	41	36	110	43	153
Türkiye	1993	64	46	54	28	19	74	25	99
Türkiye	1998	55	38	52	38	24	76	11	87

Türkiye	2003/4	44	30	34	14	10	44	19	63
Yemen	2013	249	186	222	126	90	312	84	396
Tajikistan	2012	43	40	51	39	28	79	23	102
Tajikistan	2017	47	32	34	28	25	59	14	73
Bangladesh	1993/94	200	133	160	91	64	225	124	349
Bangladesh	1996/97	194	96	103	71	64	167	102	269
Bangladesh	1999/0	194	125	136	74	63	199	79	278
Bangladesh	2004	262	136	150	68	55	204	78	282
Bangladesh	2007	175	109	121	47	36	157	53	210
Bangladesh	2011	226	131	148	80	63	211	50	261
Bangladesh	2014	175	107	118	74	63	181	40	221
Bangladesh	2017/18	219	119	137	75	56	194	48	241
Cambodia	2010/11	72	116	134	53	35	170	40	209
Cambodia	2014	42	73	85	24	12	97	23	119
India	2005/6	1094	1003	1131	593	464	1596	469	2065
India	2015/16	3092	3876	4324	1662	1214	5538	1128	6666
India	2019/21	2644	2951	3281	1379	1049	4330	910	5241
Indonesia	1991	123	179	194	85	70	264	174	438
Indonesia	1994	187	227	256	114	85	341	145	486
Indonesia	1997	161	180	202	64	42	244	98	342
Indonesia	2002/3	145	147	160	69	56	216	63	280
Indonesia	2007	172	170	193	62	40	232	65	298
Indonesia	2012	181	171	198	75	48	246	48	294
Indonesia	2017	149	140	158	68	51	209	43	252
Maldives	2009	32	26	27	5	4	32	3	34
Myanmar	2015/16	48	51	63	23	11	75	18	93
Nepal	2006	126	74	92	53	35	127	51	178
Nepal	2011	53	89	102	54	42	144	25	169
Nepal	2016	78	51	55	17	13	68	21	89
Pakistan	2012/13	396	324	372	188	141	512	133	645
Pakistan	2017/18	240	188	222	156	122	344	76	420
Philippines	1993	79	70	87	40	23	110	30	140
Philippines	1998	93	51	63	48	36	99	20	119
Philippines	2003	75	54	61	33	26	87	28	115
Timor-Leste	2009/10	22	100	113	53	39	152	44	196
Bolivia	1993/94	72	82	96	33	20	116	70	185
Bolivia	2003/4	123	109	127	54	36	163	75	238
Bolivia	2008	98	87	95	52	44	139	69	207
Brazil	1991/92	47	35	37	19	16	54	31	85
Brazil	1996	44	44	47	18	15	62	13	75
Colombia	1995	48	40	49	28	19	68	17	85
Colombia	2000	53	38	41	11	9	50	13	63
Colombia	2004/5	91	61	77	40	23	100	25	126
Colombia	2009/10	85	45	51	27	21	72	43	115
Colombia	2015/16	82	34	36	12	10	46	14	60
Dominican Republic	1991	63	39	44	23	18	62	23	85
Dominican Republic	1996	85	50	58	32	24	82	29	110
Dominican Republic	2002	87	104	131	49	22	153	43	196
Guatemala	1995	101	130	143	42	29	172	49	221
Guatemala	1998/99	75	61	64	12	9	73	29	102

Guatemala	2014/15	129	72	83	50	40	122	64	186
Honduras	2005/6	124	59	66	36	28	95	31	125
Honduras	2011/12	97	56	64	26	18	81	32	113
Nicaragua	1997/98	59	57	65	34	26	91	27	118
Nicaragua	2001	63	40	44	32	28	72	31	103
Paraguay	1990	68	37	43	18	12	55	17	72
Peru	1991/92	81	90	109	50	31	140	66	206
Peru	1996	147	182	198	66	51	249	116	365
Peru	2000	113	86	107	43	22	129	65	194
Peru	2004/6	51	44	48	8	4	52	17	69
Peru	2007/8	104	31	36	16	10	47	22	69
Peru	2009	80	32	37	25	20	57	31	88
Peru	2010	51	20	25	26	21	46	8	54
Peru	2011	69	21	25	14	11	35	14	50
Peru	2012	69	33	39	25	19	59	11	70

D0-1 Deaths between day 0-1
D0-2 Deaths between day 0-2
D2-6 Deaths between day 2-6
D3-6 Deaths between day 3-6
ENM Early Neonatal day 0-6
LNM Late Neonatal day 7-27
NNM Neonatal day 0-27

Table A5 (*cont.*)

Country Name	Survey fieldwork	STB/ D0-1	STB/ D0-2	STB/ ENM	STB/ NNM	D0/D1	D0-1/ D2-6	D0-2/ D3-6
Angola	2015/16	0.43	0.39	0.36	0.33	2.41	4.86	9.45
Benin	2006	0.86	0.75	0.53	0.43	1.03	1.59	2.43
Benin	2011/12	0.51	0.45	0.37	0.31	2.54	2.68	4.57
Benin	2017/18	0.54	0.48	0.38	0.32	1.57	2.27	3.56
Burkina Faso	2003	1.35	1.09	0.77	0.53	1.44	1.34	2.45
Burkina Faso	2010	1.09	0.82	0.61	0.43	1.07	1.24	2.82
Burundi	2010/11	1.49	1.16	0.92	0.65	1.88	1.61	3.89
Ethiopia	2005	0.60	0.49	0.40	0.28	0.72	1.95	4.20
Ethiopia	2010/11	0.86	0.79	0.60	0.48	3.14	2.31	3.15
Ethiopia	2016	0.69	0.65	0.55	0.42	1.79	3.87	5.21
Gambia	2013	1.03	0.92	0.64	0.57	3.67	1.61	2.24
Gambia	2019/20	1.51	1.22	0.93	0.73	3.74	1.58	3.12
Ghana	2003	0.50	0.46	0.32	0.26	0.58	1.76	2.17
Ghana	2008	0.82	0.78	0.57	0.48	0.49	2.21	2.70
Ghana	2014	0.73	0.69	0.60	0.52	0.43	4.28	6.63
Guinea	2005	1.21	0.83	0.60	0.44	0.60	0.99	2.61
Guinea	2018	0.79	0.70	0.56	0.45	1.94	2.41	4.14
Kenya	1998	0.66	0.56	0.46	0.37	5.24	2.38	4.49
Kenya	2003	0.81	0.68	0.54	0.44	2.69	2.01	3.75
Kenya	2008/9	0.74	0.62	0.50	0.41	2.65	2.16	4.44
Kenya	2014	1.32	1.02	0.87	0.60	2.16	1.89	5.62
Lesotho	2009/10	0.67	0.59	0.51	0.41	2.86	3.09	6.04
Lesotho	2014	1.46	1.23	0.98	0.81	1.08	2.08	3.95
Liberia	2013	0.94	0.74	0.58	0.44	0.67	1.62	3.78
Liberia	2019/20	0.49	0.43	0.39	0.32	4.39	3.64	9.64
Madagascar	2003/4	0.88	0.79	0.65	0.45	1.27	2.83	4.49
Madagascar	2008/9	1.05	0.92	0.72	0.57	1.49	2.12	3.55
Malawi	2000	0.79	0.65	0.47	0.33	1.60	1.52	2.74
Malawi	2004/5	1.20	1.01	0.82	0.59	1.61	2.14	4.27
Malawi	2010	0.95	0.86	0.71	0.54	1.53	2.86	4.56
Malawi	2015/16	0.97	0.84	0.71	0.58	2.34	2.80	5.65
Mali	2001	0.53	0.46	0.35	0.24	1.82	1.92	3.18
Mali	2006	0.65	0.54	0.40	0.31	0.69	1.60	2.85
Mali	2012/13	0.29	0.27	0.23	0.20	2.63	3.67	5.59
Mali	2018	0.78	0.65	0.47	0.39	2.31	1.50	2.54
Mozambique	2003/4	1.96	1.51	0.87	0.56	1.10	0.80	1.37
Mozambique	2011	0.52	0.45	0.41	0.37	2.40	3.70	9.19
Namibia	2006/7	0.65	0.58	0.49	0.42	0.72	3.01	5.59

Namibia	2013	0.67	0.59	0.53	0.43	0.76	3.64	7.74
Niger	2006	1.24	0.94	0.65	0.42	1.69	1.10	2.25
Niger	2012	1.58	1.27	1.00	0.73	0.45	1.73	3.69
Nigeria	2008	0.41	0.35	0.27	0.21	1.51	1.97	3.25
Nigeria	2013	0.76	0.62	0.46	0.36	0.89	1.59	3.06
Nigeria	2018	0.77	0.68	0.56	0.46	2.36	2.58	4.62
Rwanda	2000	0.90	0.78	0.61	0.44	2.87	2.16	3.77
Rwanda	2005	1.08	0.95	0.76	0.55	5.17	2.39	3.89
Rwanda	2010/11	1.41	1.21	0.95	0.64	2.37	2.10	3.71
Rwanda	2014/15	1.87	1.49	1.11	0.81	1.84	1.47	2.95
Rwanda	2019/20	1.36	1.15	0.95	0.77	7.75	2.34	4.79
Senegal	2005	1.60	1.37	0.90	0.67	1.59	1.28	1.90
Senegal	2010/11	1.15	1.05	0.81	0.64	1.31	2.34	3.35
Senegal	2015	2.99	2.45	1.32	0.94	0.69	0.79	1.16
Senegal	2016	3.61	2.53	1.35	1.07	0.54	0.60	1.15
Senegal	2018	1.72	1.38	0.95	0.81	0.84	1.23	2.17
Senegal	2019	4.72	2.66	1.41	1.09	0.60	0.43	1.13
Sierra Leone	2008	0.53	0.45	0.34	0.24	1.60	1.80	3.35
Sierra Leone	2013	0.45	0.37	0.27	0.22	0.69	1.44	2.56
South Africa	2016	0.92	0.88	0.76	0.65	3.28	5.00	6.59
Eswatini	2006/7	1.15	0.95	0.73	0.58	0.86	1.72	3.25
Tanzania	2004/5	1.15	0.96	0.83	0.63	2.20	2.56	5.97
Tanzania	2009/10	1.45	1.24	0.98	0.73	1.34	2.06	3.66
Tanzania	2015/16	1.35	1.14	0.86	0.74	2.02	1.77	3.07
Uganda	2000/1	0.94	0.83	0.70	0.51	2.20	2.93	5.57
Uganda	2006	1.29	1.18	0.88	0.67	2.85	2.14	2.91
Uganda	2011	1.42	1.19	1.02	0.78	1.61	2.51	5.93
Zambia	2007	0.98	0.84	0.62	0.44	2.07	1.73	2.82
Zambia	2013/14	1.08	0.87	0.71	0.55	1.77	1.94	4.49
Zambia	2018/19	0.76	0.67	0.57	0.44	3.62	3.01	5.84
Zimbabwe	1994	1.59	1.31	0.92	0.66	1.96	1.38	2.38
Zimbabwe	1999	0.91	0.80	0.73	0.55	1.32	3.90	10.27
Zimbabwe	2005/6	0.62	0.58	0.46	0.34	3.59	2.88	4.08
Zimbabwe	2010/11	0.98	0.86	0.72	0.58	4.13	2.85	5.18
Zimbabwe	2015	0.99	0.78	0.65	0.48	1.87	1.94	5.03
Azerbaijan	2006	0.99	0.85	0.70	0.55	0.89	2.34	4.64
Egypt	1992/93	1.51	1.23	0.82	0.50	0.93	1.17	1.98
Egypt	1995/96	1.53	1.21	0.79	0.50	1.04	1.06	1.88
Egypt	2000	1.78	1.43	0.99	0.68	0.93	1.24	2.22
Egypt	2003	1.21	1.04	0.87	0.49	1.43	2.52	5.13
Egypt	2005	1.35	1.08	0.74	0.52	1.94	1.23	2.23
Egypt	2008	1.53	1.26	0.82	0.56	1.03	1.16	1.90
Egypt	2014	1.58	1.27	0.83	0.52	0.75	1.09	1.85

Jordan	1990	1.34	1.13	0.81	0.59	0.48	1.51	2.53
Jordan	1997	0.94	0.84	0.62	0.43	0.86	1.93	2.79
Jordan	2002	2.07	1.32	0.80	0.61	0.79	0.63	1.58
Jordan	2007	1.28	1.06	0.70	0.45	2.22	1.19	1.91
Jordan	2009	1.30	1.24	0.96	0.60	1.81	2.85	3.45
Jordan	2012	0.90	0.64	0.47	0.39	1.92	1.08	2.65
Jordan	2017/18	1.23	0.91	0.58	0.45	1.09	0.88	1.71
Morocco	1992	2.20	1.93	1.20	0.70	0.67	1.19	1.63
Morocco	2003/4	1.42	1.33	0.90	0.64	1.72	1.70	2.05
Türkiye	1993	1.39	1.17	0.86	0.64	0.99	1.64	2.80
Türkiye	1998	1.44	1.06	0.73	0.63	0.95	1.02	2.16
Türkiye	2003/4	1.47	1.29	0.99	0.69	1.74	2.07	3.30
Yemen	2013	1.34	1.12	0.80	0.63	0.74	1.48	2.46
Tajikistan	2012	1.08	0.85	0.54	0.42	0.20	1.03	1.78
Tajikistan	2017	1.47	1.35	0.79	0.64	0.30	1.15	1.39
Bangladesh	1993/94	1.50	1.25	0.89	0.57	1.27	1.46	2.50
Bangladesh	1996/97	2.02	1.89	1.16	0.72	1.16	1.37	1.59
Bangladesh	1999/0	1.56	1.43	0.97	0.70	1.61	1.68	2.14
Bangladesh	2004	1.92	1.75	1.28	0.93	2.05	2.00	2.73
Bangladesh	2007	1.60	1.44	1.11	0.83	3.16	2.30	3.41
Bangladesh	2011	1.73	1.52	1.07	0.86	2.85	1.62	2.37
Bangladesh	2014	1.64	1.48	0.97	0.79	2.70	1.44	1.88
Bangladesh	2017/18	1.85	1.60	1.13	0.91	4.75	1.58	2.44
Cambodia	2010/11	0.62	0.54	0.43	0.34	1.87	2.19	3.82
Cambodia	2014	0.57	0.50	0.43	0.35	1.11	3.09	6.99
India	2005/6	1.09	0.97	0.69	0.53	2.00	1.69	2.44
India	2015/16	0.80	0.72	0.56	0.46	1.85	2.33	3.56
India	2019/21	0.90	0.81	0.61	0.50	1.96	2.14	3.13
Indonesia	1991	0.69	0.63	0.47	0.28	1.17	2.10	2.79
Indonesia	1994	0.82	0.73	0.55	0.38	1.13	2.00	3.01
Indonesia	1997	0.90	0.80	0.66	0.47	1.30	2.81	4.85
Indonesia	2002/3	0.99	0.91	0.67	0.52	1.44	2.12	2.86
Indonesia	2007	1.01	0.89	0.74	0.58	1.53	2.75	4.86
Indonesia	2012	1.06	0.91	0.74	0.62	1.34	2.28	4.17
Indonesia	2017	1.06	0.94	0.71	0.59	1.15	2.06	3.11
Maldives	2009	1.22	1.18	1.02	0.94	1.79	5.17	6.70
Myanmar	2015/16	0.94	0.76	0.64	0.52	1.97	2.21	5.49
Nepal	2006	1.72	1.38	1.00	0.71	2.30	1.39	2.63
Nepal	2011	0.59	0.52	0.37	0.31	2.18	1.64	2.42
Nepal	2016	1.53	1.43	1.15	0.88	12.21	3.06	4.13
Pakistan	2012/13	1.22	1.07	0.77	0.61	1.63	1.72	2.64
Pakistan	2017/18	1.28	1.08	0.70	0.57	1.39	1.21	1.81
Philippines	1993	1.12	0.90	0.71	0.56	0.88	1.76	3.85

Philippines	1998	1.82	1.48	0.94	0.78	1.72	1.06	1.73
Philippines	2003	1.38	1.22	0.86	0.65	1.24	1.66	2.40
Timor-Leste	2009/10	0.22	0.19	0.14	0.11	1.68	1.89	2.90
Bolivia	1993/94	0.87	0.75	0.62	0.39	1.91	2.47	4.86
Bolivia	2003/4	1.13	0.97	0.76	0.52	1.35	2.03	3.53
Bolivia	2008	1.13	1.03	0.71	0.47	1.88	1.68	2.18
Brazil	1991/92	1.36	1.25	0.88	0.55	0.49	1.82	2.32
Brazil	1996	0.99	0.93	0.70	0.58	1.17	2.43	3.13
Colombia	1995	1.20	0.98	0.71	0.57	1.84	1.45	2.61
Colombia	2000	1.37	1.30	1.05	0.84	3.18	3.38	4.35
Colombia	2004/5	1.49	1.18	0.91	0.72	1.51	1.54	3.33
Colombia	2009/10	1.91	1.68	1.19	0.75	0.87	1.66	2.45
Colombia	2015/16	2.40	2.31	1.79	1.37	2.11	2.91	3.39
Dominican Republic	1991	1.62	1.43	1.02	0.74	1.06	1.69	2.47
Dominican Republic	1996	1.71	1.47	1.04	0.77	1.44	1.56	2.42
Dominican Republic	2002	0.83	0.66	0.57	0.44	1.11	2.13	6.02
Guatemala	1995	0.77	0.70	0.58	0.45	1.66	3.11	4.90
Guatemala	1998/99	1.22	1.17	1.03	0.73	0.62	5.30	7.14
Guatemala	2014/15	1.79	1.56	1.06	0.69	1.69	1.43	2.08
Honduras	2005/6	2.10	1.87	1.31	0.99	4.99	1.66	2.33
Honduras	2011/12	1.74	1.53	1.19	0.86	3.19	2.19	3.58
Nicaragua	1997/98	1.04	0.91	0.65	0.50	0.56	1.69	2.50
Nicaragua	2001	1.58	1.44	0.88	0.61	2.81	1.25	1.54
Paraguay	1990	1.82	1.59	1.24	0.95	0.75	2.12	3.58
Peru	1991/92	0.90	0.74	0.58	0.39	1.03	1.80	3.52
Peru	1996	0.81	0.74	0.59	0.40	1.74	2.75	3.89
Peru	2000	1.31	1.06	0.88	0.58	1.53	2.02	4.77
Peru	2004/6	1.17	1.07	0.98	0.74	2.43	5.12	10.57
Peru	2007/8	3.35	2.86	2.22	1.51	3.87	1.98	3.52
Peru	2009	2.52	2.17	1.40	0.91	1.28	1.25	1.83
Peru	2010	2.51	2.01	1.11	0.95	1.04	0.79	1.23
Peru	2011	3.28	2.80	1.96	1.39	1.00	1.49	2.34
Peru	2012	2.06	1.74	1.17	0.98	1.67	1.31	2.05

STB/ D0-1	Ratio of Stillbirth to D0-1
STB/ D0-2	Ratio of Stillbirth to D0-2
STB/ ENM	Ratio of Stillbirth to ENM
STB/ NNM	Ratio of Stillbirth to NNM
D0/D1	Ratio of D0 to D1
D0-1/D2-6	Ratio of D0-1 to D2-6
D0-2/D3-6	Ratio of D0-2 to D3-6

<u>Table A5 (*cont.*)</u>

Country Name	Survey fieldwork	D0/ D0-1	D0-1/ ENM	D0-2/ ENM	D0-1/ NNM	D0-2/ NNM	ENM/ NNM	Heaping Index
Angola	2015/16	0.706	0.829	0.904	0.762	0.831	0.919	1.964
Benin	2006	0.506	0.614	0.709	0.495	0.571	0.806	1.406
Benin	2011/12	0.717	0.728	0.820	0.598	0.674	0.822	2.163
Benin	2017/18	0.611	0.694	0.781	0.585	0.659	0.843	2.114
Burkina Faso	2003	0.591	0.573	0.710	0.394	0.489	0.688	1.771
Burkina Faso	2010	0.517	0.553	0.738	0.392	0.523	0.708	2.075
Burundi	2010/11	0.653	0.617	0.796	0.439	0.566	0.711	2.837
Ethiopia	2005	0.417	0.661	0.808	0.464	0.567	0.702	2.342
Ethiopia	2010/11	0.759	0.698	0.759	0.556	0.605	0.797	2.538
Ethiopia	2016	0.642	0.795	0.839	0.614	0.648	0.773	2.567
Gambia	2013	0.786	0.617	0.692	0.550	0.617	0.891	0.692
Gambia	2019/20	0.789	0.612	0.757	0.480	0.594	0.785	1.079
Ghana	2003	0.366	0.638	0.685	0.528	0.567	0.828	1.953
Ghana	2008	0.330	0.689	0.730	0.583	0.618	0.847	2.555
Ghana	2014	0.301	0.811	0.869	0.710	0.760	0.875	2.777
Guinea	2005	0.377	0.499	0.723	0.368	0.533	0.737	1.901
Guinea	2018	0.660	0.707	0.805	0.568	0.648	0.804	1.405
Kenya	1998	0.840	0.704	0.818	0.564	0.656	0.802	3.079
Kenya	2003	0.729	0.668	0.789	0.544	0.644	0.815	2.115
Kenya	2008/9	0.726	0.684	0.816	0.552	0.659	0.807	3.820
Kenya	2014	0.683	0.654	0.849	0.455	0.591	0.696	3.658
Lesotho	2009/10	0.741	0.755	0.858	0.606	0.688	0.802	2.471
Lesotho	2014	0.519	0.675	0.798	0.554	0.655	0.821	3.244
Liberia	2013	0.401	0.619	0.791	0.464	0.593	0.749	2.804
Liberia	2019/20	0.814	0.785	0.906	0.658	0.760	0.838	2.917
Madagascar	2003/4	0.560	0.739	0.818	0.513	0.568	0.695	4.225
Madagascar	2008/9	0.599	0.680	0.780	0.543	0.623	0.798	2.563
Malawi	2000	0.615	0.603	0.733	0.420	0.511	0.697	3.008
Malawi	2004/5	0.616	0.681	0.810	0.493	0.586	0.724	2.739
Malawi	2010	0.605	0.741	0.820	0.567	0.628	0.765	3.641
Malawi	2015/16	0.701	0.737	0.850	0.601	0.693	0.815	3.722
Mali	2001	0.645	0.658	0.761	0.454	0.525	0.690	2.128
Mali	2006	0.408	0.615	0.740	0.482	0.580	0.783	1.854
Mali	2012/13	0.724	0.786	0.848	0.678	0.731	0.862	1.689
Mali	2018	0.698	0.600	0.718	0.500	0.598	0.834	1.602
Mozambique	2003/4	0.524	0.445	0.578	0.287	0.372	0.645	2.927
Mozambique	2011	0.706	0.787	0.902	0.707	0.810	0.898	3.213
Namibia	2006/7	0.417	0.751	0.848	0.638	0.720	0.849	3.339
Namibia	2013	0.430	0.785	0.886	0.640	0.722	0.816	2.811

Niger	2006	0.628	0.524	0.692	0.340	0.450	0.650	1.439
Niger	2012	0.312	0.633	0.787	0.463	0.575	0.731	1.034
Nigeria	2008	0.602	0.663	0.765	0.521	0.601	0.786	1.665
Nigeria	2013	0.471	0.614	0.754	0.481	0.590	0.783	1.065
Nigeria	2018	0.703	0.720	0.822	0.595	0.679	0.826	1.718
Rwanda	2000	0.742	0.684	0.790	0.494	0.571	0.722	2.871
Rwanda	2005	0.838	0.705	0.796	0.514	0.580	0.729	2.870
Rwanda	2010/11	0.703	0.678	0.788	0.458	0.532	0.676	4.009
Rwanda	2014/15	0.648	0.595	0.747	0.433	0.544	0.729	3.346
Rwanda	2019/20	0.886	0.700	0.827	0.567	0.670	0.810	2.723
Senegal	2005	0.614	0.561	0.655	0.417	0.487	0.742	1.352
Senegal	2010/11	0.568	0.700	0.770	0.557	0.612	0.795	1.641
Senegal	2015	0.409	0.441	0.538	0.314	0.383	0.712	1.892
Senegal	2016	0.350	0.374	0.535	0.297	0.424	0.794	0.735
Senegal	2018	0.457	0.551	0.685	0.471	0.584	0.853	0.488
Senegal	2019	0.376	0.299	0.531	0.230	0.409	0.770	0.946
Sierra Leone	2008	0.615	0.643	0.770	0.457	0.548	0.712	2.662
Sierra Leone	2013	0.408	0.591	0.719	0.479	0.583	0.811	2.070
South Africa	2016	0.767	0.833	0.868	0.712	0.742	0.855	3.087
Eswatini	2006/7	0.462	0.632	0.765	0.507	0.613	0.802	3.588
Tanzania	2004/5	0.687	0.719	0.857	0.549	0.654	0.764	2.894
Tanzania	2009/10	0.572	0.673	0.786	0.506	0.590	0.751	3.351
Tanzania	2015/16	0.669	0.639	0.754	0.550	0.649	0.861	1.797
Uganda	2000/1	0.687	0.746	0.848	0.544	0.618	0.729	3.389
Uganda	2006	0.740	0.682	0.744	0.522	0.570	0.765	3.190
Uganda	2011	0.617	0.715	0.856	0.545	0.652	0.762	3.751
Zambia	2007	0.674	0.634	0.738	0.448	0.521	0.706	3.201
Zambia	2013/14	0.639	0.659	0.818	0.509	0.631	0.772	2.664
Zambia	2018/19	0.784	0.751	0.854	0.579	0.659	0.772	4.221
Zimbabwe	1994	0.662	0.579	0.704	0.414	0.504	0.715	2.554
Zimbabwe	1999	0.568	0.796	0.911	0.603	0.690	0.757	3.476
Zimbabwe	2005/6	0.782	0.742	0.803	0.544	0.589	0.733	3.324
Zimbabwe	2010/11	0.805	0.740	0.838	0.598	0.678	0.809	2.652
Zimbabwe	2015	0.652	0.660	0.834	0.483	0.611	0.732	3.585
Azerbaijan	2006	0.472	0.701	0.823	0.555	0.652	0.793	2.152
Egypt	1992/93	0.481	0.540	0.665	0.331	0.408	0.613	3.049
Egypt	1995/96	0.509	0.515	0.653	0.323	0.410	0.628	3.025
Egypt	2000	0.481	0.554	0.689	0.379	0.473	0.686	4.178
Egypt	2003	0.589	0.716	0.837	0.406	0.474	0.566	3.974
Egypt	2005	0.660	0.552	0.690	0.385	0.482	0.698	2.743
Egypt	2008	0.507	0.537	0.655	0.368	0.448	0.684	2.198
Egypt	2014	0.428	0.522	0.649	0.329	0.409	0.630	2.653
Jordan	1990	0.326	0.602	0.717	0.436	0.520	0.725	2.096

Jordan	1997	0.463	0.658	0.736	0.458	0.512	0.696	2.467
Jordan	2002	0.441	0.388	0.612	0.294	0.463	0.757	2.232
Jordan	2007	0.689	0.544	0.657	0.351	0.425	0.646	0.718
Jordan	2009	0.644	0.740	0.776	0.465	0.488	0.629	1.569
Jordan	2012	0.657	0.520	0.726	0.433	0.605	0.833	1.192
Jordan	2017/18	0.522	0.467	0.631	0.363	0.491	0.777	2.041
Morocco	1992	0.400	0.543	0.620	0.318	0.363	0.586	1.889
Morocco	2003/4	0.632	0.630	0.672	0.453	0.483	0.719	2.406
Türkiye	1993	0.498	0.621	0.737	0.462	0.547	0.743	2.054
Türkiye	1998	0.488	0.504	0.684	0.438	0.594	0.869	1.230
Türkiye	2003/4	0.635	0.675	0.767	0.472	0.536	0.699	4.277
Yemen	2013	0.425	0.597	0.711	0.471	0.561	0.789	2.168
Tajikistan	2012	0.163	0.507	0.640	0.393	0.497	0.775	0.671
Tajikistan	2017	0.233	0.534	0.581	0.431	0.469	0.807	0.723
Bangladesh	1993/94	0.559	0.593	0.714	0.382	0.460	0.644	1.749
Bangladesh	1996/97	0.537	0.577	0.615	0.358	0.381	0.620	1.898
Bangladesh	1999/0	0.617	0.627	0.682	0.448	0.487	0.715	1.151
Bangladesh	2004	0.672	0.667	0.732	0.483	0.530	0.724	1.592
Bangladesh	2007	0.760	0.697	0.773	0.521	0.578	0.748	1.217
Bangladesh	2011	0.740	0.619	0.703	0.500	0.568	0.808	1.357
Bangladesh	2014	0.729	0.590	0.652	0.484	0.535	0.820	0.349
Bangladesh	2017/18	0.826	0.612	0.709	0.491	0.569	0.802	1.346
Cambodia	2010/11	0.652	0.687	0.793	0.556	0.642	0.810	1.909
Cambodia	2014	0.526	0.755	0.875	0.613	0.710	0.811	3.158
India	2005/6	0.667	0.629	0.709	0.486	0.548	0.773	0.760
India	2015/16	0.650	0.700	0.781	0.581	0.649	0.831	0.872
India	2019/21	0.662	0.682	0.758	0.563	0.626	0.826	1.053
Indonesia	1991	0.540	0.678	0.736	0.408	0.443	0.602	2.945
Indonesia	1994	0.531	0.666	0.751	0.467	0.526	0.701	1.662
Indonesia	1997	0.565	0.738	0.829	0.527	0.592	0.714	2.900
Indonesia	2002/3	0.589	0.679	0.741	0.525	0.573	0.773	1.430
Indonesia	2007	0.604	0.733	0.829	0.572	0.647	0.780	3.040
Indonesia	2012	0.572	0.695	0.807	0.581	0.675	0.837	2.854
Indonesia	2017	0.534	0.674	0.757	0.558	0.627	0.828	2.132
Maldives	2009	0.642	0.838	0.870	0.769	0.799	0.918	0.000
Myanmar	2015/16	0.664	0.689	0.846	0.552	0.678	0.802	1.030
Nepal	2006	0.697	0.581	0.725	0.414	0.516	0.713	0.308
Nepal	2011	0.686	0.621	0.707	0.529	0.602	0.851	1.501
Nepal	2016	0.924	0.753	0.805	0.577	0.617	0.766	1.362
Pakistan	2012/13	0.620	0.632	0.725	0.502	0.576	0.794	0.654
Pakistan	2017/18	0.581	0.547	0.644	0.448	0.528	0.819	1.363
Philippines	1993	0.467	0.638	0.794	0.502	0.624	0.786	3.015
Philippines	1998	0.633	0.514	0.633	0.427	0.525	0.830	1.804

Philippines	2003	0.553	0.624	0.706	0.471	0.533	0.755	2.194
Timor-Leste	2009/10	0.627	0.654	0.743	0.507	0.576	0.775	2.053
Bolivia	1993/94	0.656	0.712	0.829	0.443	0.517	0.623	3.309
Bolivia	2003/4	0.575	0.670	0.779	0.458	0.533	0.684	3.654
Bolivia	2008	0.653	0.627	0.685	0.419	0.458	0.669	2.364
Brazil	1991/92	0.331	0.646	0.699	0.408	0.442	0.632	1.298
Brazil	1996	0.538	0.709	0.758	0.587	0.627	0.828	1.608
Colombia	1995	0.647	0.592	0.723	0.472	0.576	0.797	0.326
Colombia	2000	0.761	0.772	0.813	0.612	0.645	0.793	1.608
Colombia	2004/5	0.601	0.606	0.769	0.485	0.615	0.799	0.502
Colombia	2009/10	0.466	0.623	0.710	0.390	0.444	0.626	1.967
Colombia	2015/16	0.679	0.744	0.772	0.569	0.590	0.764	0.555
Dominican Republic	1991	0.514	0.628	0.712	0.457	0.518	0.727	1.777
Dominican Republic	1996	0.589	0.609	0.707	0.452	0.525	0.742	0.542
Dominican Republic	2002	0.527	0.680	0.857	0.531	0.669	0.781	1.876
Guatemala	1995	0.624	0.756	0.831	0.590	0.647	0.779	0.819
Guatemala	1998/99	0.382	0.841	0.877	0.600	0.625	0.713	0.937
Guatemala	2014/15	0.628	0.589	0.676	0.387	0.444	0.657	1.055
Honduras	2005/6	0.833	0.624	0.700	0.472	0.529	0.757	0.730
Honduras	2011/12	0.761	0.686	0.782	0.492	0.561	0.718	1.157
Nicaragua	1997/98	0.359	0.628	0.714	0.484	0.550	0.770	0.285
Nicaragua	2001	0.737	0.556	0.606	0.387	0.423	0.697	1.659
Paraguay	1990	0.429	0.679	0.782	0.521	0.599	0.767	1.094
Peru	1991/92	0.508	0.643	0.779	0.437	0.530	0.680	1.593
Peru	1996	0.636	0.733	0.796	0.500	0.542	0.682	1.836
Peru	2000	0.605	0.668	0.827	0.445	0.550	0.665	2.363
Peru	2004/6	0.709	0.837	0.914	0.635	0.693	0.759	2.385
Peru	2007/8	0.794	0.664	0.779	0.450	0.528	0.678	0.767
Peru	2009	0.562	0.556	0.647	0.360	0.418	0.647	1.612
Peru	2010	0.509	0.442	0.551	0.378	0.472	0.856	0.723
Peru	2011	0.499	0.598	0.701	0.423	0.496	0.708	2.917
Peru	2012	0.625	0.566	0.672	0.476	0.566	0.841	1.823

D0/D0-1	Prop. of D0 to D0-1
D0-1/ENM	Prop. of D0-1 to ENM
D0-2/ENM	Prop. of D0-2 to ENM
D0-1/NNM	Prop. of D0-1 to NNM
D0-2/NNM	Prop. of D0-2 to NNM
ENM/NNM	Prop. of ENM to NNM
heaping_index	Heaping Index at day 7

Appendix 6: data quality correlations and associations

Table A6.1: Pair-wise correlations (*r*) between the data quality indicators and early neonatal mortality

		Heaping index	Stillbirth to deaths on D0-1	Deaths on D0-1 to deaths on D2-6	Early Neonatal mortality
Heaping index		1			
Stillbirths to deaths on D0-1	*r*(157)	-0.2686	1		
	p-value	*(0.0007)*			
Deaths on D0-1 to deaths D2-6	*r*(157)	0.2219	-0.4491	1	
	p-value	*(0.0052)*	*(0.0000)*		
Early neonatal mortality	*r*(157)	0.0636	-0.4245	0.0414	1
	p-value	*(0.4289)*	*(0.0000)*	*(0.6069)*	

Table A6.2: Level of significance (P-values) of the association between the data quality indicators and the surveys and respondents' characteristics

Characteristics	Heaping index	Stillbirths to deaths on D0-1	Deaths on D0-1 to deaths on D2-6
Region	*(0.0000)*	*(0.0012)*	*(0.0101)*
Wealth	*(0.8690)*	*(0.3981)*	*(0.0777)*
Place of residence	*(0.2827)*	*(0.3661)*	*(0.0288)*
Level of education	*(0.8338)*	*(0.3385)*	*(0.0440)*
Maternal age	*(0.4549)*	*(0.0000)*	*(0.0137)*
Number of livebirths	*(0.6386)*	*(0.5985)*	*(0.3390)*
Birth Calendar	*(0.1296)*	*(0.3935)*	*(0.8012)*

Appendix 7: Scatter plots of data quality indicators

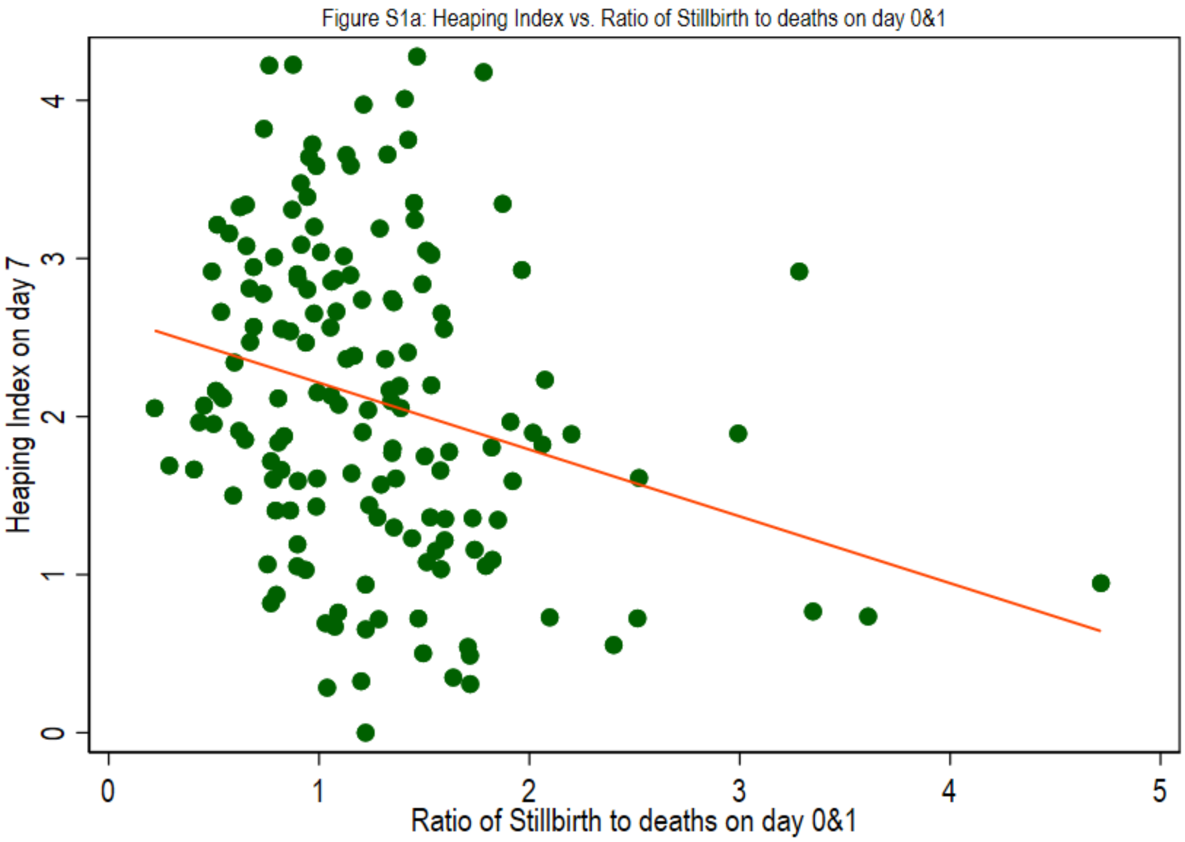

Figure S1b: Heaping Index vs. Ratio of deaths on day 1&2 to day 2&6

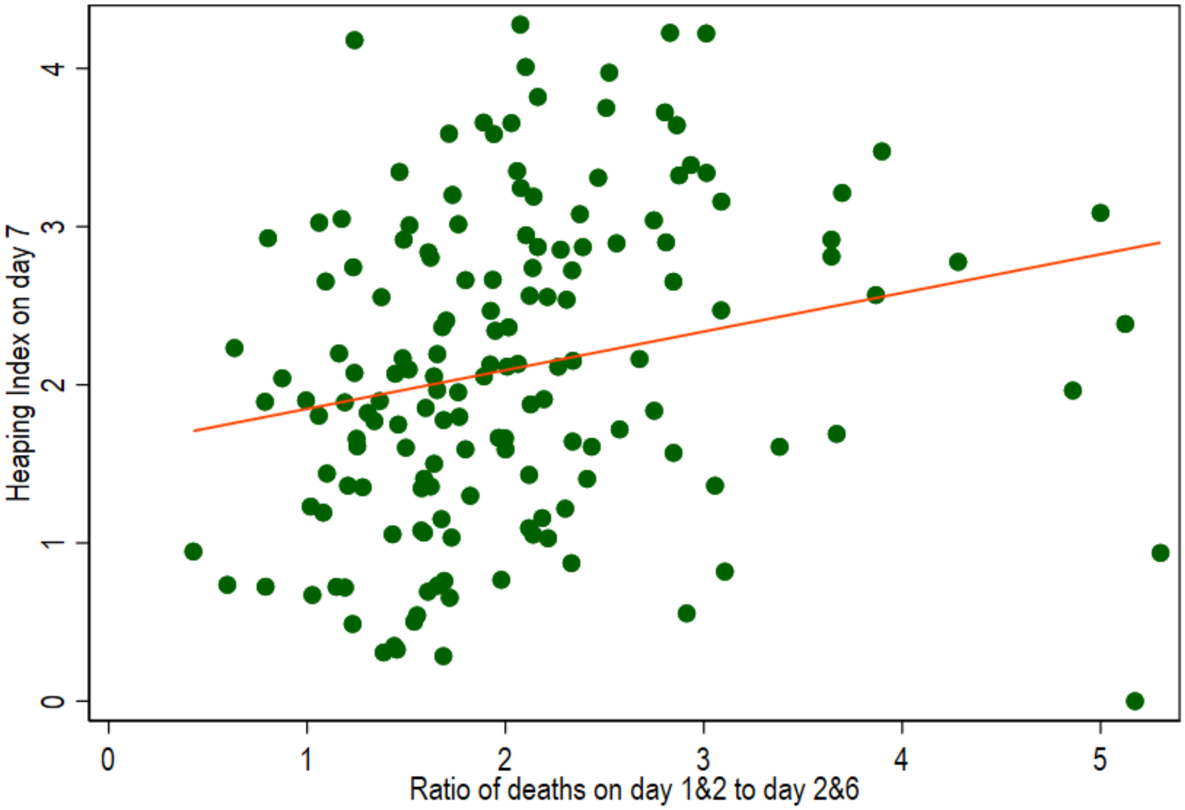

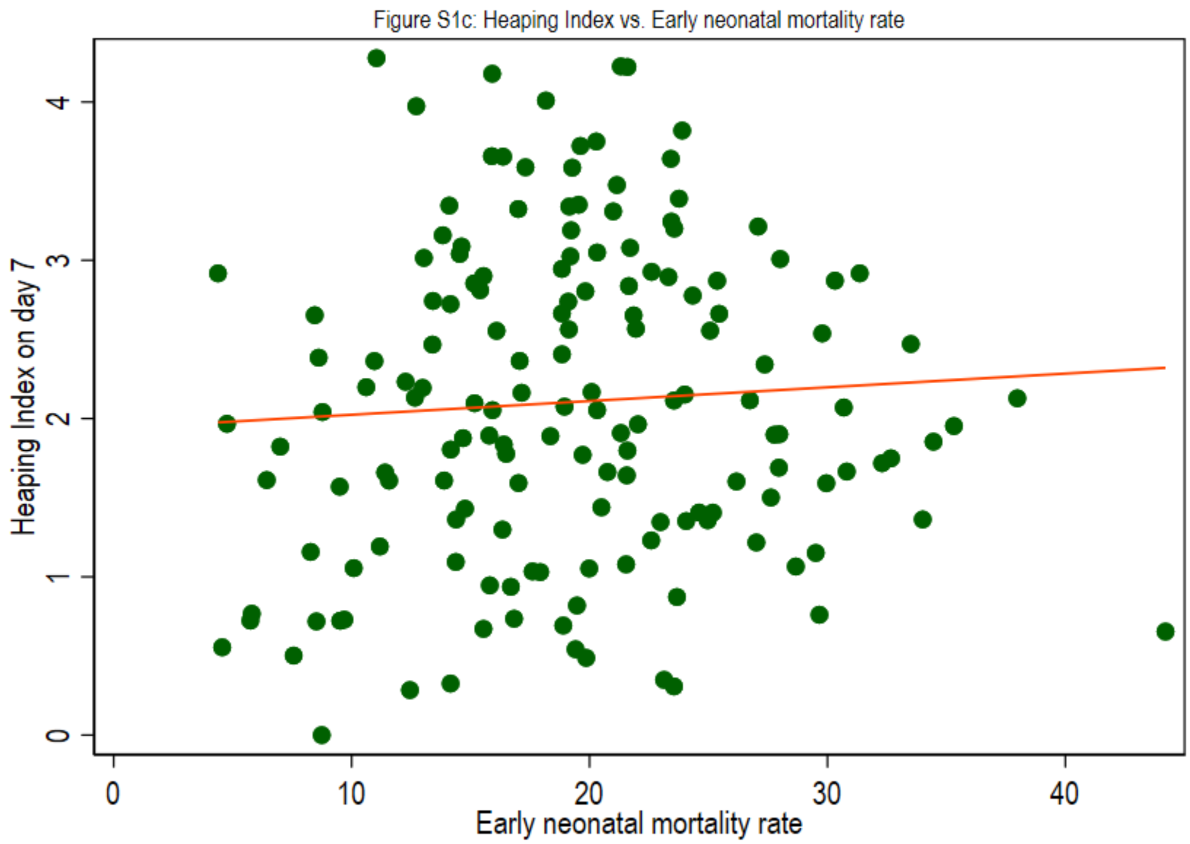
Figure S1c: Heaping Index vs. Early neonatal mortality rate
Heaping Index on day 7
0
1
2
3
4
0
10
20
30
40
Early neonatal mortality rate

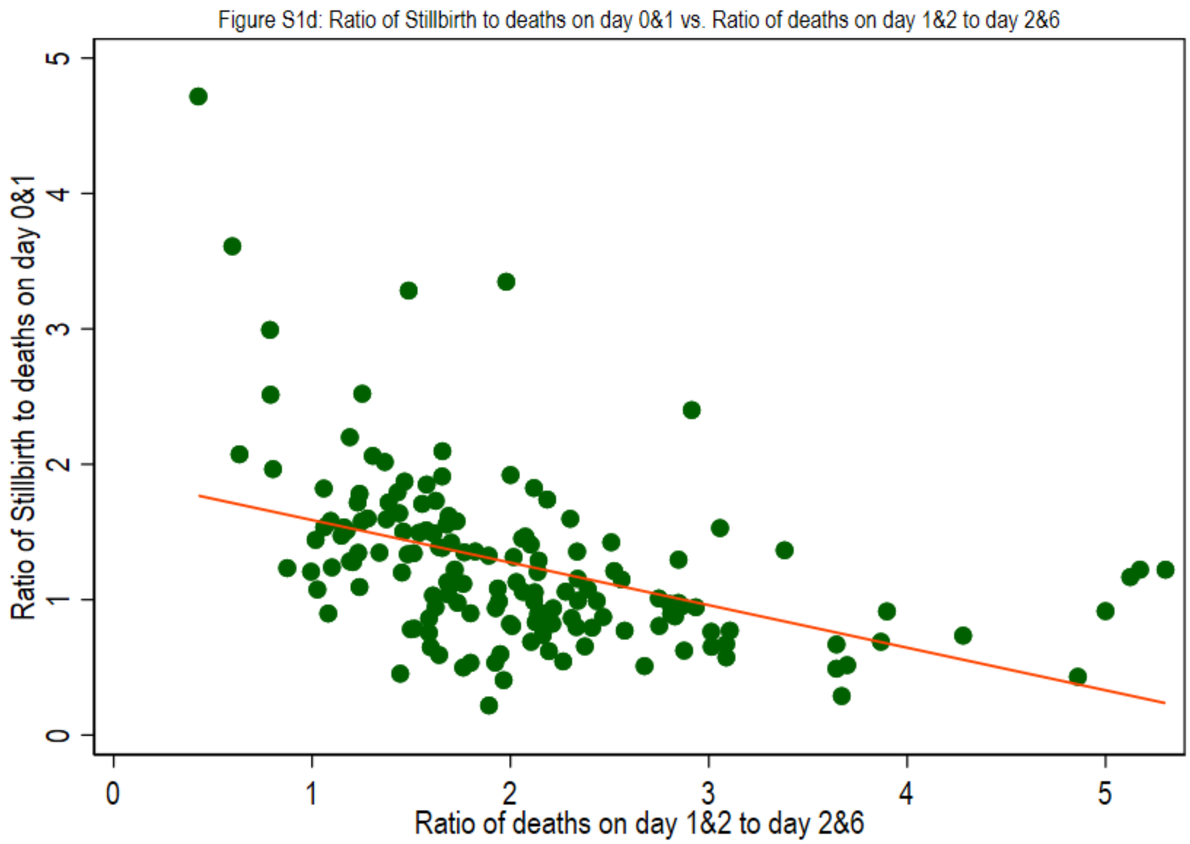
Figure S1d: Ratio of Stillbirth to deaths on day 0&1 vs. Ratio of deaths on day 1&2 to day 2&6
Ratio of Stillbirth to deaths on day 0&1
0
1
2
3
4
5
0
1
2
3
4
5
Ratio of deaths on day 1&2 to day 2&6

Figure S1f: Ratio of deaths on day 1&2 to day 2&6 vs. Early neonatal mortality rate

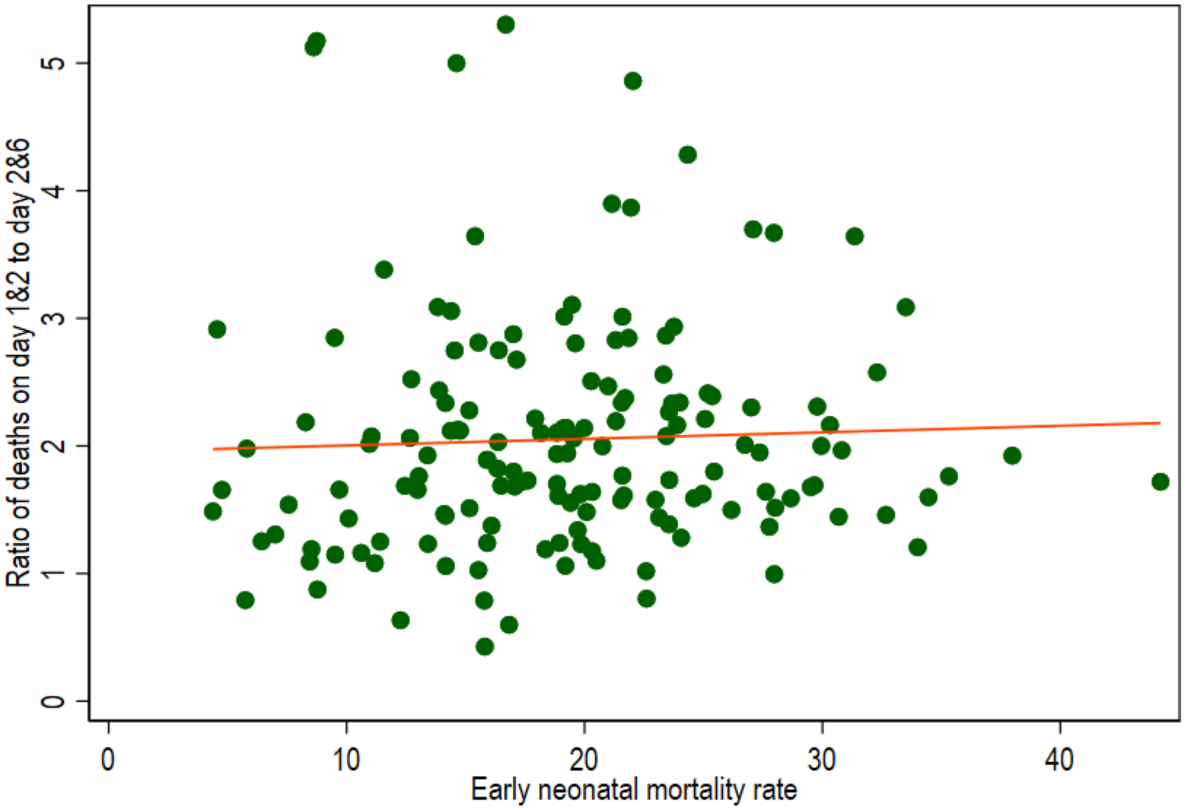

Figure S1f: Ratio of deaths on day 1&2 to day 2&6 vs. Early neonatal mortality rate

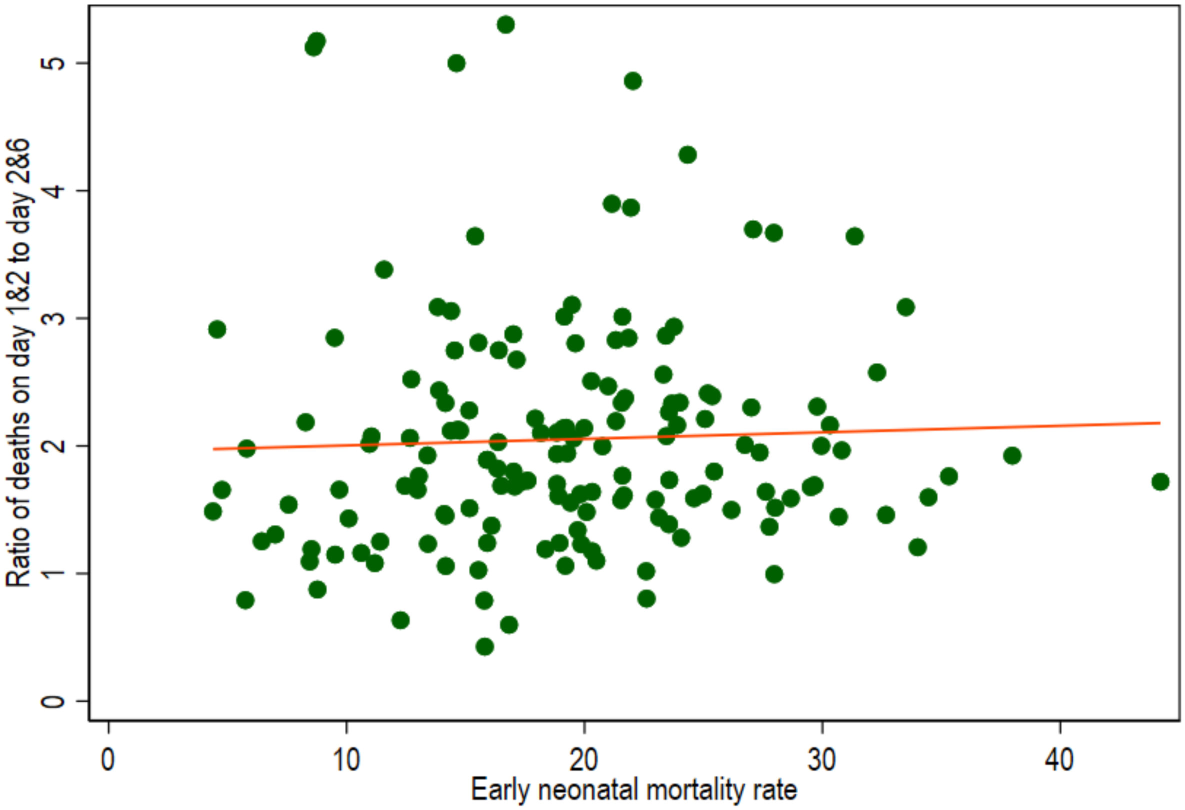

Appendix 8: Country-specific results

Table A8: country specific observed and adjusted stillbirth, early neonatal and perinatal mortality rates

Country	Survey Year	Stillbirth rates per 1000 births				Early neonatal mortality per 1000 livebirths				Perinatal mortality per 1000 births			
		M1	M2	M3	M4	M1	M2	M3	M4	M1	M2	M3	M4
Angola	2015/16	7.5	16.6	16.6	7.5	22.0	12.8	12.8	22.2	29.4	29.1	29.1	29.6
Benin	2006	12.4	34.9	26.1	12.4	24.6	27.8	23.1	31.8	36.7	61.8	48.6	43.8
Benin	2011/12	6.1	12.0	12.0	6.1	17.1	10.9	10.9	17.0	23.2	22.8	22.8	23.0
Benin	2017/18	8.5	26.2	25.0	8.5	23.5	21.5	20.9	24.8	31.9	47.1	45.4	33.1
Burkina Faso	2003	14.4	33.2	22.9	14.4	19.7	25.7	20.2	28.0	33.8	58.0	42.6	42.0
Burkina Faso	2010	10.9	31.1	19.7	10.9	18.9	24.4	18.4	27.9	29.7	54.8	37.7	38.6
Burundi	2010/11	18.7	35.6	28.2	18.7	21.7	27.4	23.4	28.2	40.0	62.1	50.9	46.4
Ethiopia	2005	10.2	32.9	28.1	10.2	27.4	27.5	24.9	32.1	37.3	59.5	52.3	42.0
Ethiopia	2010/11	17.0	36.6	35.6	17.0	29.8	28.4	27.9	30.5	46.3	64.0	62.6	46.9
Ethiopia	2016	11.4	18.6	18.6	11.4	21.9	14.5	14.5	22.1	33.1	32.8	32.8	33.2
Gambia	2013	11.4	28.0	21.4	11.4	18.9	21.7	18.2	24.1	30.1	49.1	39.1	35.2
Gambia	2019/20	18.7	35.7	27.9	18.7	21.5	27.5	23.4	28.4	39.8	62.3	50.6	46.5
Ghana	2003	10.7	42.9	33.6	10.7	35.3	36.0	31.1	43.4	45.6	77.3	63.6	53.6
Ghana	2008	13.5	31.0	29.3	13.5	25.1	24.0	23.1	26.2	38.2	54.3	51.8	39.3
Ghana	2014	13.8	21.6	21.6	13.8	24.3	16.3	16.3	24.6	37.8	37.6	37.6	38.0
Guinea	2005	15.7	49.0	26.8	15.7	28.0	41.4	29.6	48.1	43.3	88.4	55.6	63.0
Guinea	2018	13.5	20.2	20.2	13.5	25.2	17.9	17.9	25.0	38.3	37.7	37.7	38.1
Kenya	1998	9.6	24.6	24.5	9.6	21.7	19.1	19.0	21.4	31.1	43.3	43.0	30.8
Kenya	2003	13.6	34.0	30.0	13.6	26.7	26.1	24.0	29.2	40.0	59.3	53.3	42.4
Kenya	2008/9	11.5	29.1	27.0	11.5	23.9	23.6	22.5	26.1	35.1	52.0	48.9	37.4
Kenya	2014	13.1	24.3	21.0	13.1	15.9	19.2	17.4	19.4	28.8	43.0	38.1	32.3
Lesotho	2009/10	16.2	26.7	26.7	16.2	33.5	23.6	23.6	34.6	49.2	49.7	49.7	50.3
Lesotho	2014	21.7	35.8	33.0	21.7	23.4	26.5	25.0	25.7	44.6	61.3	57.1	46.8
Liberia	2013	10.9	28.5	21.7	10.9	19.8	23.0	19.4	25.8	30.5	50.8	40.7	36.5
Liberia	2019/20	11.5	23.1	23.1	11.5	31.4	19.6	19.6	31.8	42.4	42.2	42.2	42.9
Madagascar	2003/4	13.1	18.6	18.6	13.1	21.3	17.8	17.8	23.6	34.1	36.0	36.0	36.3
Madagascar	2008/9	13.0	26.0	24.0	13.0	19.1	20.8	19.7	21.6	31.9	46.2	43.3	34.3
Malawi	2000	12.5	39.4	28.1	12.5	28.0	32.7	26.7	38.1	40.2	70.8	54.1	50.1
Malawi	2004/5	14.8	27.0	25.1	14.8	19.1	21.4	20.4	21.6	33.6	47.8	45.0	36.1
Malawi	2010	15.7	21.4	21.4	15.7	23.4	18.7	18.7	24.6	38.8	39.7	39.7	40.0
Malawi	2015/16	13.3	17.9	17.9	13.3	19.6	15.4	15.4	20.3	32.6	33.1	33.1	33.3
Mali	2001	12.6	44.5	37.5	12.6	38.0	37.3	33.6	44.4	50.1	80.1	69.8	56.4
Mali	2006	13.0	45.6	33.5	13.0	34.5	37.0	30.6	44.1	47.0	80.9	63.1	56.5
Mali	2012/13	6.1	18.0	18.0	6.1	28.0	15.1	15.1	27.5	33.9	32.8	32.8	33.4
Mali	2018	11.7	37.2	26.3	11.7	26.2	29.6	23.8	34.6	37.5	65.7	49.5	45.9
Mozambique	2003/4	18.7	31.5	19.0	18.7	22.6	30.1	23.7	43.2	40.9	60.6	42.3	61.1
Mozambique	2011	10.4	20.3	20.3	10.4	27.1	16.6	16.6	27.0	37.2	36.6	36.6	37.2
Namibia	2006/7	8.9	15.0	15.0	8.9	19.2	13.1	13.1	19.5	27.9	27.9	27.9	28.2
Namibia	2013	7.7	12.7	12.7	7.7	15.4	10.0	10.0	15.3	23.0	22.6	22.6	22.9
Niger	2006	12.5	35.5	21.0	12.5	20.5	28.8	21.1	33.0	32.8	63.3	41.6	45.1
Niger	2012	16.6	29.1	24.1	16.6	17.6	22.2	19.6	22.1	33.9	50.7	43.2	38.3
Nigeria	2008	7.9	34.5	29.3	7.9	30.8	28.8	26.1	35.2	38.5	62.3	54.6	42.8
Nigeria	2013	12.4	39.0	28.8	12.4	28.7	31.7	26.2	37.1	40.7	69.4	54.3	49.1
Nigeria	2018	16.9	25.8	25.8	16.9	32.3	22.5	22.5	32.0	48.6	47.8	47.8	48.3
Rwanda	2000	17.6	38.5	35.9	17.6	30.3	31.0	29.6	33.4	47.4	68.3	64.5	50.3

Rwanda	2005	18.2	33.1	33.0	18.2	25.4	26.3	26.3	26.6	43.1	58.6	58.5	44.3
Rwanda	2010/11	16.5	27.5	25.5	16.5	18.2	21.4	20.3	20.8	34.3	48.3	45.3	36.9
Rwanda	2014/15	14.9	26.0	19.8	14.9	14.1	20.2	16.9	20.0	28.8	45.7	36.4	34.6
Rwanda	2019/20	12.8	20.6	20.3	12.8	14.2	14.7	14.6	14.0	26.8	35.0	34.5	26.6
Senegal	2005	20.4	42.9	29.4	20.4	24.1	33.1	25.9	35.1	43.9	74.5	54.5	54.8
Senegal	2010/11	16.4	29.1	28.7	16.4	21.6	22.1	21.8	22.1	37.6	50.6	49.9	38.1
Senegal	2015	19.7	29.9	19.9	19.7	15.8	18.9	15.5	29.6	35.2	48.3	35.2	48.7
Senegal	2016	21.3	35.3	21.5	21.3	16.8	19.5	15.7	34.3	37.7	54.1	36.9	54.8
Senegal	2018	17.6	36.5	24.6	17.6	19.9	27.2	20.8	28.8	37.2	62.7	44.9	46.0
Senegal	2019	20.9	37.7	21.1	20.9	15.8	18.5	14.9	36.2	36.4	55.6	35.7	56.4
Sierra Leone	2008	8.3	31.2	24.9	8.3	25.4	27.2	23.9	32.2	33.5	57.5	48.1	40.3
Sierra Leone	2013	7.8	40.3	26.6	7.8	30.7	33.7	26.4	41.9	38.3	72.7	52.3	49.3
South Africa	2016	10.5	14.7	14.7	10.6	14.6	10.4	10.4	14.8	25.0	24.9	24.9	25.2
Eswatini	2006/7	11.9	25.9	20.9	11.9	17.3	21.4	18.7	22.8	29.0	46.7	39.1	34.4
Tanzania	2004/5	18.1	22.6	22.6	18.1	23.3	18.8	18.8	23.6	41.0	41.0	41.0	41.3
Tanzania	2009/10	18.0	29.9	27.4	18.0	19.5	22.3	21.0	21.7	37.2	51.6	47.9	39.3
Tanzania	2015/16	17.6	33.6	27.9	17.6	21.6	25.1	22.1	25.8	38.8	57.9	49.4	43.0
Uganda	2000/1	15.8	21.7	21.7	15.8	23.8	18.8	18.8	25.0	39.2	40.1	40.1	40.4
Uganda	2006	15.9	27.8	26.0	15.9	19.2	20.7	19.8	20.6	34.8	48.0	45.2	36.2
Uganda	2011	19.4	22.0	22.0	19.4	20.3	18.8	18.8	21.5	39.3	40.3	40.3	40.5
Zambia	2007	13.8	33.4	26.8	13.8	23.6	26.3	22.8	29.1	37.1	58.9	49.0	42.5
Zambia	2013/14	12.8	26.7	23.2	12.8	18.8	20.8	18.9	21.9	31.4	46.9	41.7	34.4
Zambia	2018/19	11.7	18.0	18.0	11.7	21.6	16.6	16.6	23.2	33.1	34.3	34.3	34.7
Zimbabwe	1994	14.1	28.4	20.4	14.1	16.1	21.5	17.2	22.5	29.9	49.3	37.3	36.3
Zimbabwe	1999	14.6	20.3	20.3	14.6	21.2	16.6	16.6	22.6	35.4	36.5	36.5	36.8
Zimbabwe	2005/6	7.5	13.0	13.0	7.5	17.0	11.6	11.6	17.3	24.4	24.4	24.4	24.7
Zimbabwe	2010/11	14.8	20.0	20.0	14.8	21.9	16.4	16.4	21.9	36.3	36.0	36.0	36.4
Zimbabwe	2015	12.0	26.6	23.1	12.0	19.3	21.8	19.9	23.3	31.0	47.7	42.5	35.0
Azerbaijan	2006	15.8	30.7	30.2	15.8	24.0	22.9	22.7	23.8	39.4	52.9	52.2	39.2
Egypt	1992/93	15.6	36.4	23.3	15.6	20.3	31.1	24.1	33.8	35.6	66.4	46.9	48.9
Egypt	1995/96	14.3	35.6	21.2	14.3	19.2	29.8	22.2	32.8	33.2	64.3	42.9	46.7
Egypt	2000	15.0	30.0	20.4	15.0	15.9	24.4	19.3	25.3	30.6	53.6	39.3	39.9
Egypt	2003	10.5	12.8	12.8	10.5	12.7	12.1	12.1	14.4	23.1	24.7	24.7	24.8
Egypt	2005	9.6	23.4	15.2	9.6	13.4	18.8	14.4	20.6	22.8	41.8	29.4	30.0
Egypt	2008	8.4	19.5	12.4	8.4	10.6	15.2	11.4	16.5	18.9	34.4	23.6	24.8
Egypt	2014	6.7	15.9	9.7	6.7	8.4	12.6	9.3	13.8	15.1	28.4	19.0	20.4
Jordan	1990	11.6	24.7	18.5	11.6	15.2	19.5	16.2	20.8	26.6	43.7	34.4	32.1
Jordan	1997	7.9	18.3	15.7	7.9	13.4	14.9	13.5	16.1	21.2	32.9	29.0	23.9
Jordan	2002	9.4	18.0	9.5	9.4	12.3	16.3	12.2	25.3	21.5	34.1	21.7	34.5
Jordan	2007	5.6	14.8	9.3	5.6	8.5	12.2	9.2	13.6	14.1	26.8	18.4	19.2
Jordan	2009	8.7	10.2	10.2	8.7	9.5	8.8	8.8	10.4	18.1	18.9	18.9	19.0
Jordan	2012	5.0	18.6	10.2	5.0	11.2	15.0	10.6	17.9	16.1	33.3	20.7	22.8
Jordan	2017/18	4.8	16.4	8.0	4.8	8.8	13.6	9.2	16.1	13.5	29.8	17.1	20.7
Morocco	1992	20.6	27.5	21.0	20.6	18.4	21.9	19.0	29.2	38.6	48.8	39.5	49.2
Morocco	2003/4	15.9	30.2	24.6	15.9	18.8	21.8	18.8	22.4	34.5	51.4	43.0	38.0
Türkiye	1993	16.5	32.6	25.8	16.5	20.3	24.3	20.7	25.3	36.5	56.1	46.0	41.4
Türkiye	1998	15.6	41.5	23.8	15.6	22.6	31.8	22.4	36.1	37.8	72.0	45.6	51.0
Türkiye	2003/4	10.4	17.2	15.8	10.4	11.0	12.6	11.9	12.1	21.4	29.6	27.5	22.4
Yemen	2013	15.1	32.7	24.1	15.1	20.1	24.8	20.2	26.6	34.9	56.7	43.9	41.3
Tajikistan	2012	7.9	26.7	14.5	7.9	15.5	20.7	14.2	24.7	23.3	46.8	28.6	32.4
Tajikistan	2017	7.1	17.2	10.7	7.1	9.5	12.8	9.3	14.2	16.5	29.8	20.0	21.2
Bangladesh	1993/94	26.9	54.2	40.3	26.9	32.7	43.3	35.9	45.7	58.7	95.2	74.8	71.4
Bangladesh	1996/97	29.9	38.0	30.6	29.9	27.8	30.5	26.8	39.2	56.8	67.3	56.5	67.9
Bangladesh	1999/0	26.8	48.3	39.3	26.8	29.5	36.5	31.7	36.9	55.5	83.0	69.8	62.7

Bangladesh	2004	35.8	38.9	36.5	35.8	30.0	29.0	27.7	32.4	64.7	66.8	63.2	67.0
Bangladesh	2007	28.1	41.6	40.7	28.1	27.0	30.3	29.8	27.7	54.4	70.6	69.2	55.1
Bangladesh	2011	24.9	43.0	34.6	24.9	25.0	31.4	26.9	31.1	49.3	73.0	60.6	55.3
Bangladesh	2014	20.9	40.3	29.9	20.9	23.1	29.3	23.8	30.3	43.6	68.4	53.0	50.6
Bangladesh	2017/18	24.3	40.9	32.6	24.3	23.0	29.5	25.0	28.8	46.7	69.2	56.8	52.4
Cambodia	2010/11	8.6	24.6	23.0	8.6	21.3	19.4	18.5	22.2	29.7	43.5	41.1	30.7
Cambodia	2014	5.7	10.4	10.4	5.7	13.8	9.6	9.6	14.5	19.5	19.9	19.9	20.1
India	2005/6	19.1	43.4	34.5	19.1	29.7	33.3	28.6	36.3	48.2	75.2	62.1	54.7
India	2015/16	12.5	28.4	27.9	12.5	23.7	21.7	21.4	23.6	35.9	49.5	48.7	35.8
India	2019/21	11.6	25.7	23.8	11.6	20.0	19.6	18.6	21.1	31.3	44.8	41.9	32.5
Indonesia	1991	8.4	22.8	20.7	8.4	18.8	20.4	19.3	22.8	27.0	42.7	39.6	31.0
Indonesia	1994	10.7	26.7	23.5	10.8	20.8	21.6	19.9	23.9	31.3	47.7	42.9	34.4
Indonesia	1997	9.8	13.7	13.7	9.8	15.5	12.4	12.4	16.5	25.2	25.9	25.9	26.1
Indonesia	2002/3	9.4	19.7	18.2	9.4	14.8	15.5	14.7	16.4	24.1	34.9	32.6	25.6
Indonesia	2007	10.3	13.6	13.6	10.3	14.5	11.2	11.2	14.7	24.7	24.7	24.7	24.8
Indonesia	2012	10.6	20.1	19.5	10.6	15.2	15.2	14.9	15.6	25.5	35.0	34.0	26.0
Indonesia	2017	8.6	17.5	15.8	8.6	12.7	13.1	12.2	13.7	21.1	30.3	27.9	22.2
Maldives	2009	8.6	10.3	10.3	8.6	8.7	6.7	6.7	8.6	17.2	17.0	17.0	17.0
Myanmar	2015/16	11.0	23.2	22.0	11.0	17.9	17.6	16.9	18.6	28.7	40.3	38.5	29.4
Nepal	2006	22.3	42.3	30.9	22.3	23.6	31.2	25.1	32.0	45.3	72.2	55.2	53.6
Nepal	2011	9.8	36.1	26.9	9.8	27.6	28.3	23.4	33.9	37.1	63.3	49.6	43.3
Nepal	2016	15.7	17.3	17.3	15.7	14.4	11.9	11.9	13.6	29.9	29.0	29.0	29.1
Pakistan	2012/13	31.7	65.4	53.2	31.6	44.2	49.3	42.8	52.7	74.5	111.4	93.7	82.7
Pakistan	2017/18	22.4	57.3	36.7	22.4	34.0	44.8	33.8	50.3	55.6	99.6	69.2	71.6
Philippines	1993	8.9	19.3	15.8	8.9	13.0	15.3	13.4	16.3	21.8	34.3	29.0	25.0
Philippines	1998	12.5	27.7	17.0	12.5	14.2	21.1	15.4	22.7	26.5	48.2	32.1	34.9
Philippines	2003	10.7	21.0	16.7	10.7	13.0	15.9	13.6	16.4	23.5	36.5	30.1	26.9
Timor-Leste	2009/10	2.2	17.3	14.0	2.2	15.9	14.8	13.0	18.8	18.1	31.8	26.8	20.9
Bolivia	1993/94	12.3	17.6	17.6	12.3	21.0	17.0	17.0	22.6	33.1	34.3	34.3	34.6
Bolivia	2003/4	11.8	23.2	20.9	11.8	16.4	18.6	17.3	19.2	28.0	41.4	37.8	30.8
Bolivia	2008	11.5	25.6	20.3	11.5	17.1	20.5	17.7	22.0	28.4	45.6	37.6	33.3
Brazil	1991/92	13.6	25.4	21.5	13.6	16.3	19.3	17.2	19.6	29.7	44.2	38.4	32.9
Brazil	1996	9.3	12.4	12.4	9.3	13.9	9.8	9.8	13.0	23.1	22.1	22.1	22.2
Colombia	1995	9.6	22.8	16.4	9.6	14.2	17.2	13.8	18.9	23.6	39.6	30.0	28.3
Colombia	2000	11.6	13.3	13.3	11.6	11.6	9.9	9.9	11.8	23.0	23.1	23.1	23.2
Colombia	2004/5	6.6	12.9	9.9	6.6	7.6	9.2	7.6	9.5	14.1	22.0	17.3	16.0
Colombia	2009/10	5.4	6.4	5.5	5.4	4.8	5.5	5.0	6.4	10.2	11.8	10.5	11.8
Colombia	2015/16	7.8	7.9	7.9	7.8	4.6	4.3	4.3	4.4	12.3	12.2	12.2	12.2
Dominican Republic	1991	15.9	27.9	22.8	15.9	16.5	21.0	18.3	20.8	32.2	48.3	40.7	36.4
Dominican Republic	1996	18.9	33.8	26.5	18.9	19.4	24.3	20.4	24.4	38.0	57.4	46.4	42.9
Dominican Republic	2002	7.9	18.6	17.1	7.9	14.7	14.8	14.0	16.1	22.5	33.2	30.9	23.9
Guatemala	1995	10.8	16.5	16.5	10.8	19.5	13.0	13.0	18.9	30.1	29.2	29.2	29.5
Guatemala	1998/99	16.1	19.6	19.6	16.1	16.7	13.5	13.5	17.2	32.6	32.8	32.8	33.0
Guatemala	2014/15	10.1	18.5	13.8	10.1	10.1	13.7	11.2	13.8	20.1	32.0	24.9	23.8
Honduras	2005/6	12.1	14.0	12.3	12.1	9.7	9.8	8.9	11.8	21.7	23.6	21.1	23.7
Honduras	2011/12	9.4	13.6	13.0	9.4	8.3	10.1	9.7	9.1	17.6	23.6	22.6	18.4
Nicaragua	1997/98	7.8	18.3	14.5	7.7	12.5	13.4	11.3	14.7	20.1	31.5	25.6	22.4
Nicaragua	2001	9.5	20.7	13.8	9.5	11.4	15.9	12.3	17.0	20.8	36.3	26.0	26.3
Paraguay	1990	16.8	24.2	22.7	16.8	14.4	17.6	16.8	15.7	31.0	41.4	39.1	32.3
Peru	1991/92	9.4	23.5	19.2	9.4	17.0	18.7	16.4	20.7	26.3	41.8	35.4	29.9
Peru	1996	9.3	13.8	13.8	9.3	16.4	12.5	12.5	17.1	25.5	26.1	26.1	26.3

Peru	2000	9.2	16.5	14.8	9.2	11.0	13.1	12.2	13.1	20.1	29.4	26.9	22.1
Peru	2004/6	8.0	9.9	9.9	8.0	8.6	6.7	6.7	8.6	16.5	16.5	16.5	16.6
Peru	2007/8	12.2	13.0	12.4	12.2	5.8	5.9	5.7	6.6	18.0	18.8	18.0	18.8
Peru	2009	8.6	11.0	8.7	8.6	6.4	7.2	6.3	9.7	15.0	18.1	15.0	18.2
Peru	2010	6.1	9.7	6.2	6.1	5.7	7.0	5.6	10.7	11.8	16.6	11.7	16.8
Peru	2011	8.2	9.5	8.3	8.2	4.4	4.6	4.2	5.9	12.6	14.0	12.5	14.1
Peru	2012	7.8	10.1	7.9	7.8	7.0	7.5	6.5	9.9	14.8	17.5	14.3	17.6

M1 Model 1
M2 Model 2
M3 Model 3
M4 Model 4

Appendix 9: country-specific observed and adjusted risk and cumulative risk of death

Figure S2: Angola DHS-2015/16

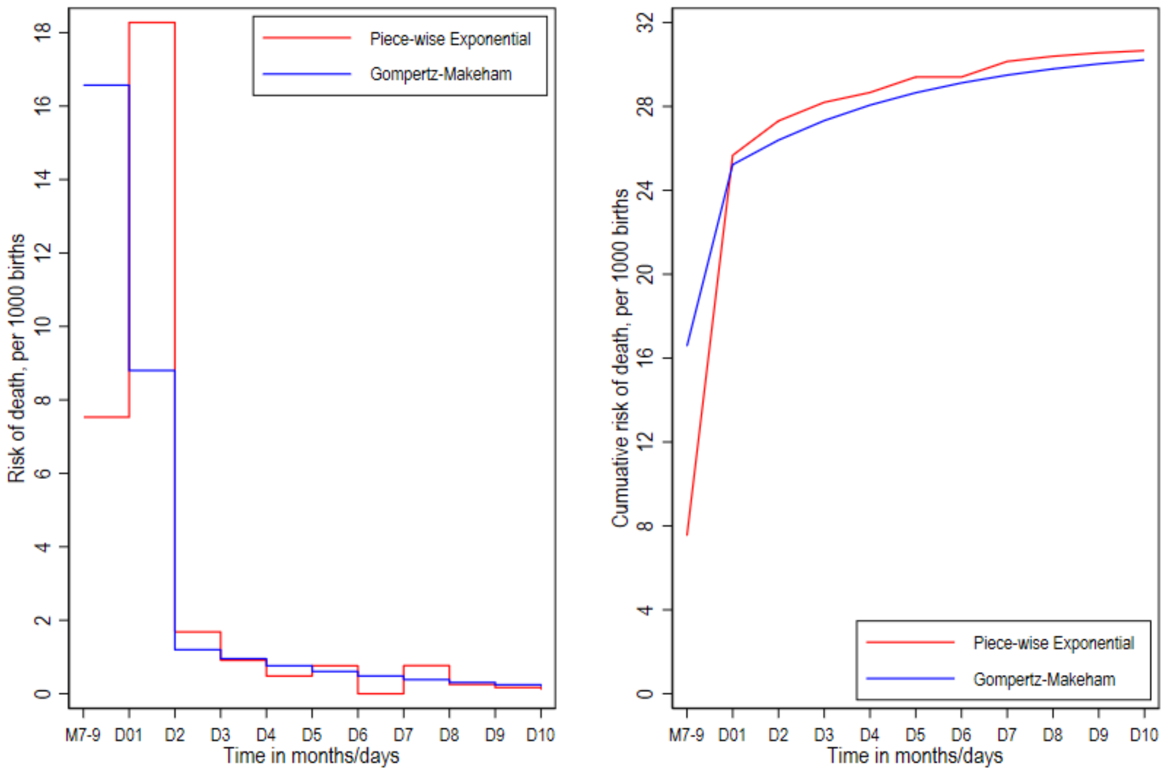

Figure S2: Benin DHS-2006

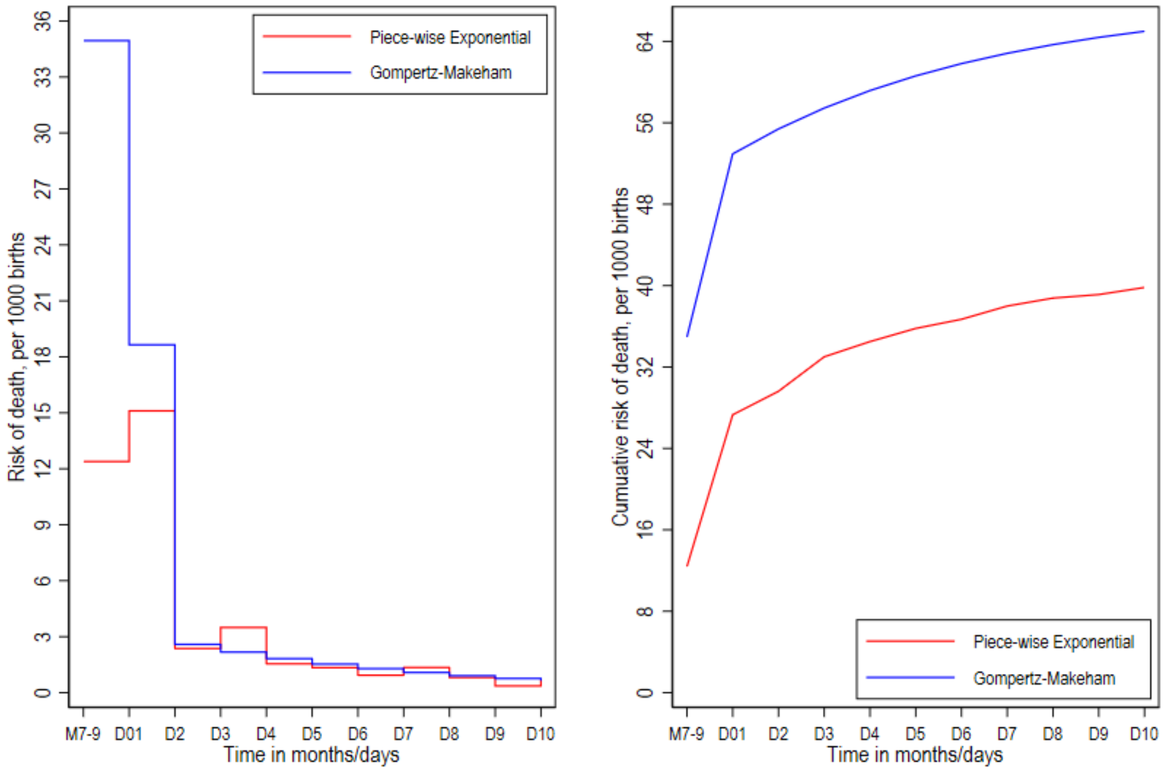

Figure S2: Benin DHS-2011/12

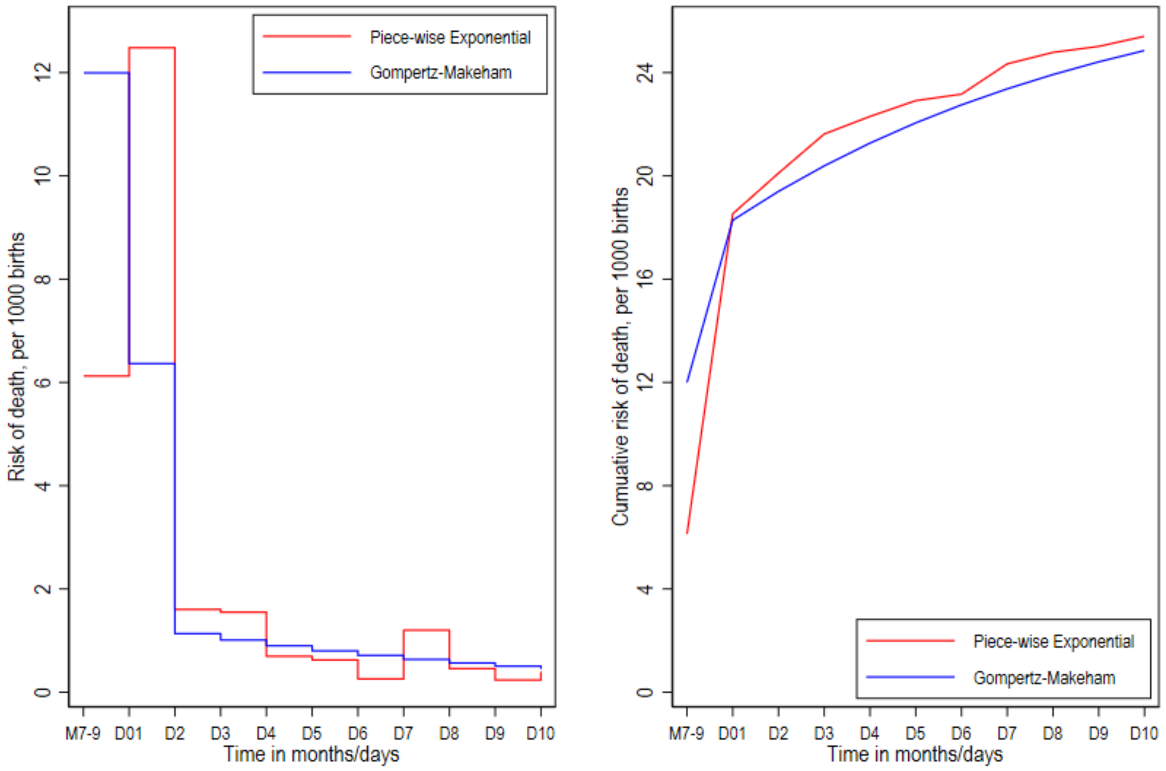

Figure S2: Benin DHS-2017/18

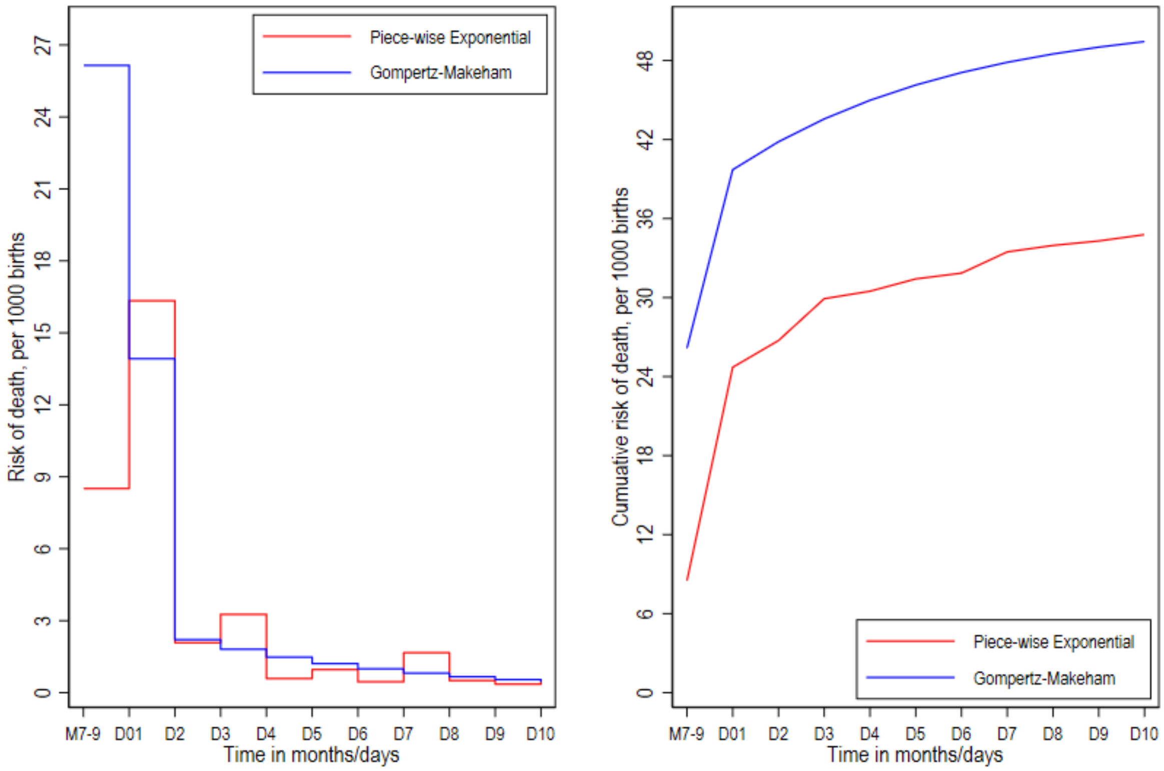

Figure S2: Burkina Faso DHS-2003

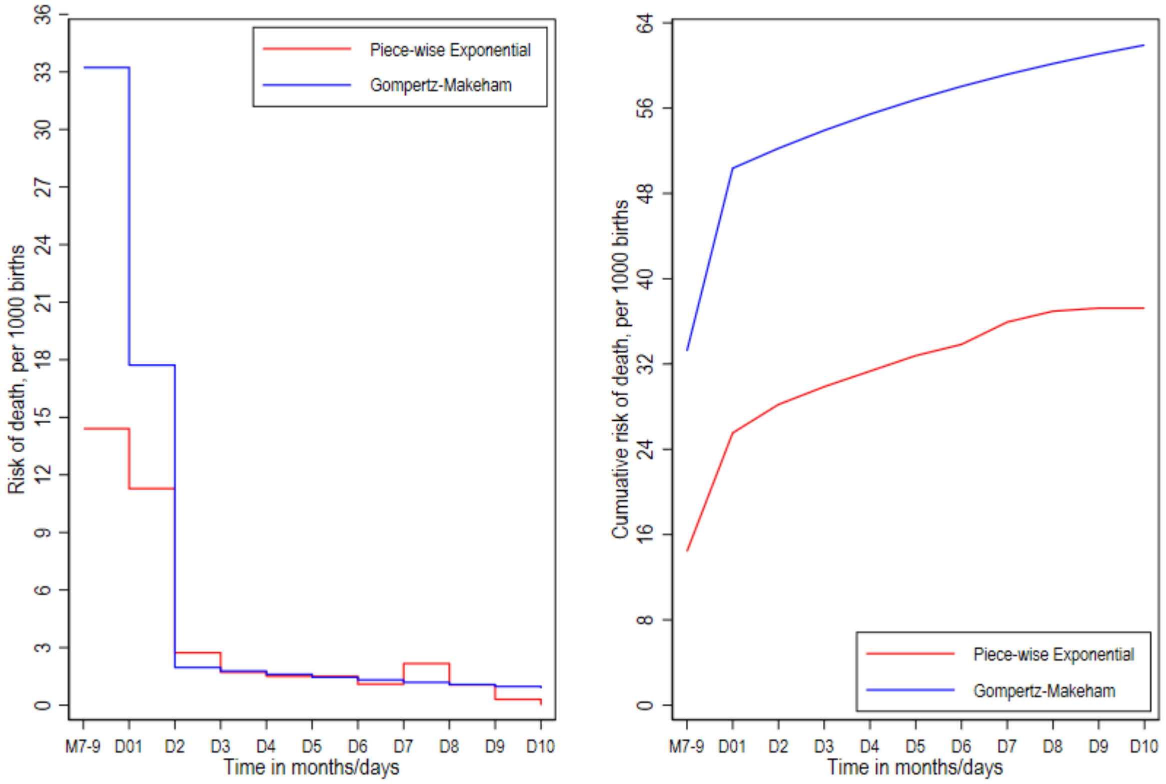

Figure S2: Burkina Faso DHS-2010

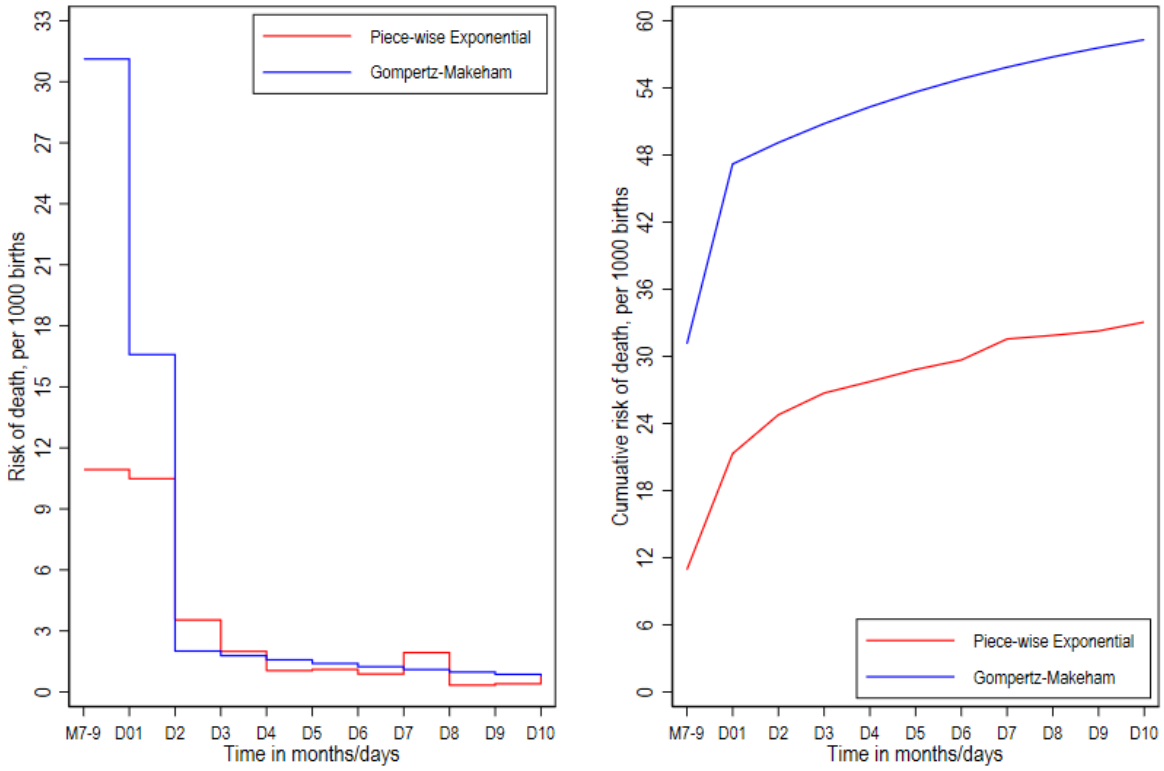

Figure S2: Burundi DHS-2010/11

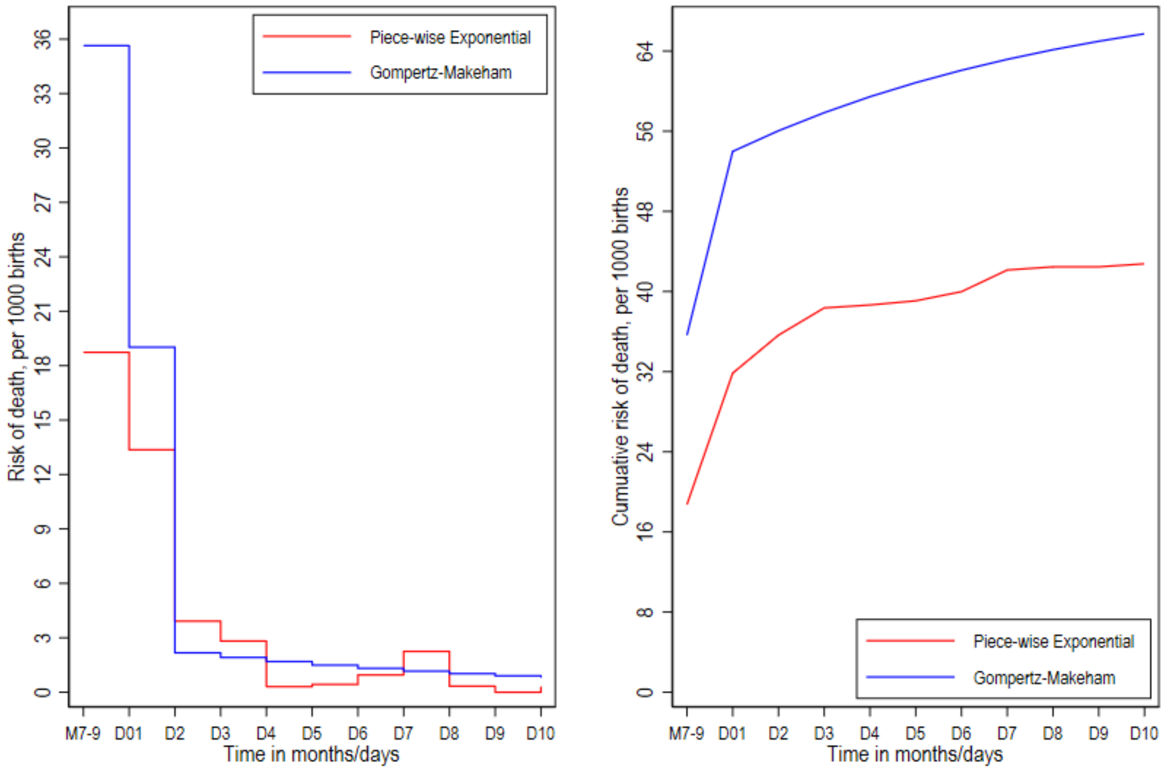

Figure S2: Ethiopia DHS-2005

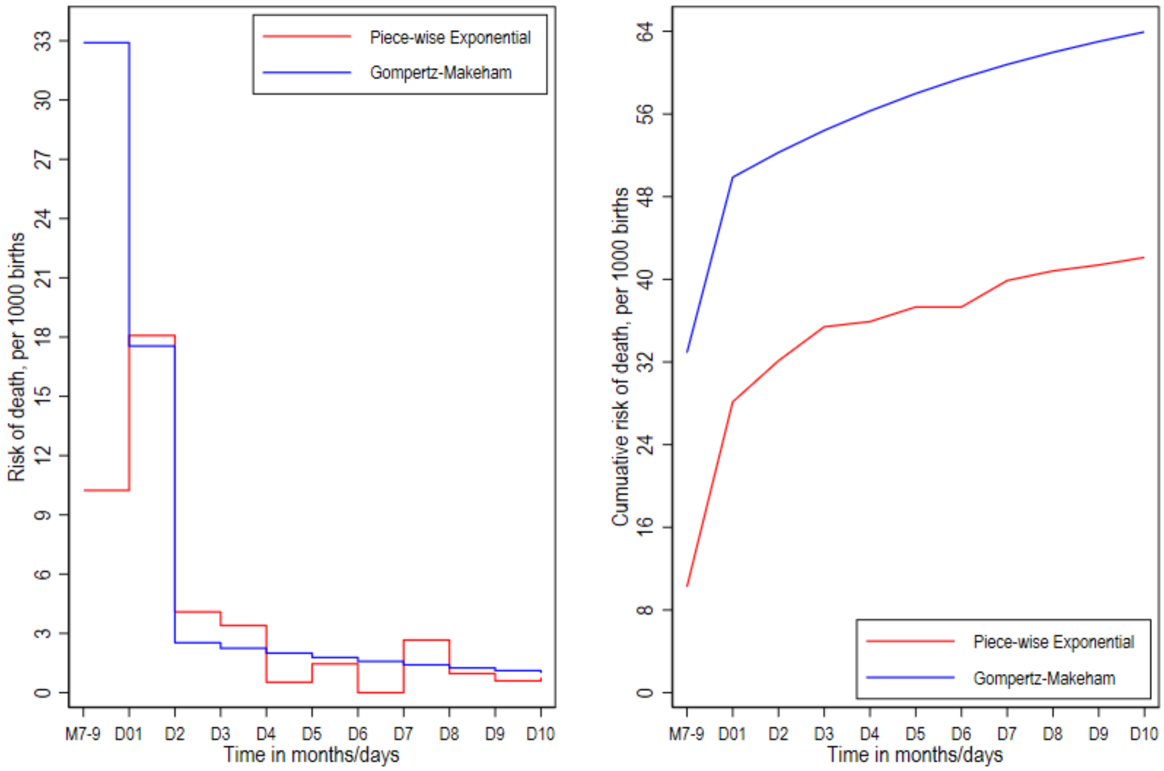

Figure S2: Ethiopia DHS-2010/11

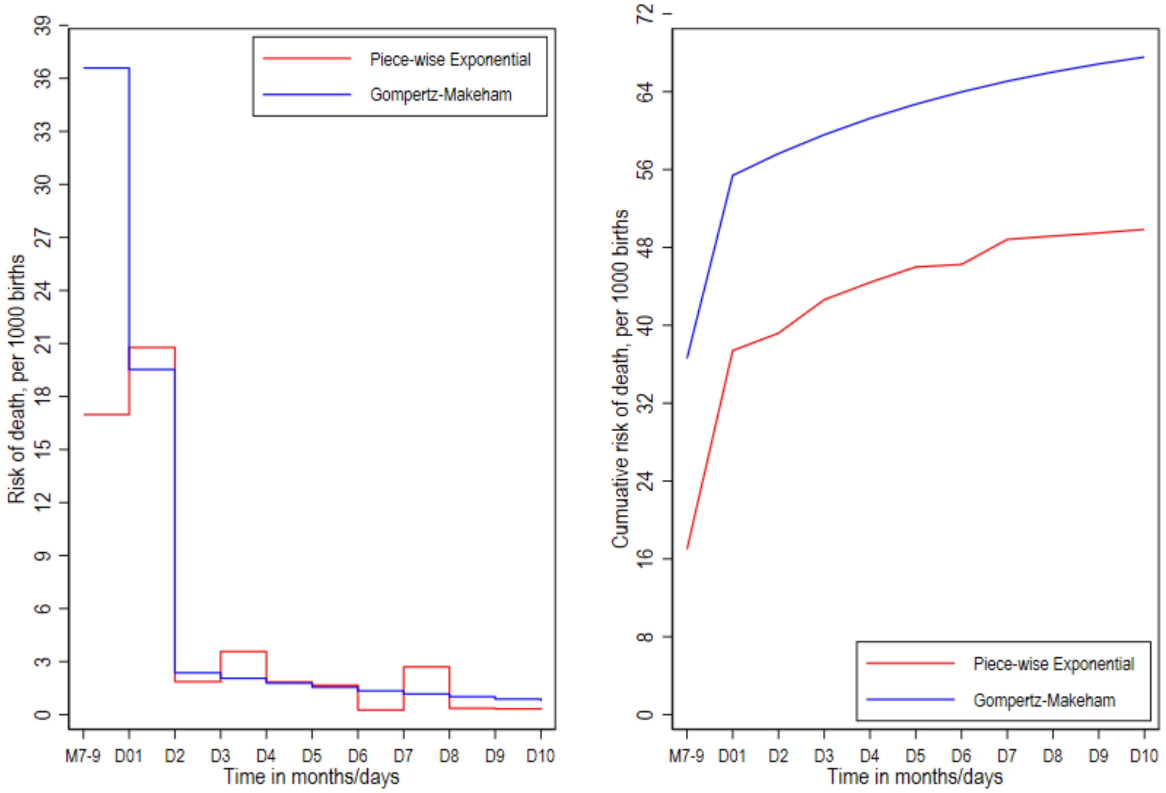

Figure S2: Ethiopia DHS-2016

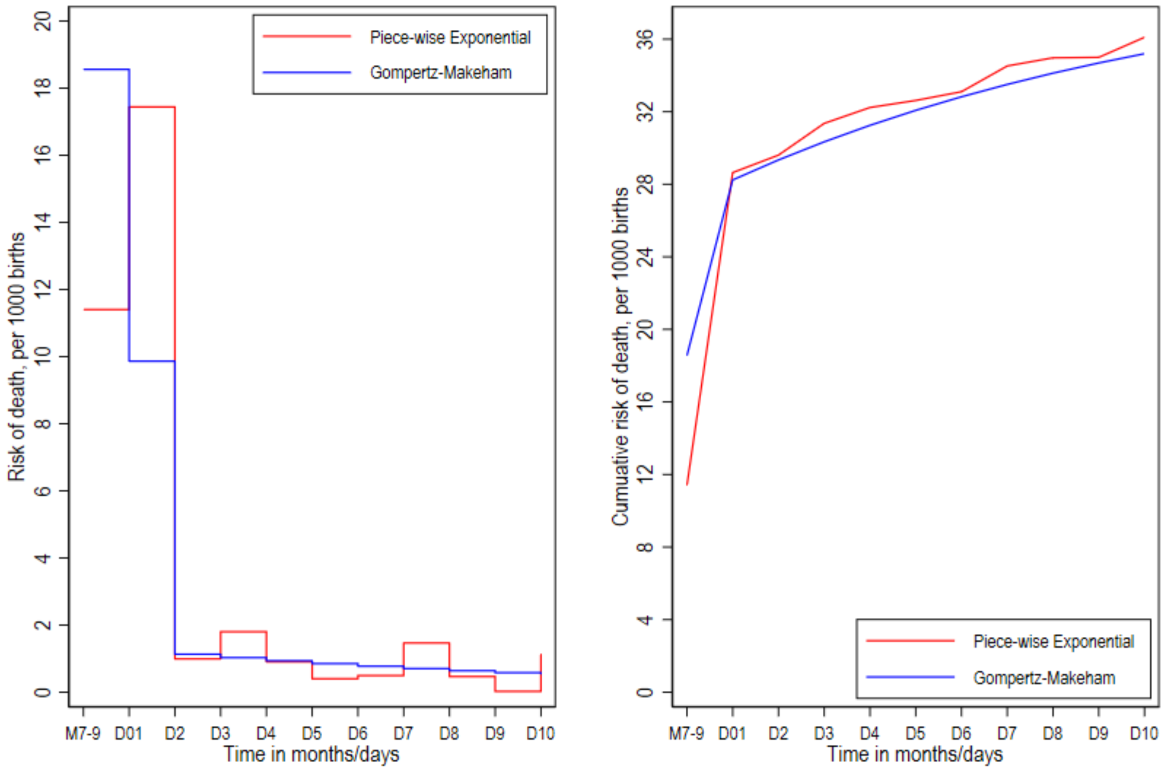

Figure S2: Gambia DHS-2013

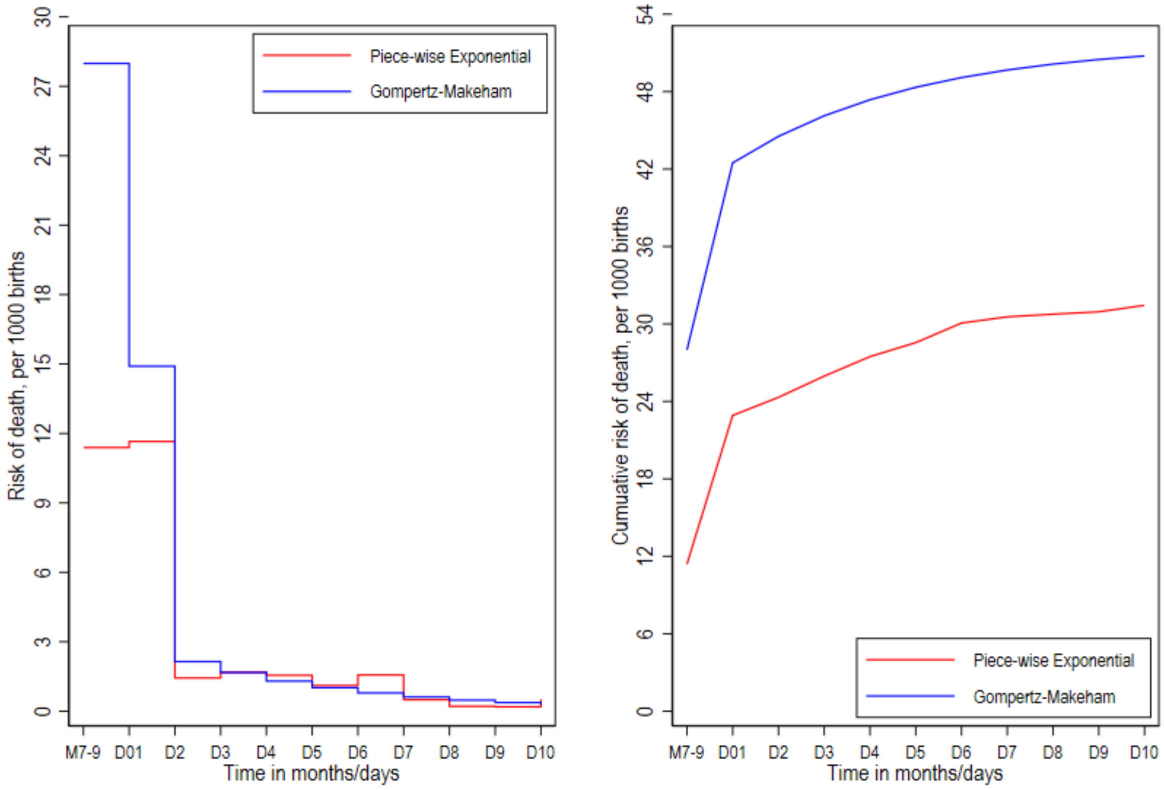

Figure S2: Gambia DHS-2019/20

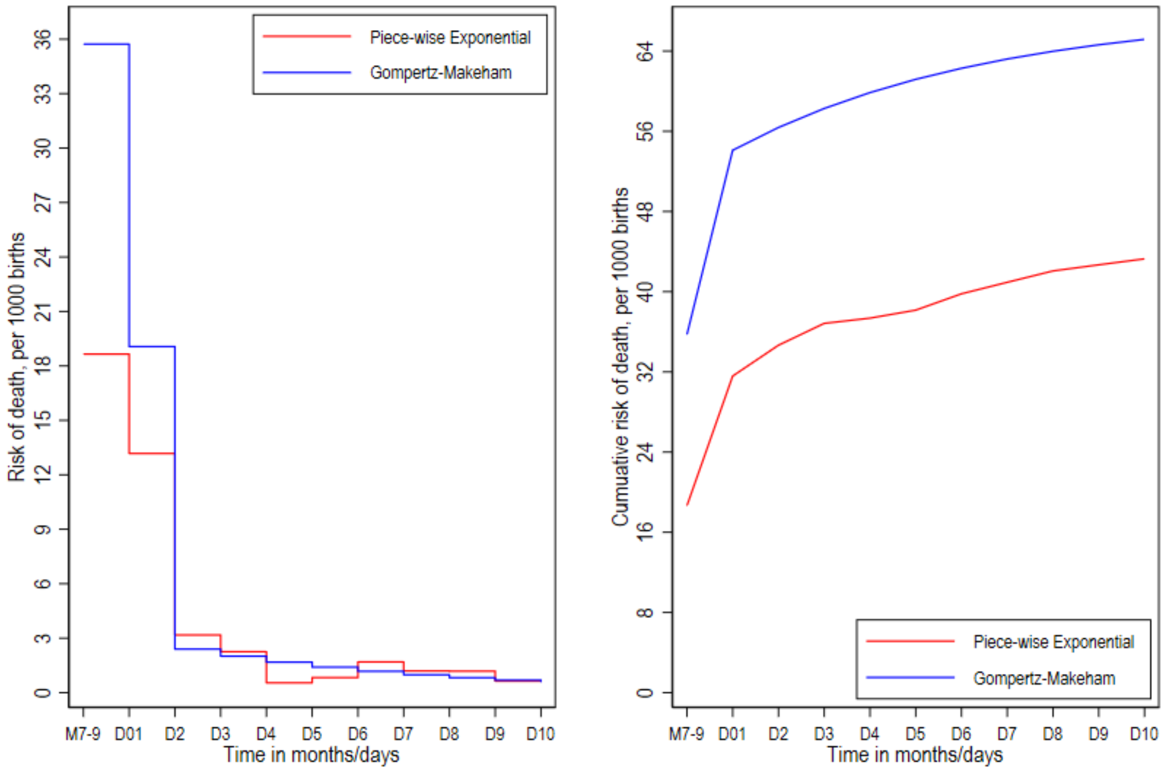

Figure S2: Ghana DHS-2003

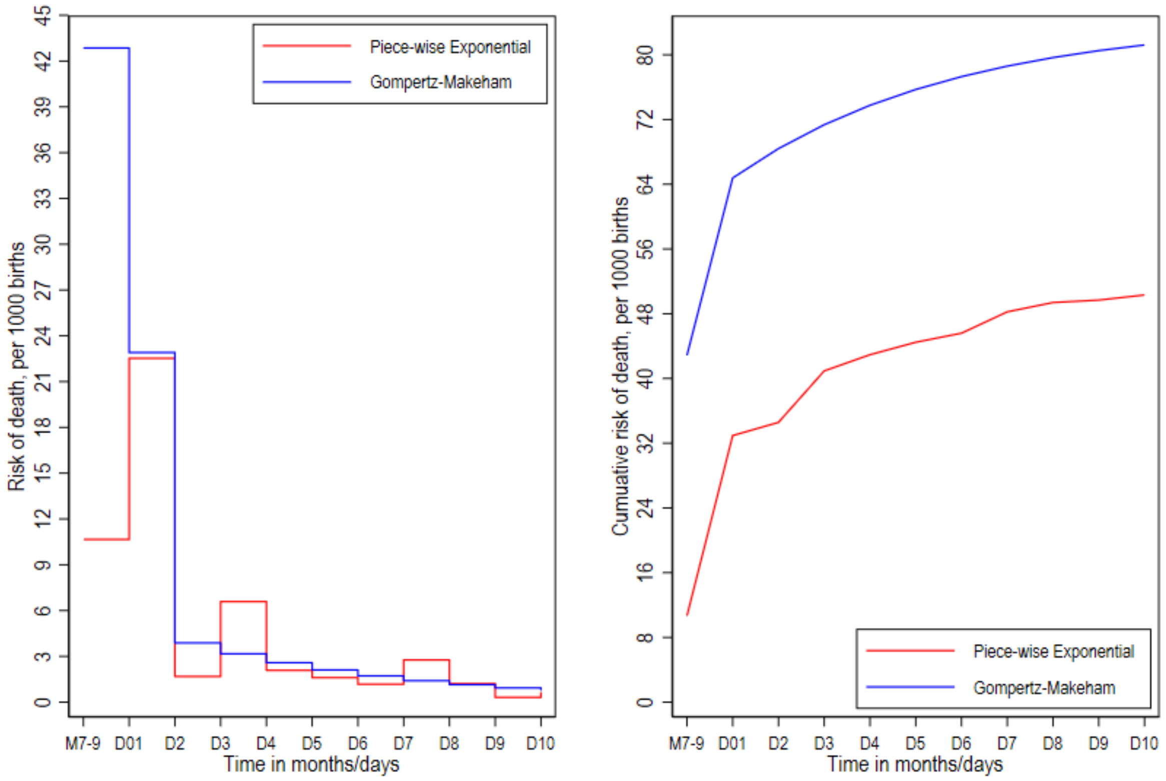

Figure S2: Ghana DHS-2008

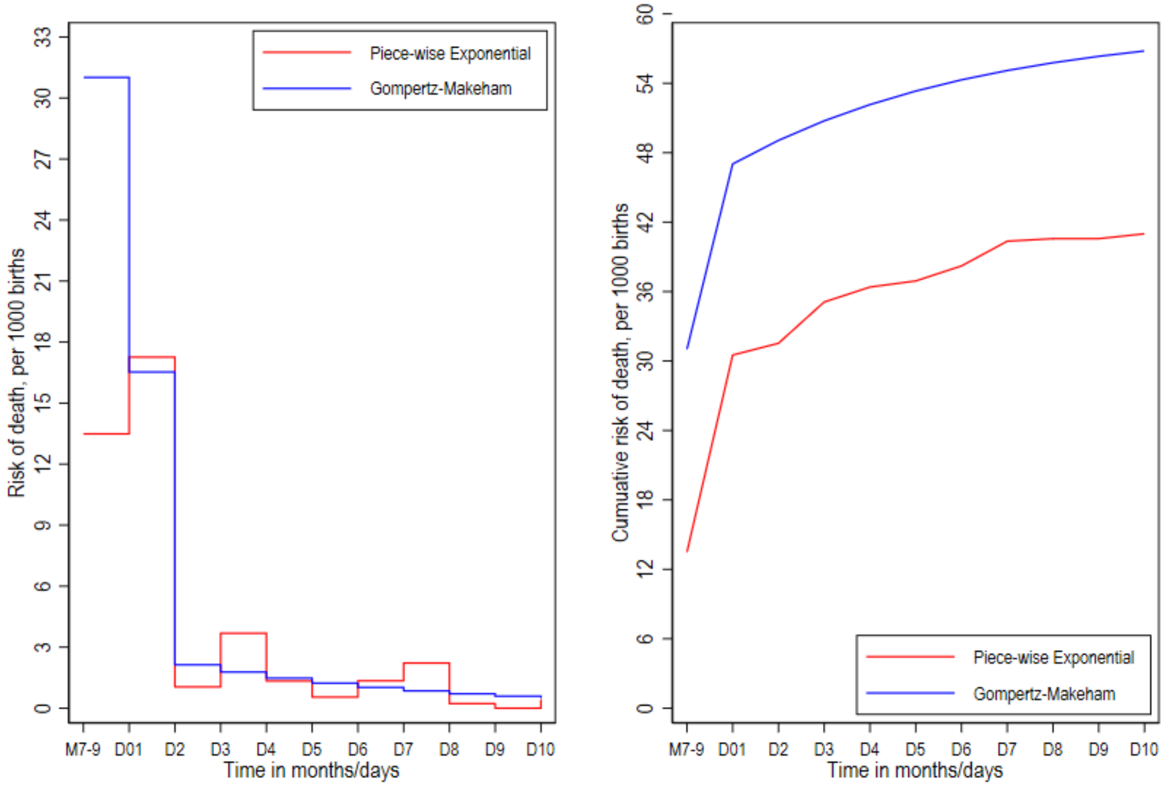

Figure S2: Ghana DHS-2014

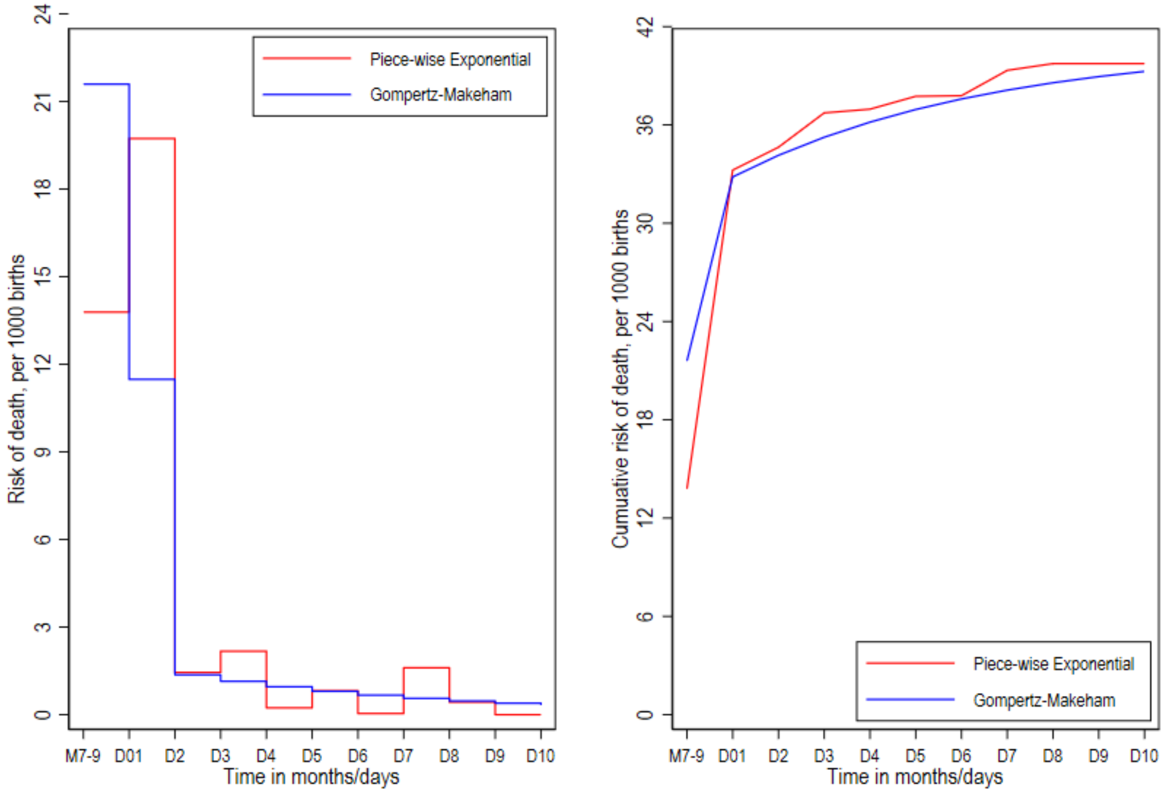

Figure S2: Guinea DHS-2005

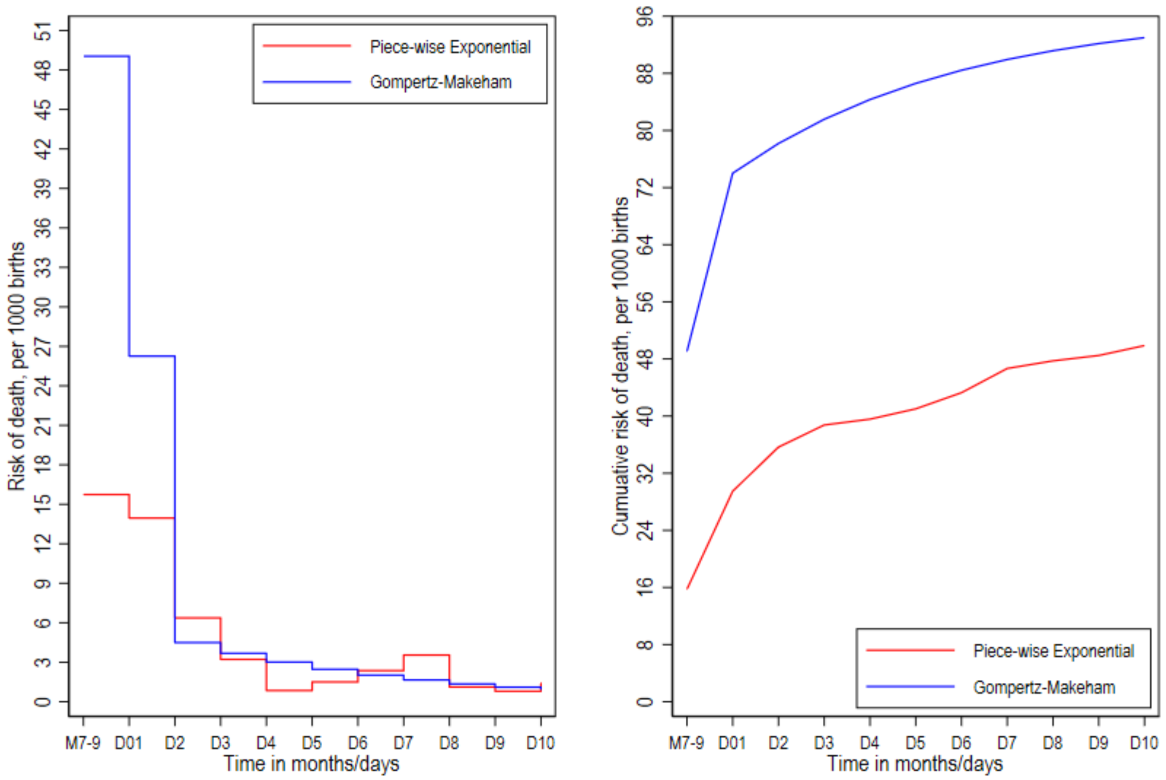

Figure S2: Guinea DHS-2018

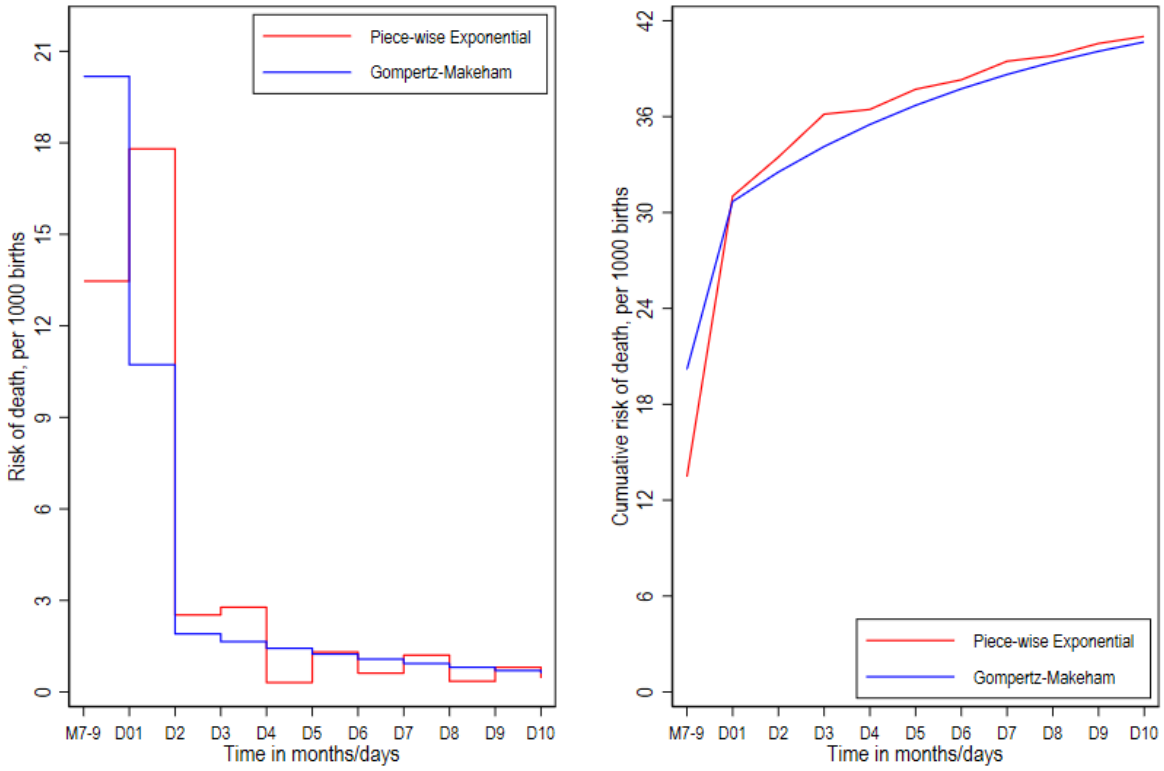

Figure S2: Kenya DHS-1998

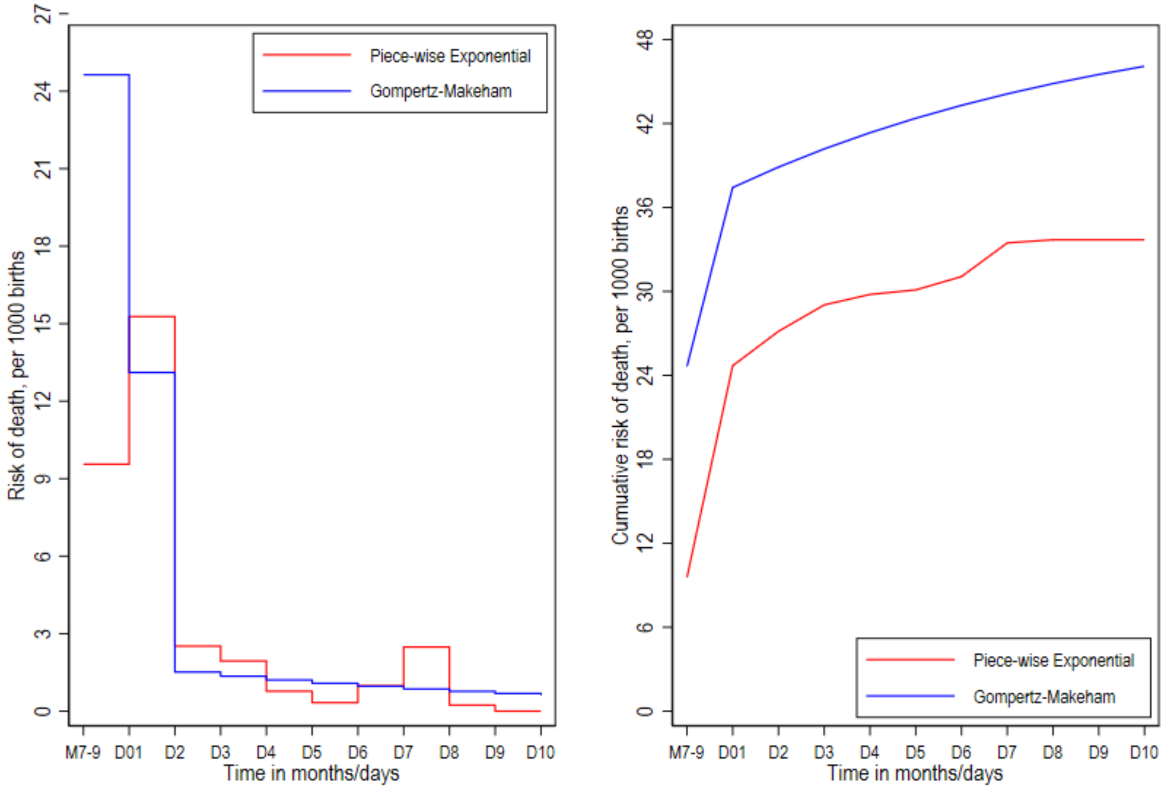

Figure S2: Kenya DHS-2003

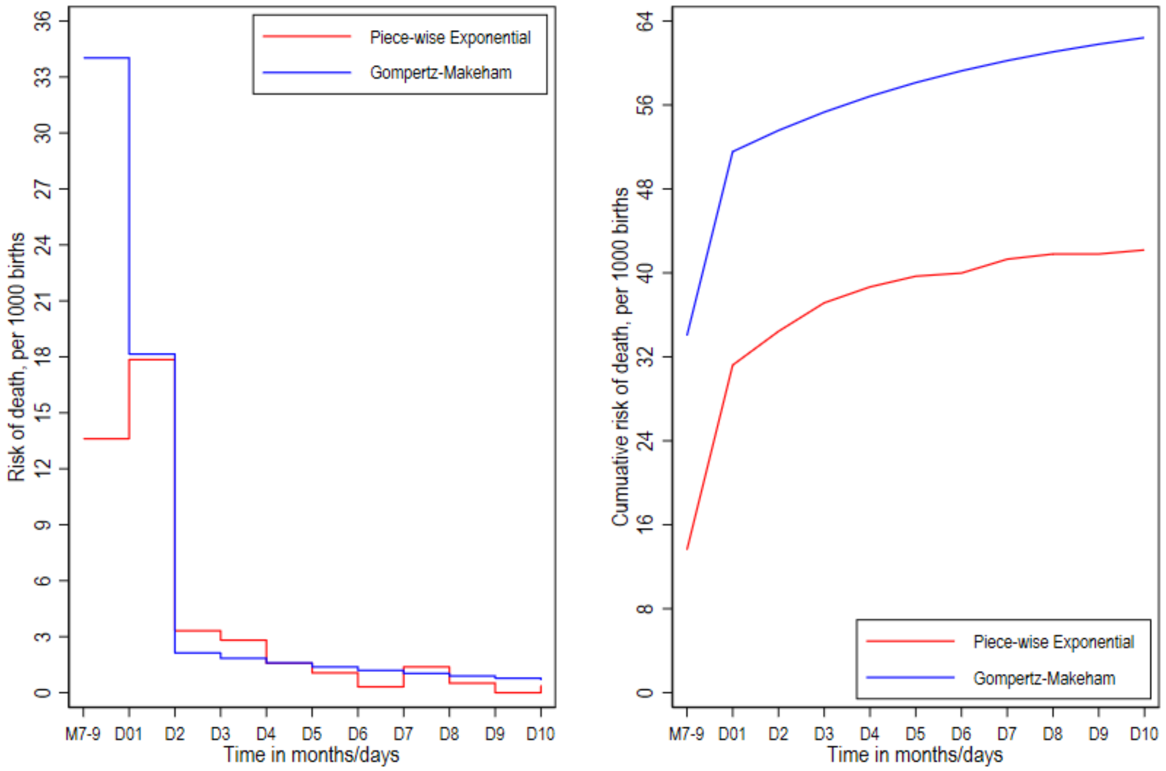

Figure S2: Kenya DHS-2008/9

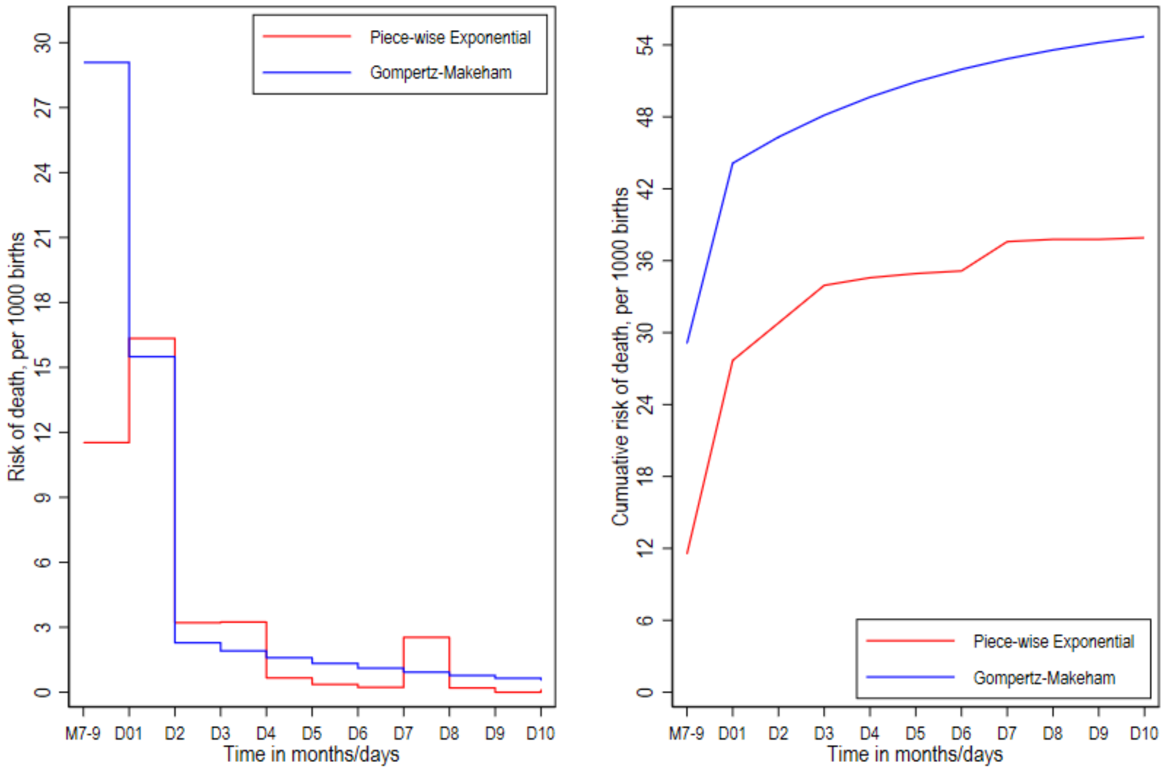

Figure S2: Kenya DHS-2014

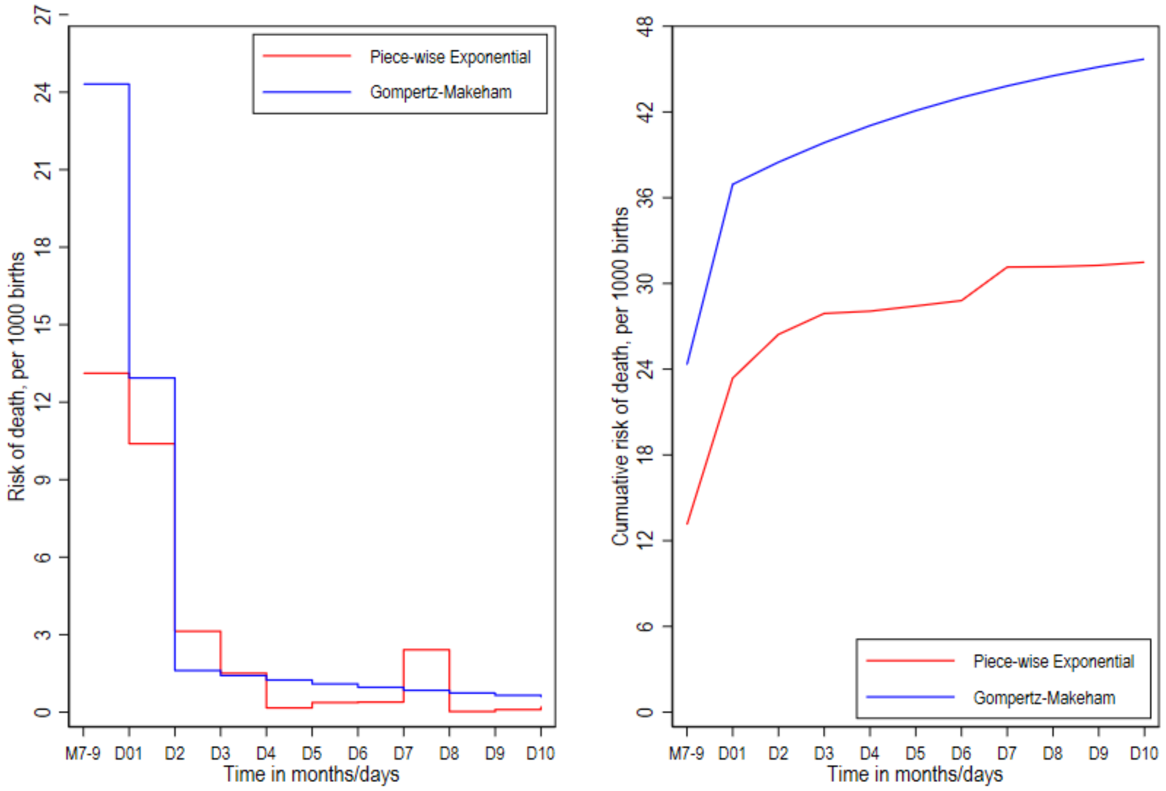

Figure S2: Lesotho DHS-2009/10

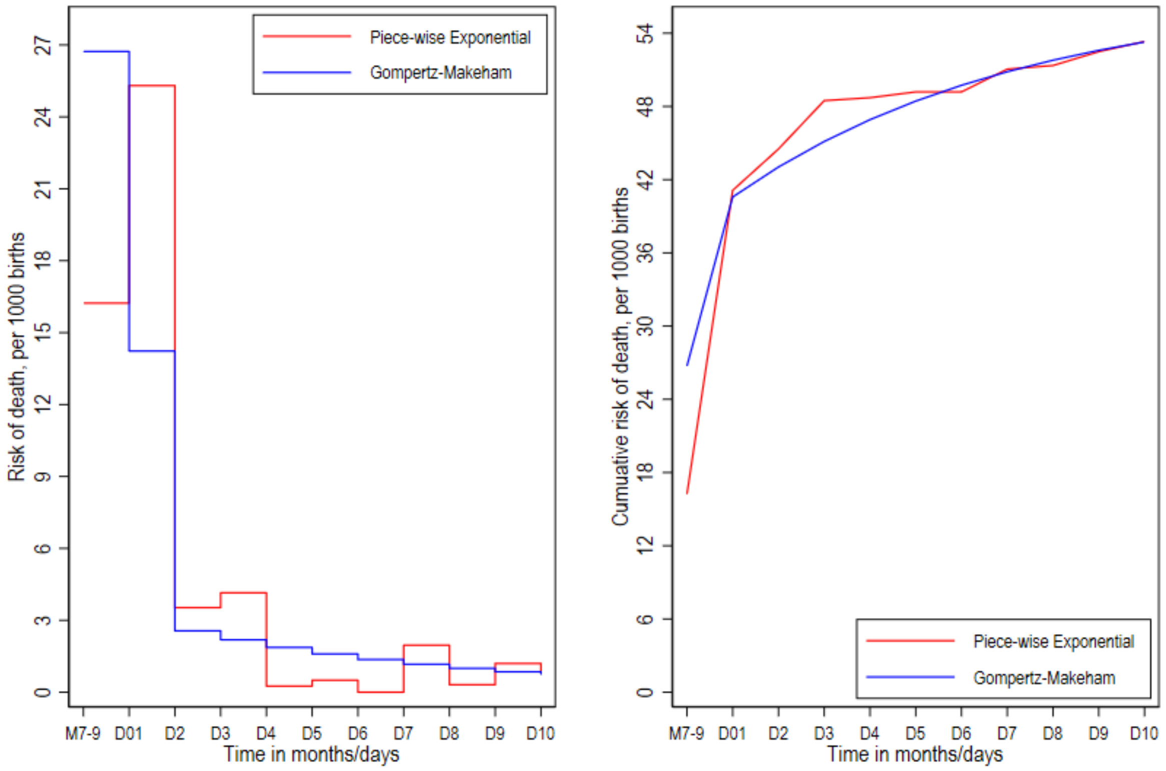

Figure S2: Lesotho DHS-2014

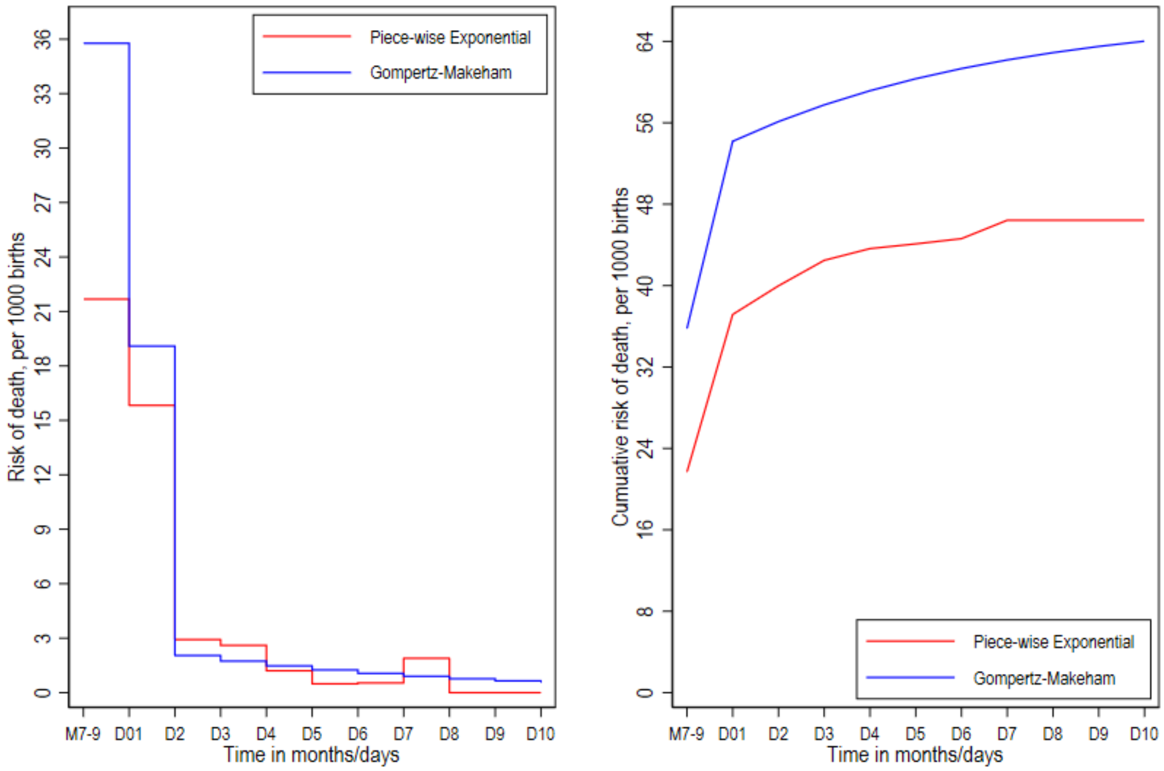

Figure S2: Liberia DHS-2013

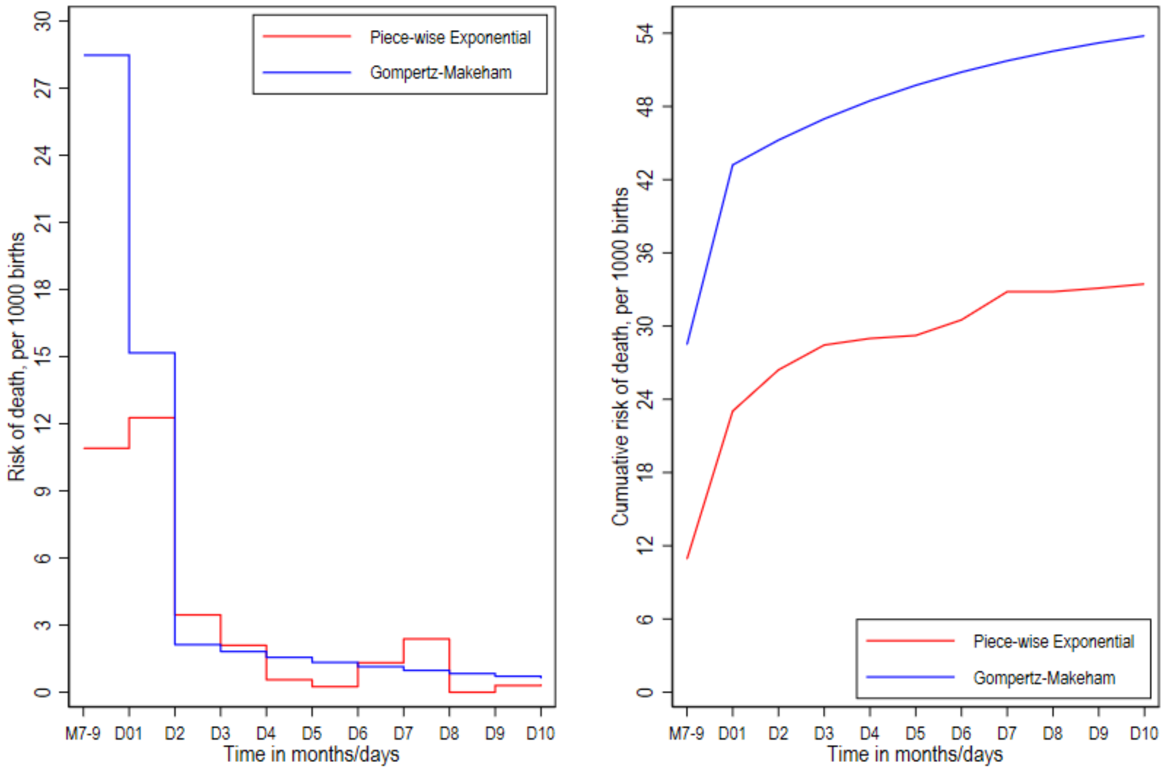

Figure S2: Liberia DHS-2019/20

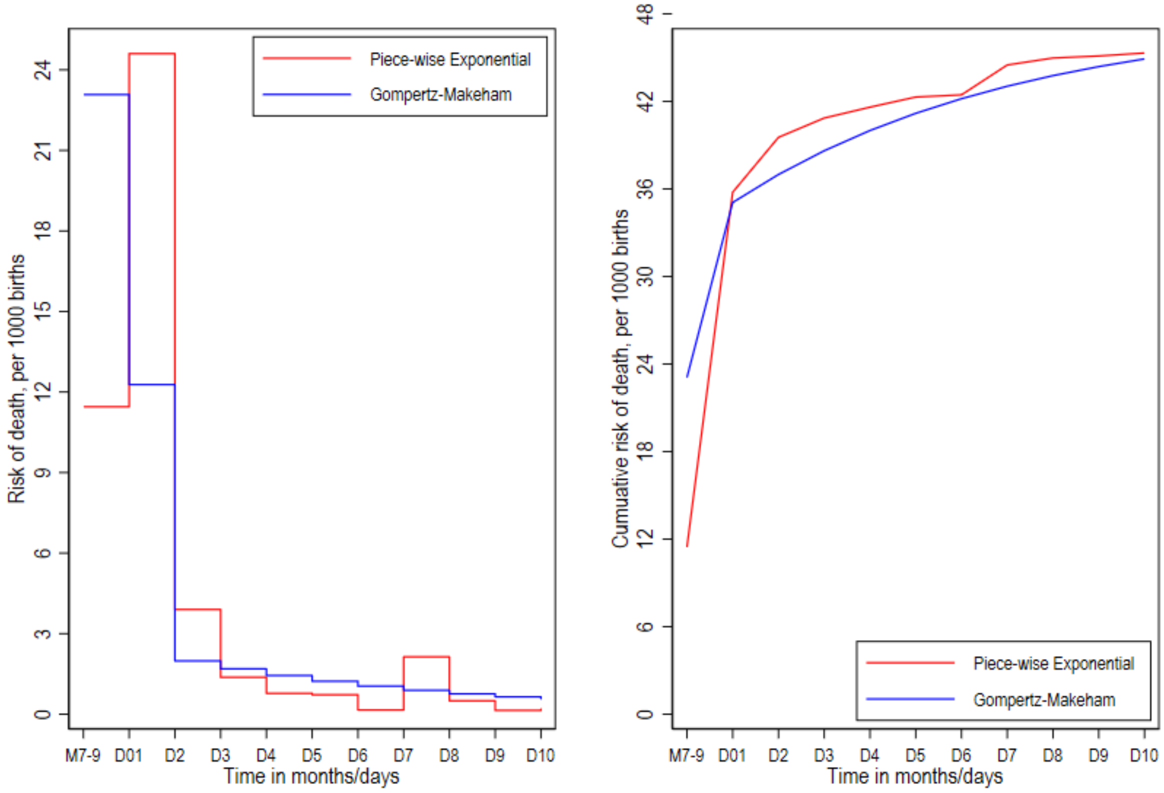

Figure S2: Madagascar DHS-2003/4

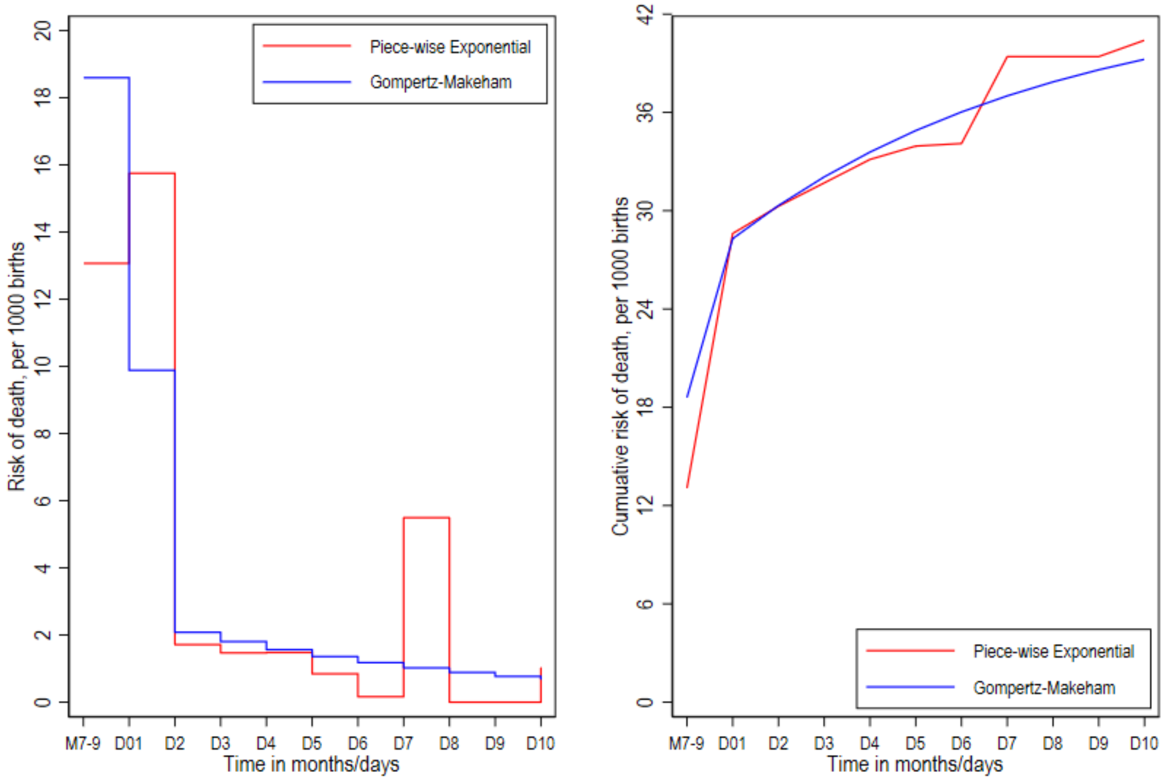

Figure S2: Madagascar DHS-2008/9

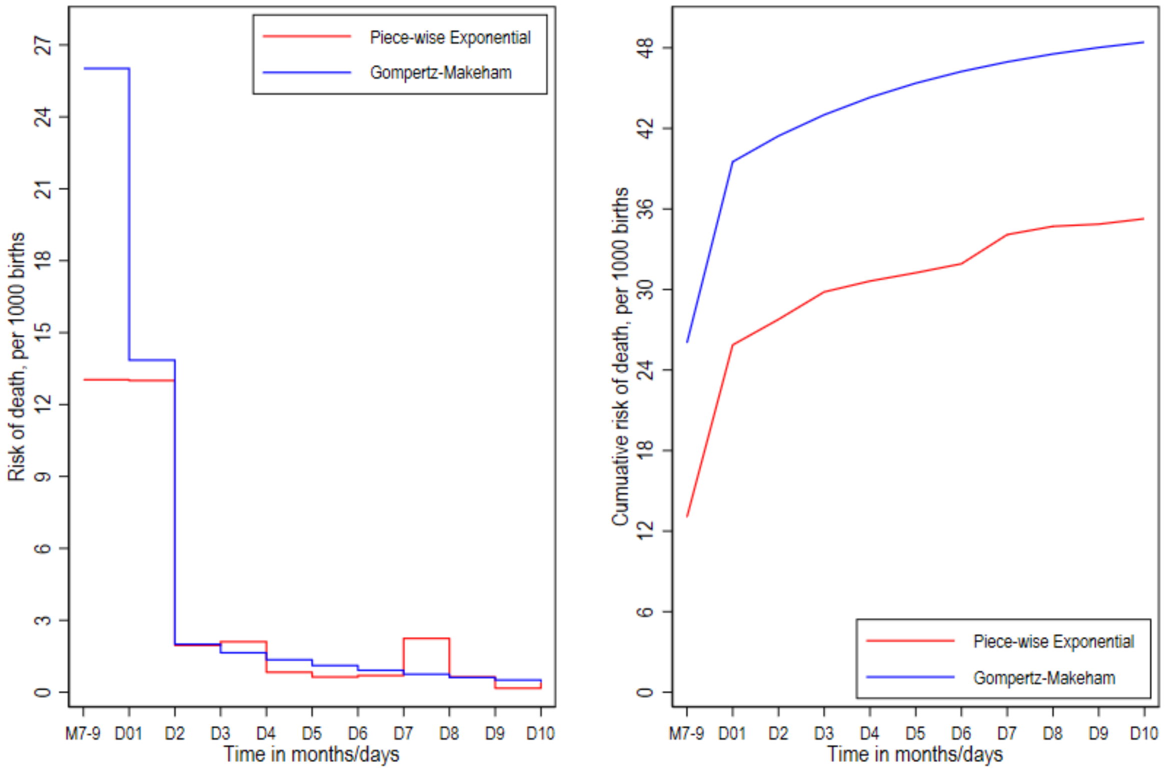

Figure S2: Malawi DHS-2000

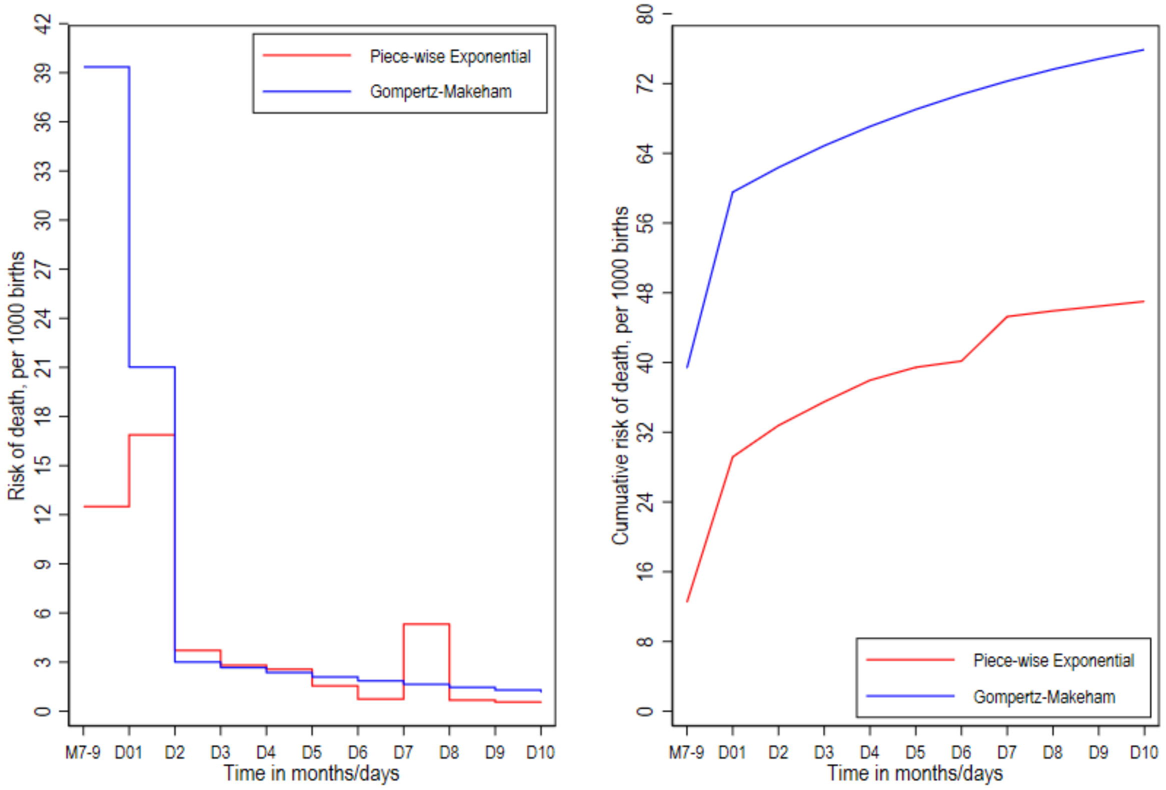

Figure S2: Malawi DHS-2004/5

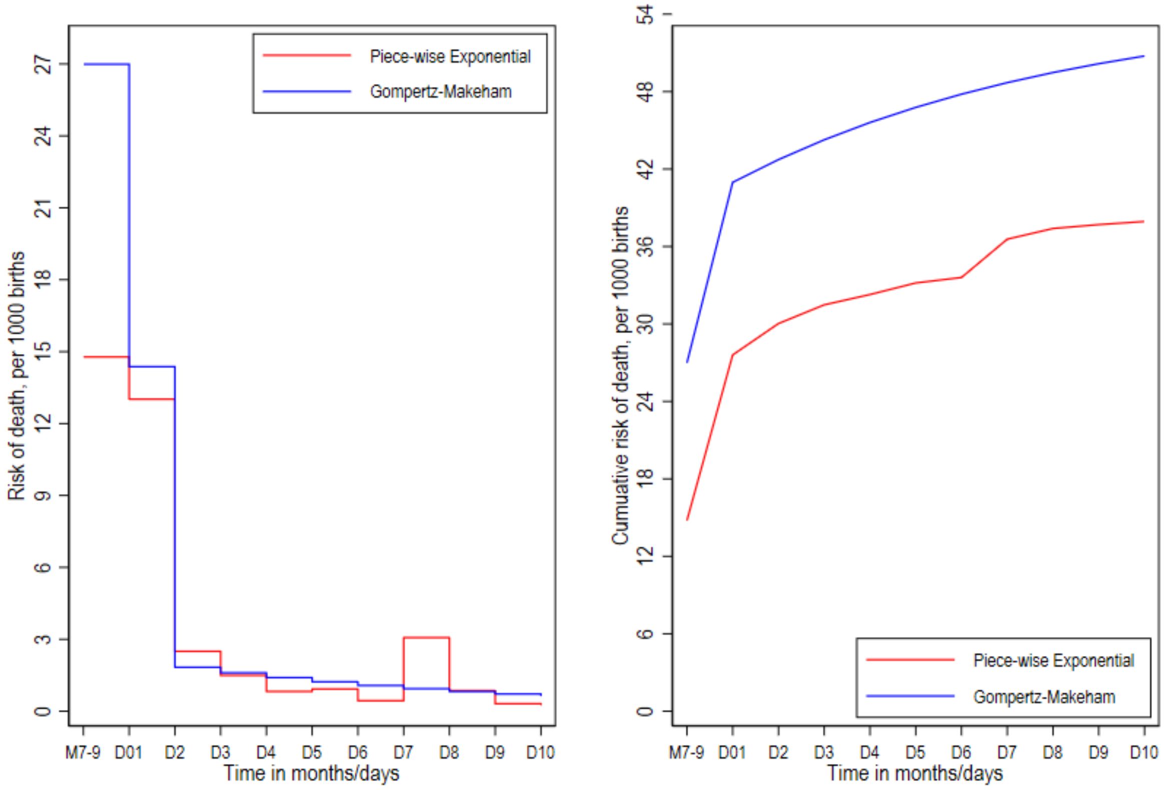

Figure S2: Malawi DHS-2010

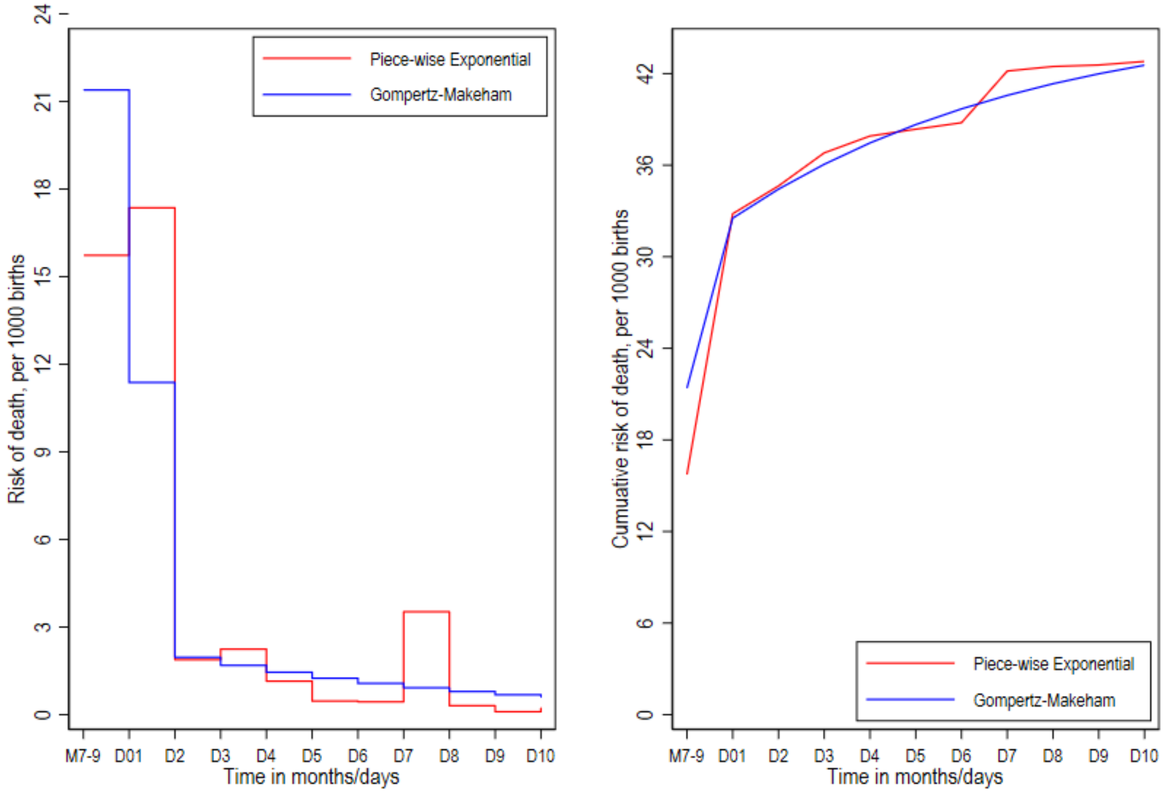

Figure S2: Malawi DHS-2015/16

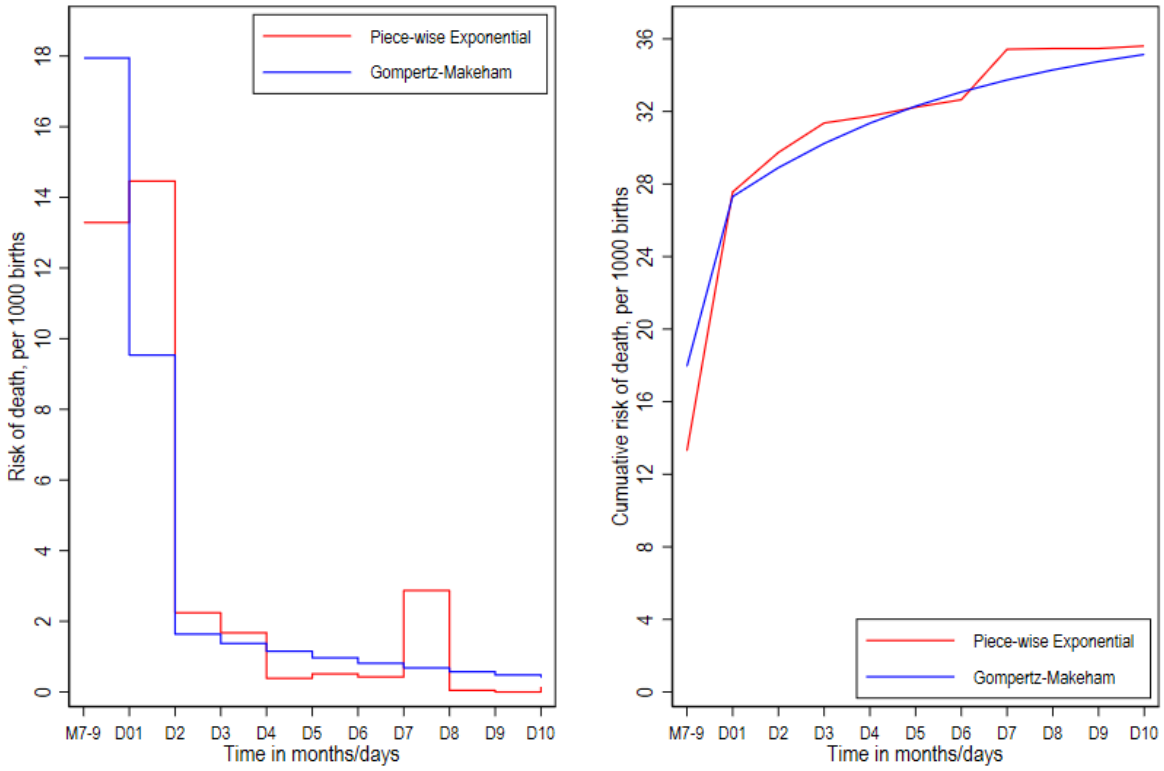

Figure S2: Mali DHS-2001

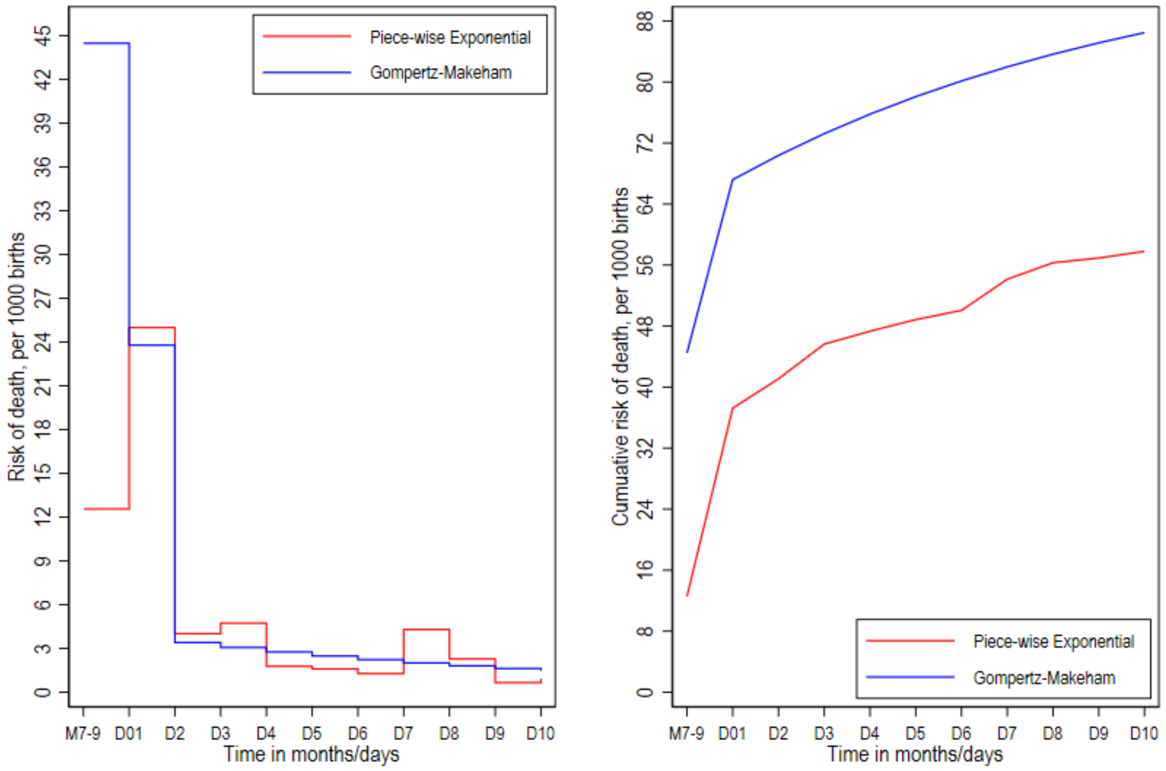

Figure S2: Mali DHS-2006

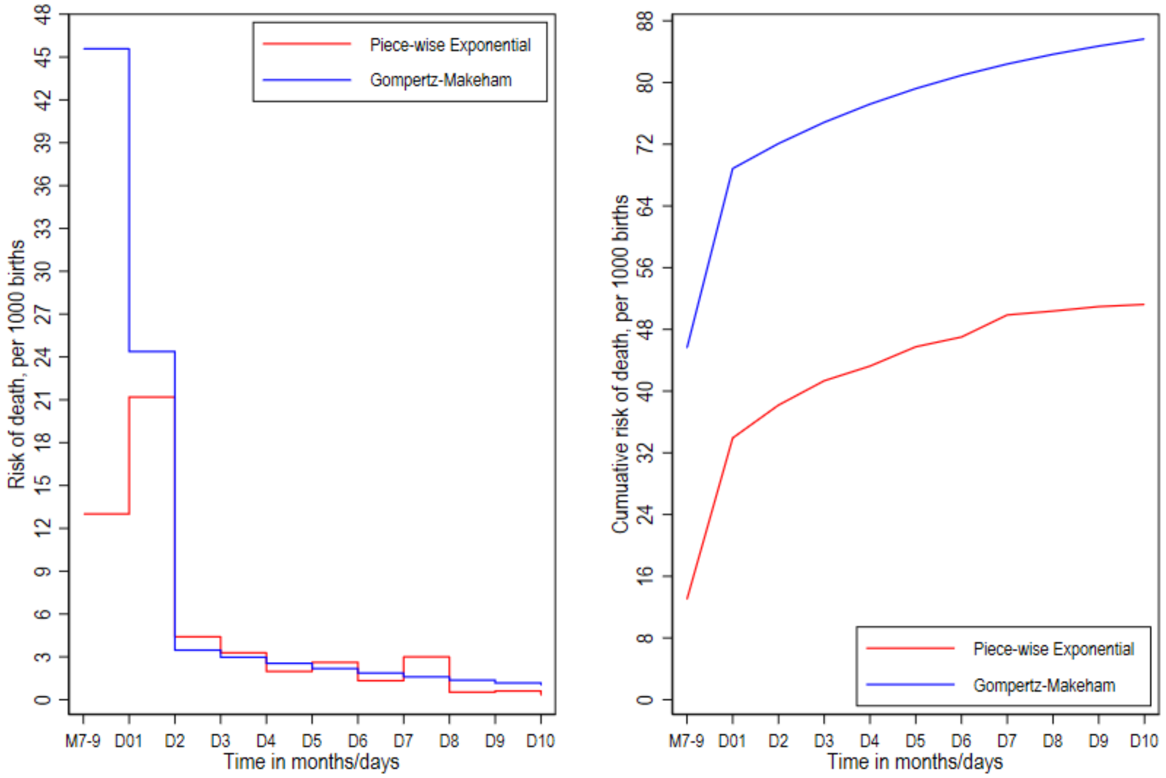

Figure S2: Mali DHS-2012/13

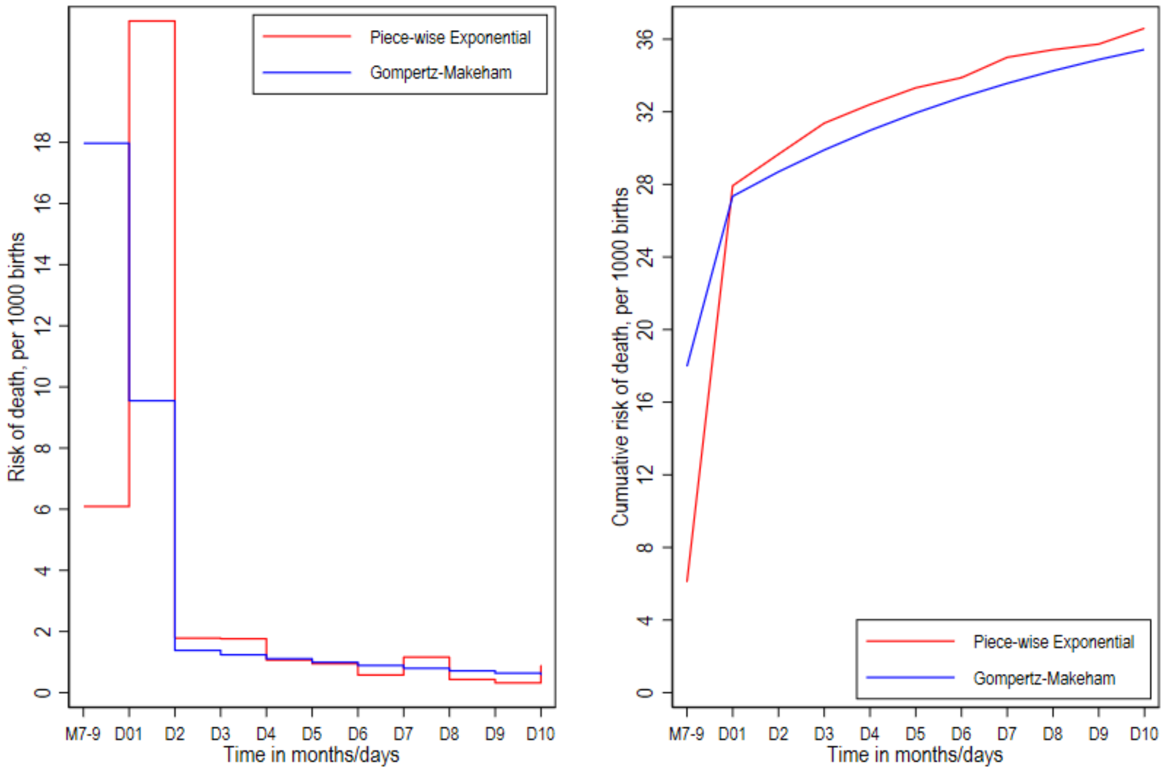

Figure S2: Mali DHS-2018

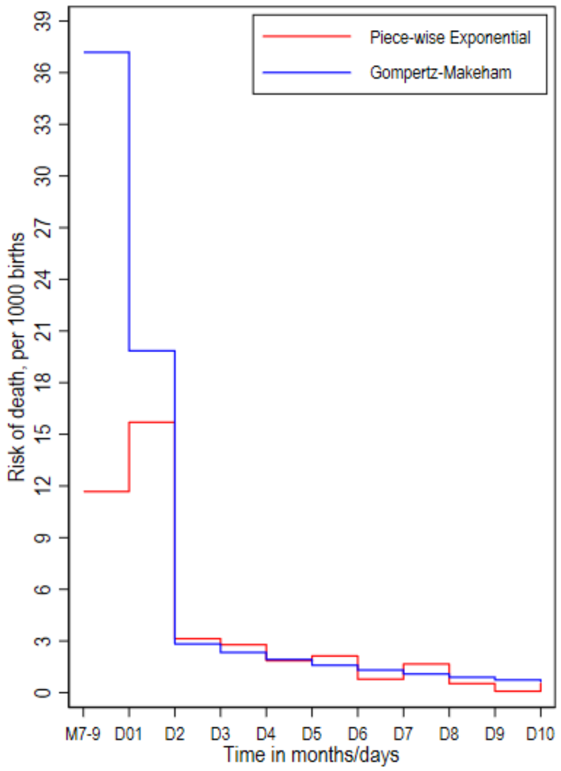

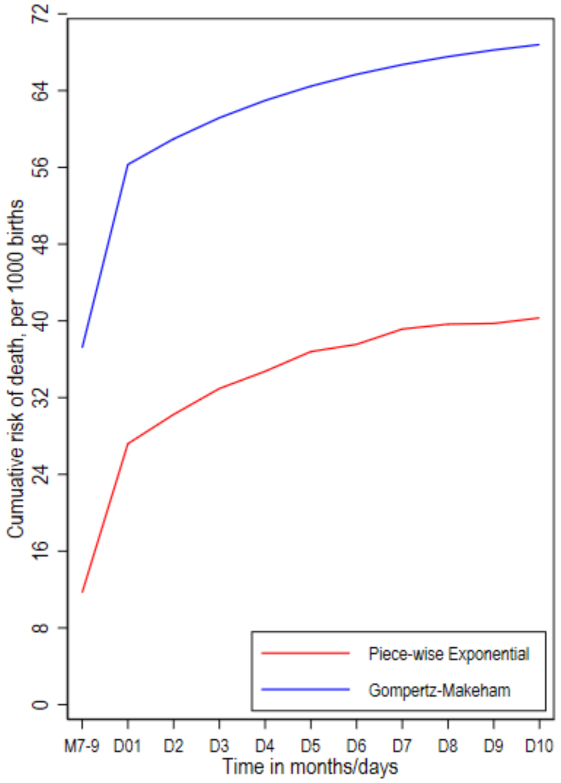

Figure S2: Mozambique DHS-2003/4

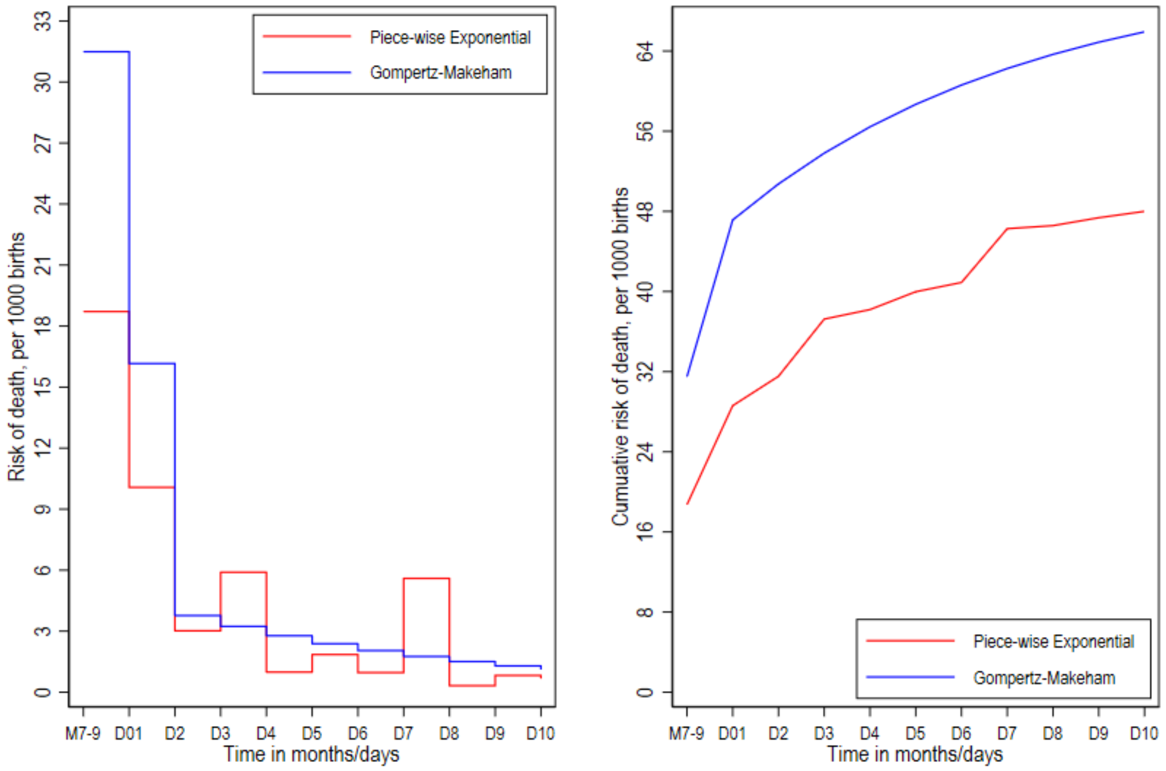

Figure S2: Mozambique DHS-2011

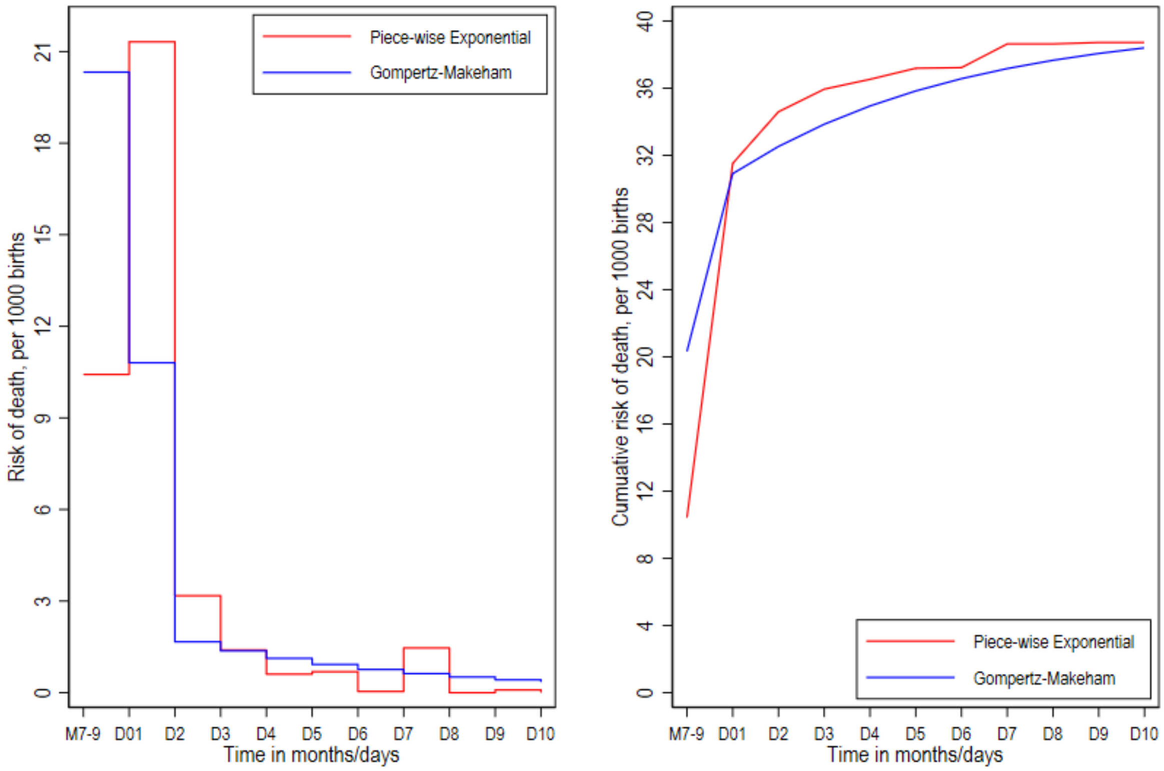

Figure S2: Namibia DHS-2006/7

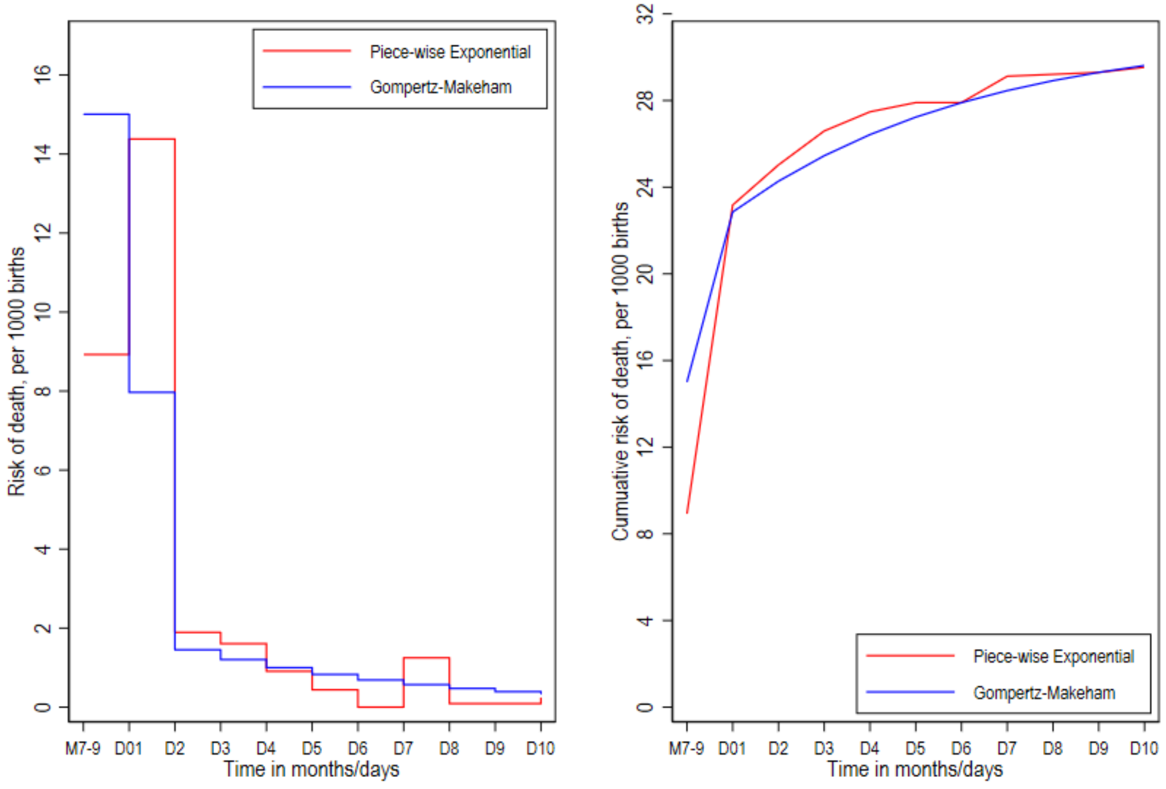

Figure S2: Namibia DHS-2013

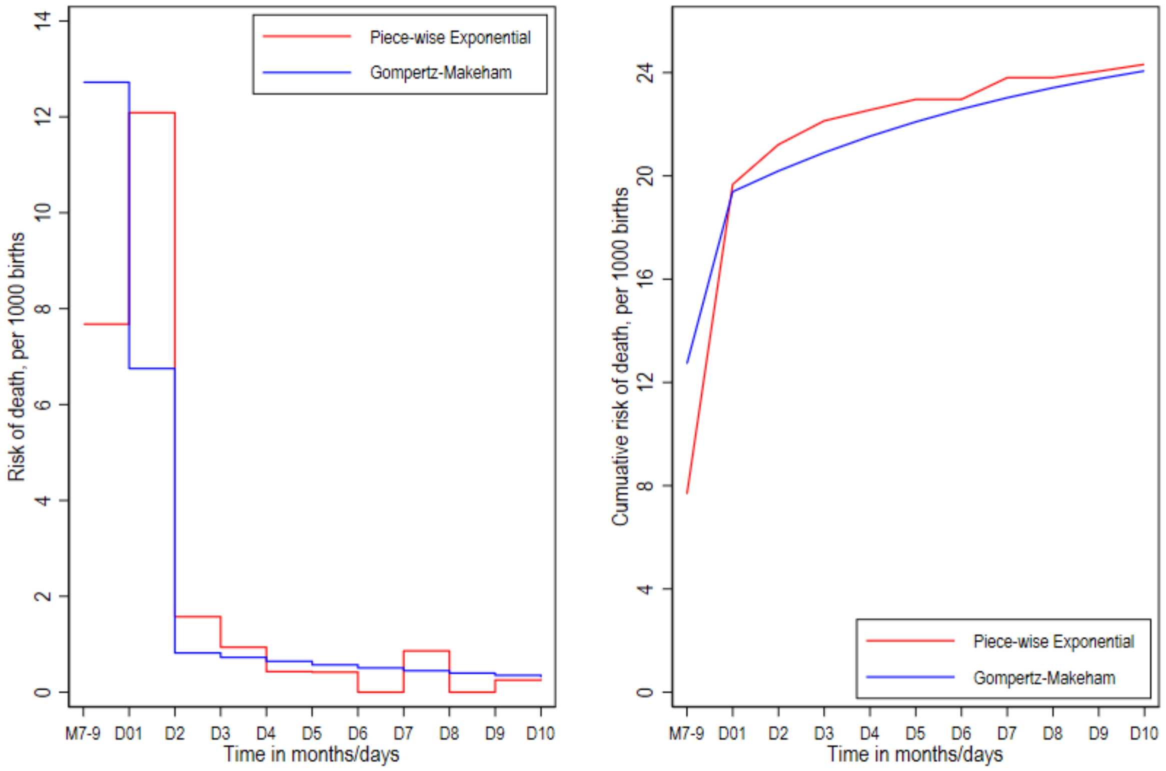

Figure S2: Niger DHS-2006

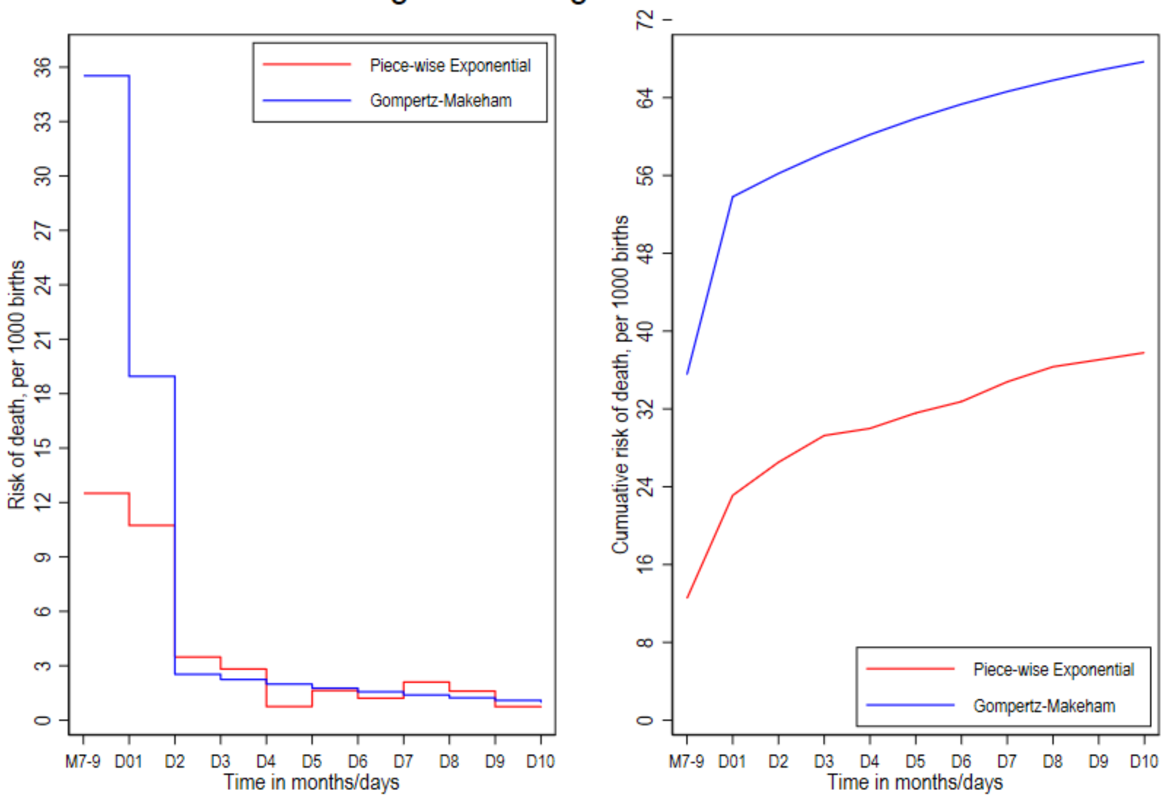

Figure S2: Niger DHS-2012

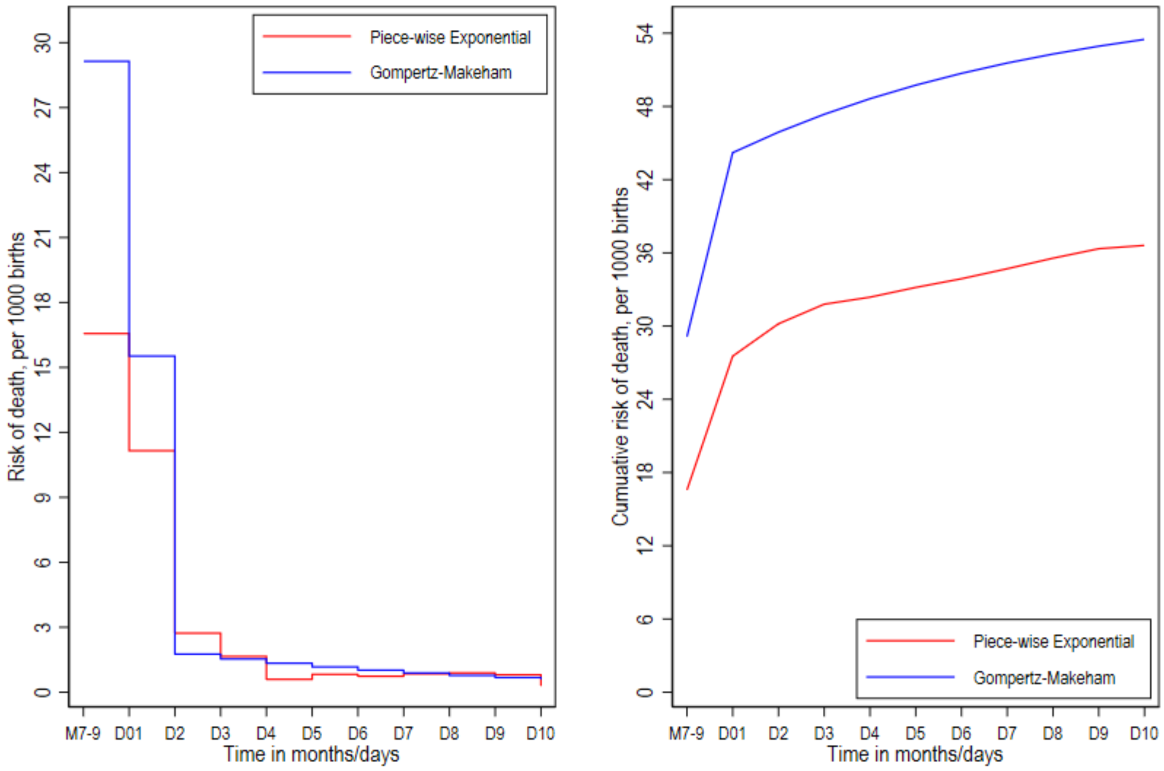

Figure S2: Nigeria DHS-2008

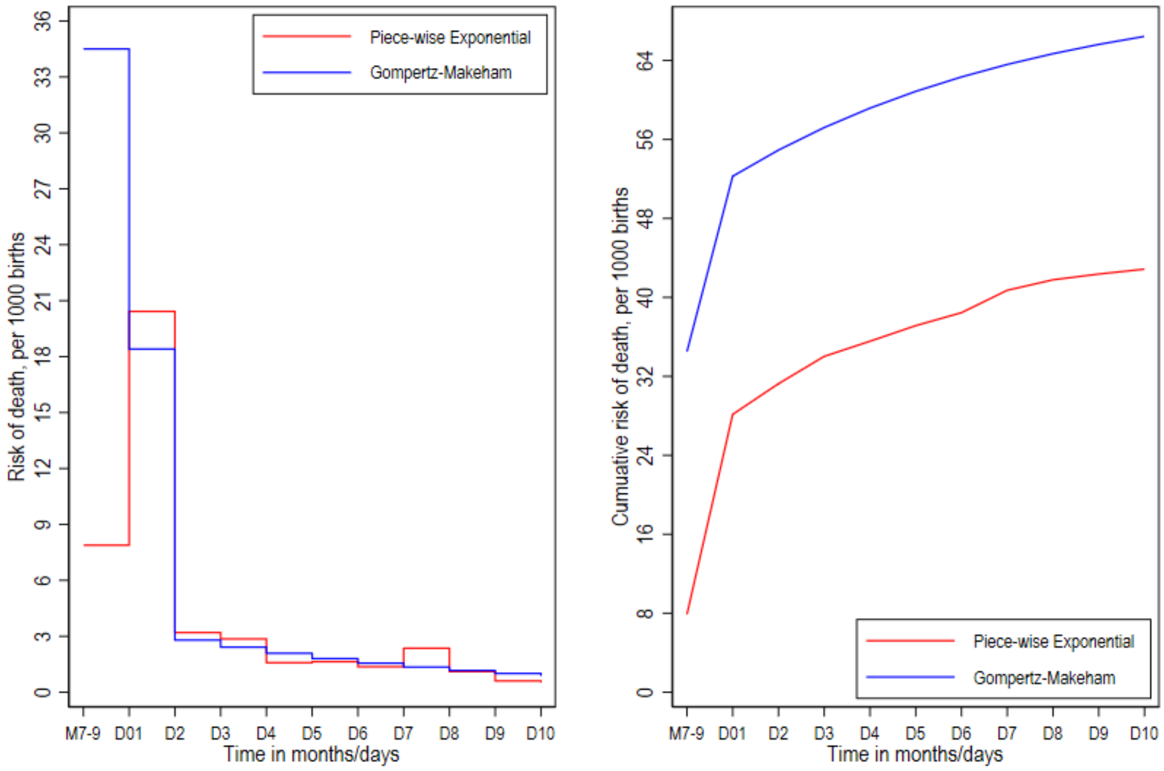

Figure S2: Nigeria DHS-2013

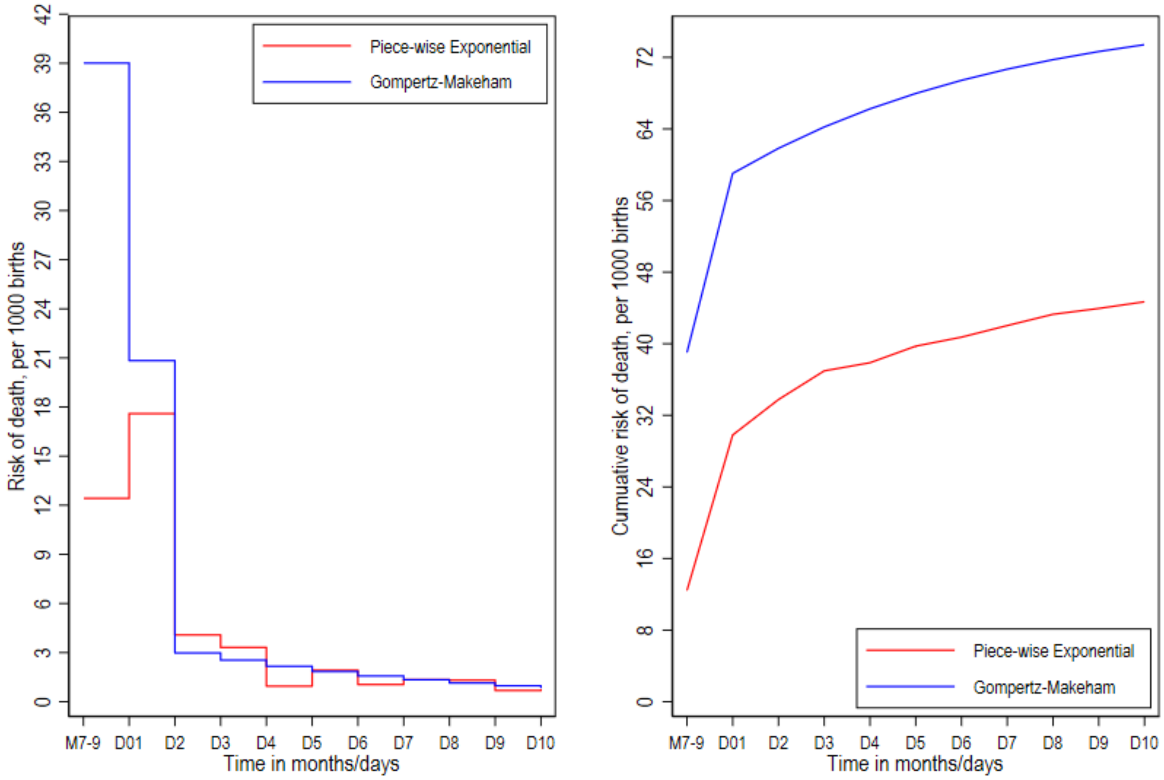

Figure S2: Nigeria DHS-2018

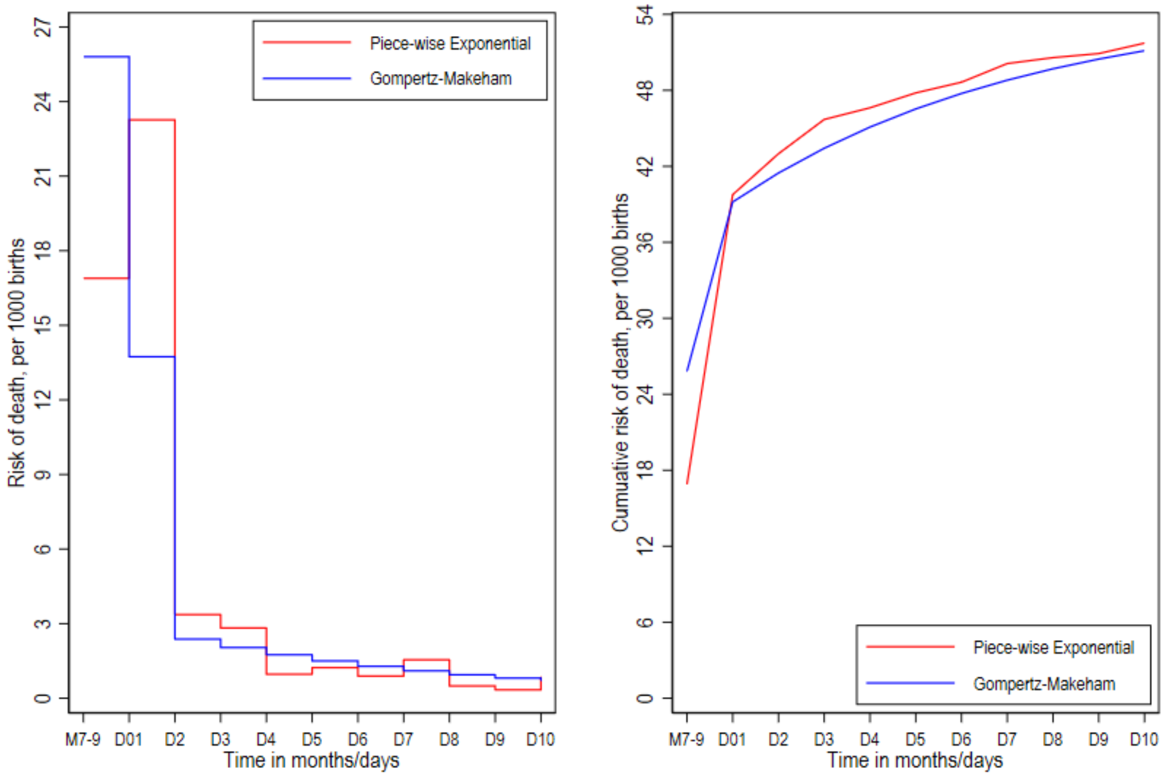

Figure S2: Rwanda DHS-2000

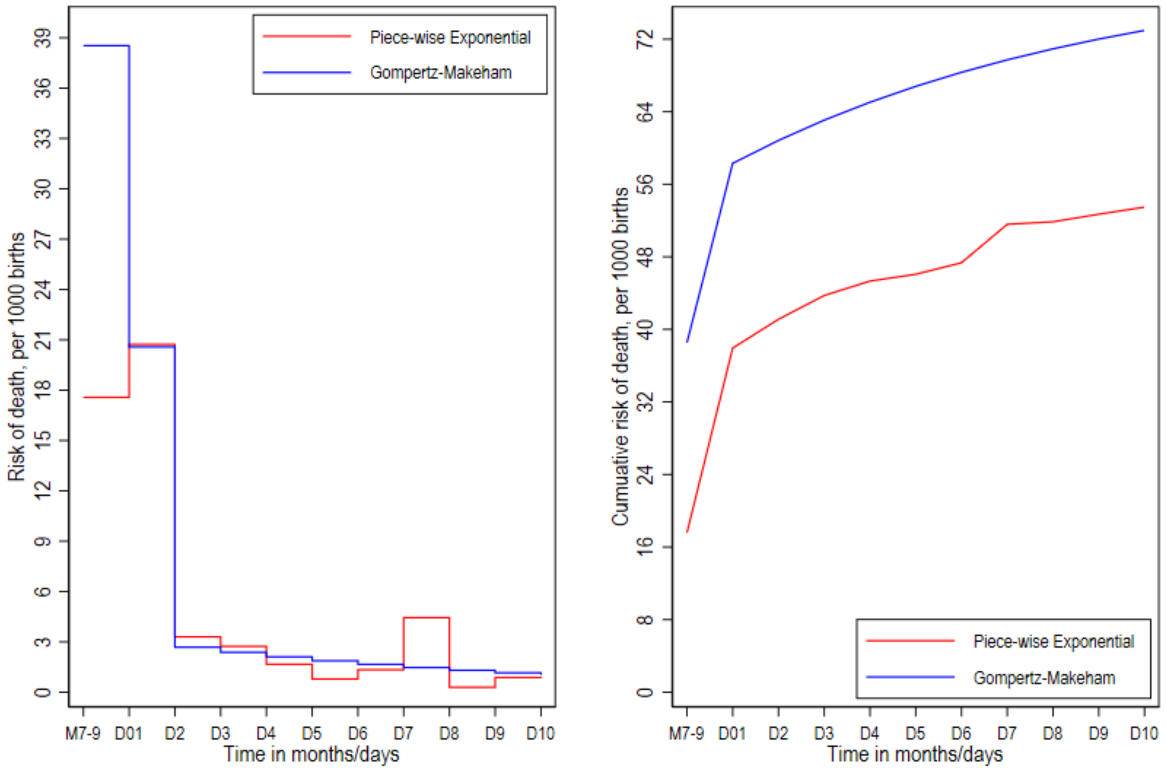

Figure S2: Rwanda DHS-2005

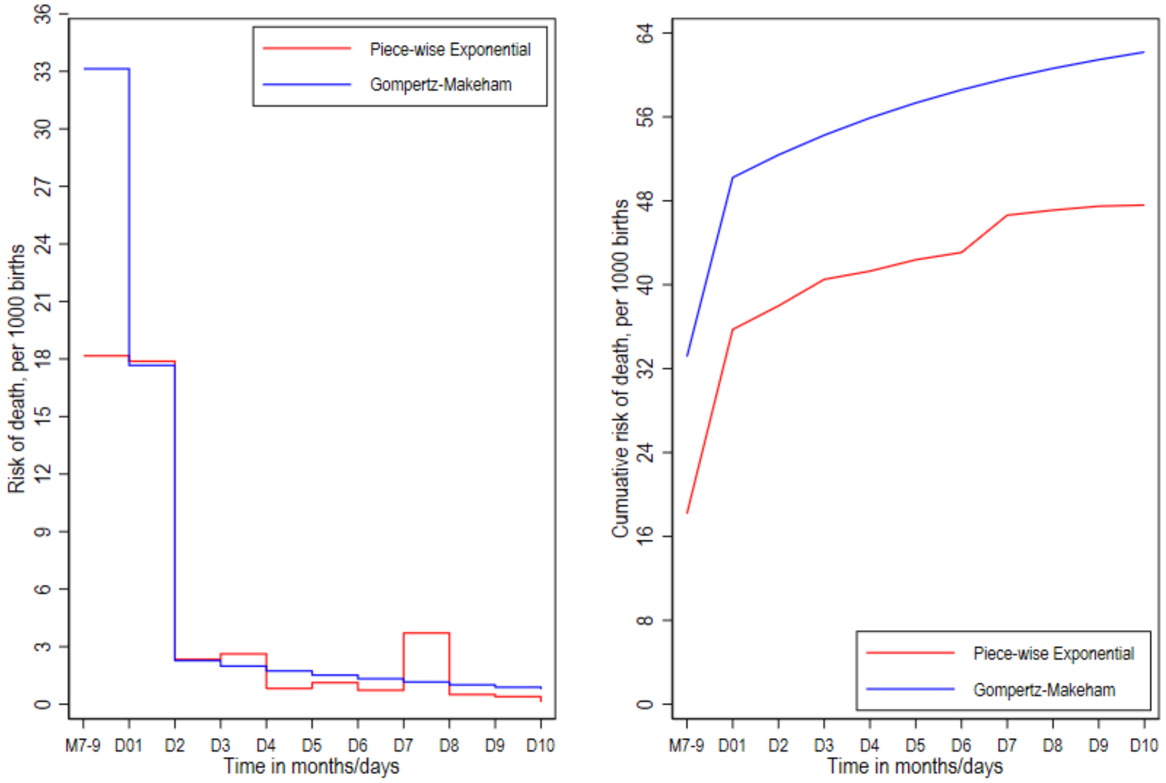

Figure S2: Rwanda DHS-2010/11

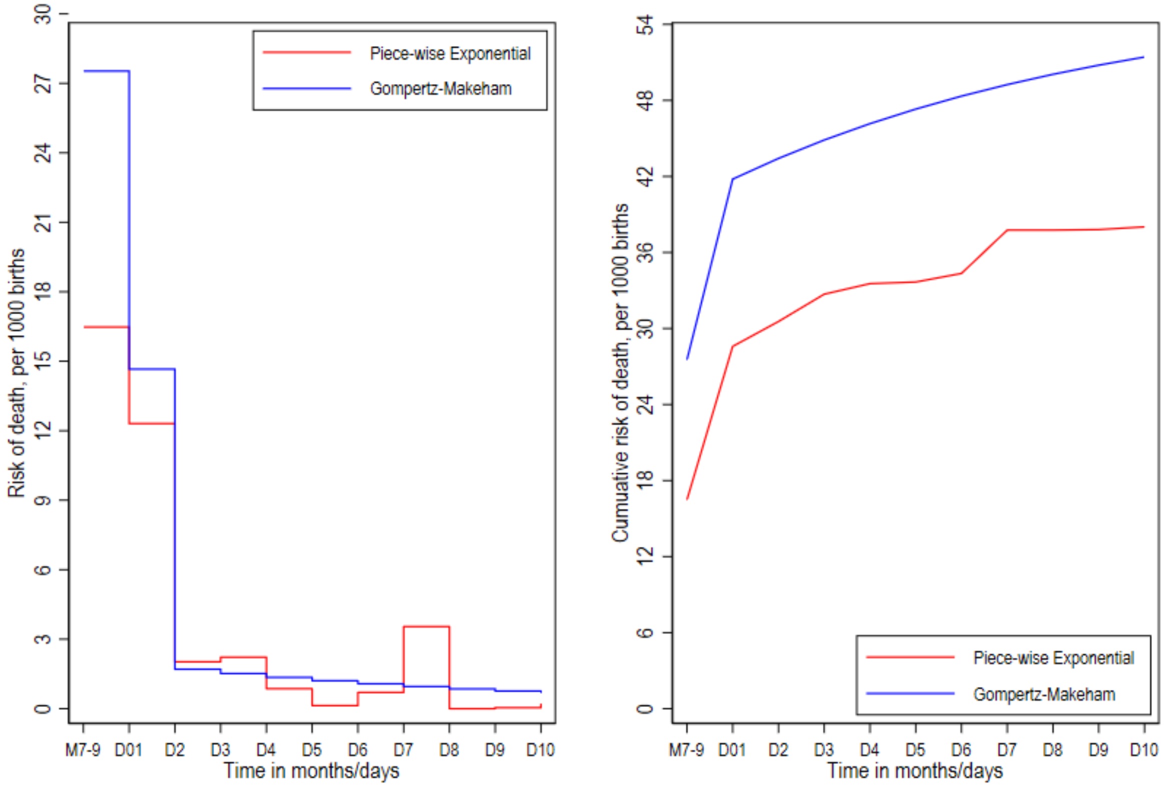

Figure S2: Rwanda DHS-2014/15

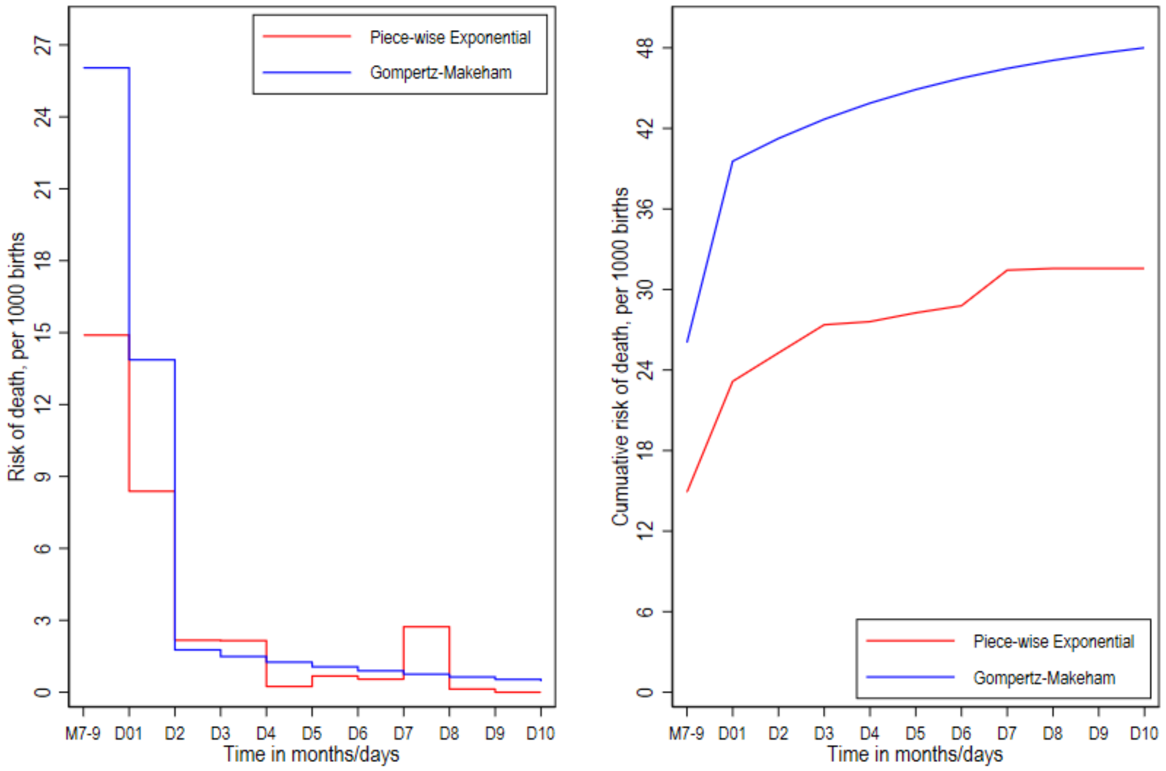

Figure S2: Rwanda DHS-2019/20

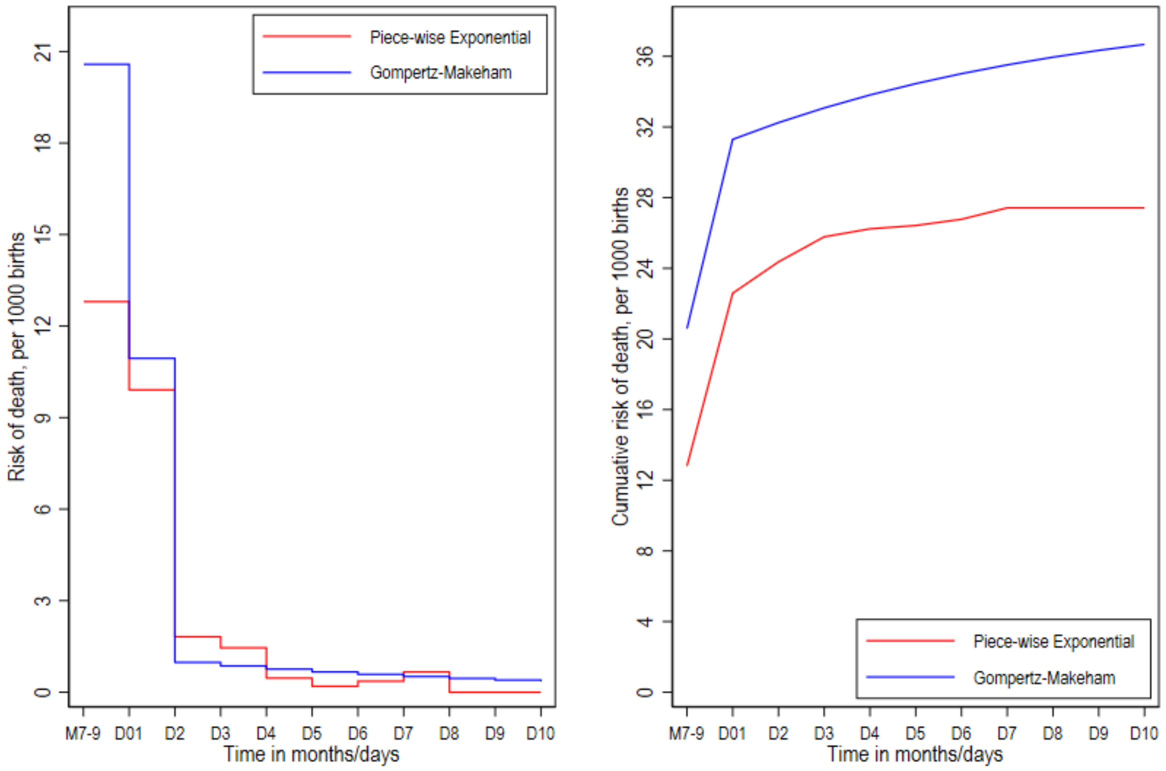

Figure S2: Senegal DHS-2005

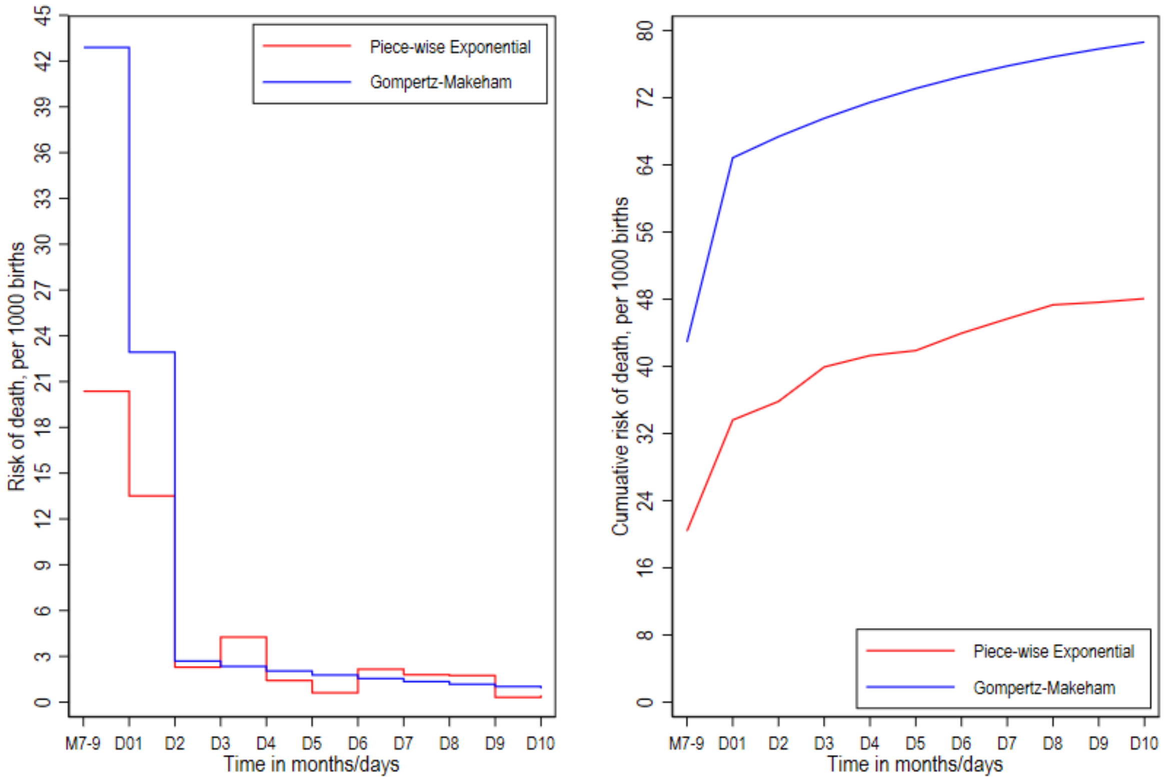

Figure S2: Senegal DHS-2010/11

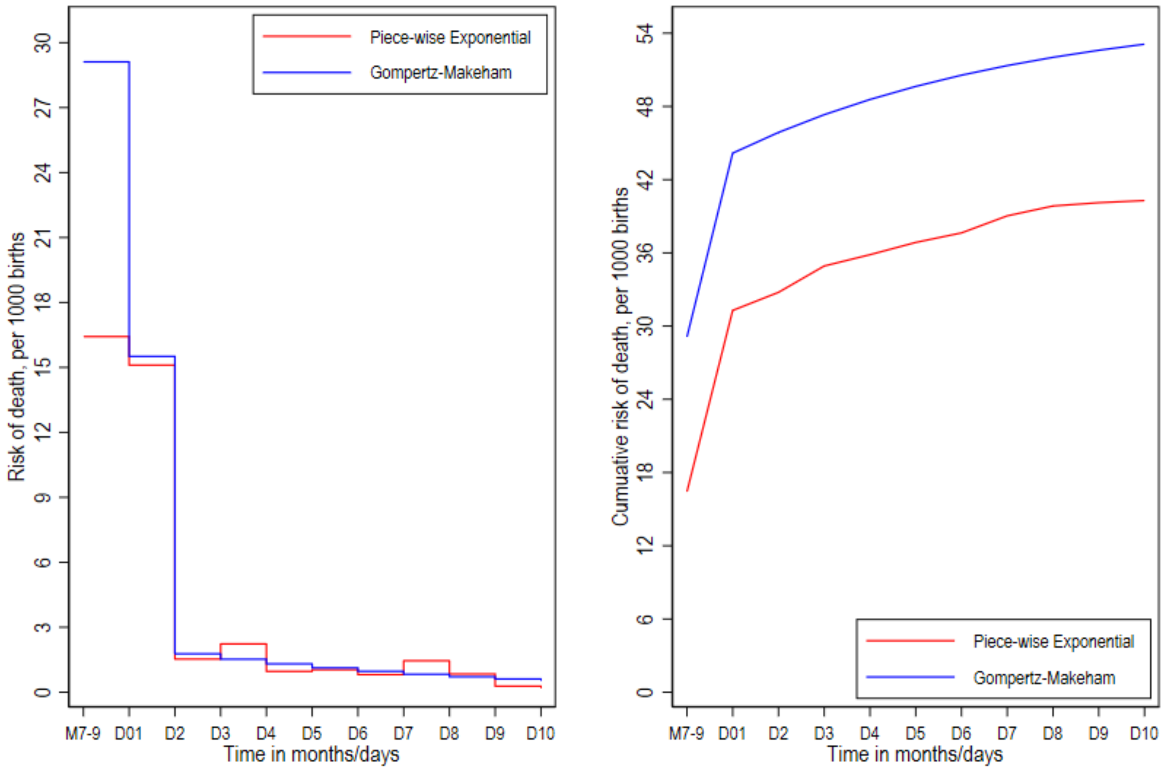

Figure S2: Senegal DHS-2015

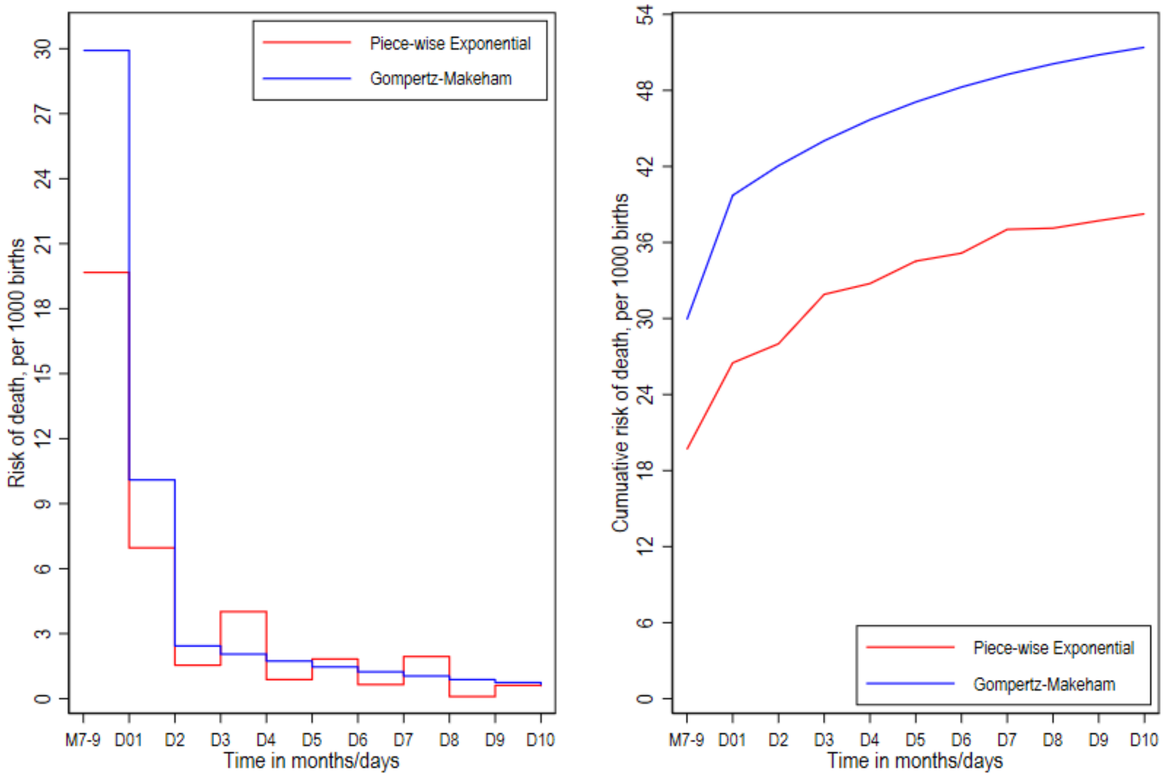

Figure S2: Senegal DHS-2016

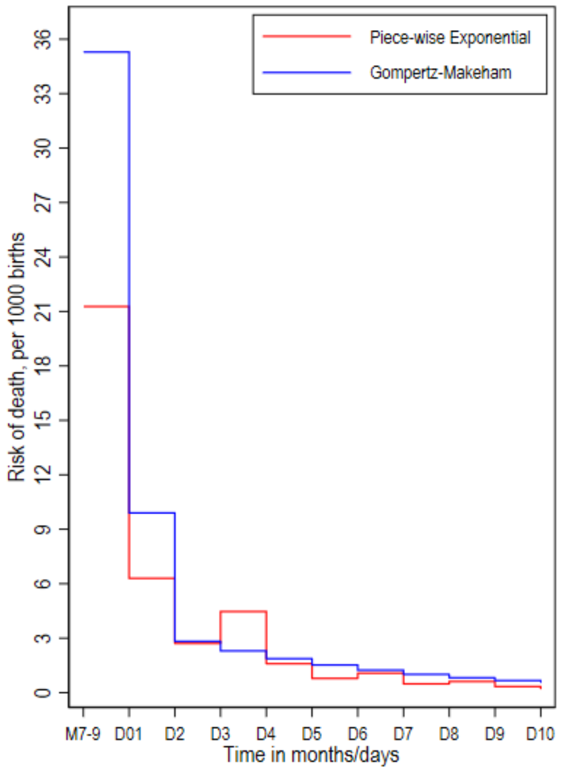

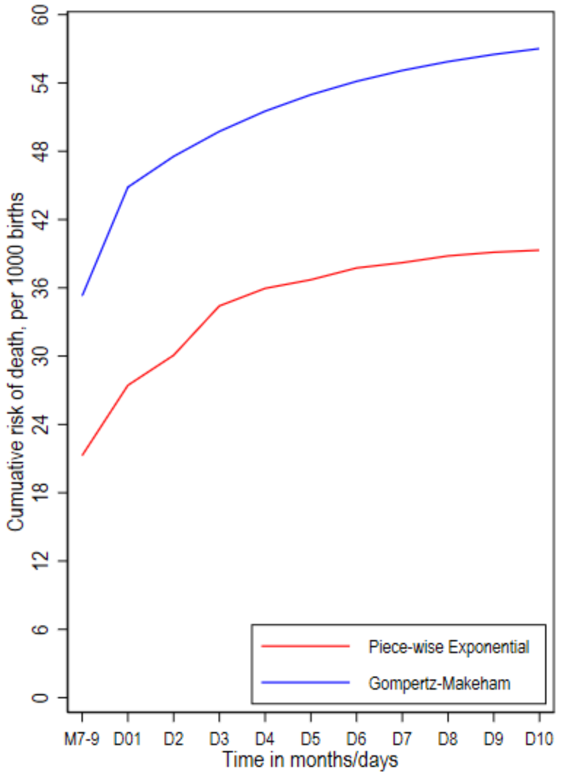

Figure S2: Senegal DHS-2018

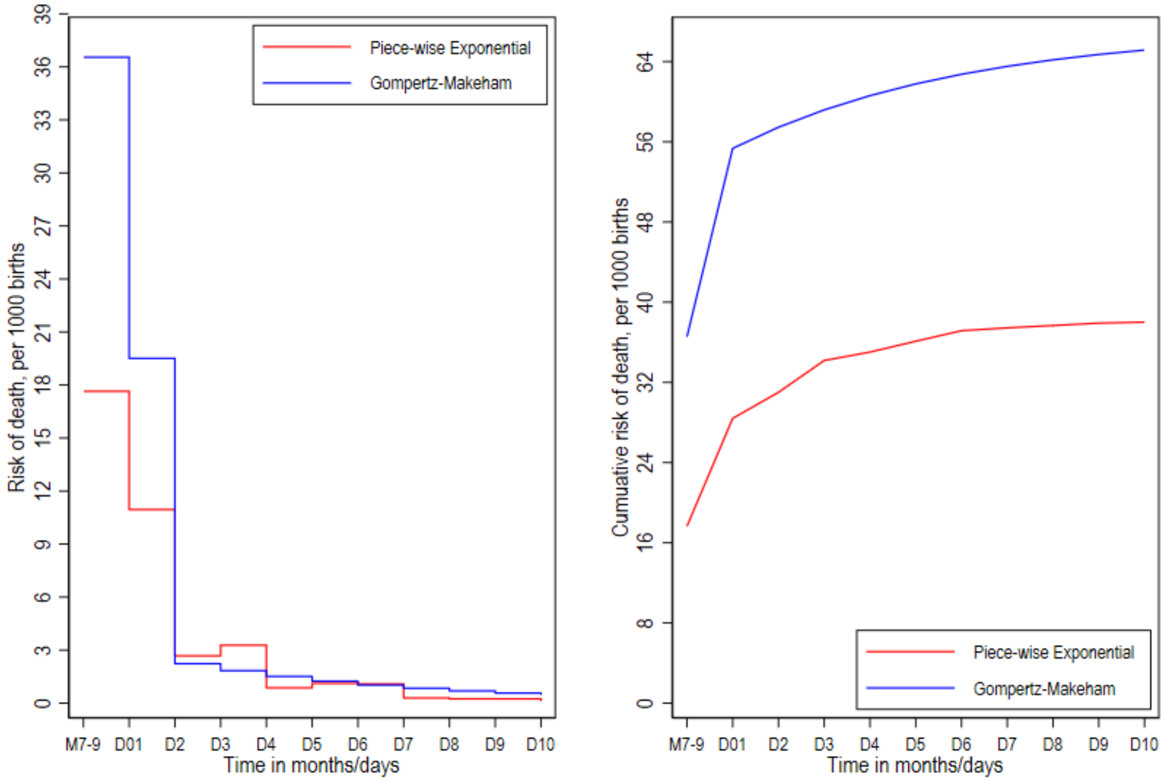

Figure S2: Senegal DHS-2019

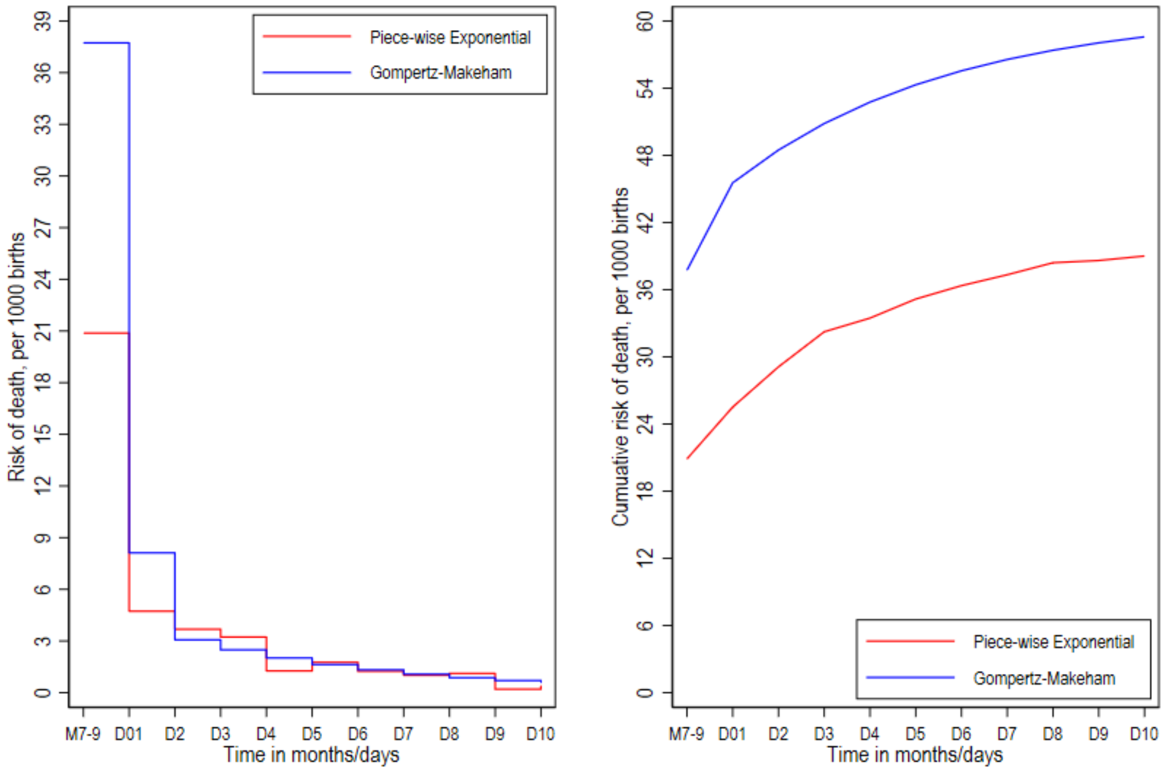

Figure S2: Sierra Leone DHS-2008

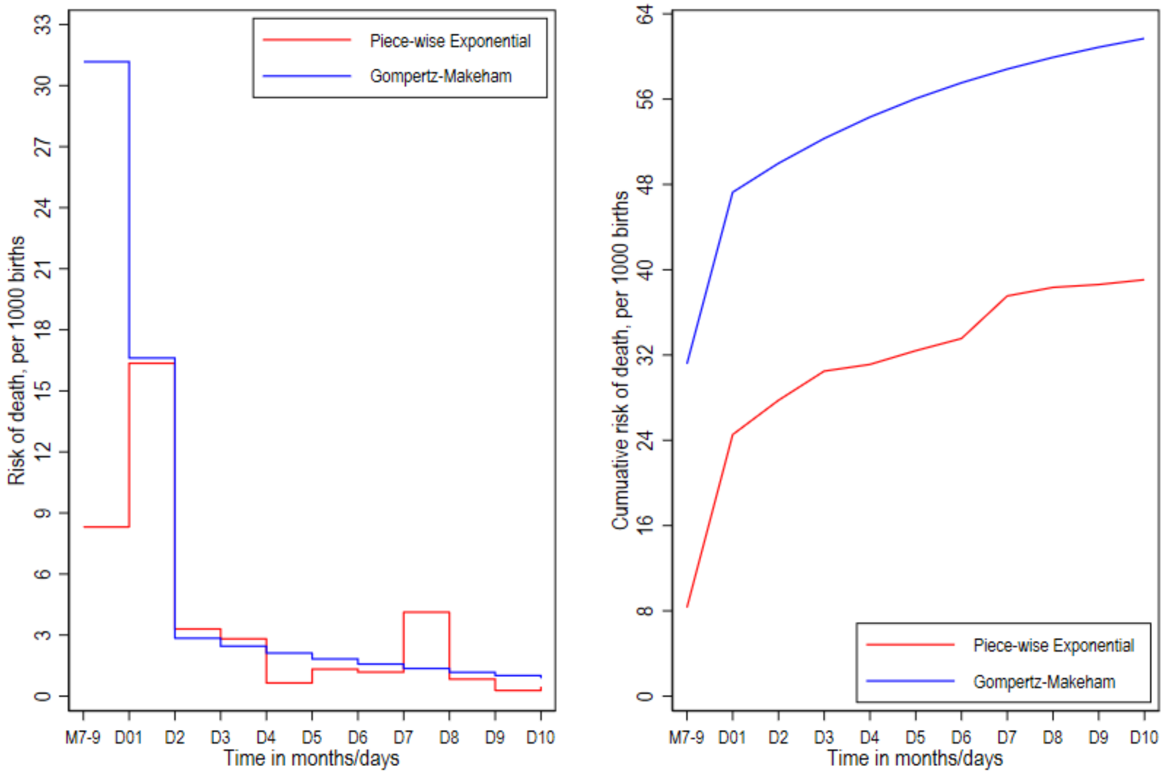

Figure S2: Sierra Leone DHS-2013

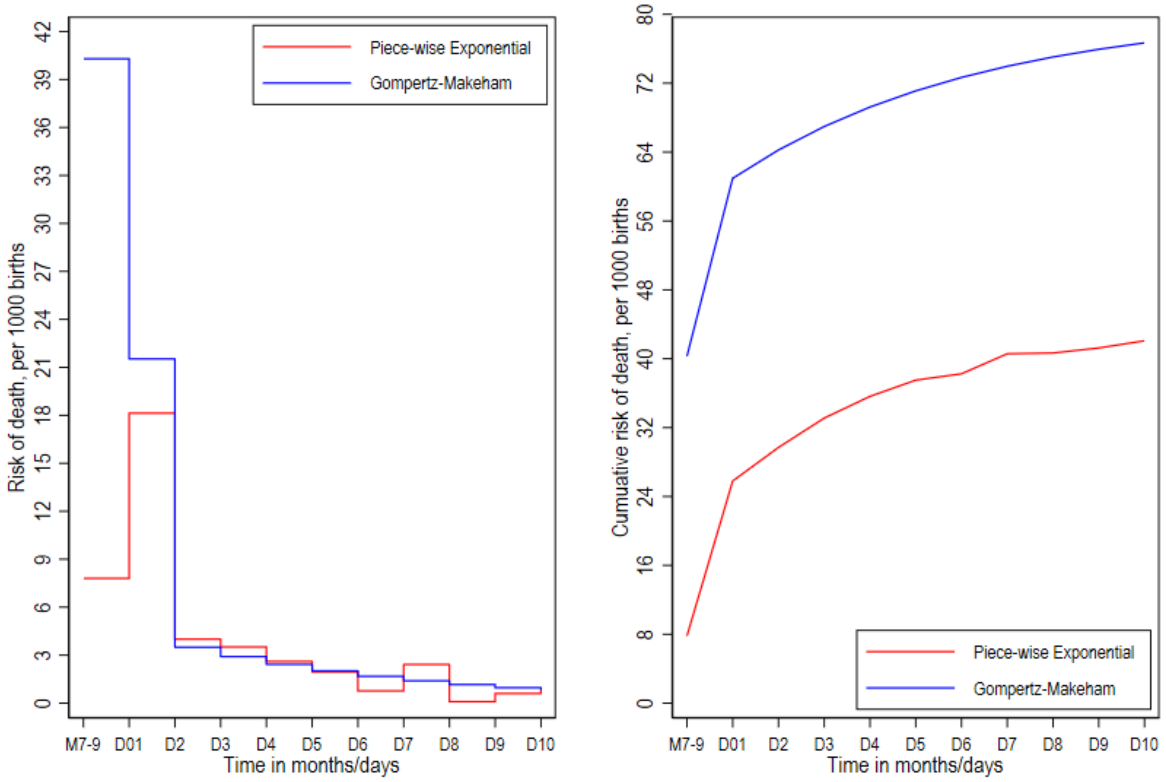

Figure S2: South Africa DHS-2016

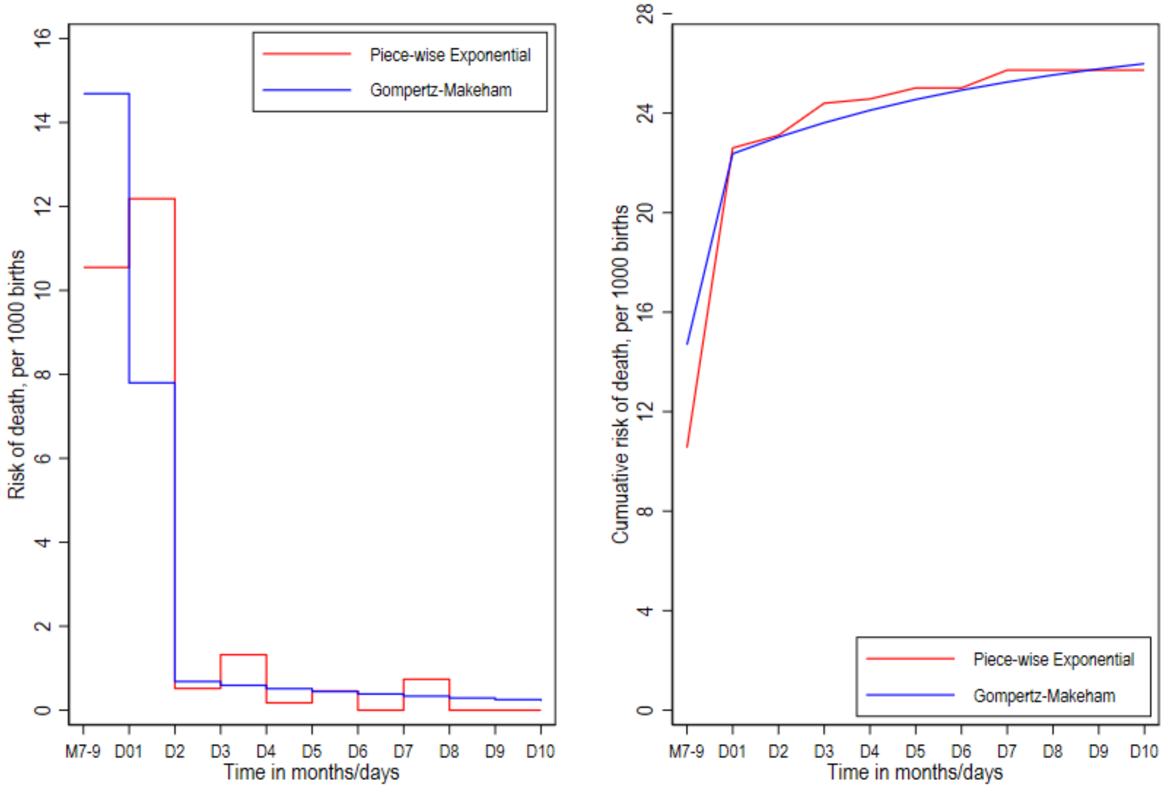

Figure S2: Eswatini DHS-2006/7

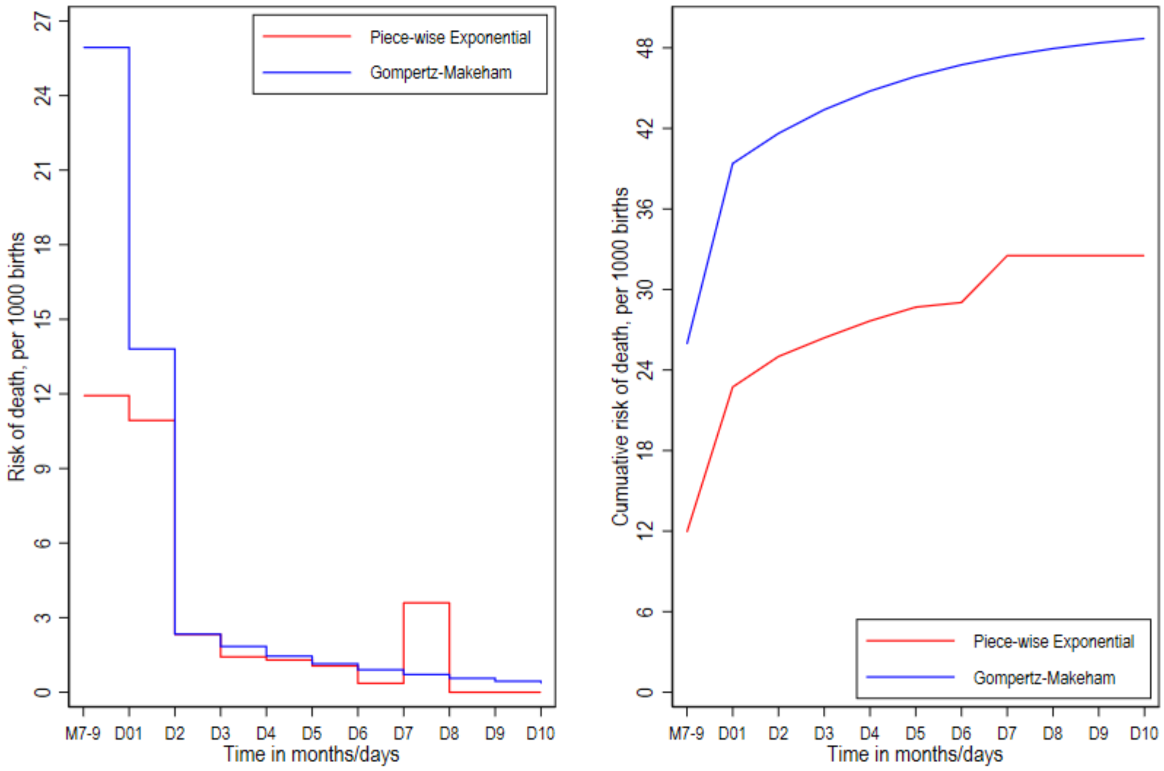

Figure S2: Tanzania DHS-2004/5

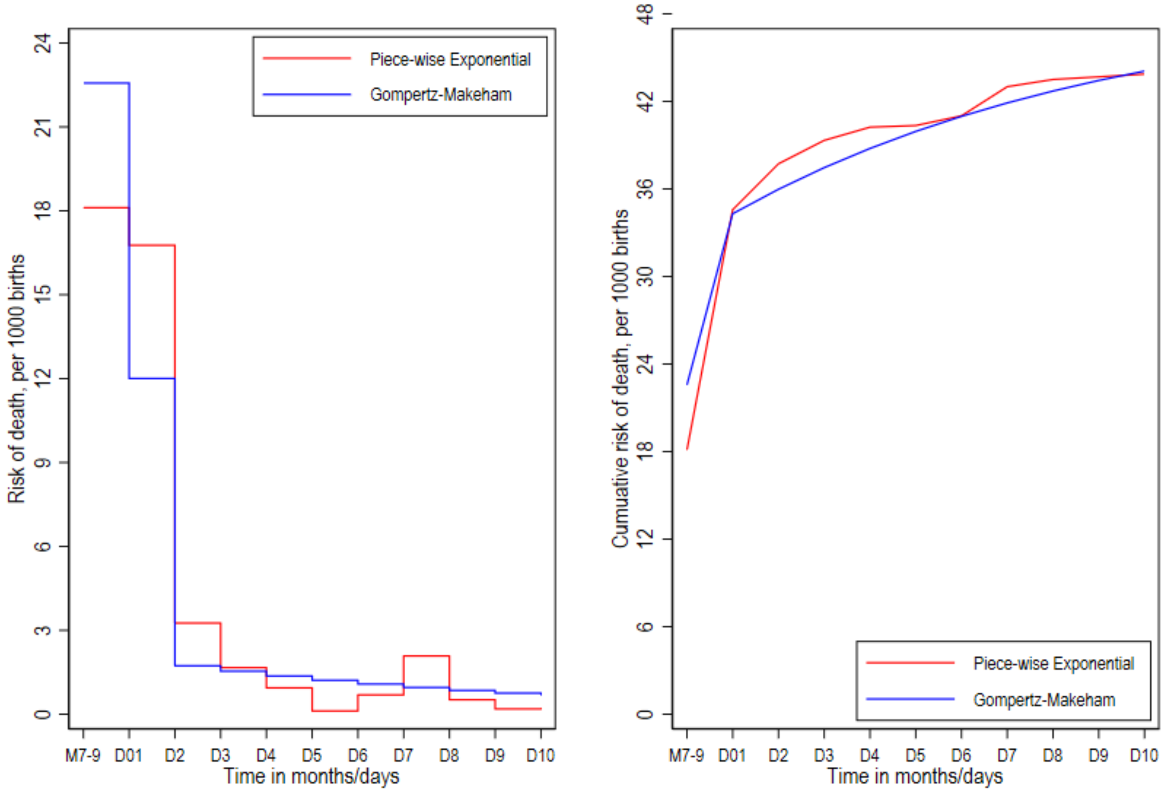

Figure S2: Tanzania DHS-2009/10

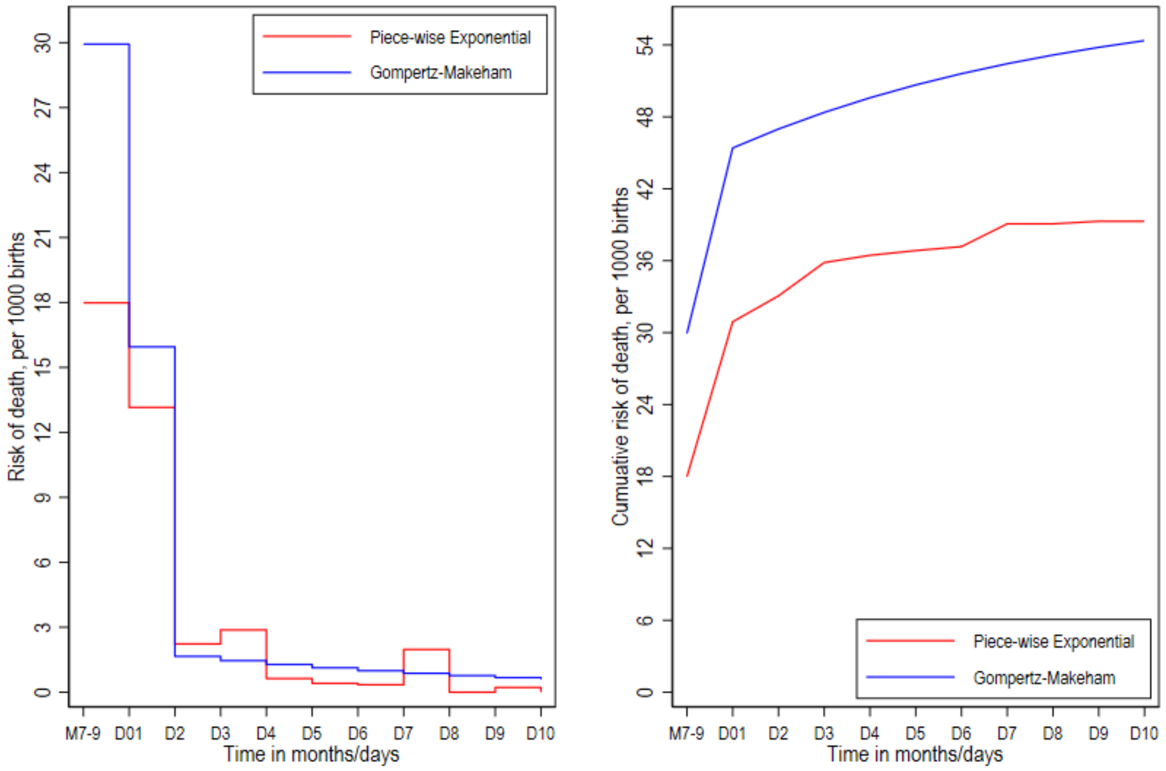

Figure S2: Tanzania DHS-2015/16

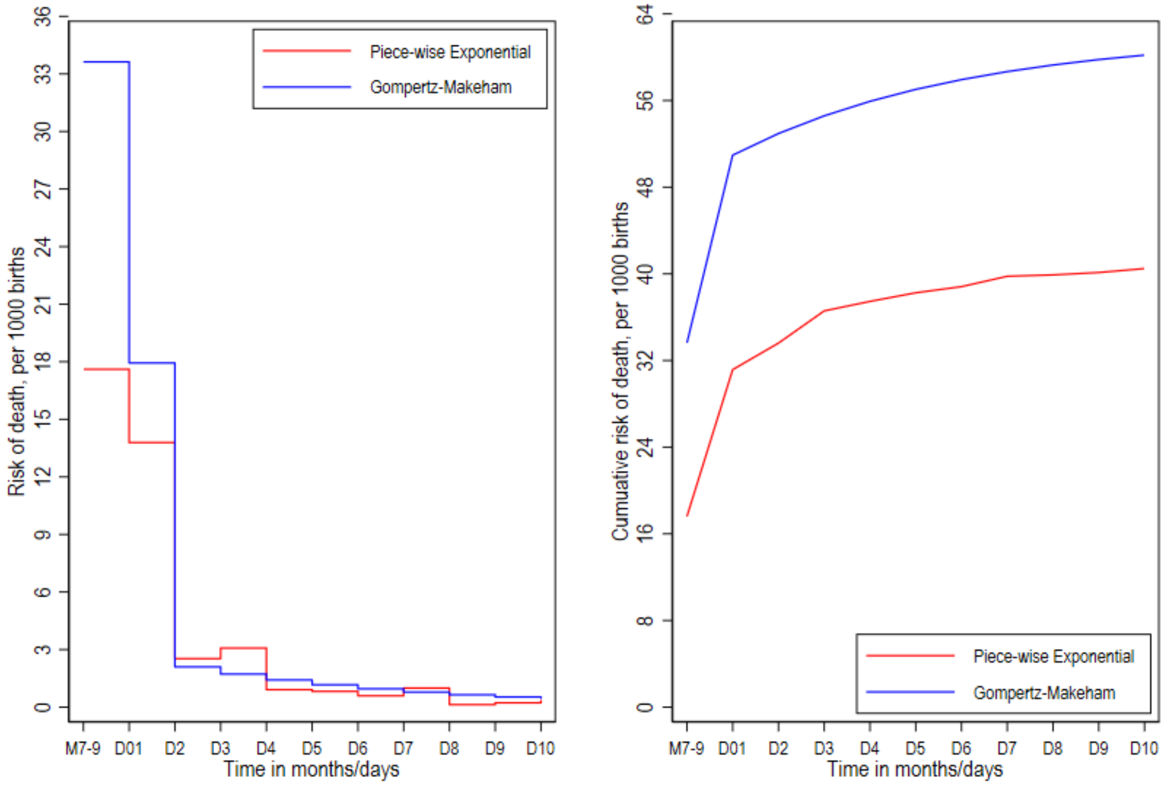

Figure S2: Uganda DHS-2000/1

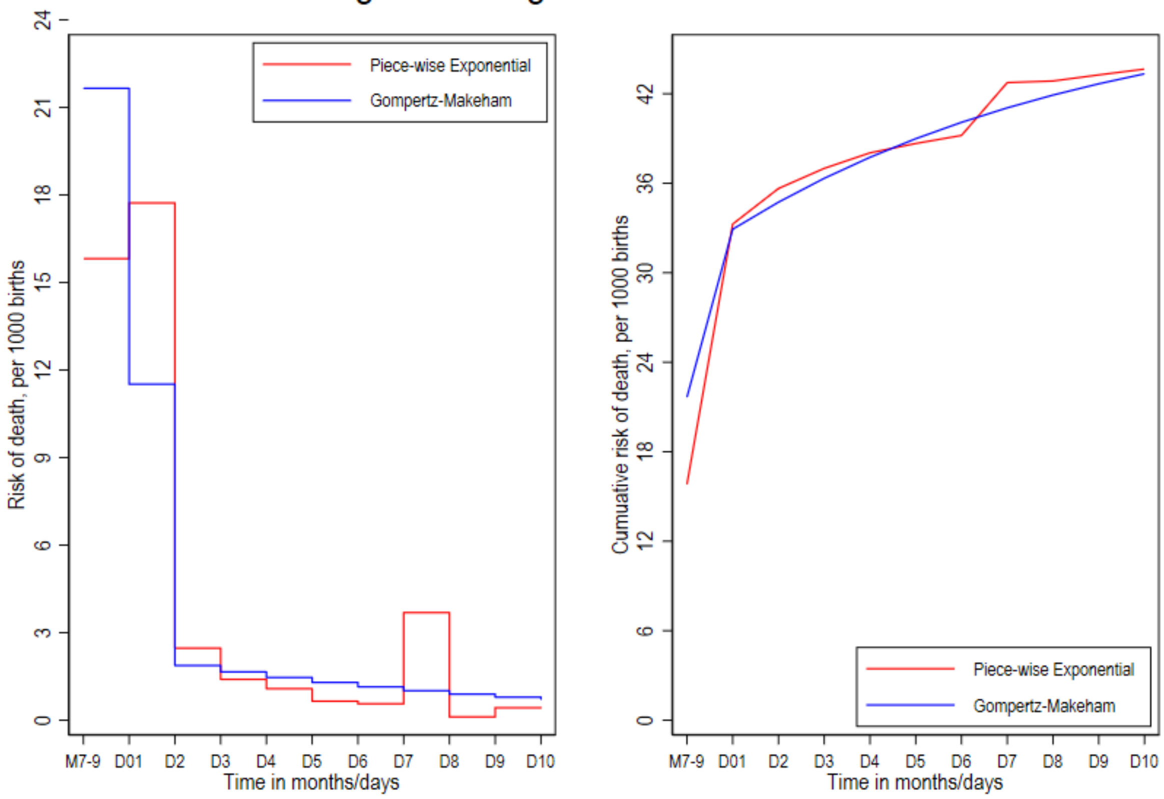

Figure S2: Uganda DHS-2006

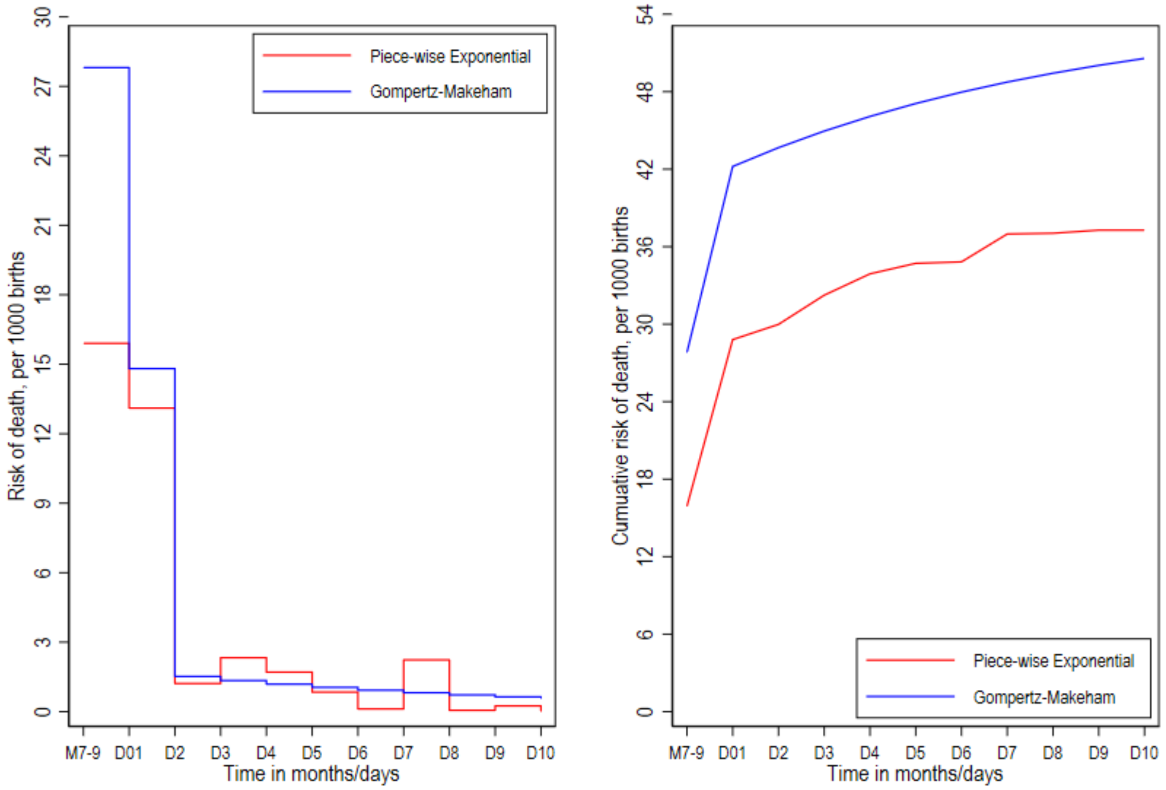

Figure S2: Uganda DHS-2011

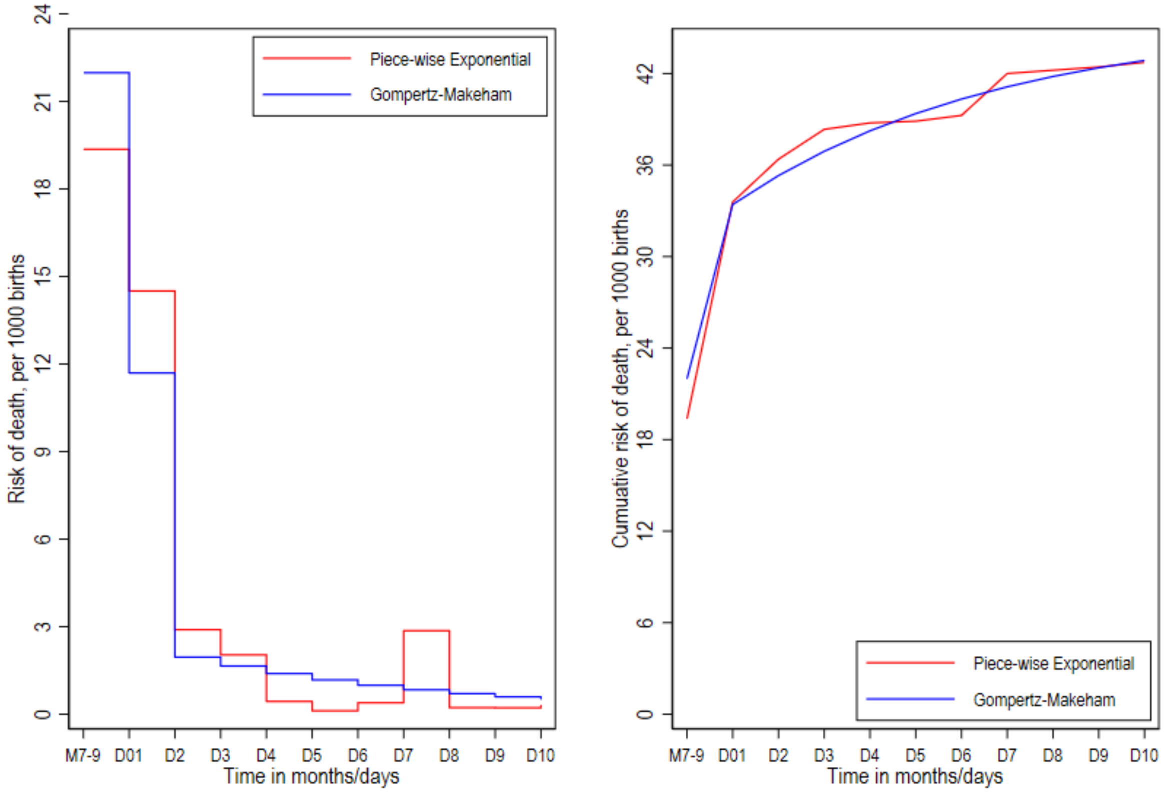

Figure S2: Zambia DHS-2007

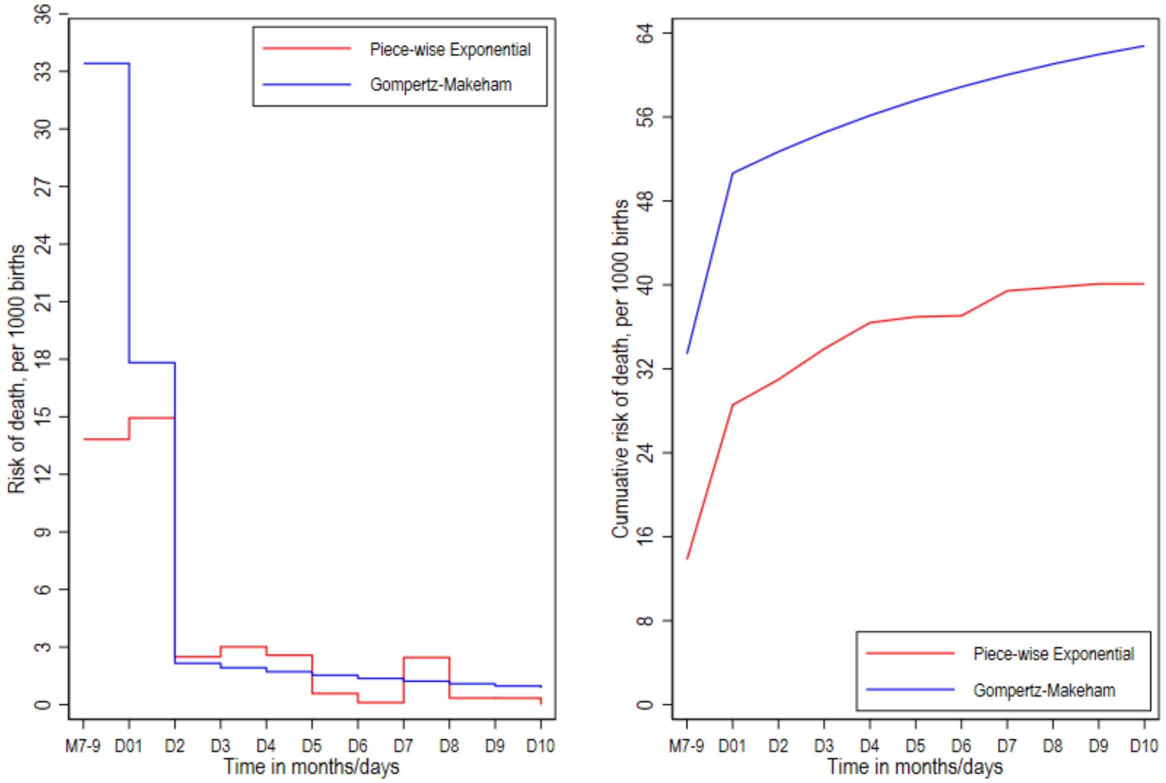

Figure S2: Zambia DHS-2013/14

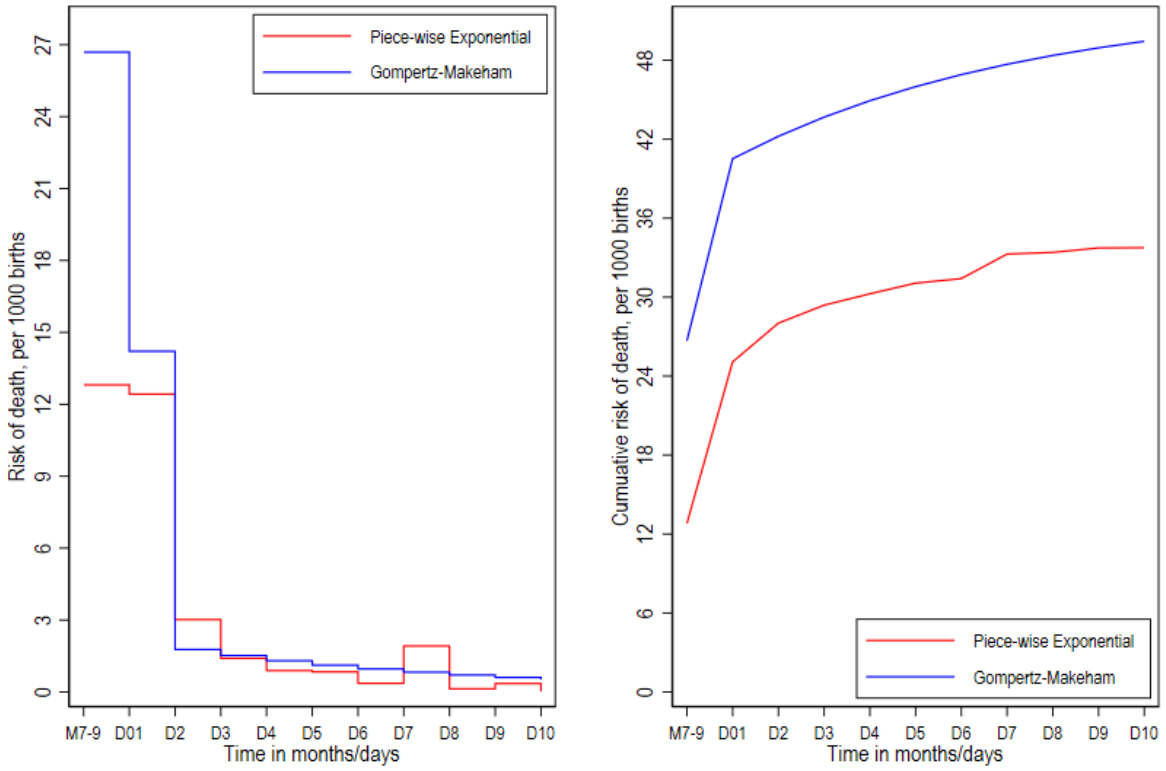

Figure S2: Zambia DHS-2018/19

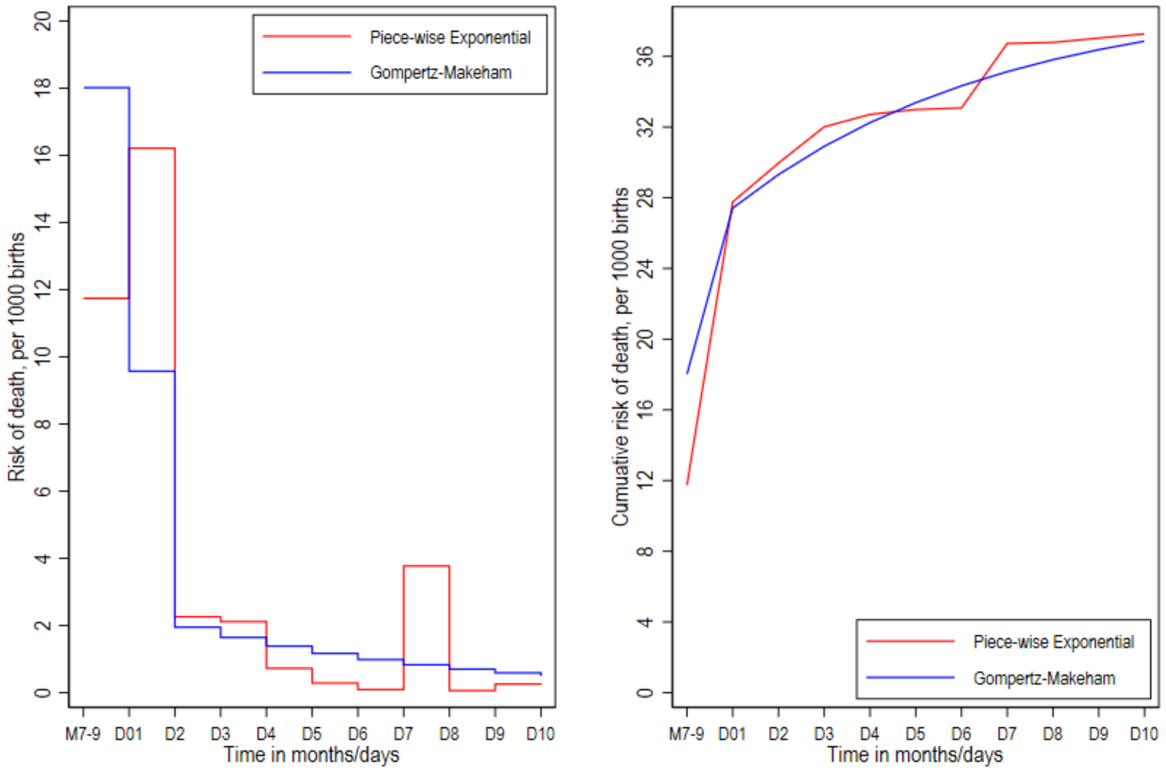

Figure S2: Zimbabwe DHS-1994

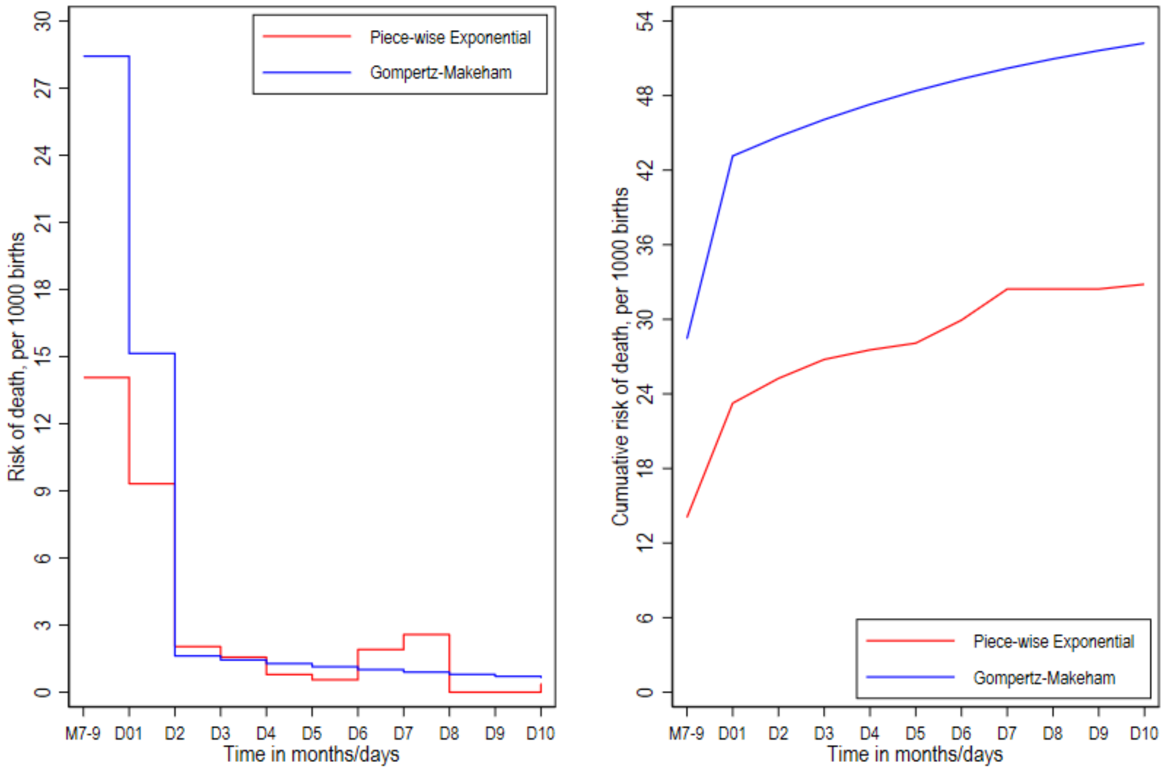

Figure S2: Zimbabwe DHS-1999

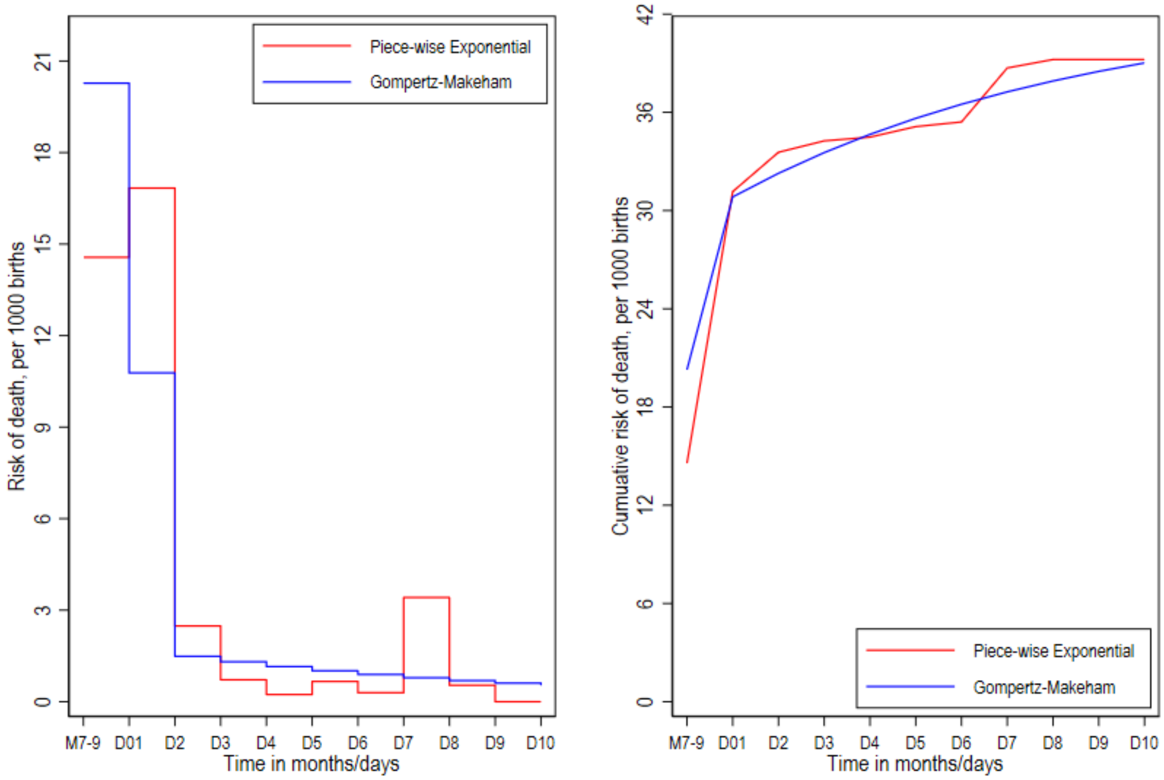

Figure S2: Zimbabwe DHS-2005/6

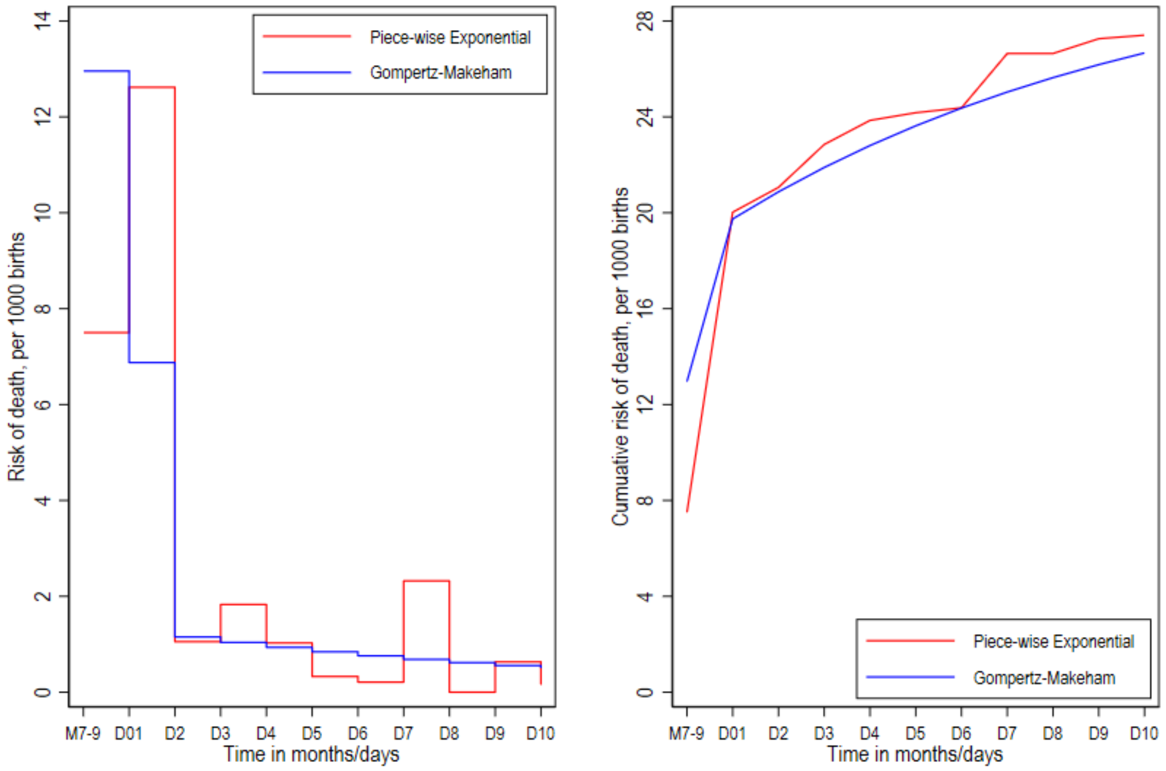

Figure S2: Zimbabwe DHS-2010/11

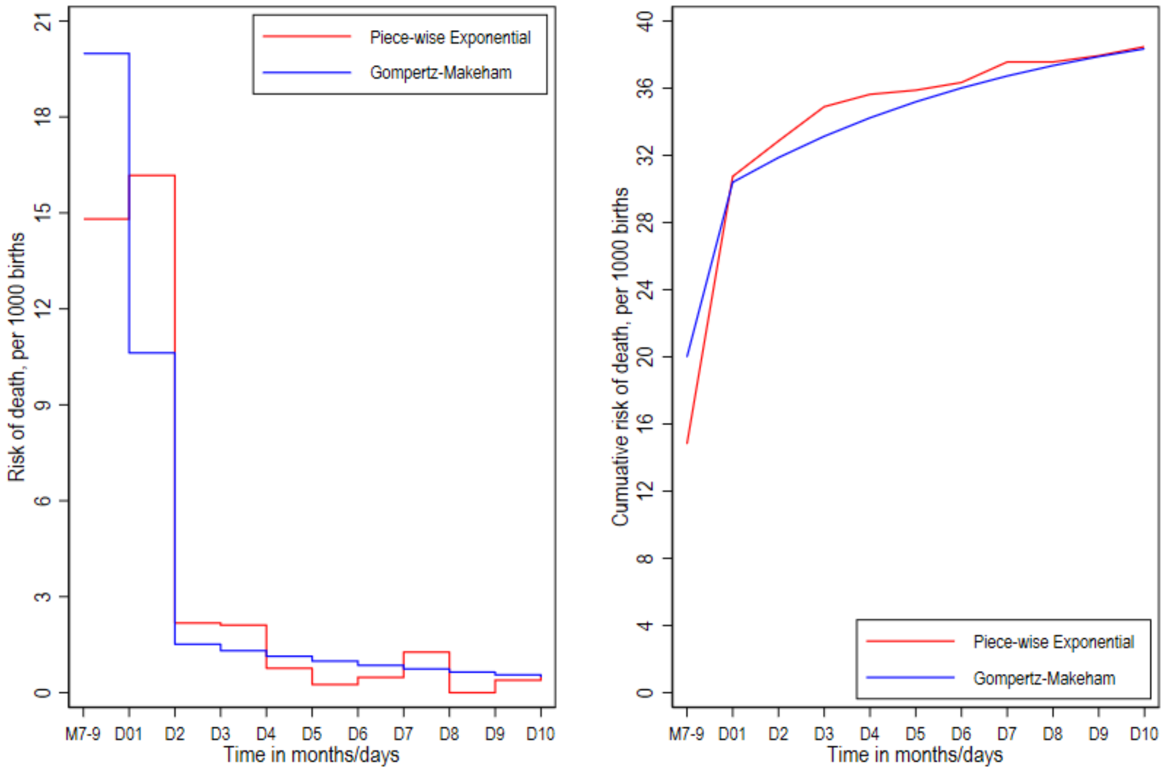

Figure S2: Zimbabwe DHS-2015

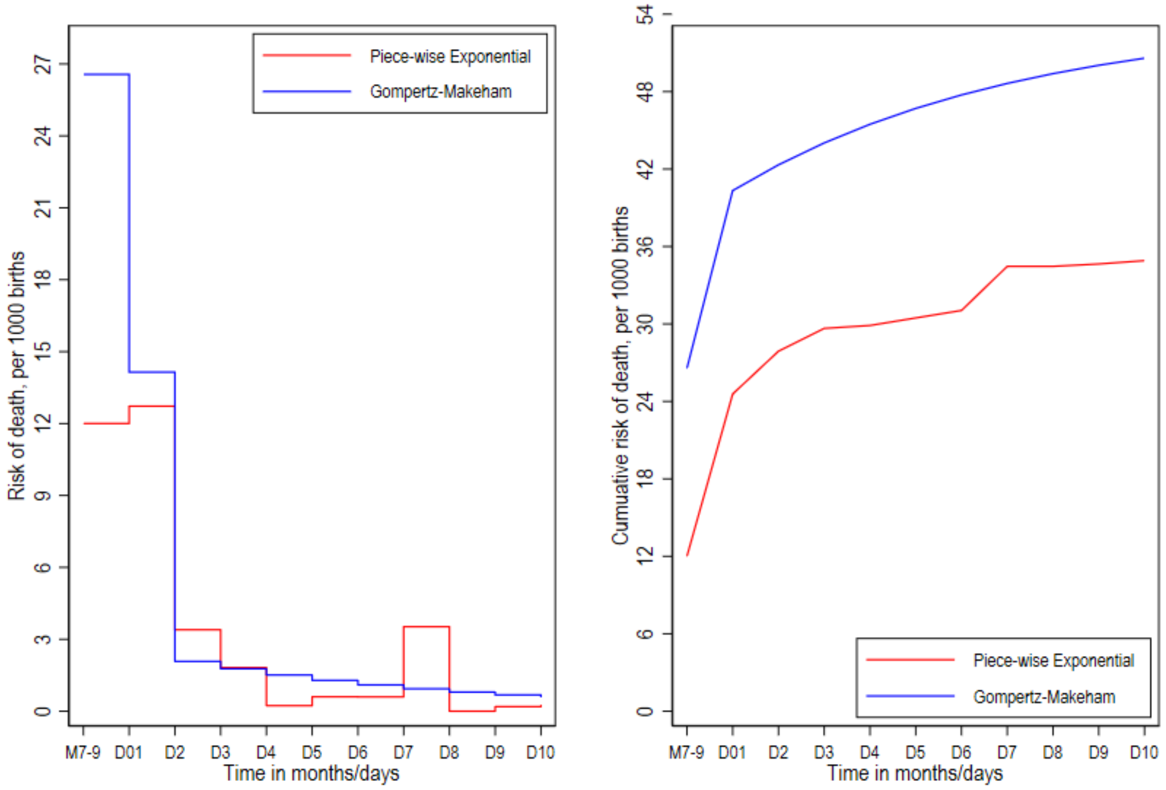

Figure S2: Azerbaijan DHS-2006

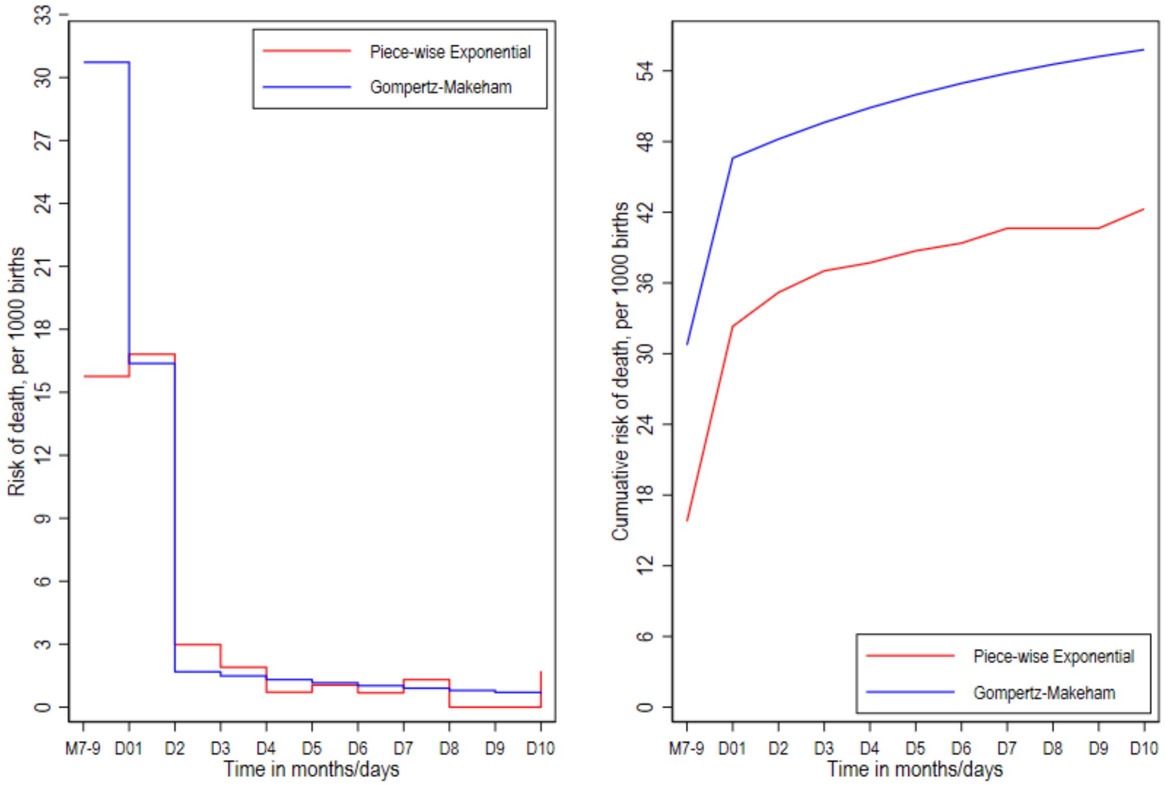

Figure S2: Egypt DHS-1992/93

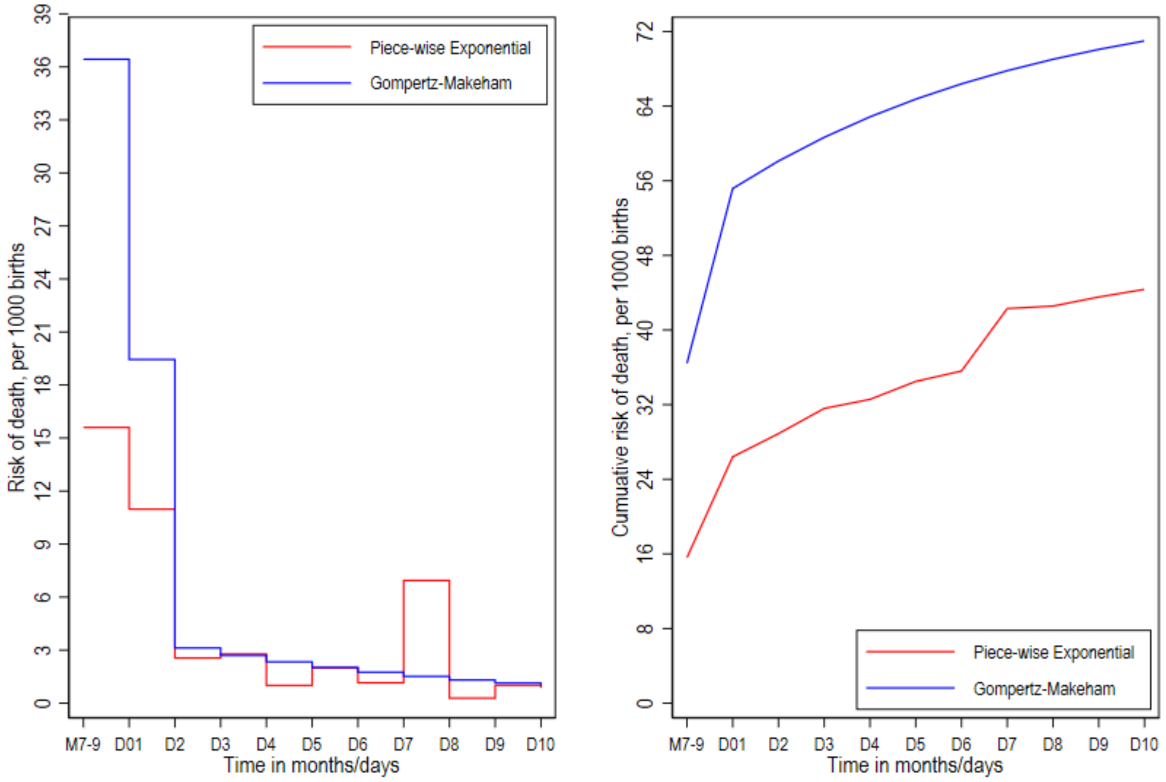

Figure S2: Egypt DHS-1995/96

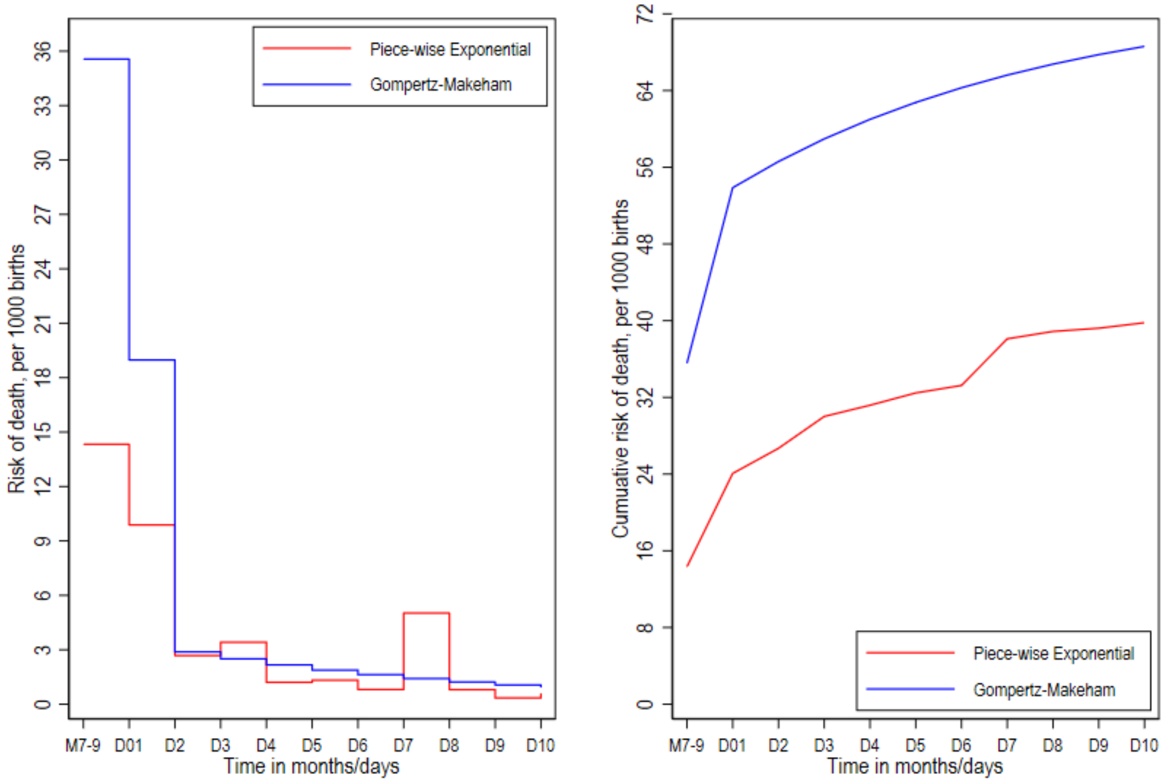

Figure S2: Egypt DHS-2000

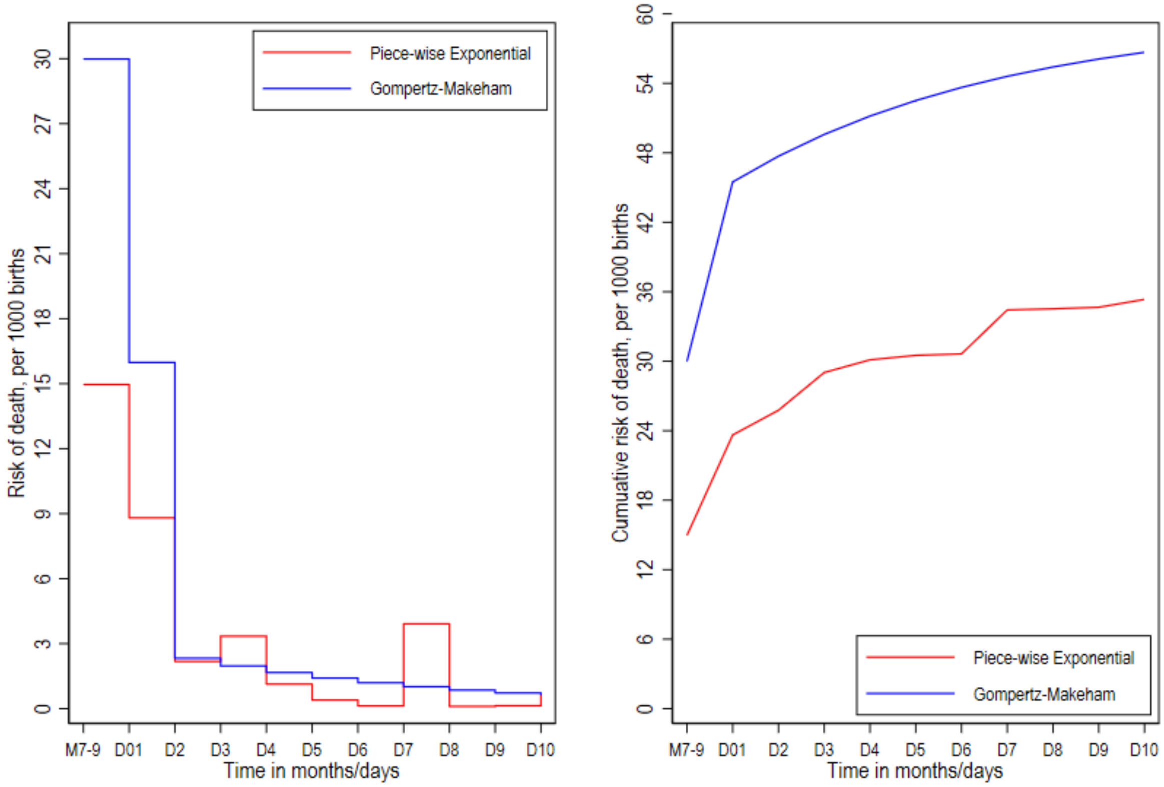

Figure S2: Egypt DHS-2003

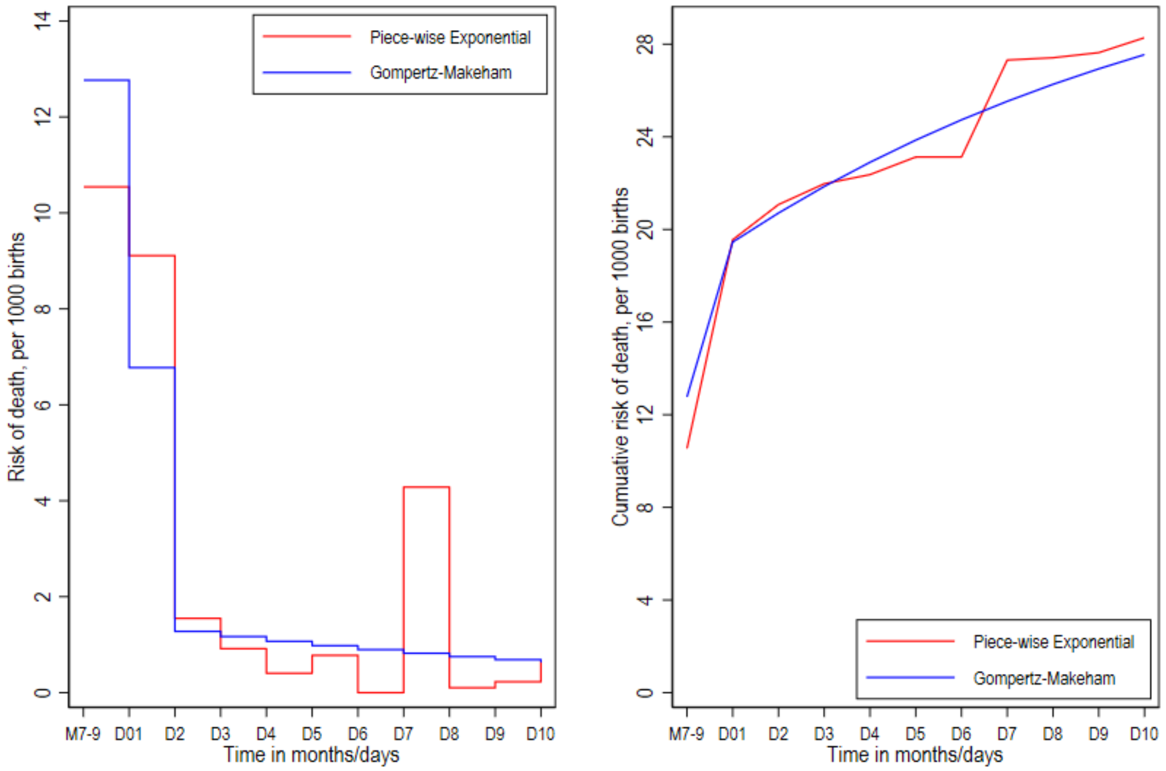

Figure S2: Egypt DHS-2005

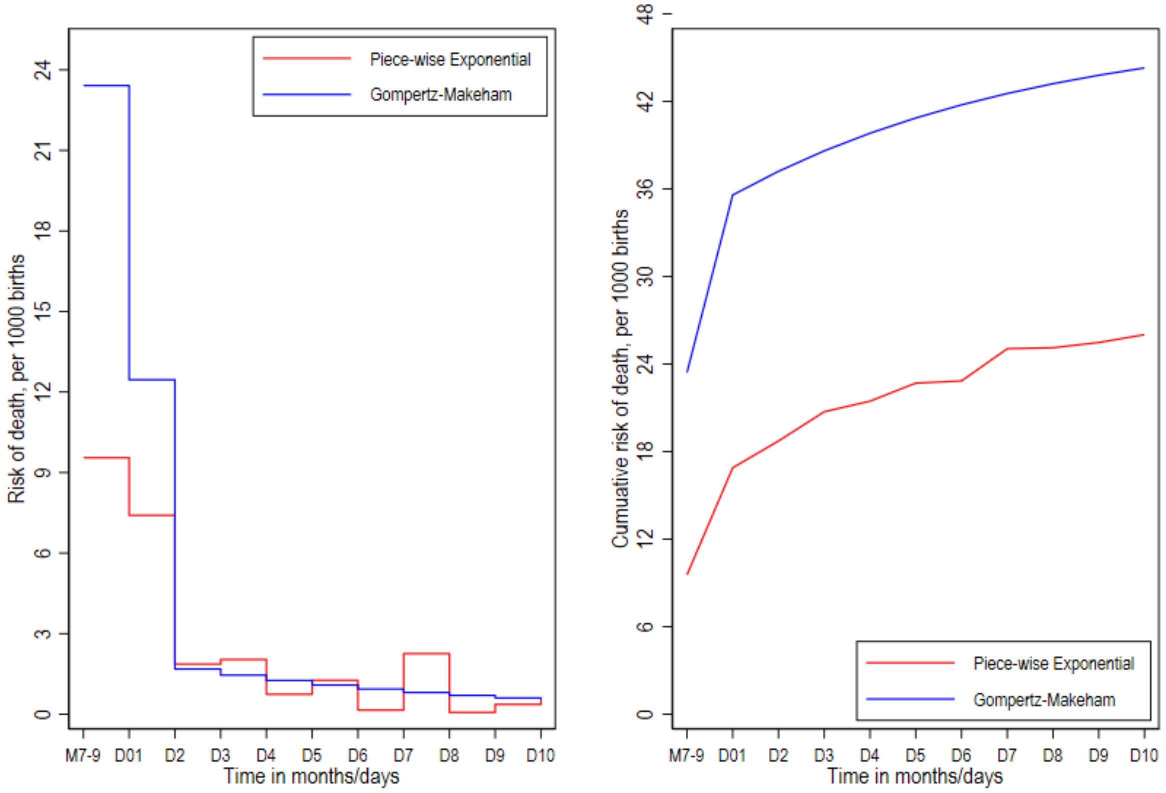

Figure S2: Egypt DHS-2008

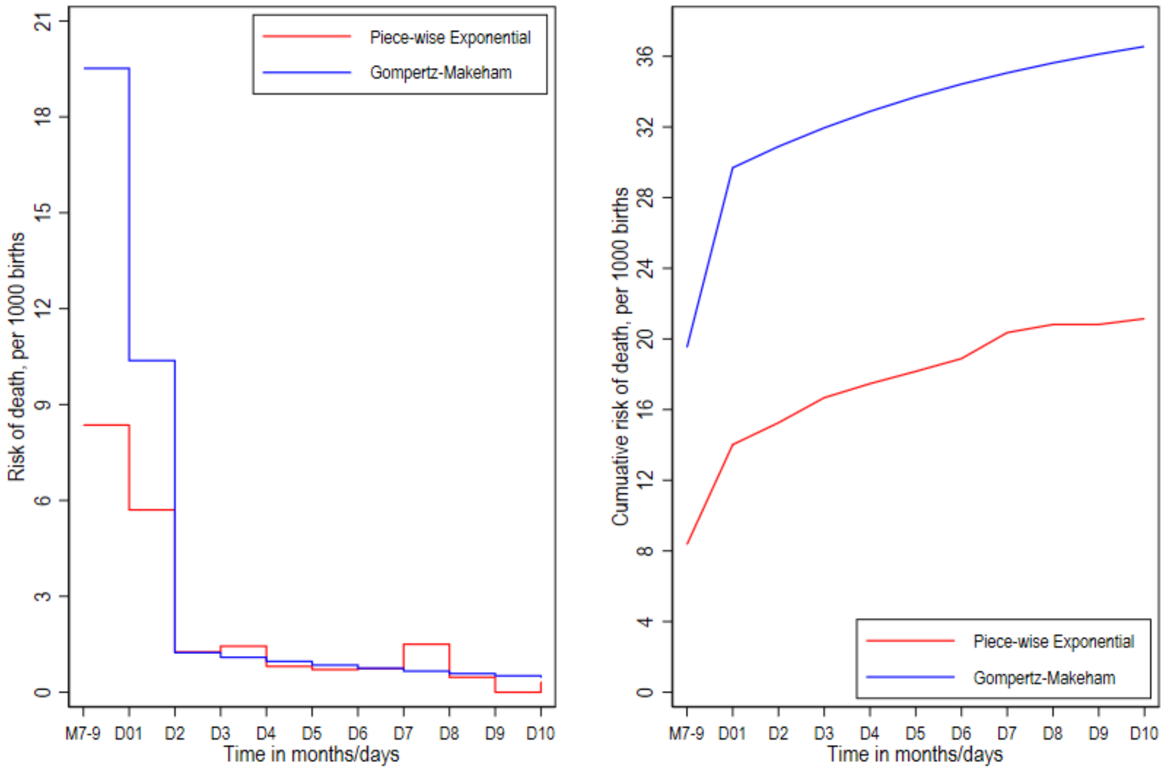

Figure S2: Egypt DHS-2014

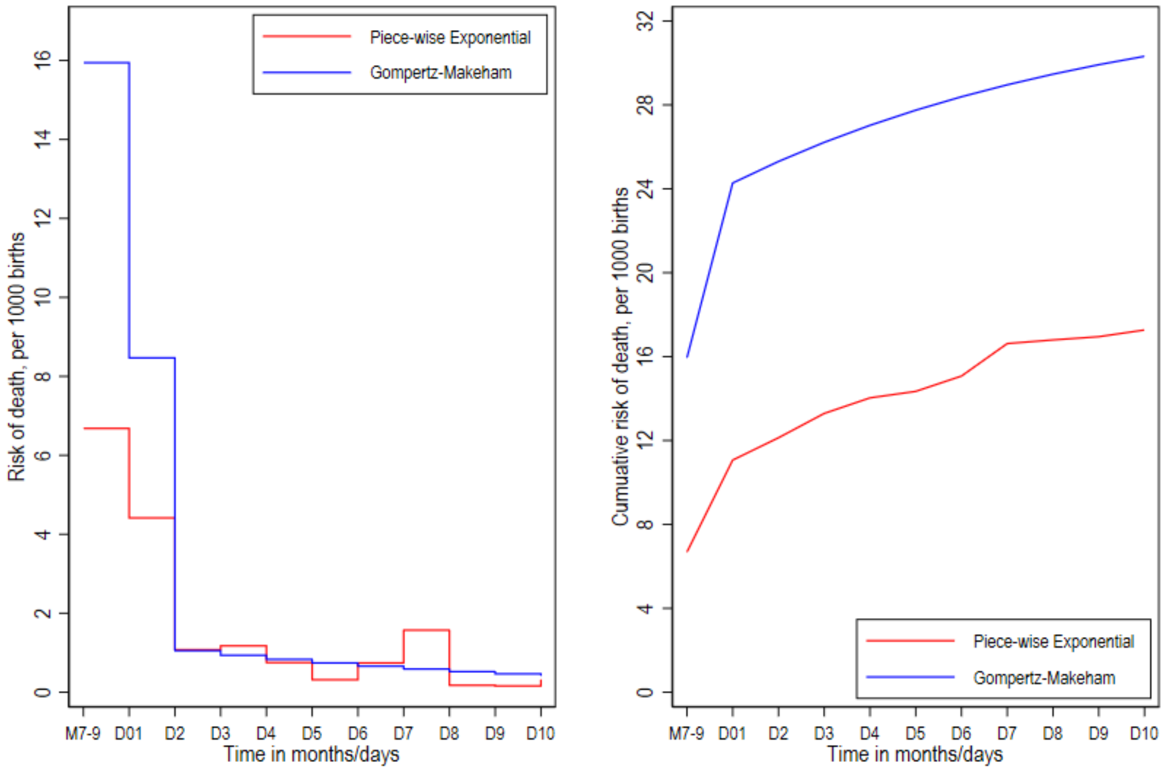

Figure S2: Jordan DHS-1990

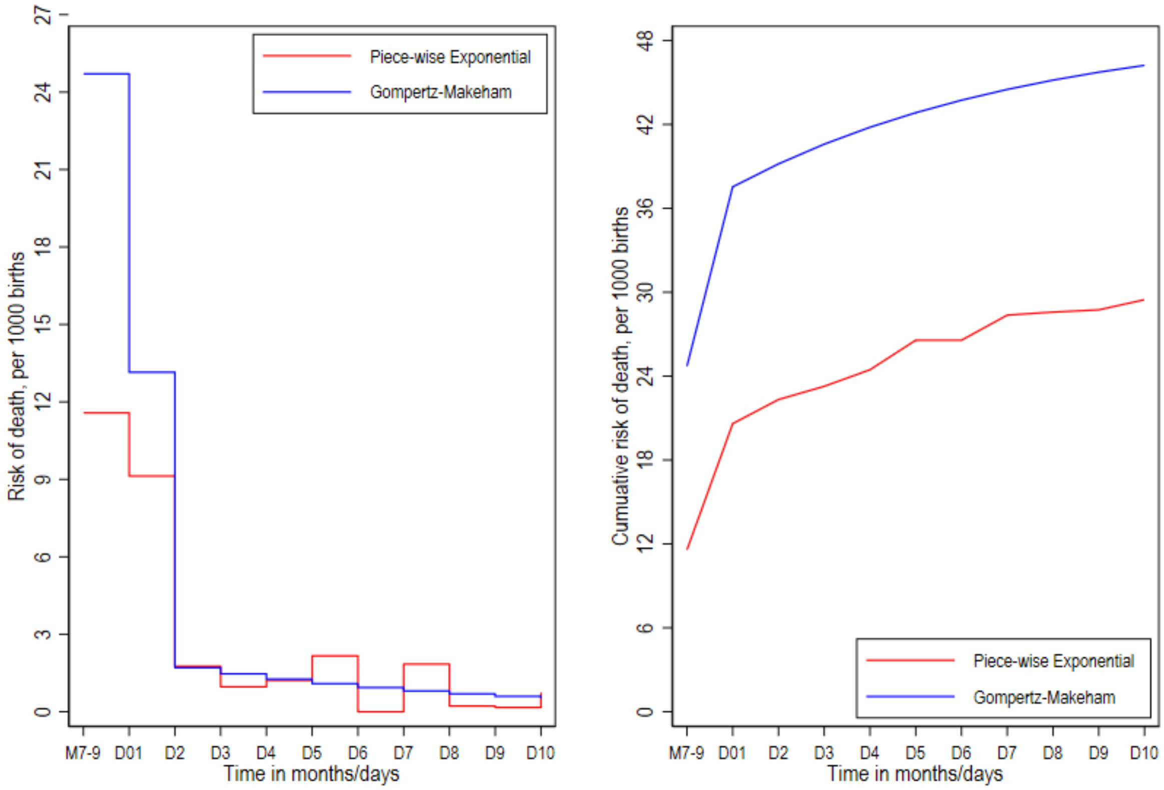

Figure S2: Jordan DHS-1997

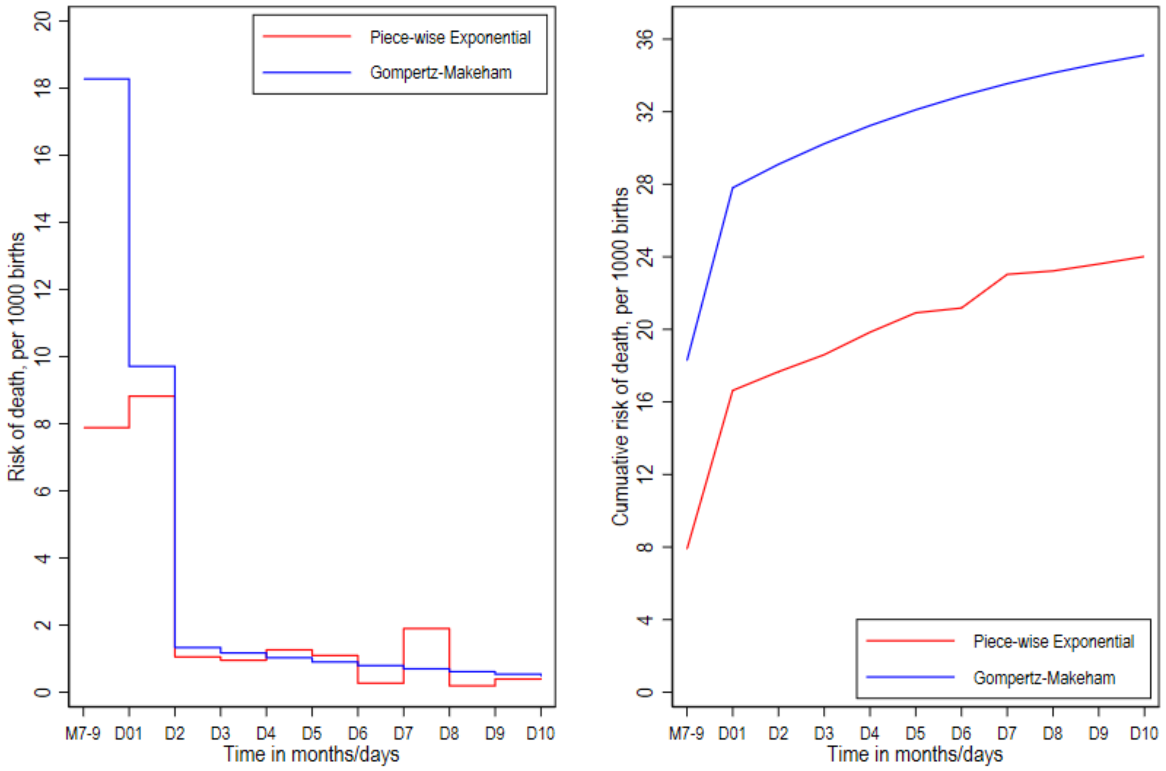

Figure S2: Jordan DHS-2002

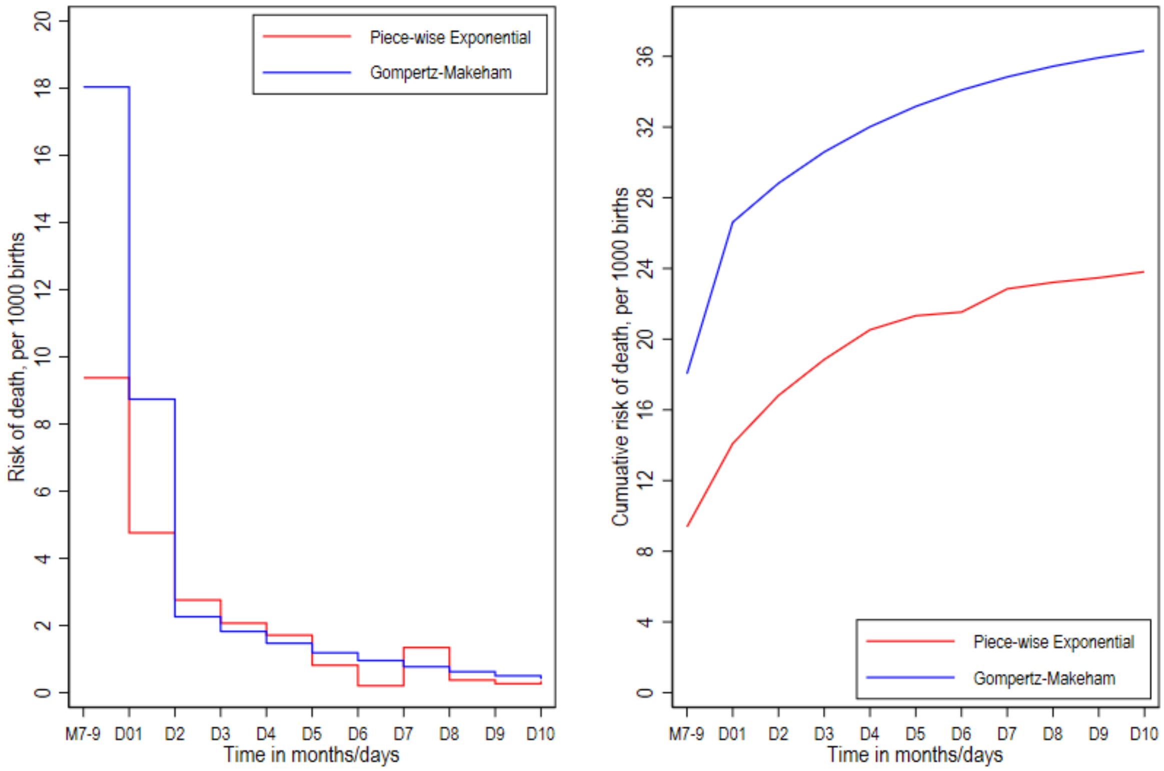

Figure S2: Jordan DHS-2007

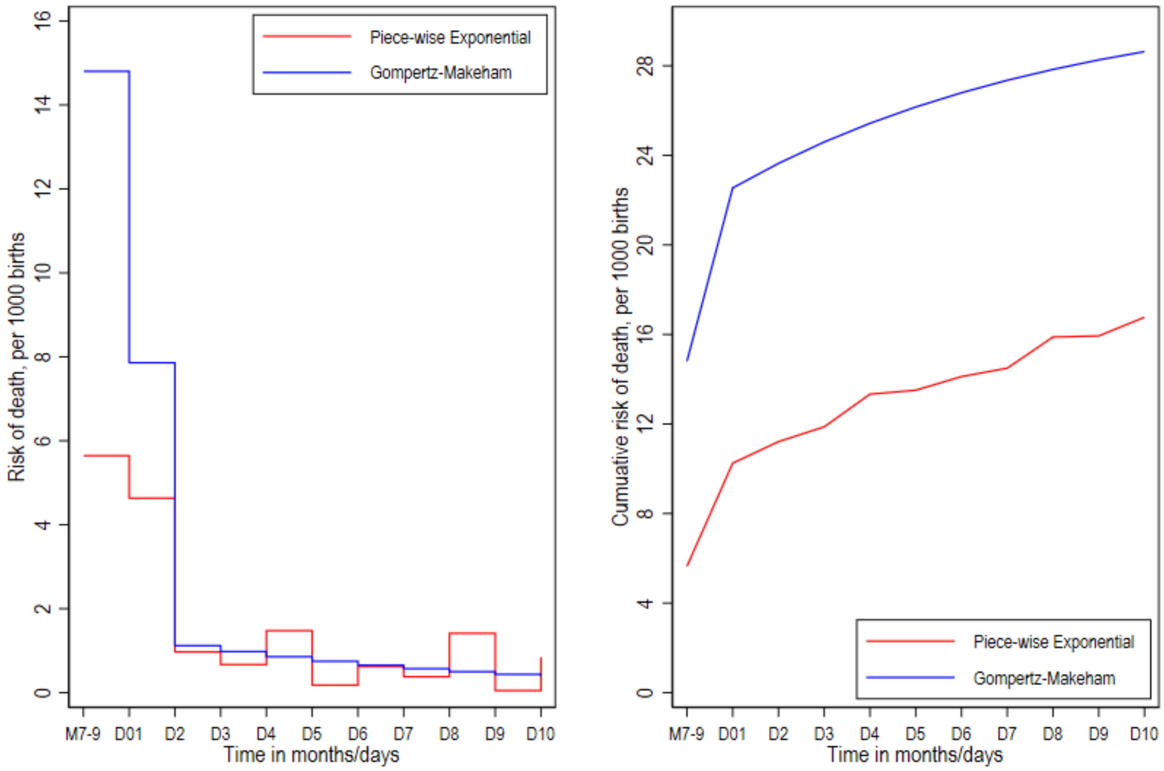

Figure S2: Jordan DHS-2009

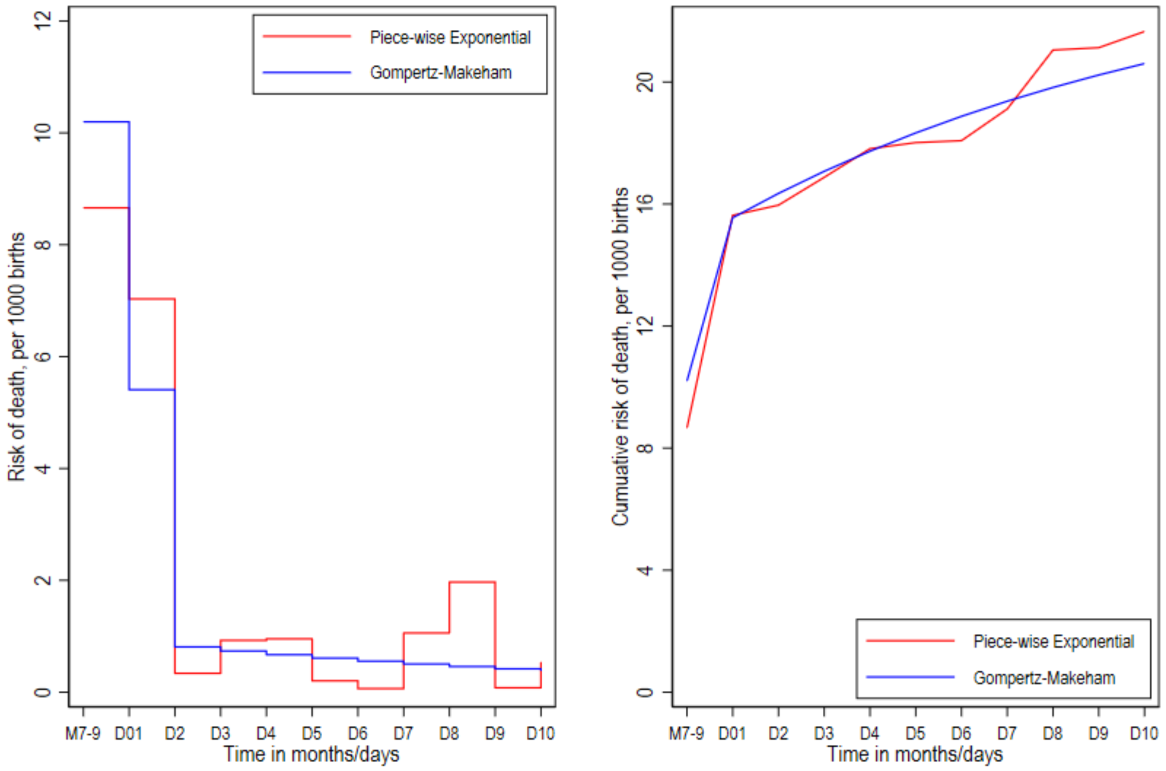

Figure S2: Jordan DHS-2012

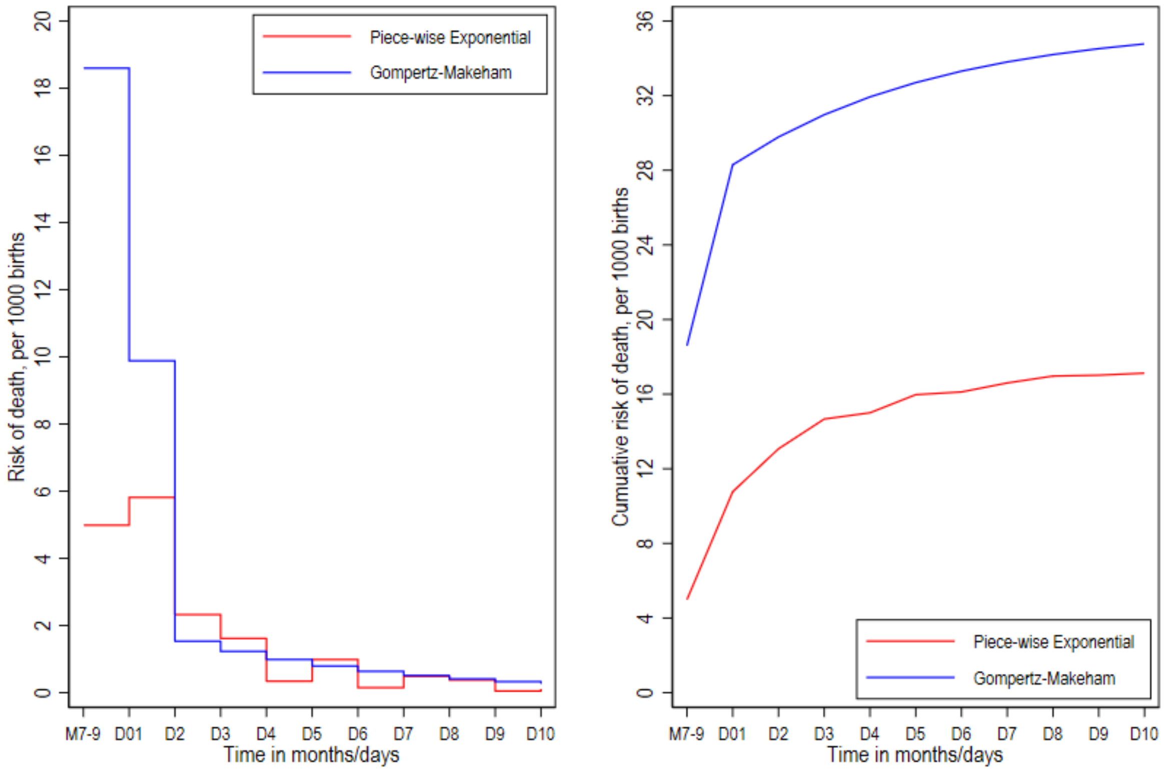

Figure S2: Jordan DHS-2017/18

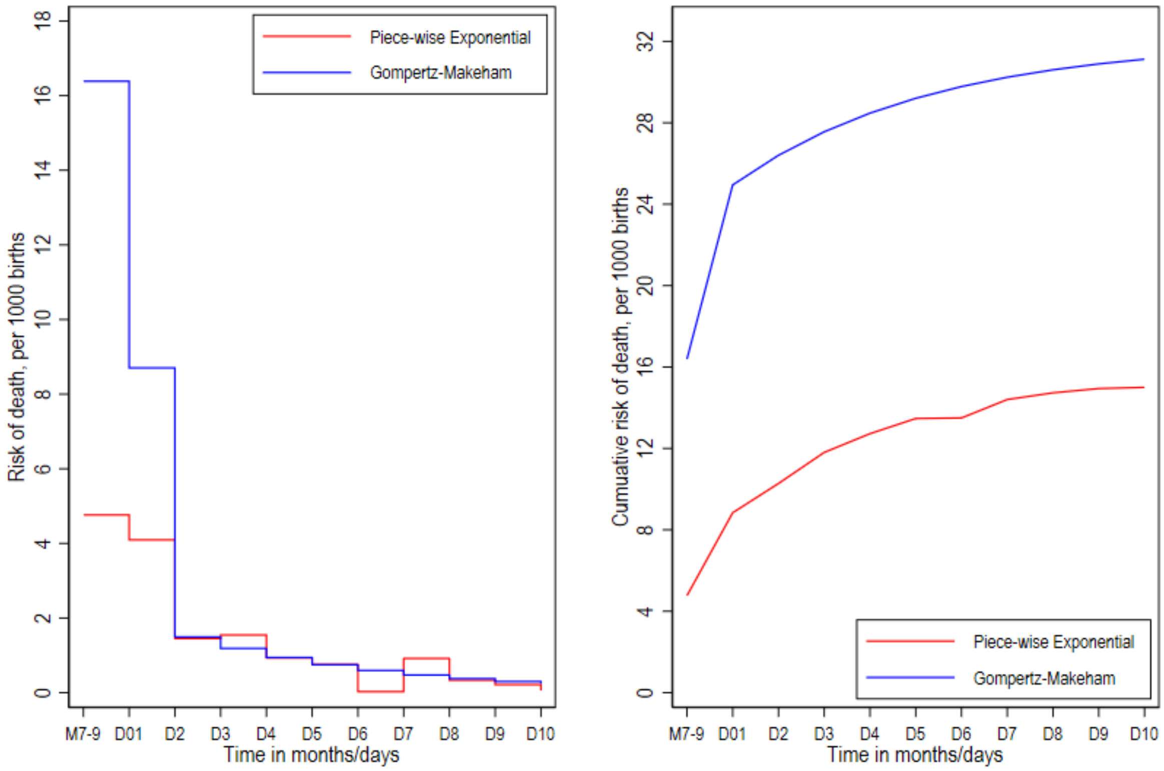

Figure S2: Morocco DHS-1992

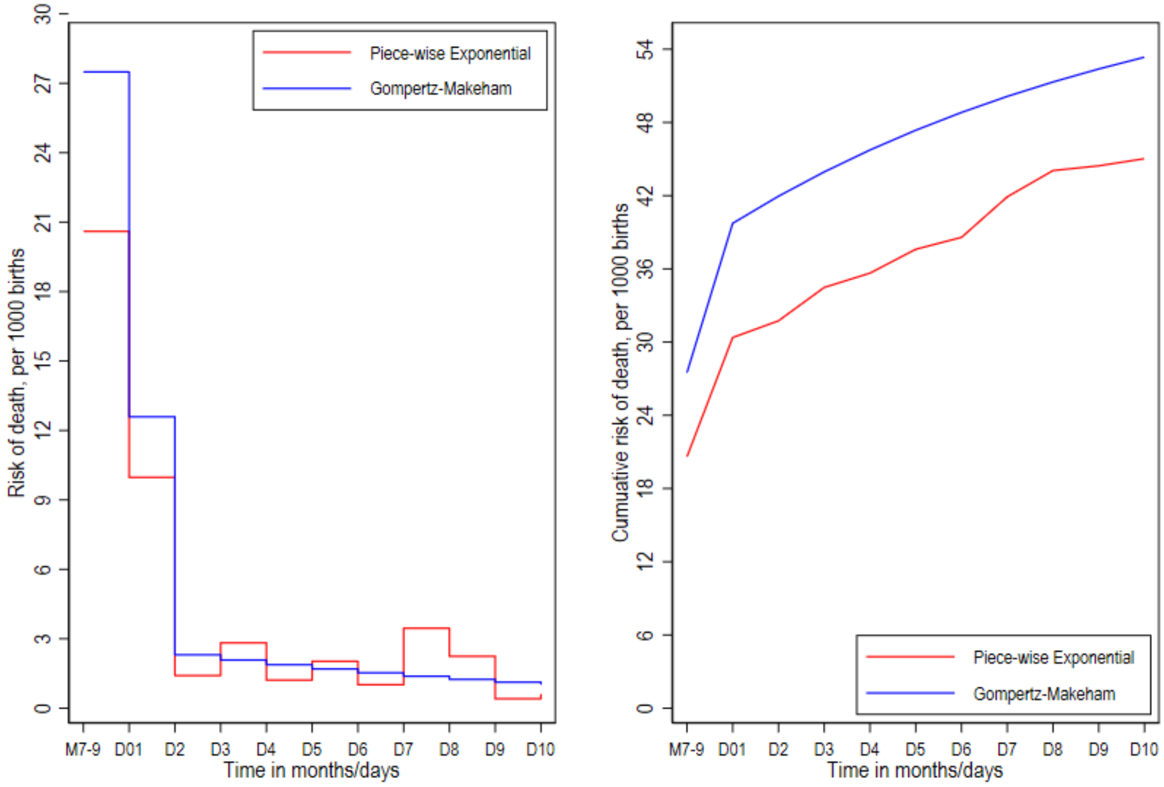

Figure S2: Morocco DHS-2003/4

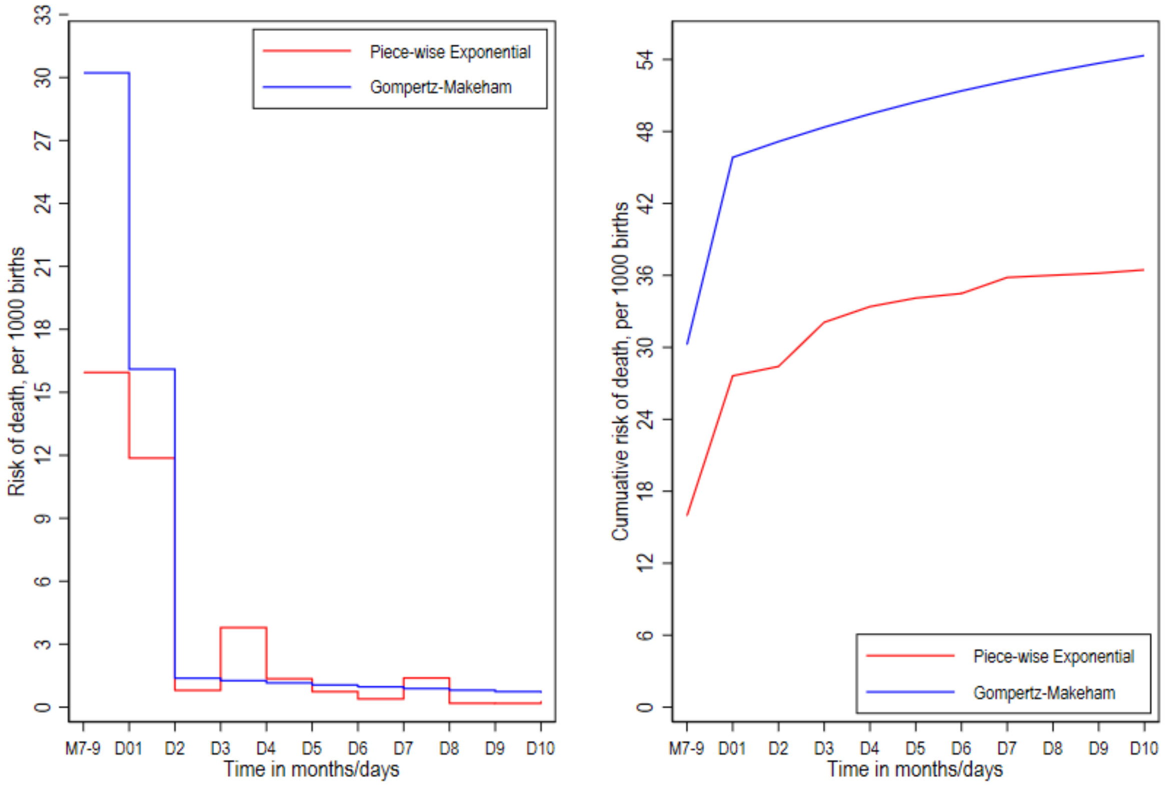

Figure S2: Türkiye DHS-1993

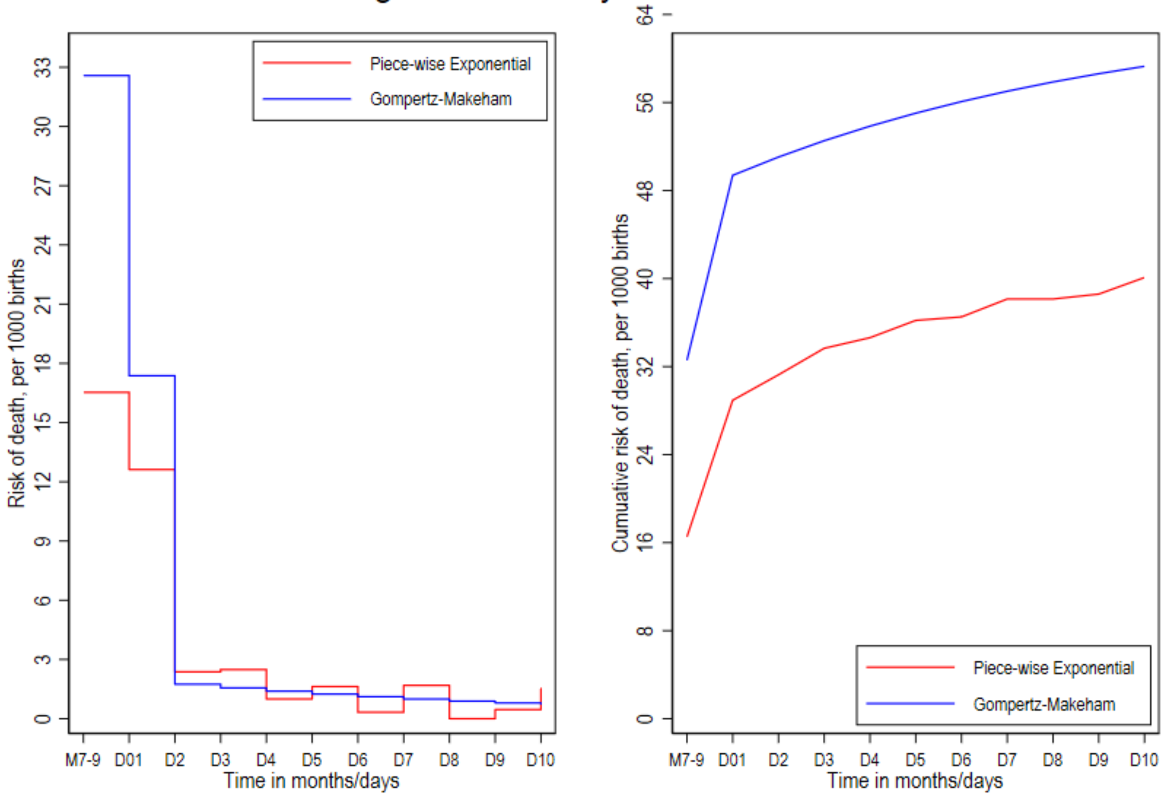

Figure S2: Türkiye DHS-1998

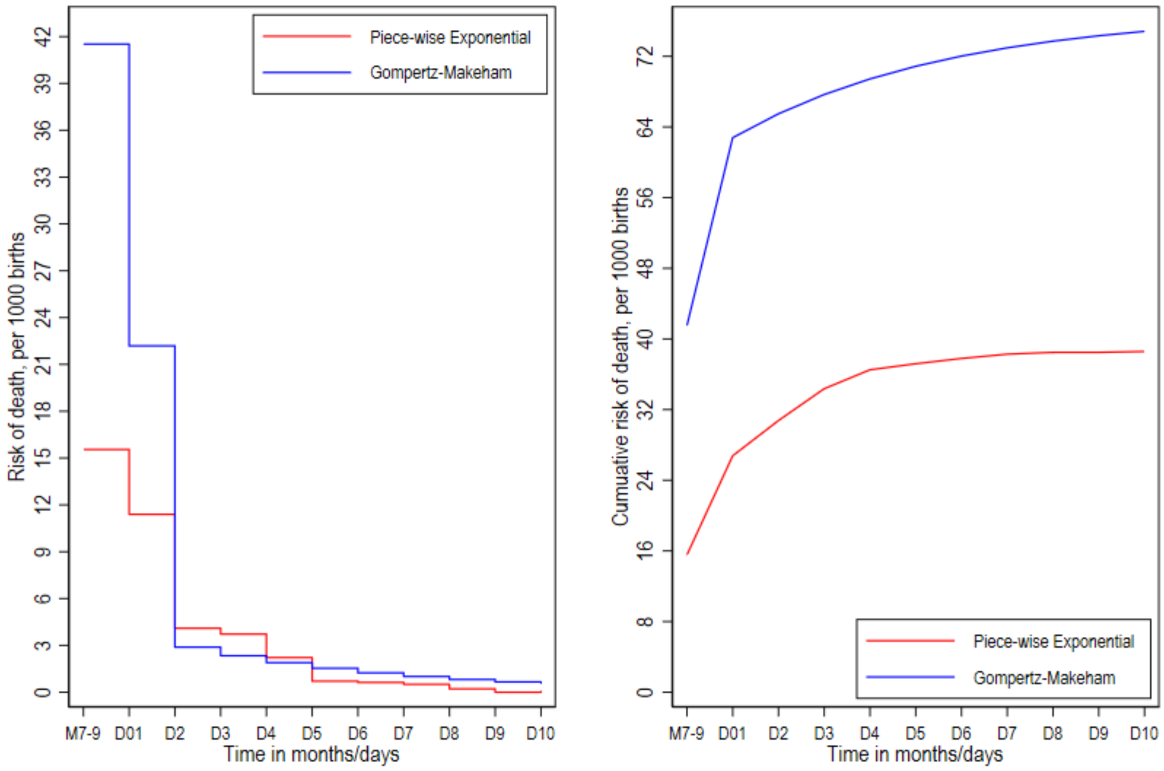

Figure S2: Türkiye DHS-2003/4

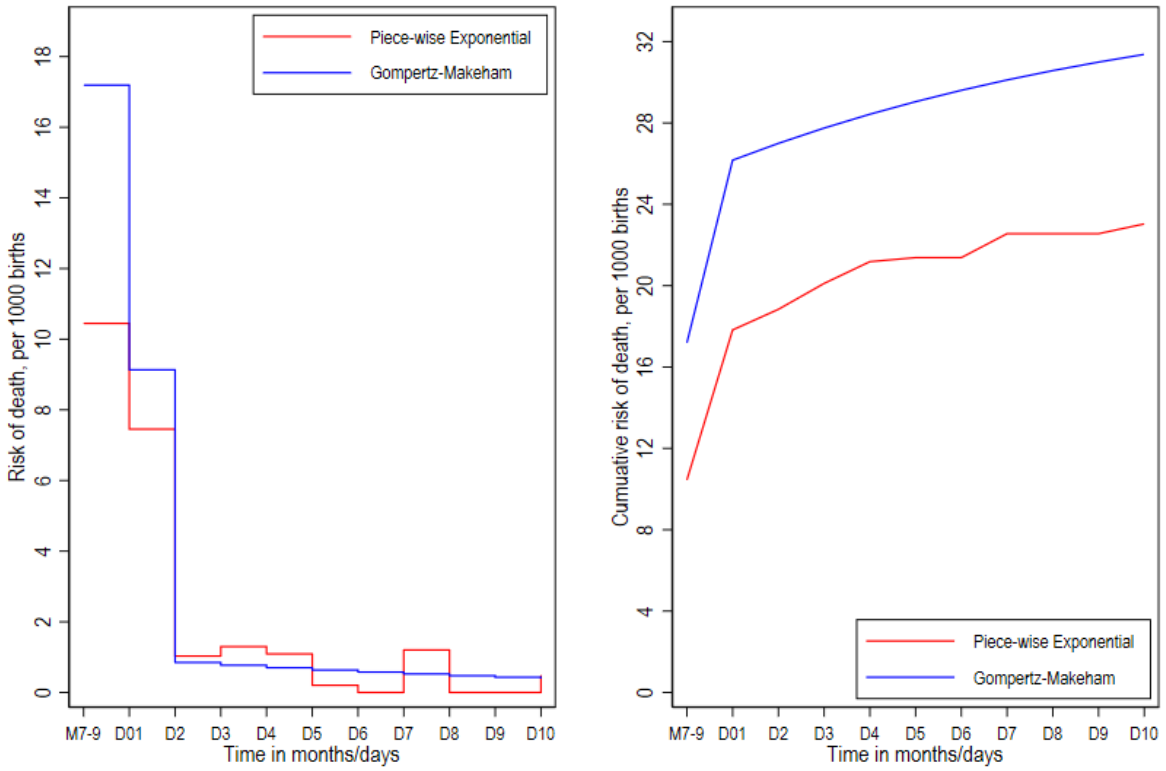

Figure S2: Yemen DHS-2013

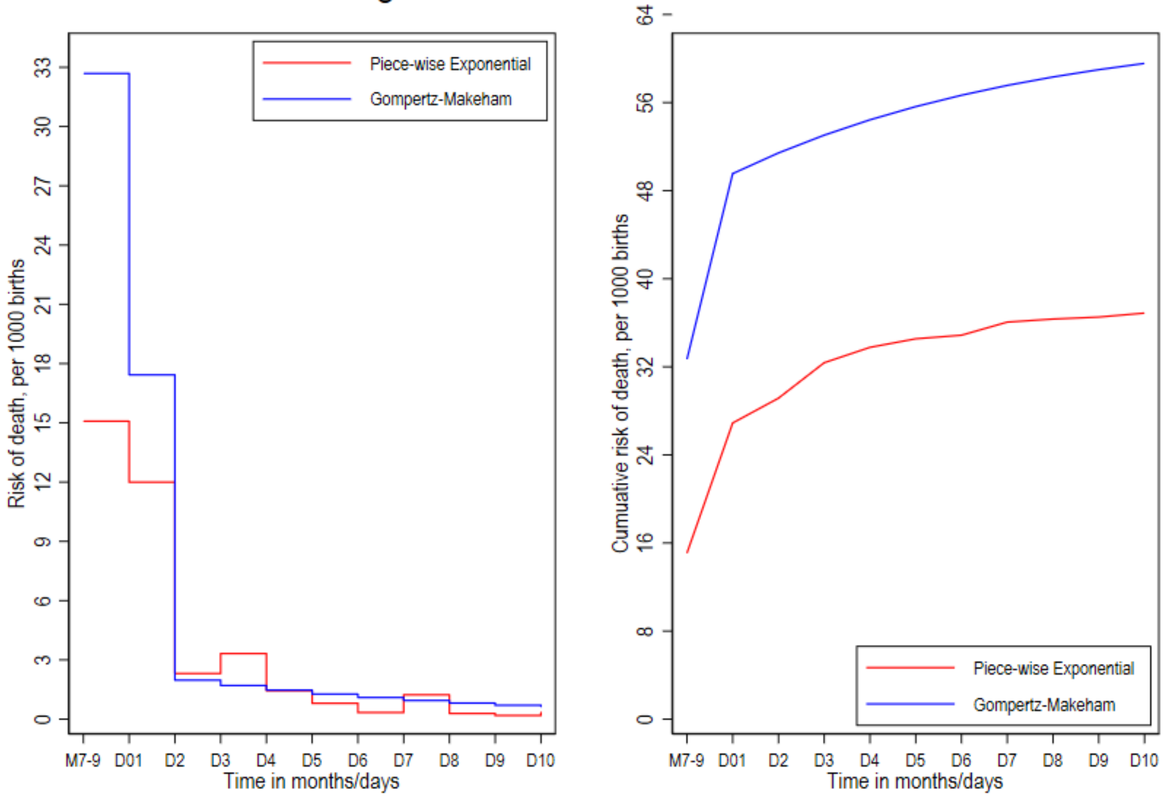

Figure S2: Tajikistan DHS-2012

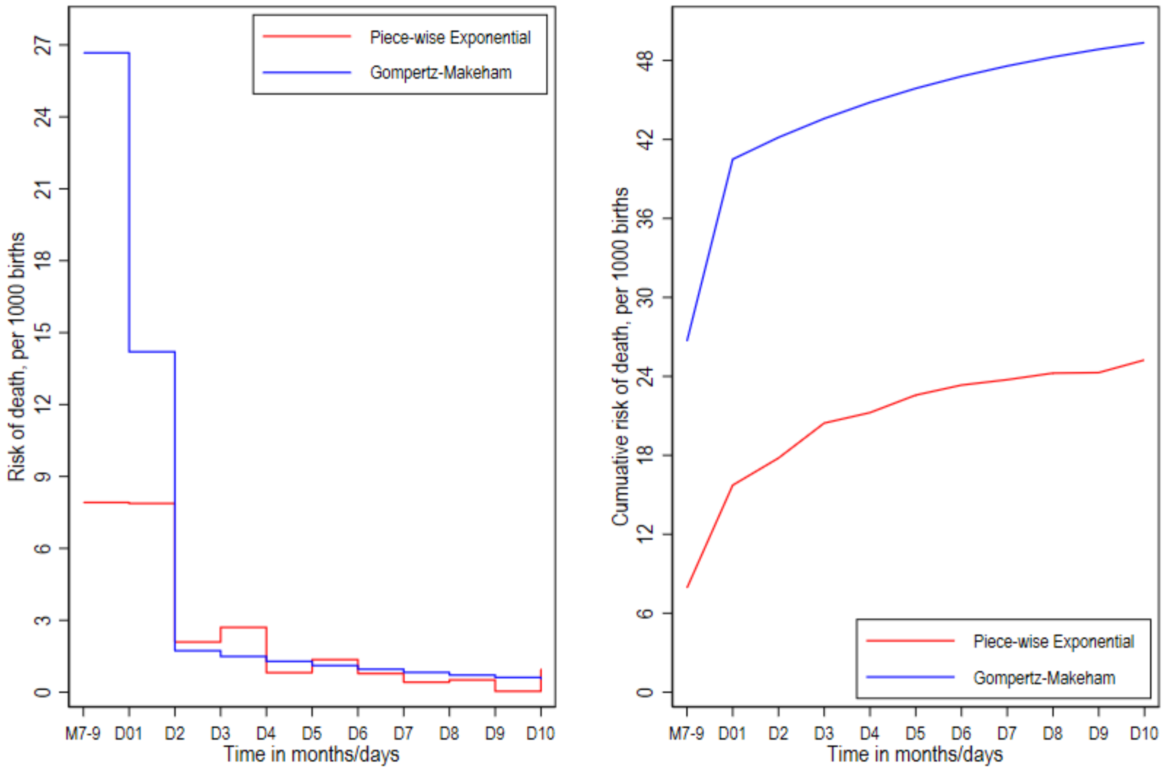

Figure S2: Tajikistan DHS-2017

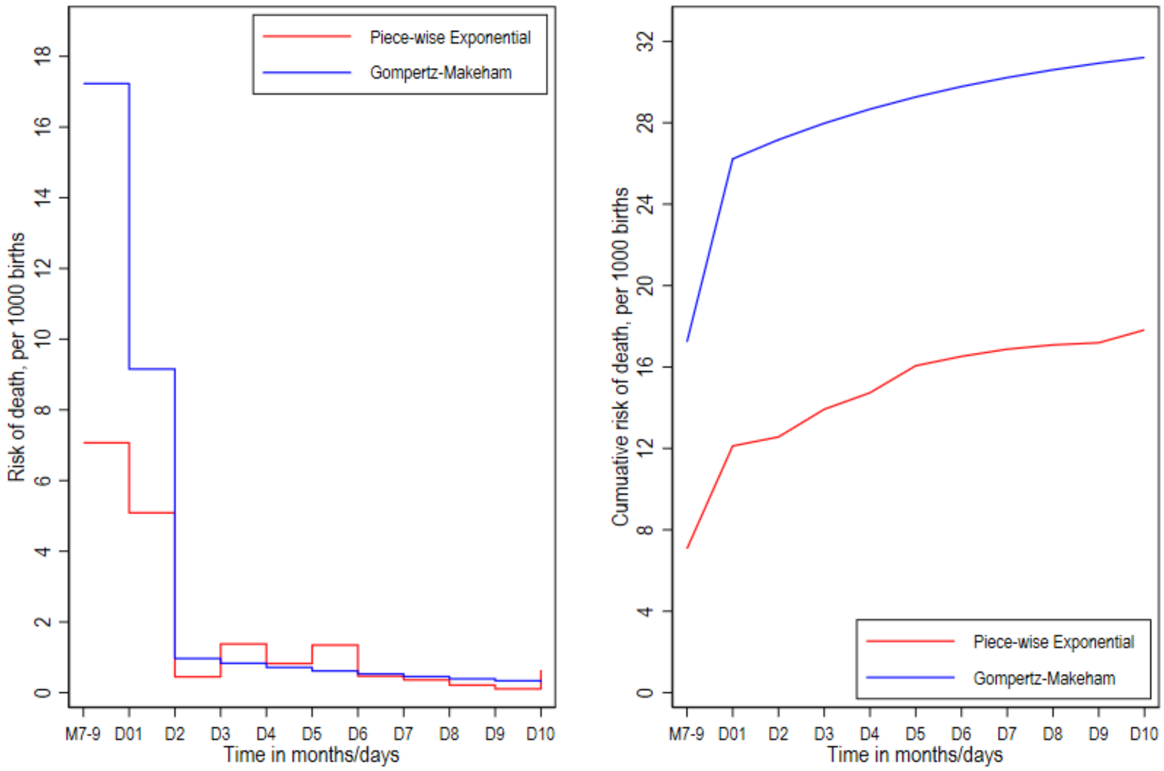

Figure S2: Bangladesh DHS-1993/94

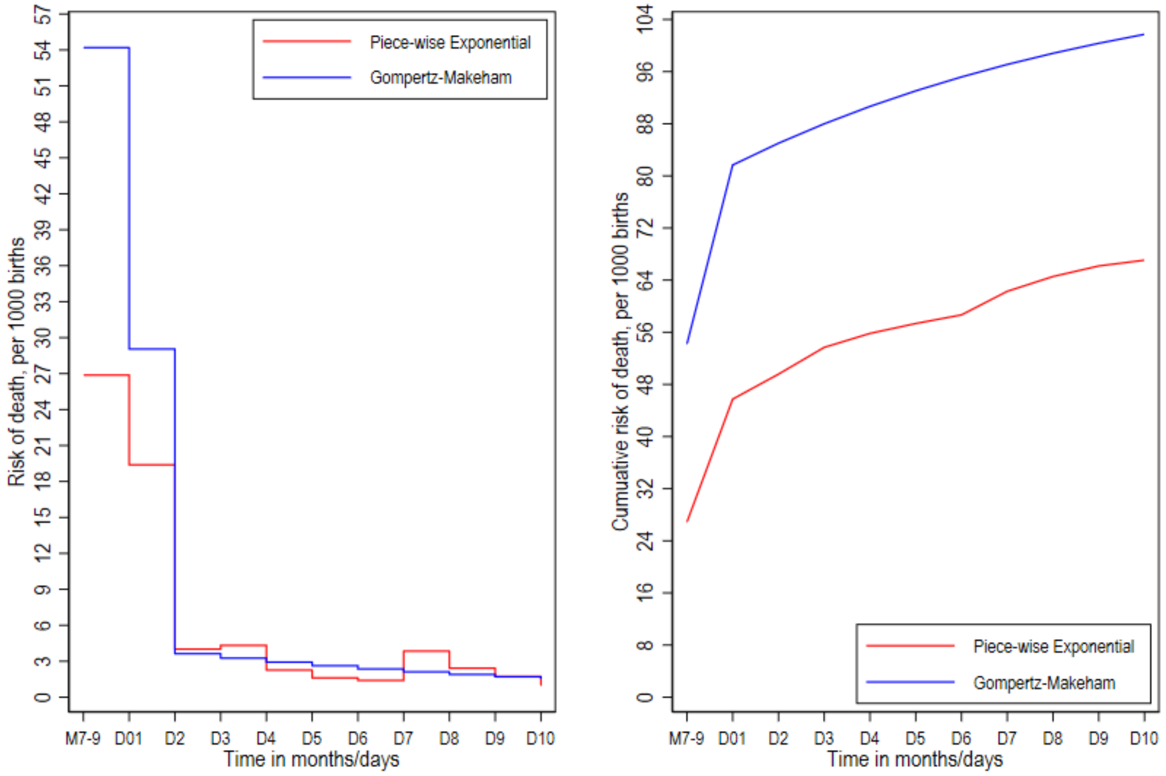

Figure S2: Bangladesh DHS-1996/97

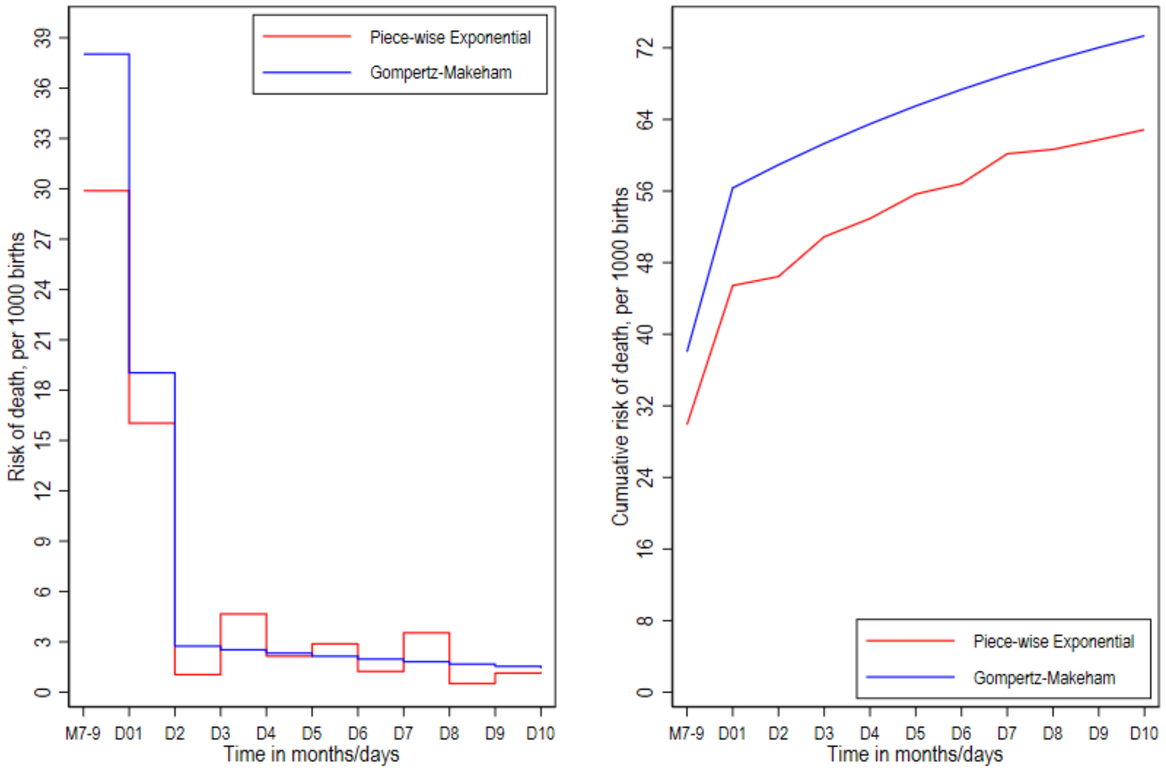

Figure S2: Bangladesh DHS-1999/0

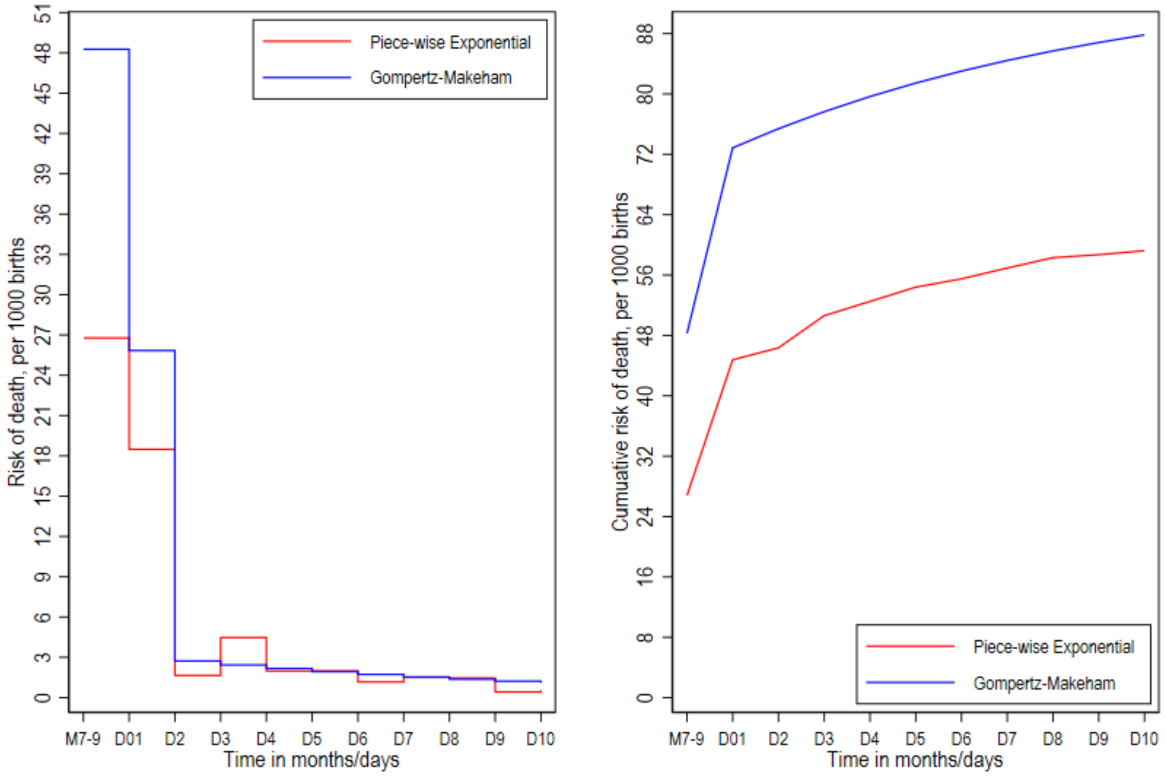

Figure S2: Bangladesh DHS-2004

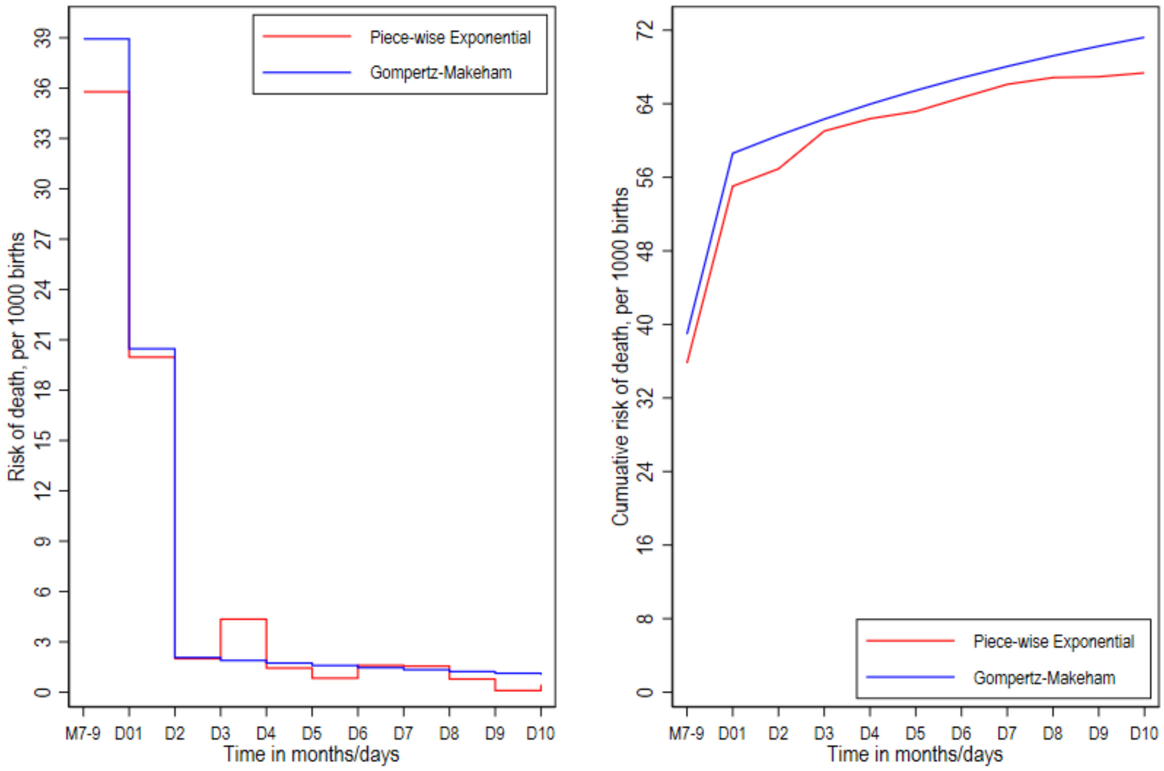

Figure S2: Bangladesh DHS-2007

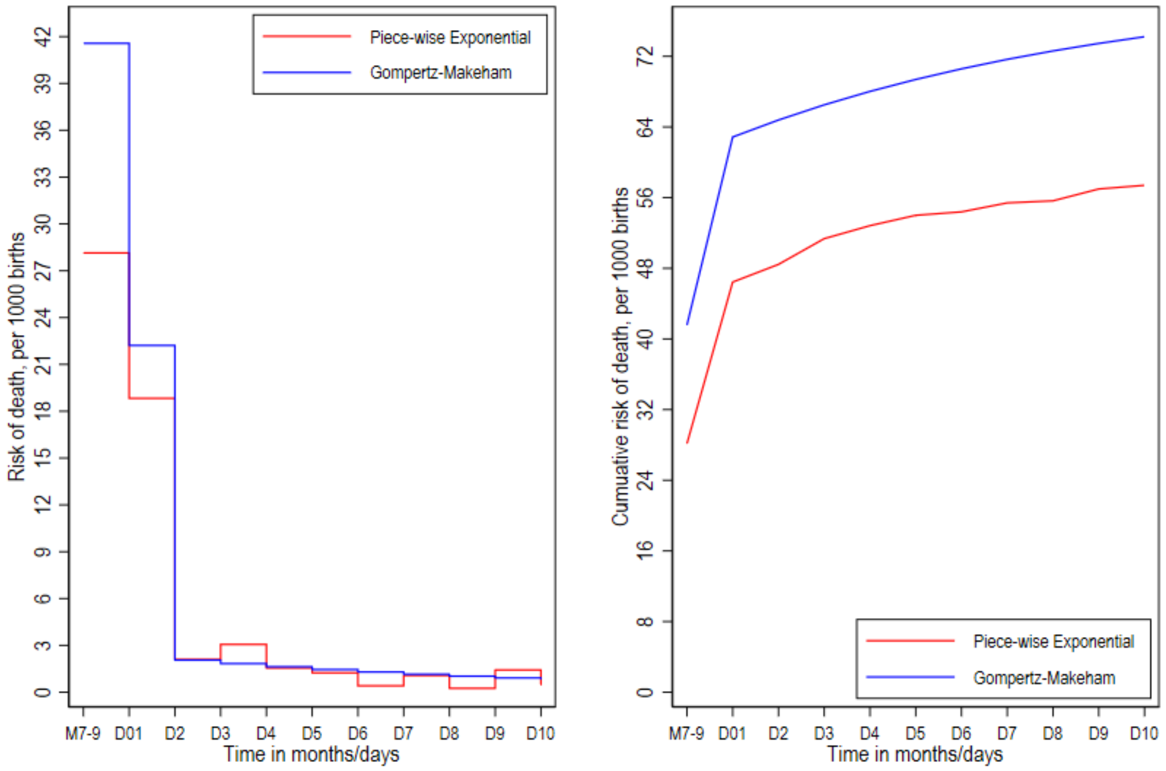

Figure S2: Bangladesh DHS-2011

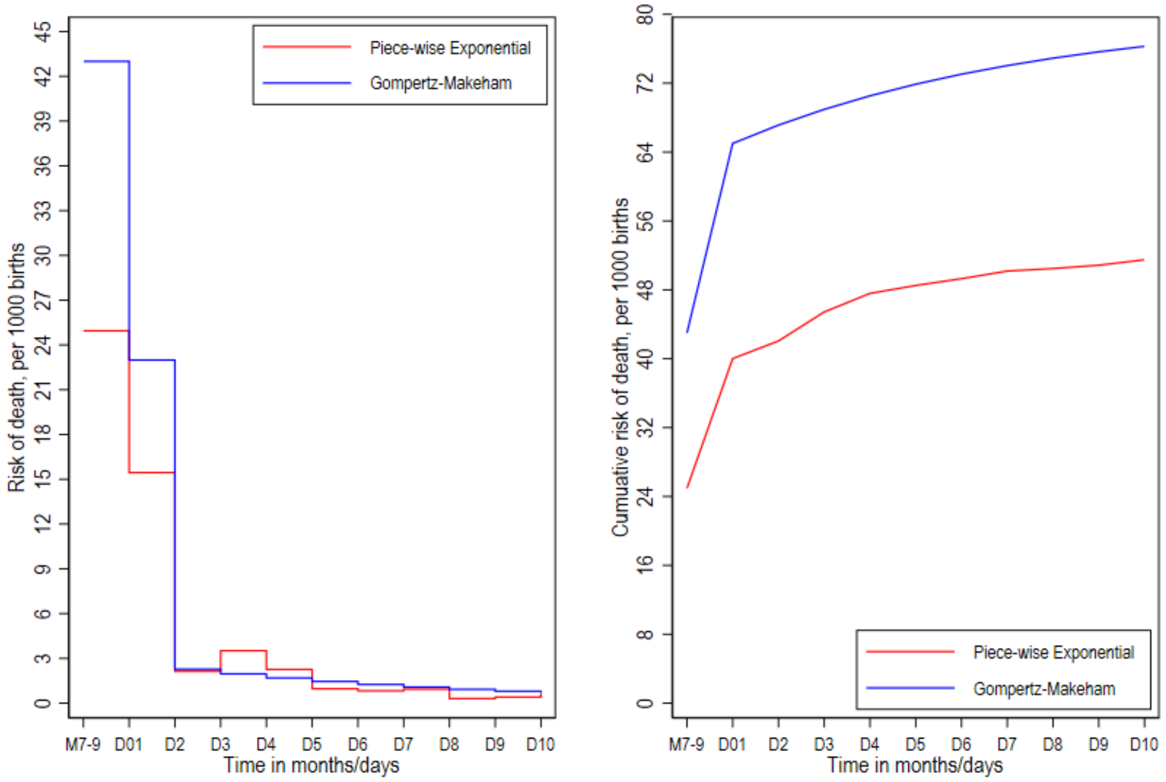

Figure S2: Bangladesh DHS-2014

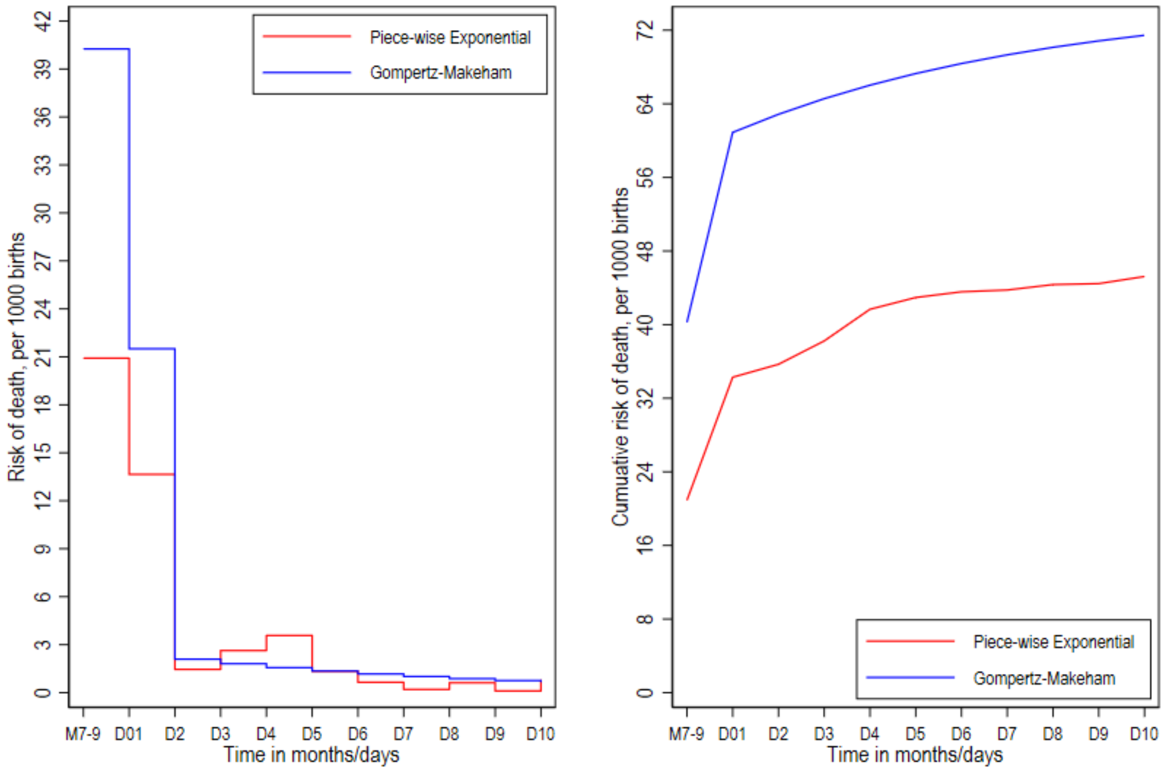

Figure S2: Bangladesh DHS-2017/18

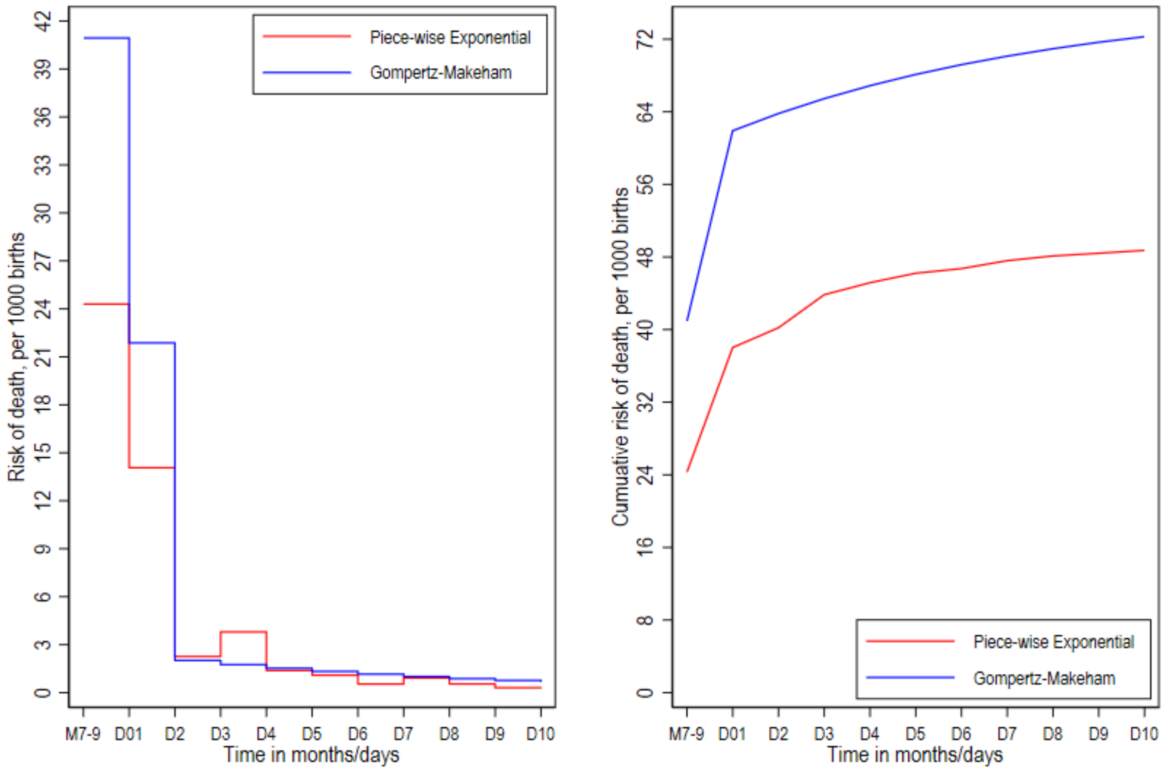

Figure S2: Cambodia DHS-2010/11

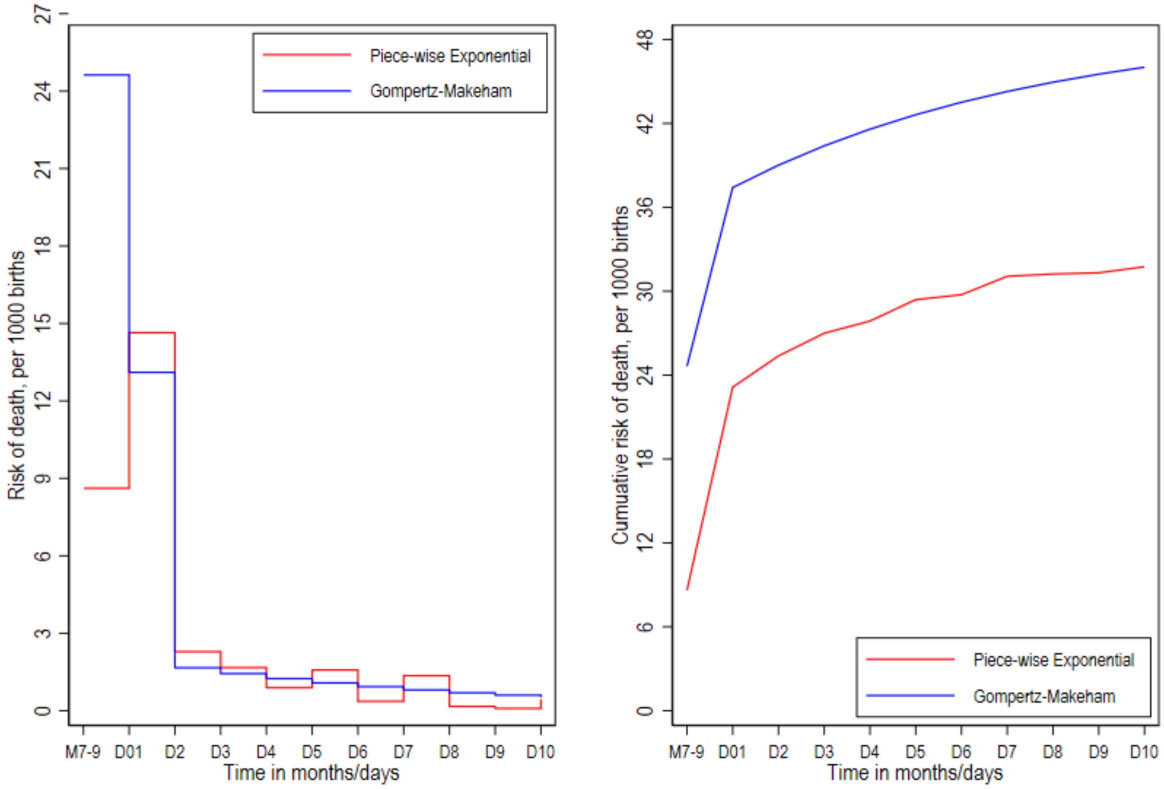

Figure S2: Cambodia DHS-2014

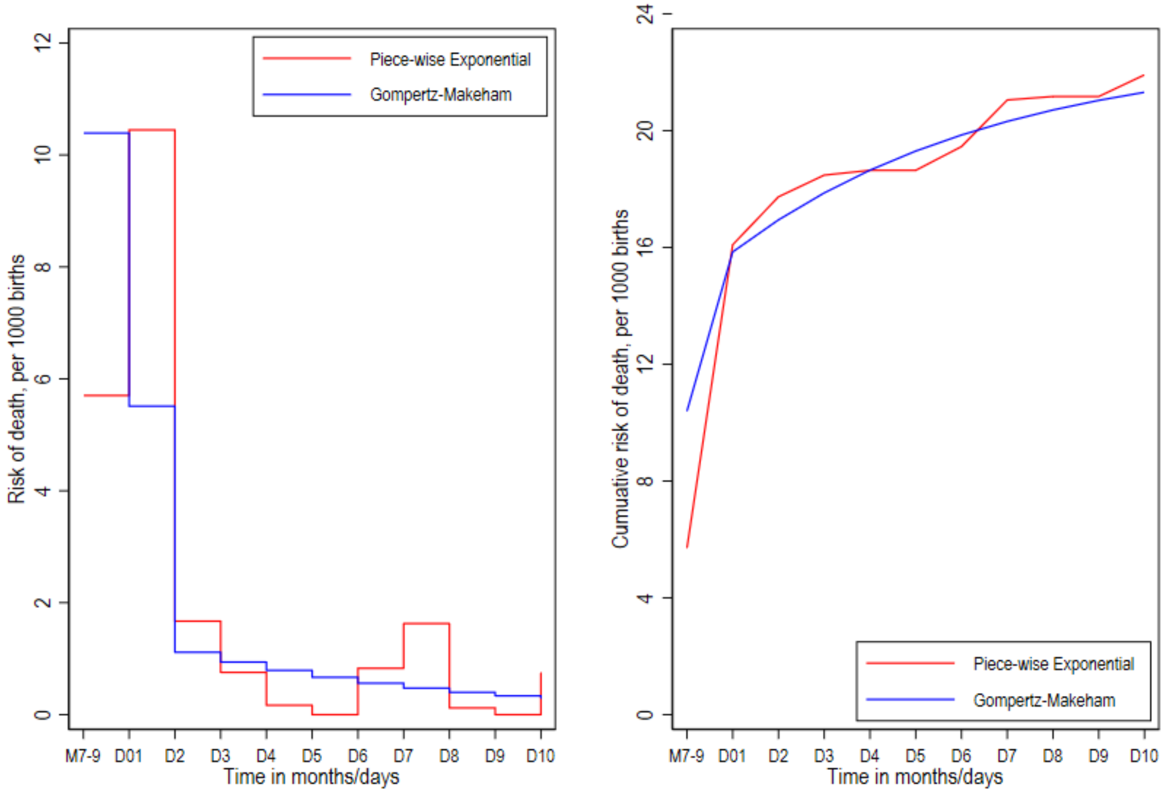

Figure S2: India DHS-2005/6

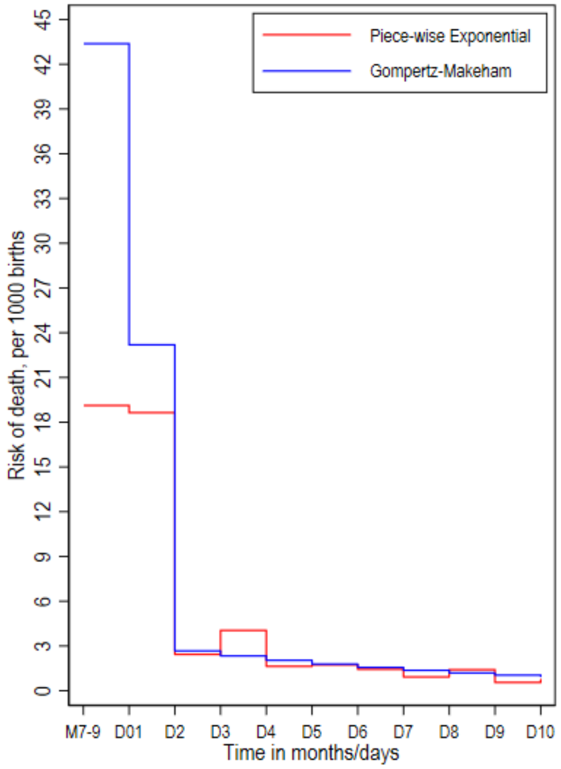

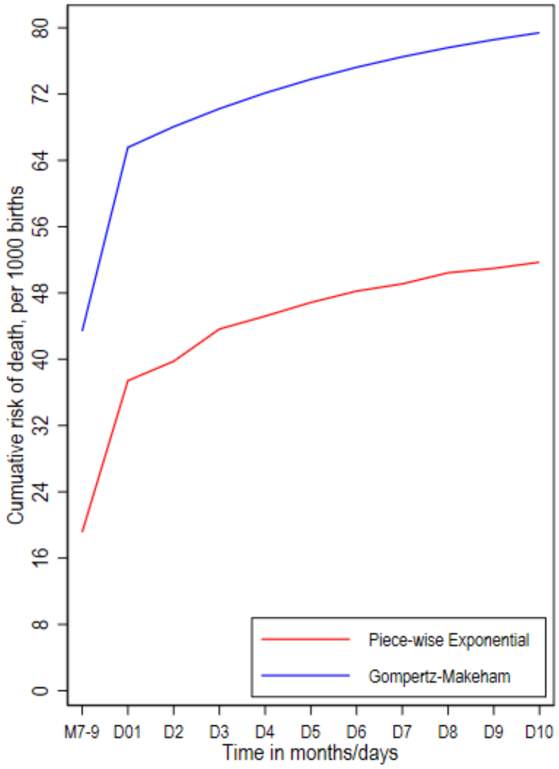

Figure S2: India DHS-2015/16

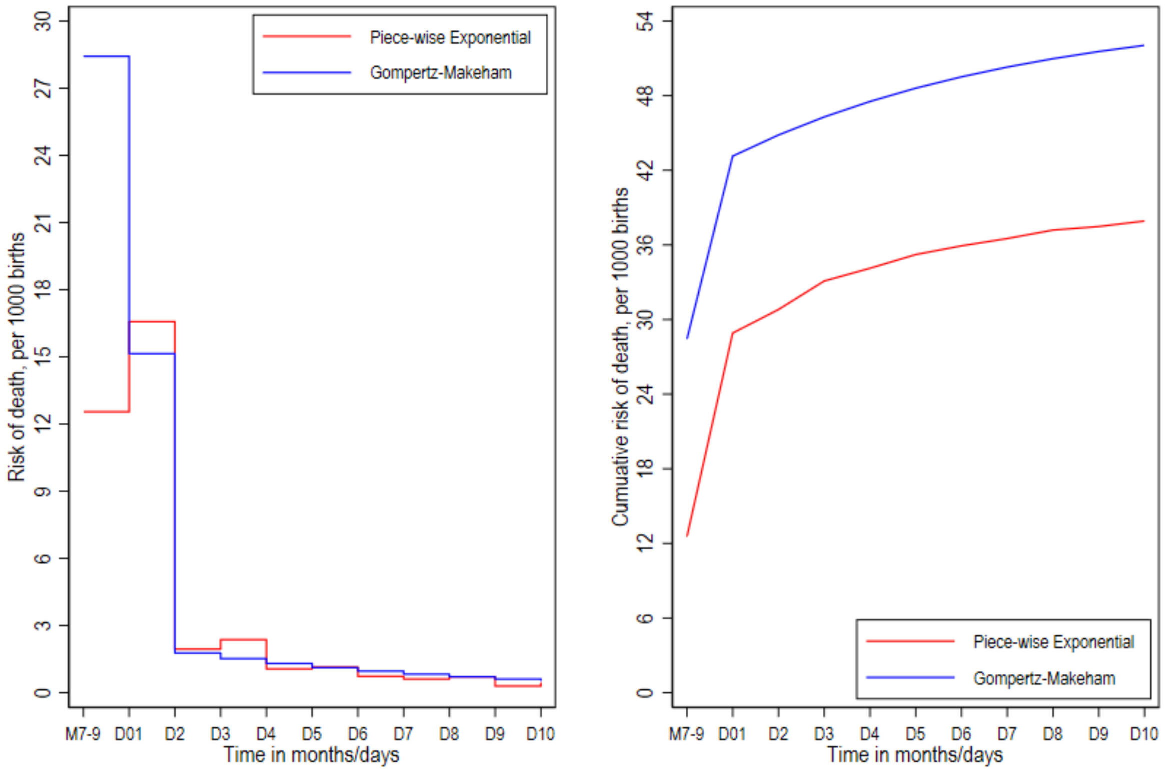

Figure S2: India DHS-2019/21

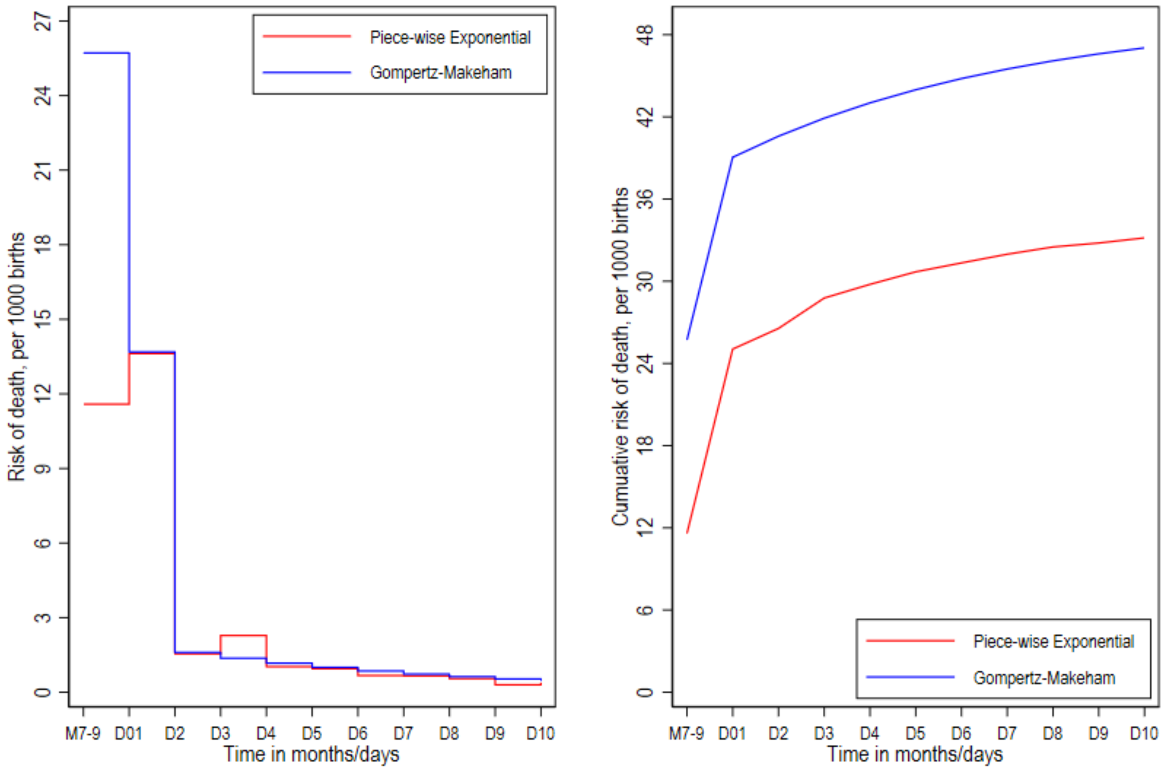

Figure S2: Indonesia DHS-1991

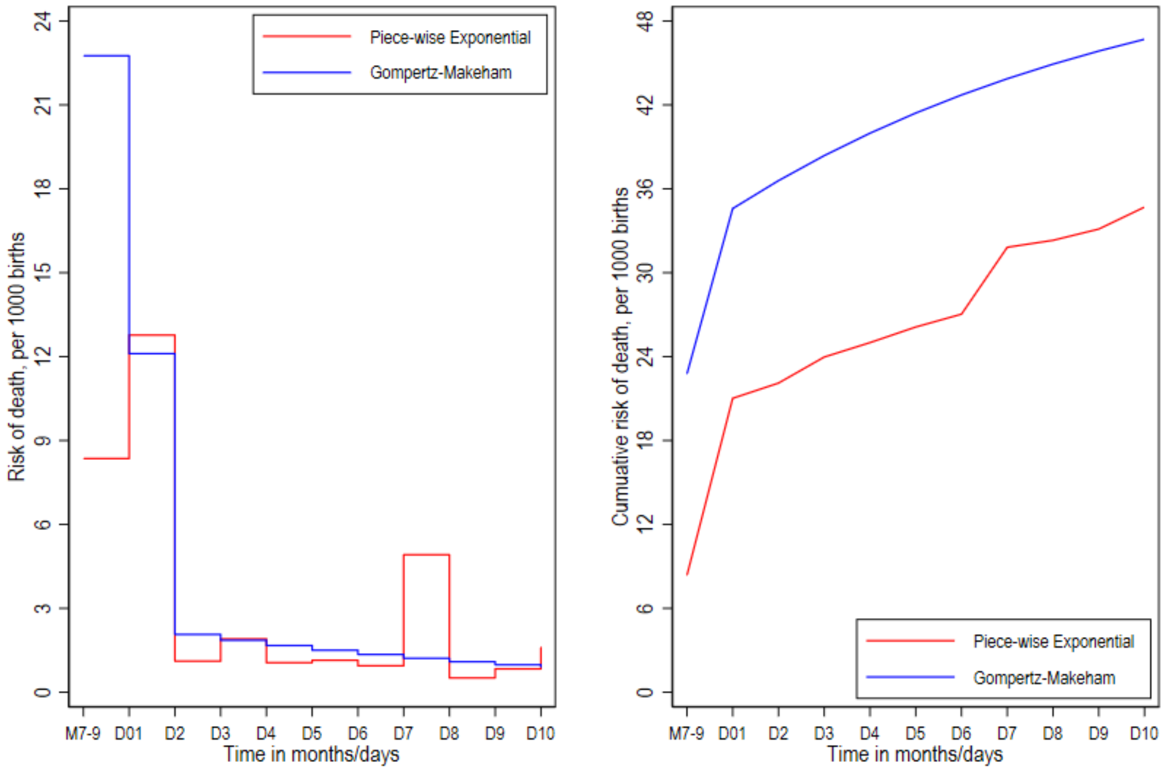

Figure S2: Indonesia DHS-1994

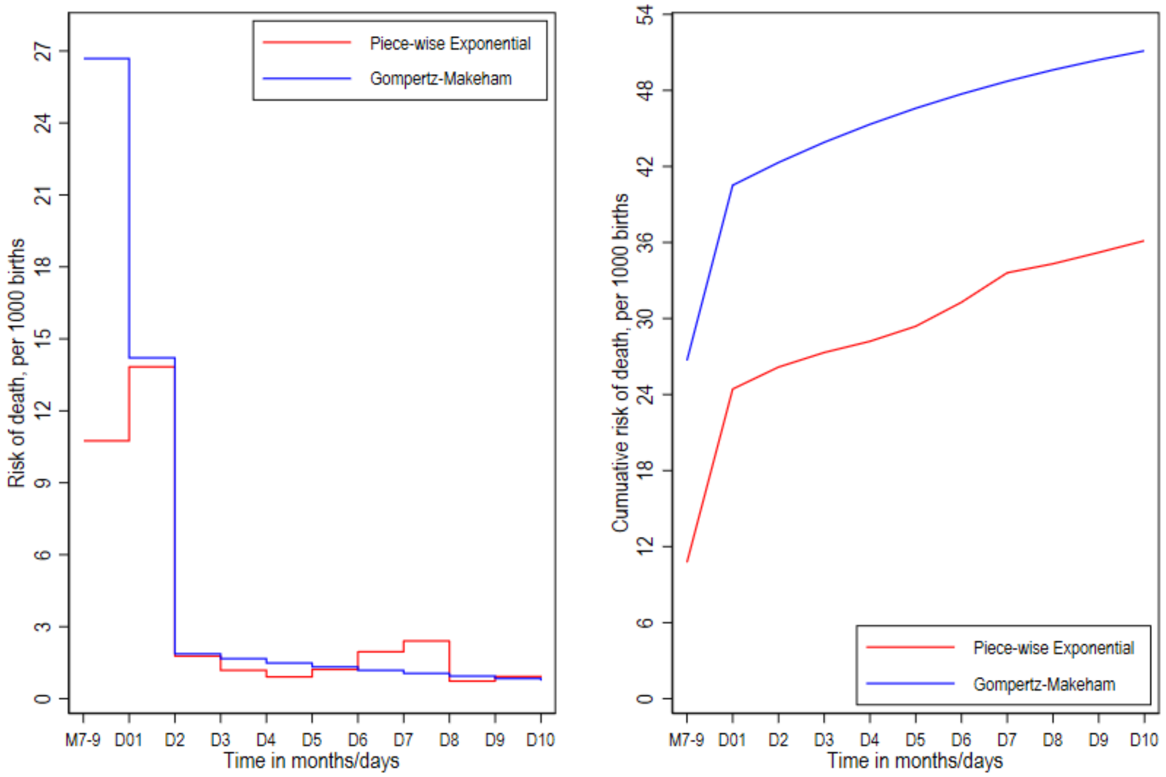

Figure S2: Indonesia DHS-1997

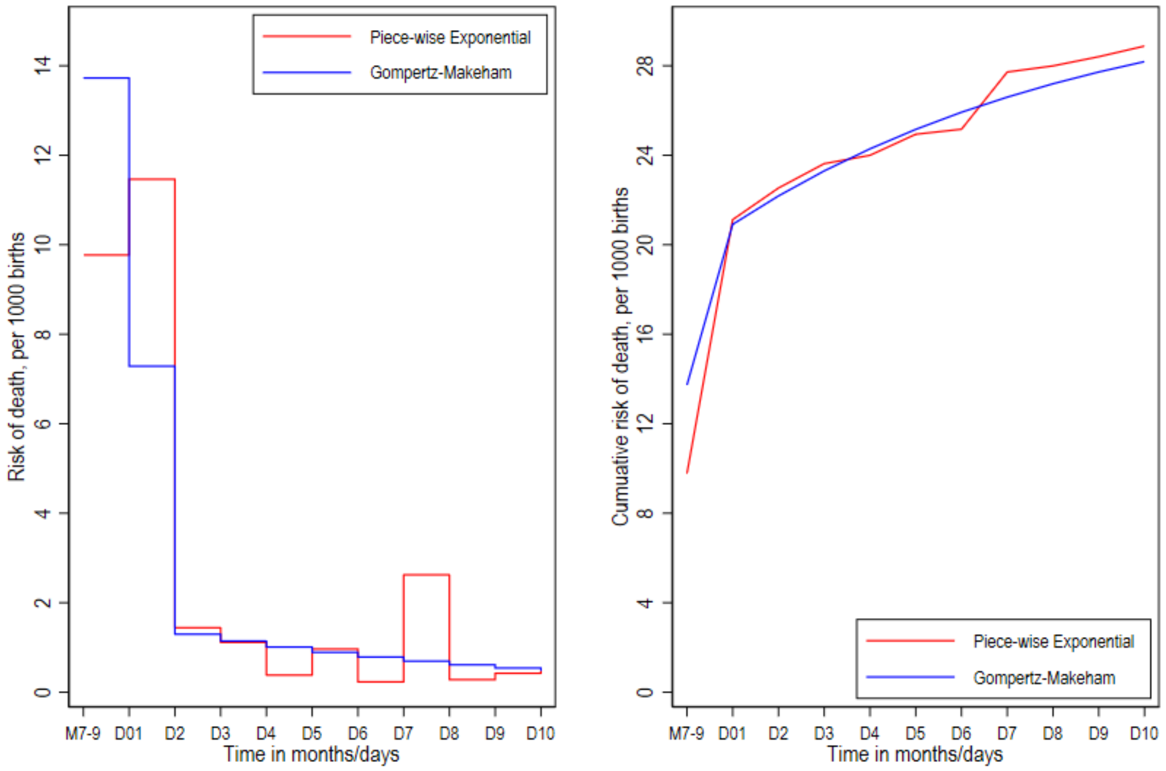

Figure S2: Indonesia DHS-2002/3

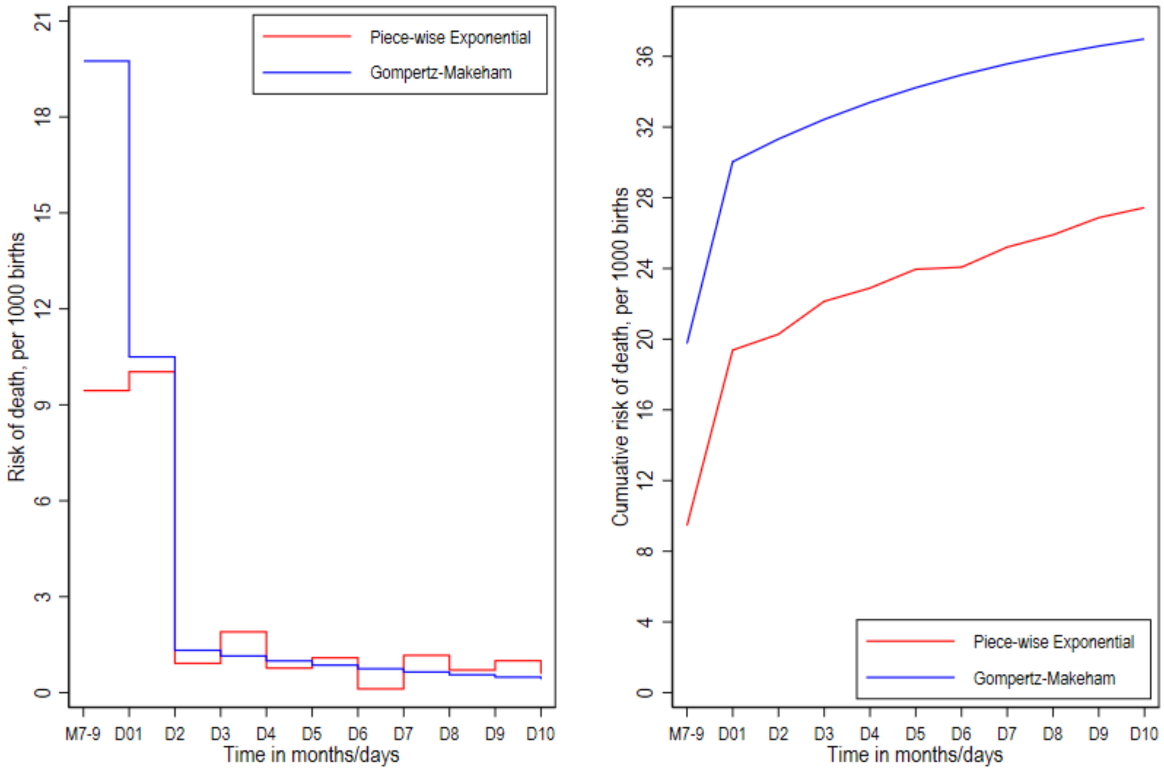

Figure S2: Indonesia DHS-2007

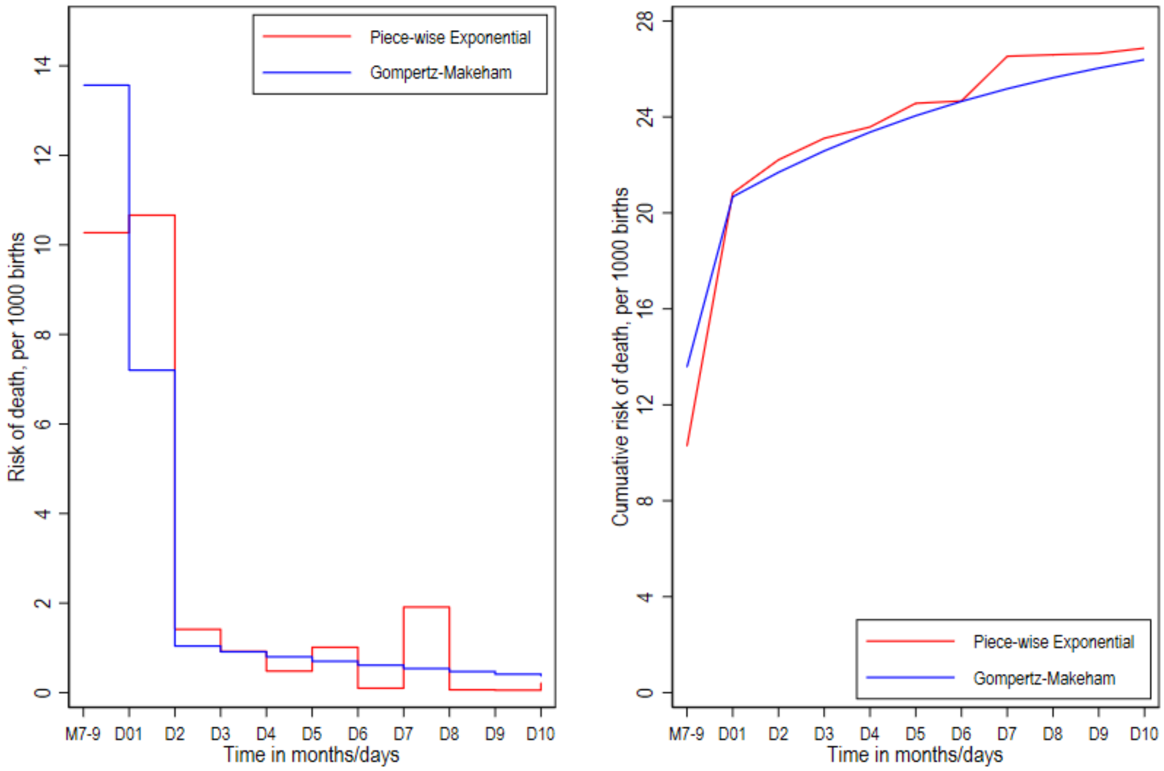

Figure S2: Indonesia DHS-2012

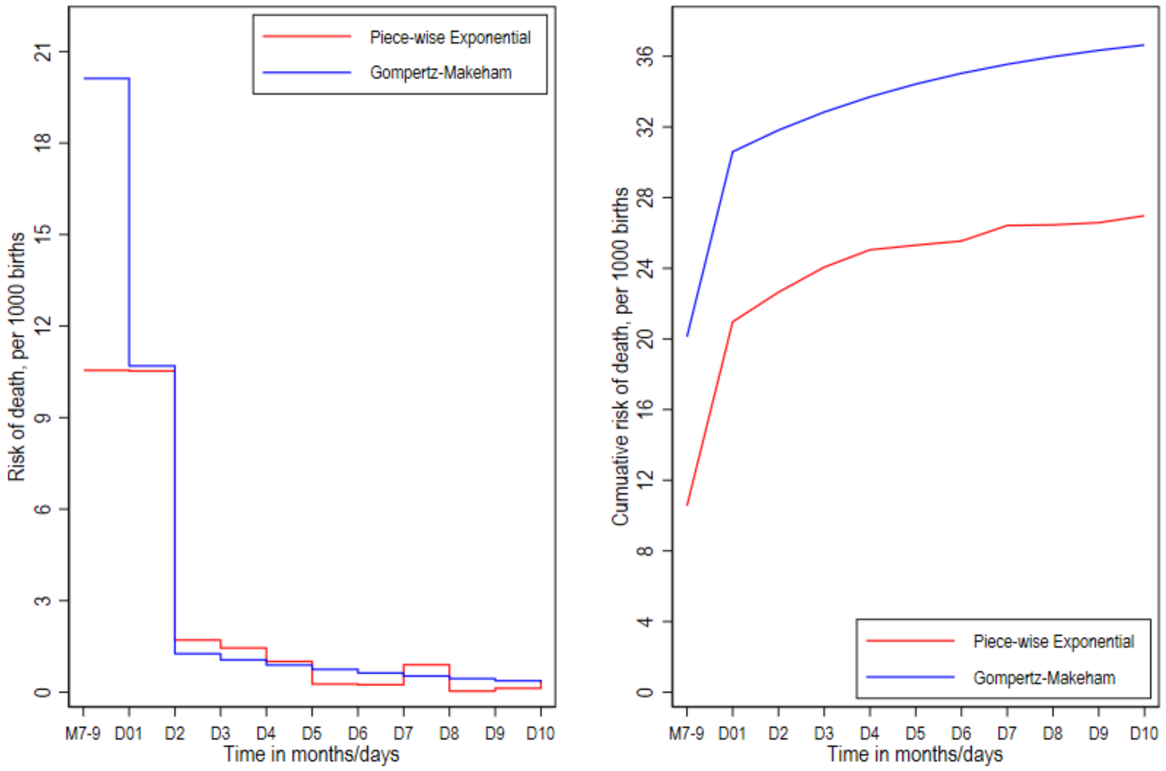

Figure S2: Indonesia DHS-2017

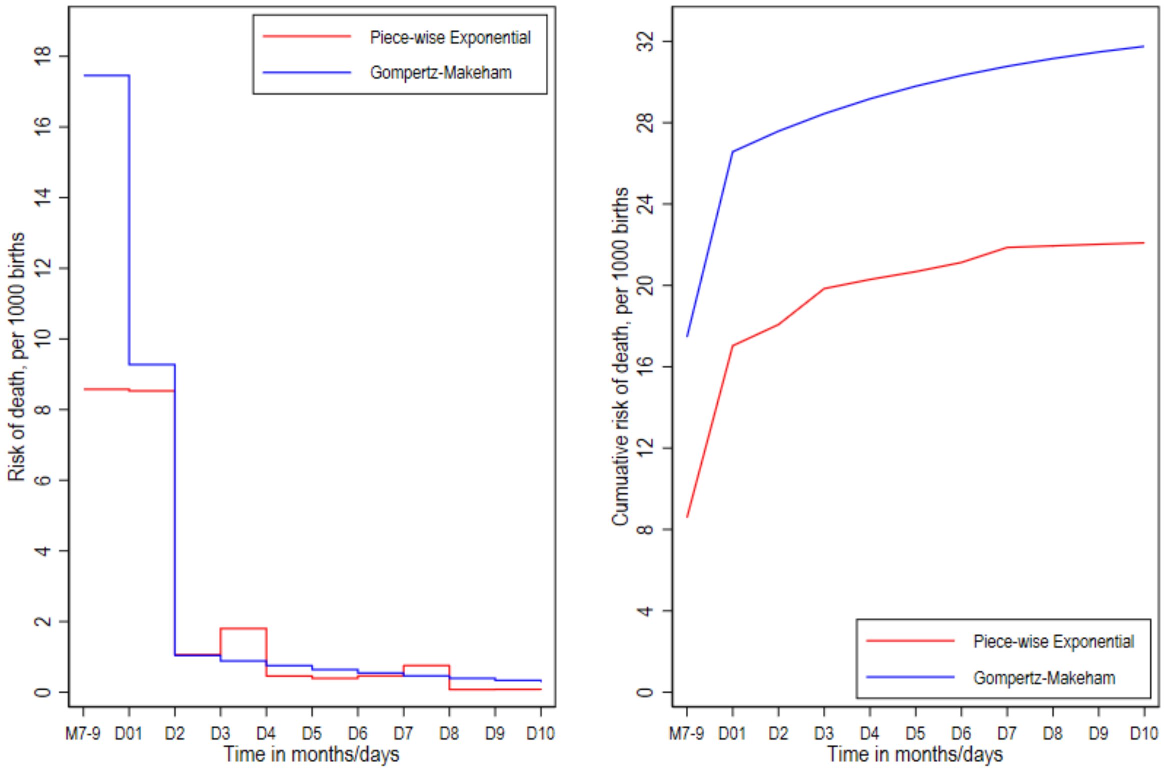

Figure S2: Maldives DHS-2009

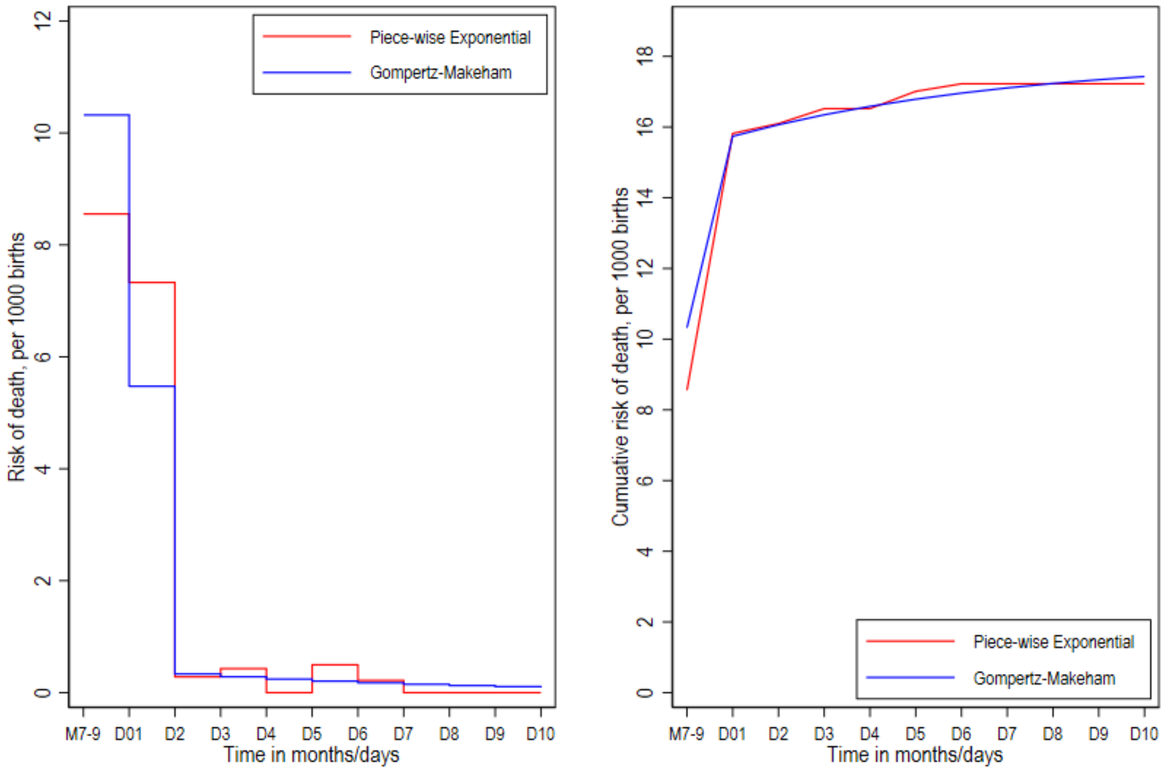

Figure S2: Myanmar DHS-2015/16

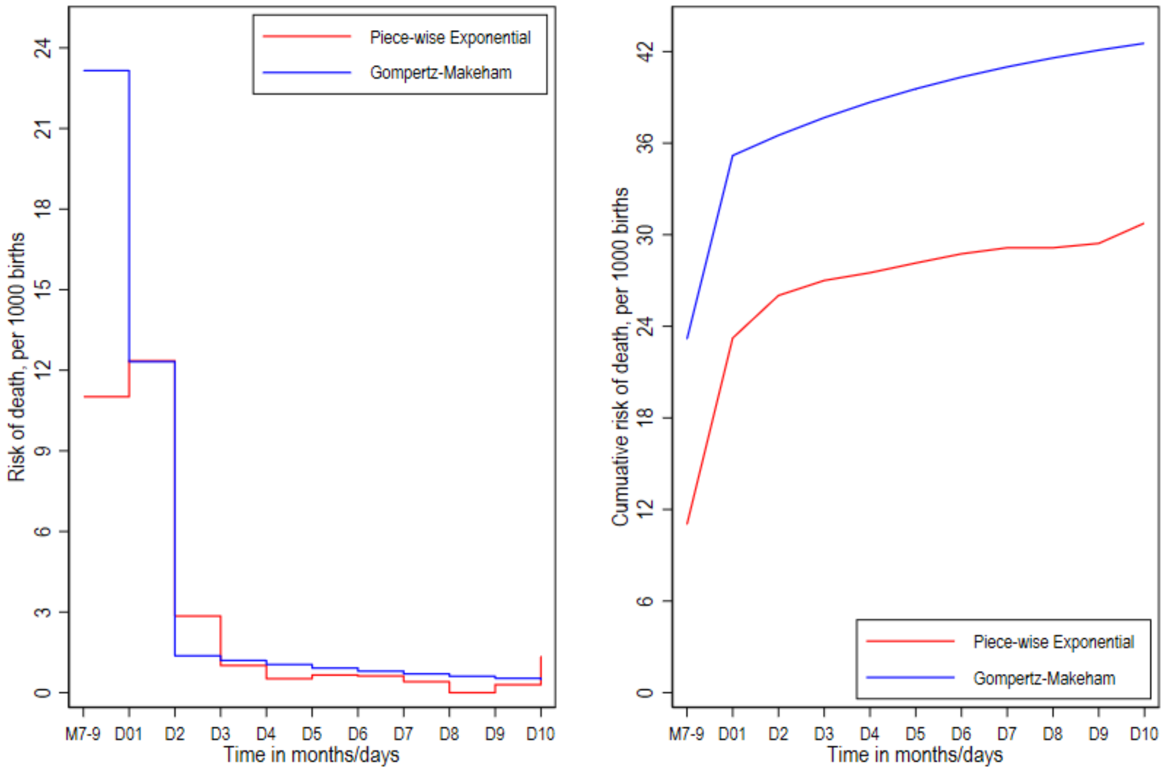

Figure S2: Nepal DHS-2006

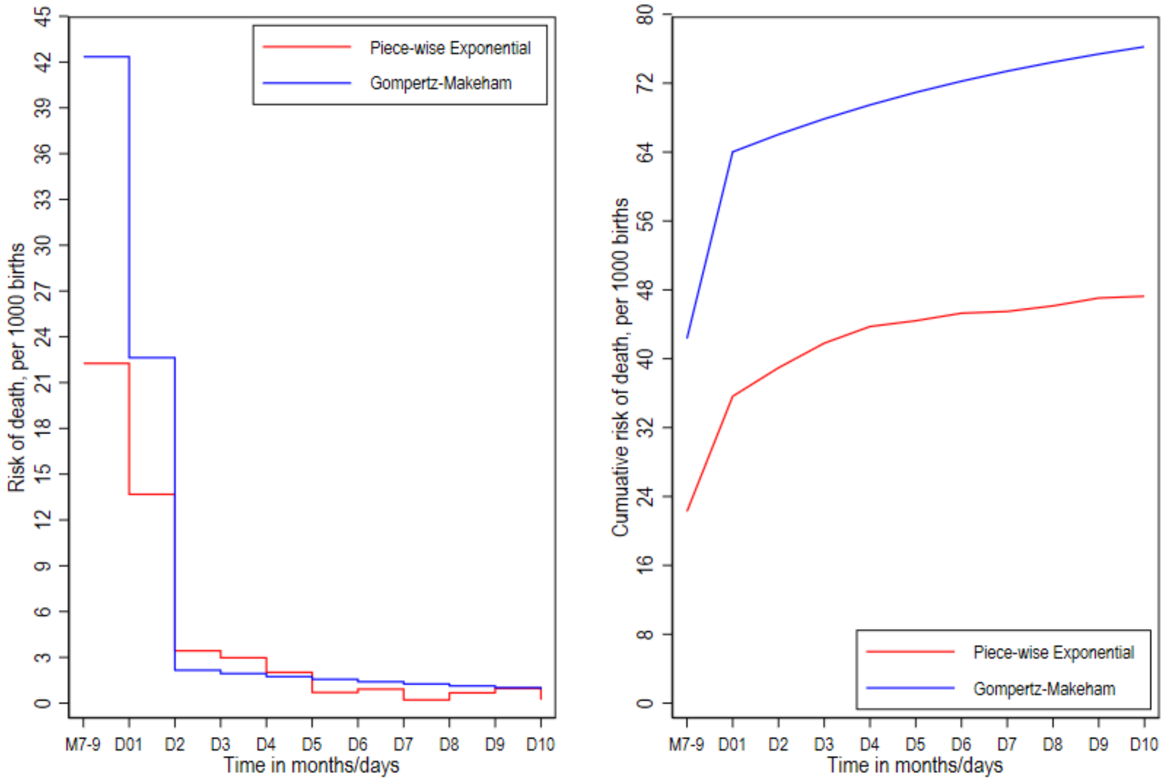

Figure S2: Nepal DHS-2011

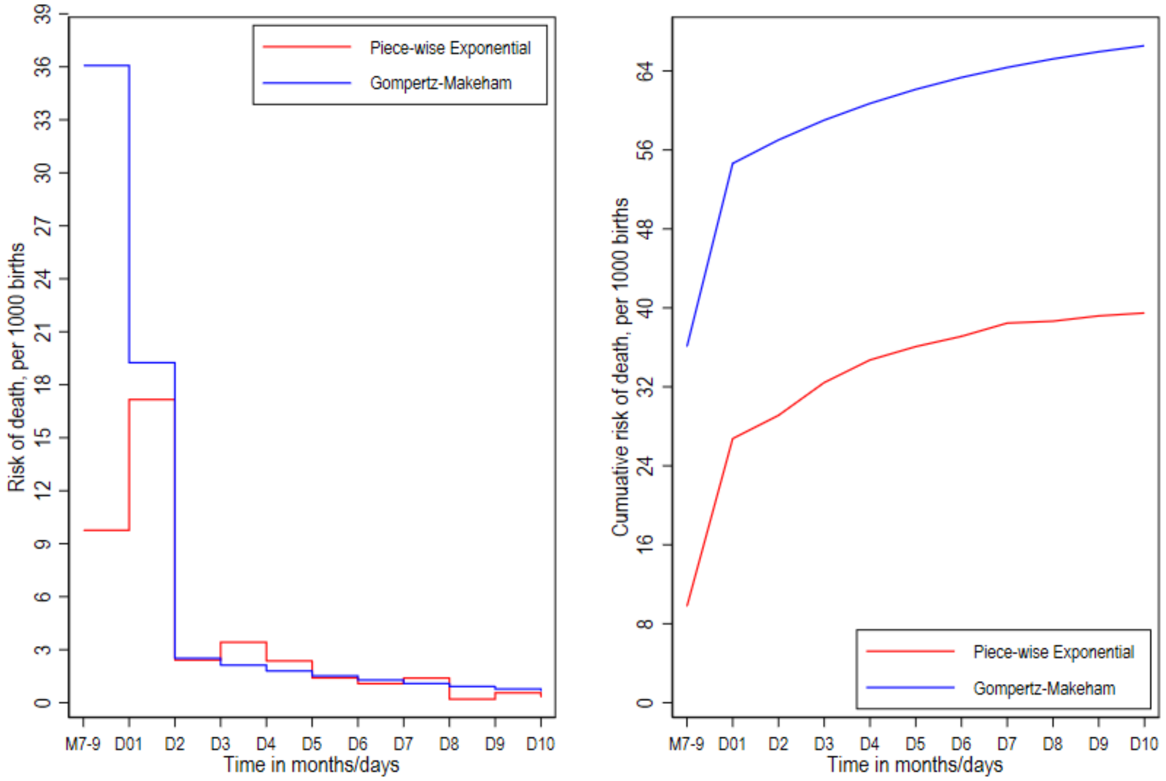

Figure S2: Nepal DHS-2016

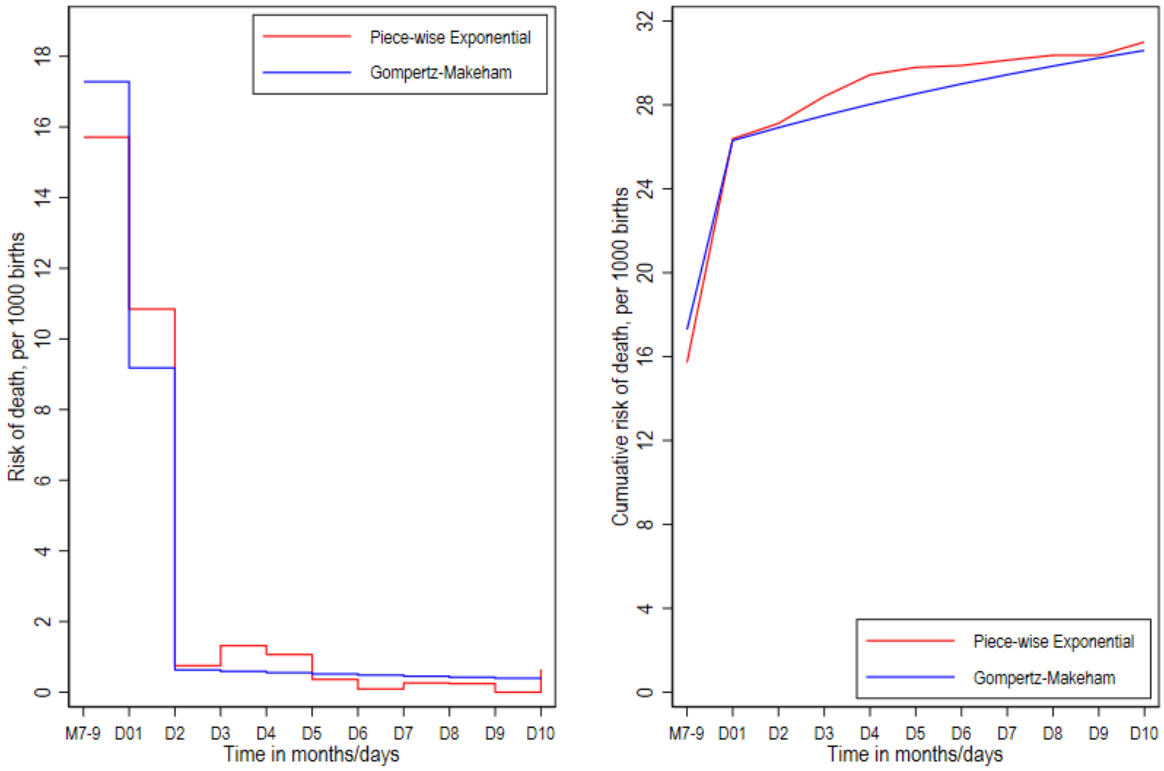

Figure S2: Pakistan DHS-2012/13

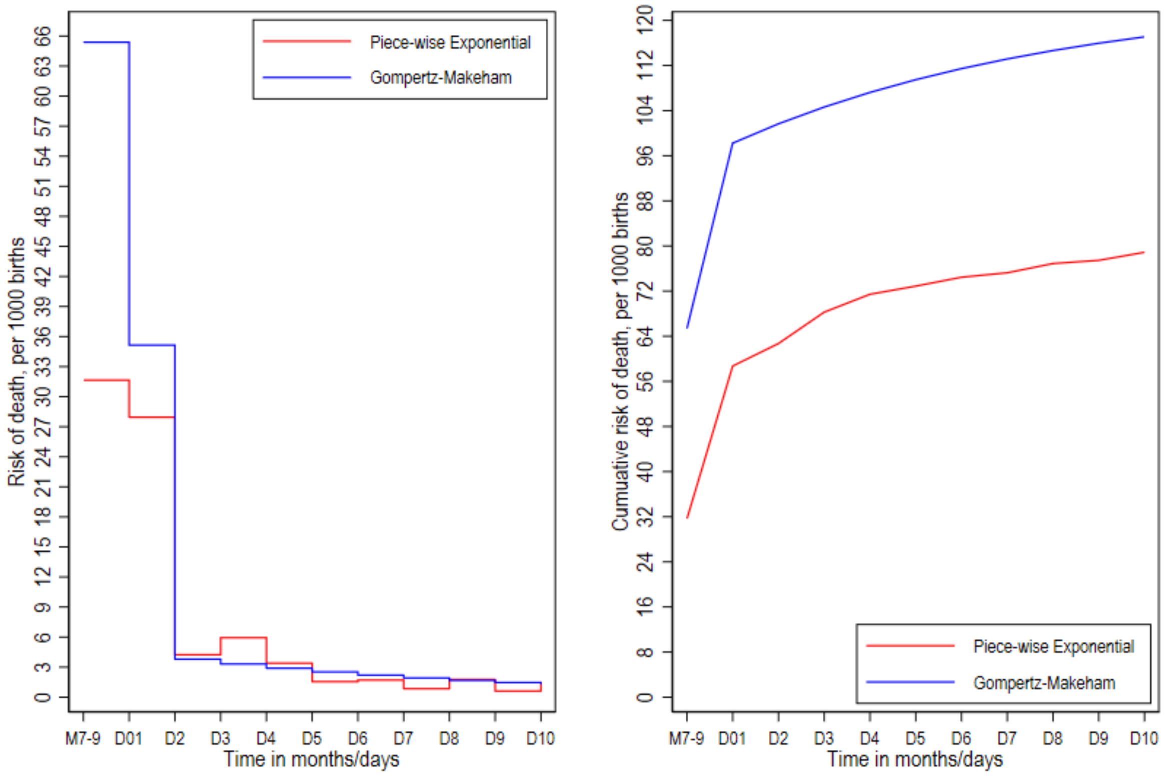

Figure S2: Pakistan DHS-2017/18

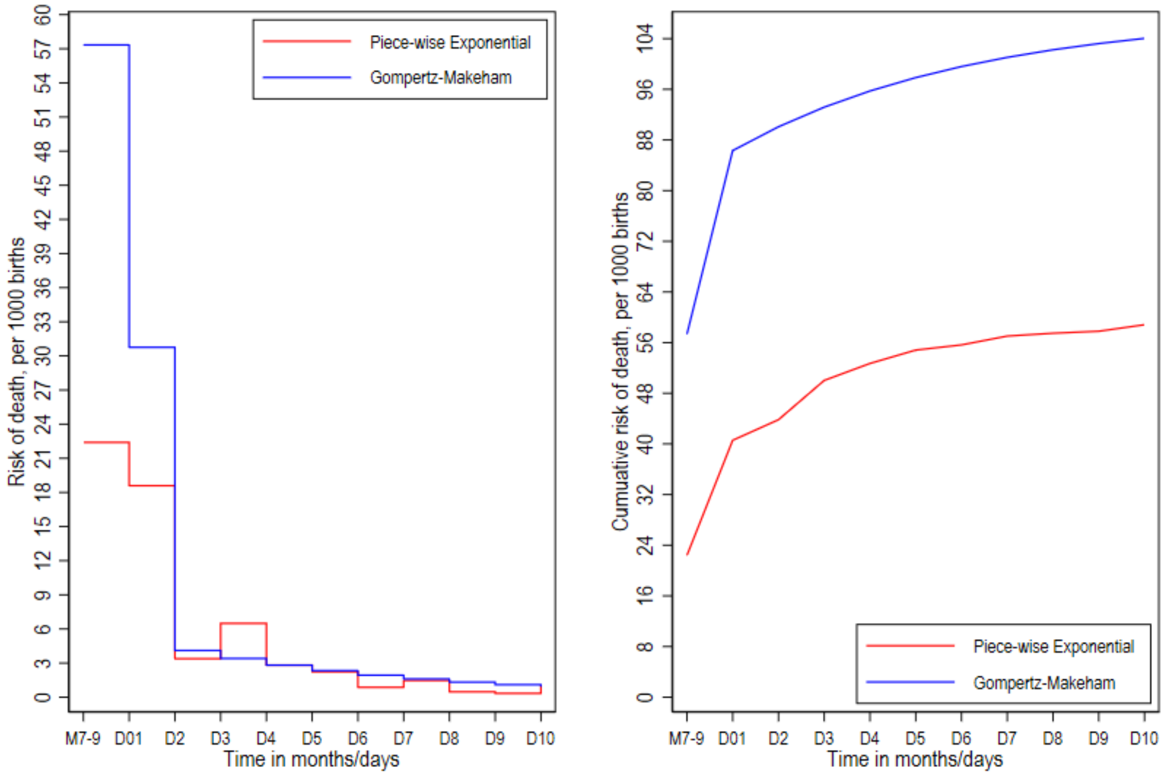

Figure S2: Philippines DHS-1993

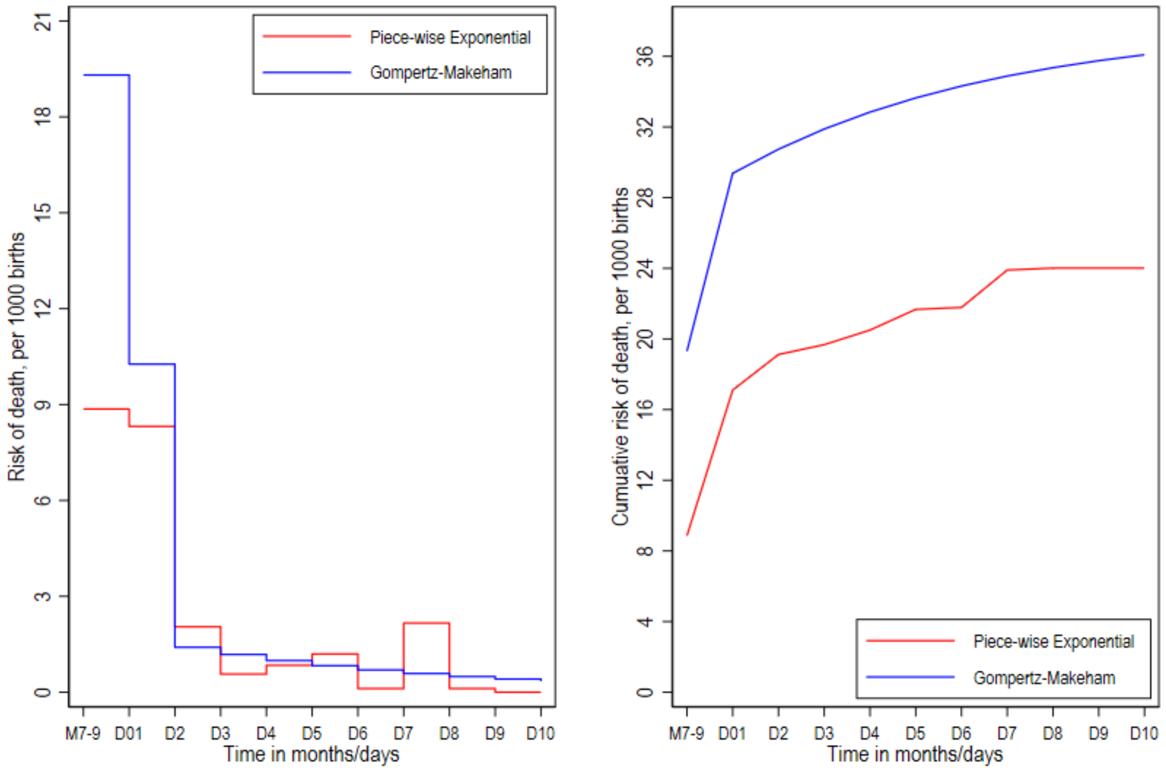

Figure S2: Philippines DHS-1998

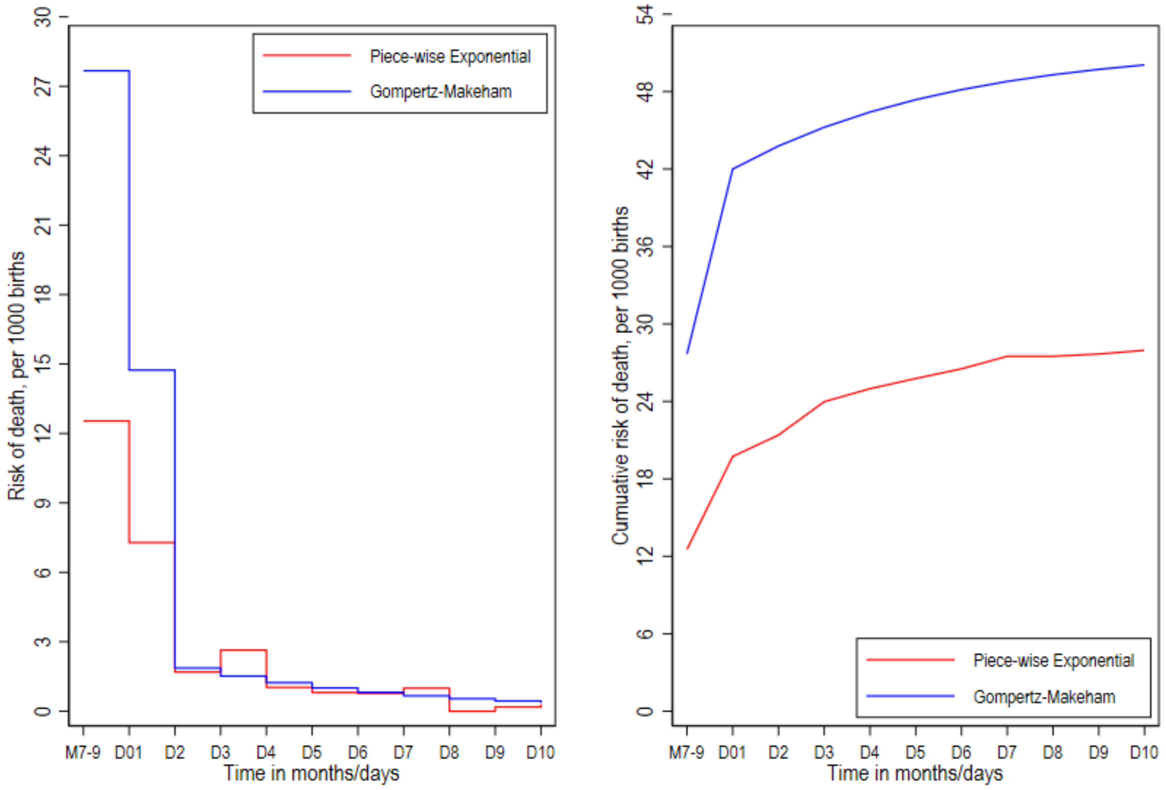

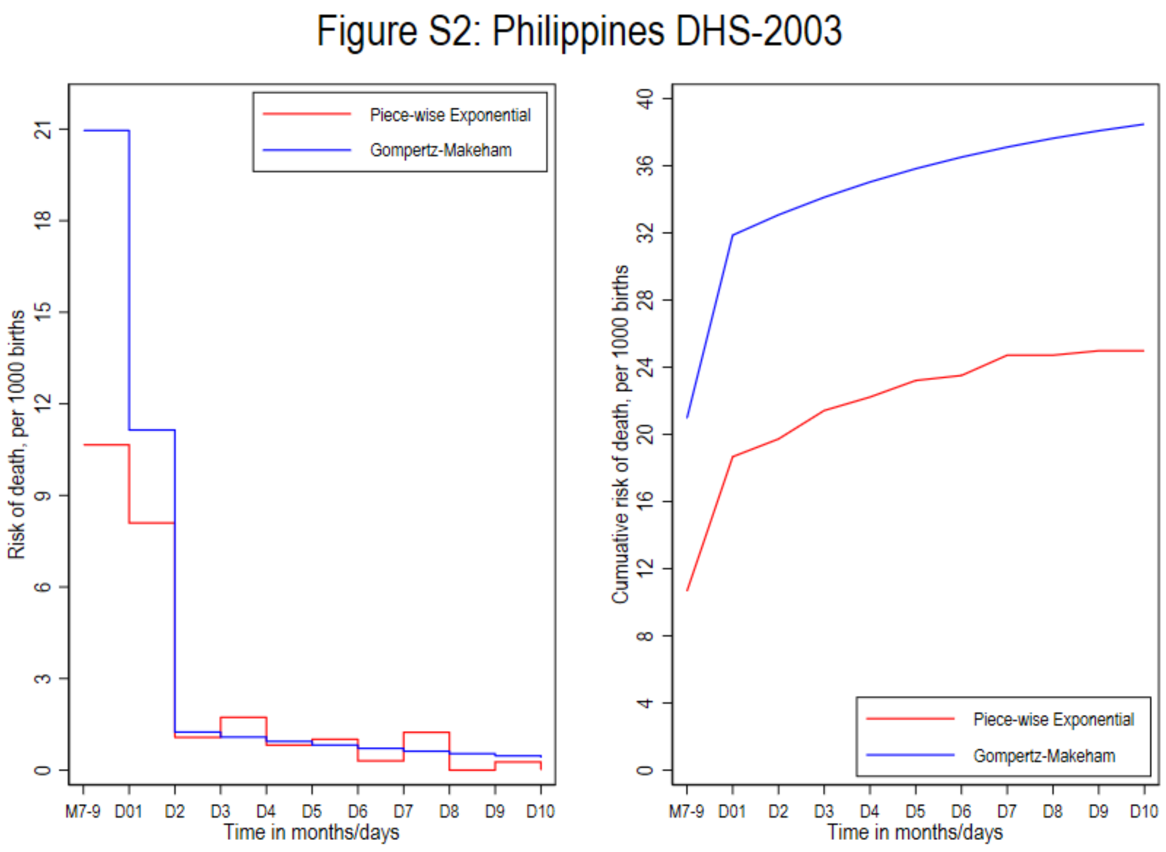
Figure S2: Philippines DHS-2003
Piece-wise Exponential
Gompertz-Makeham
Risk of death, per 1000 births
Cumuative risk of death, per 1000 births
0
3
6
9
12
15
18
21
0
4
8
12
16
20
24
28
32
36
40
M7-9
D01
D2
D3
D4
D5
D6
D7
D8
D9
D10
Time in months/days

Figure S2: Timor-Leste DHS-2009/10

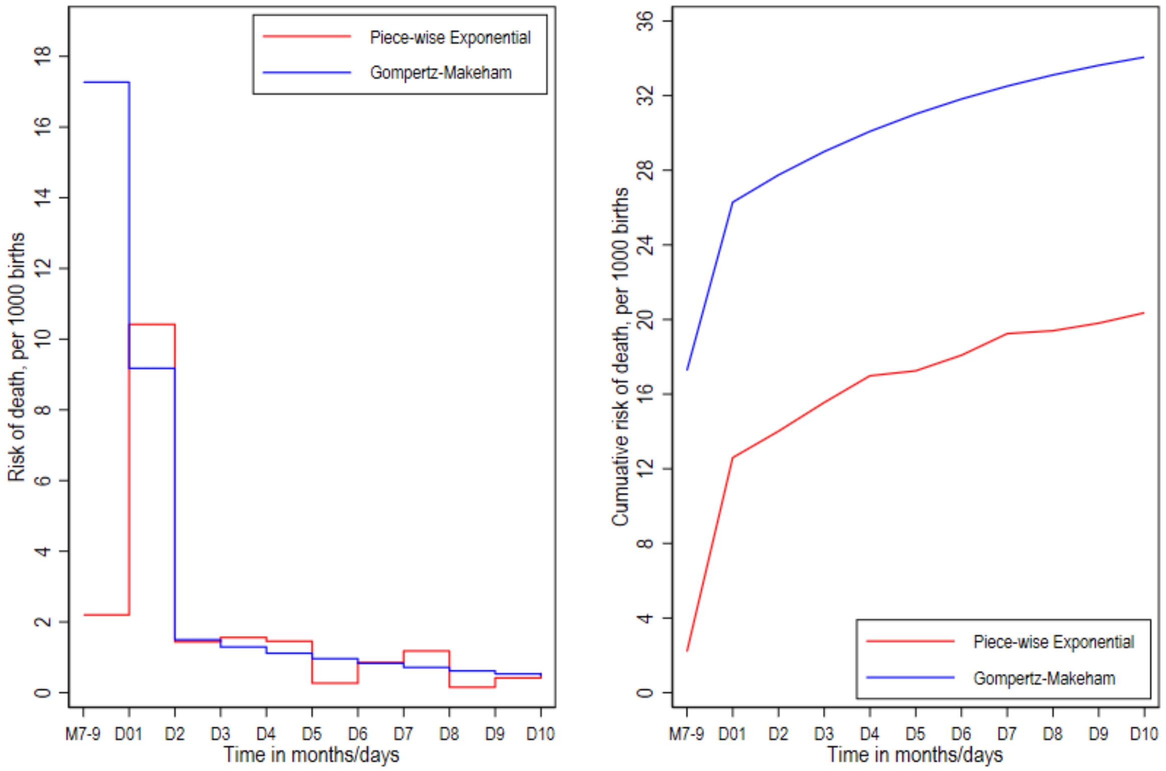

Figure S2: Bolivia DHS-1993/94

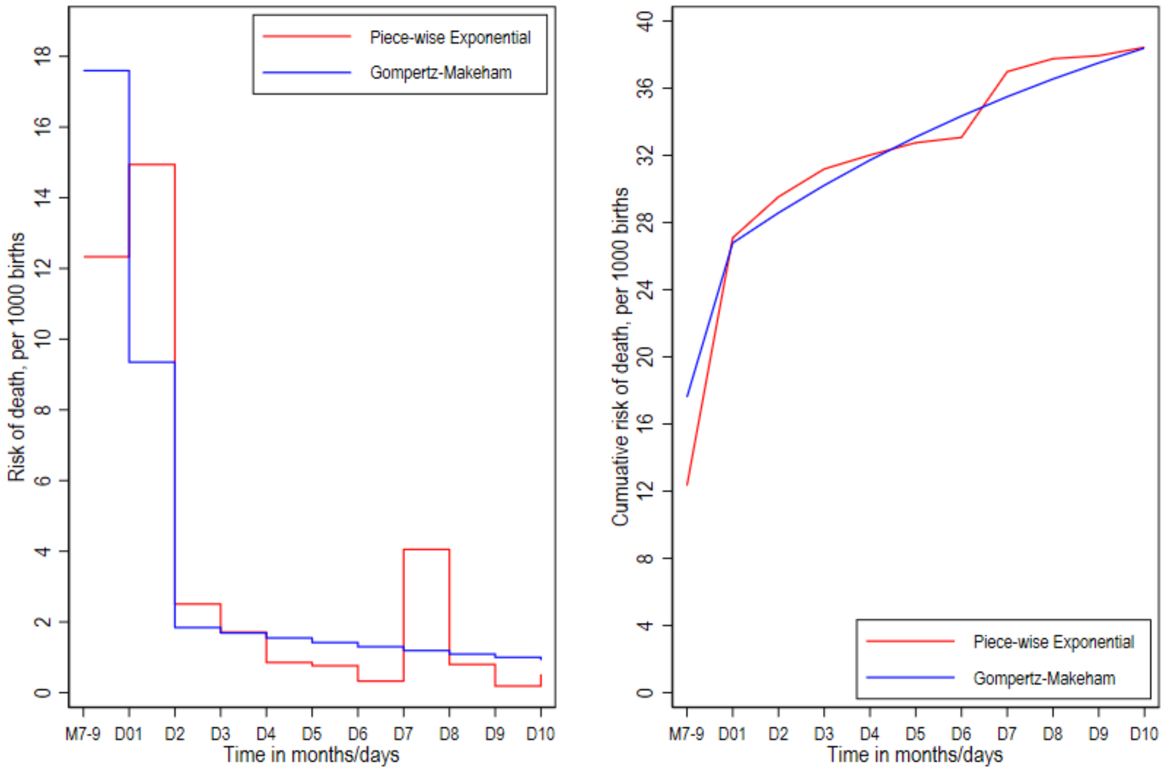

Figure S2: Bolivia DHS-2003/4

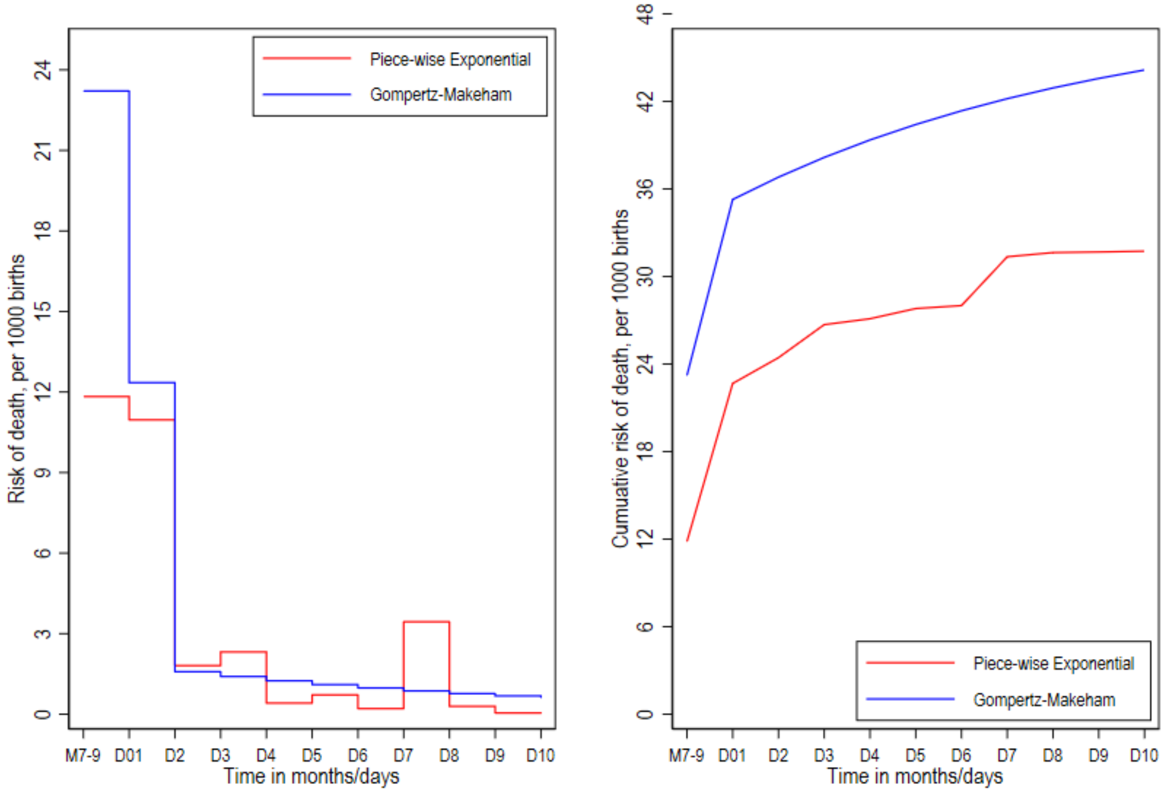

Figure S2: Bolivia DHS-2008

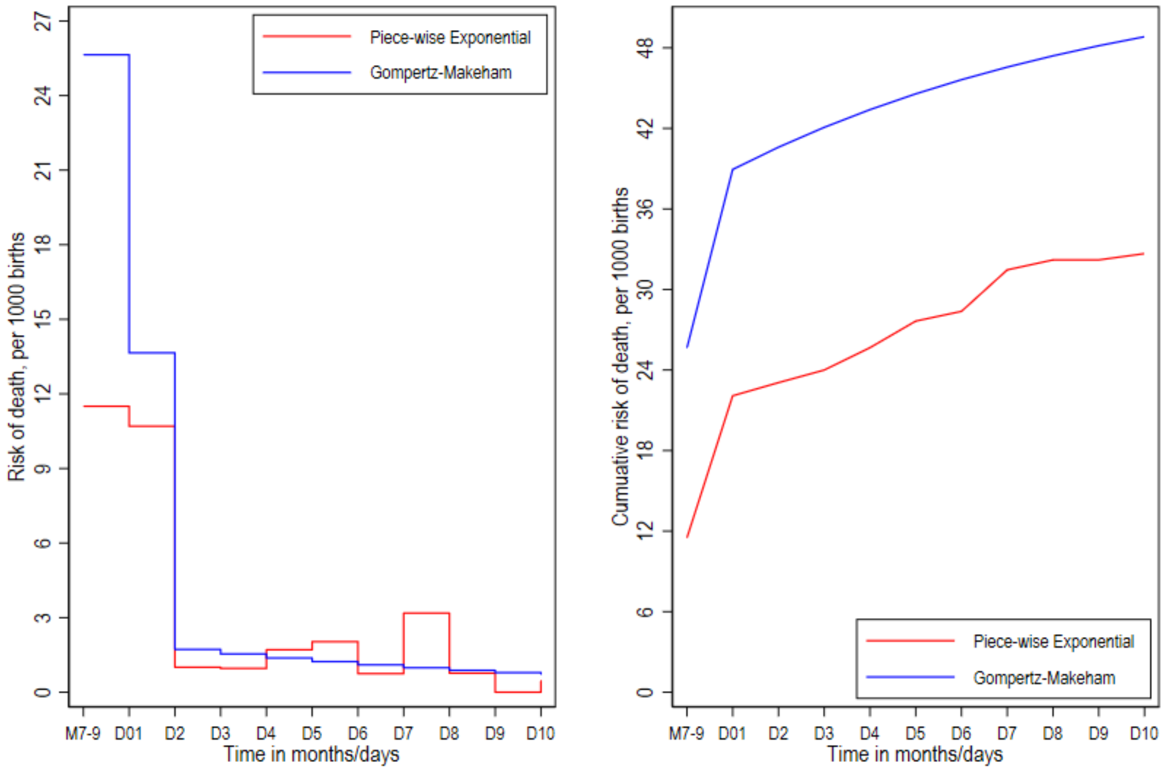

Figure S2: Brazil DHS-1991/92

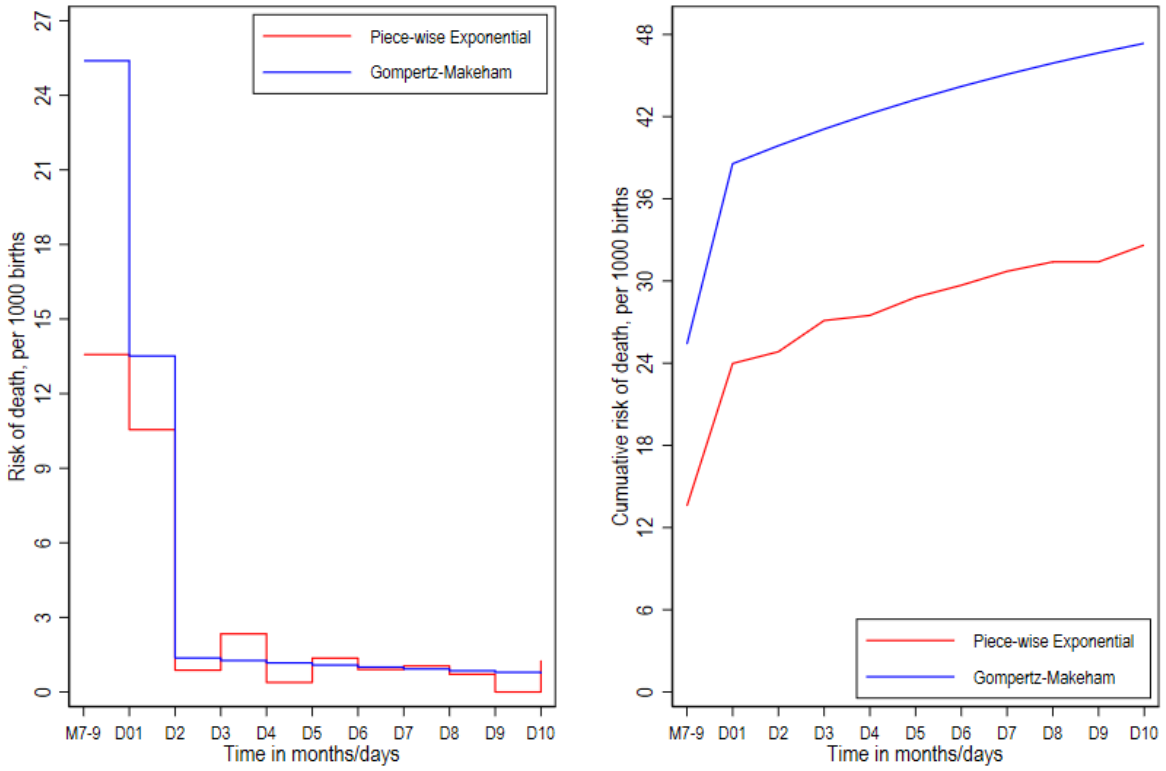

Figure S2: Brazil DHS-1996

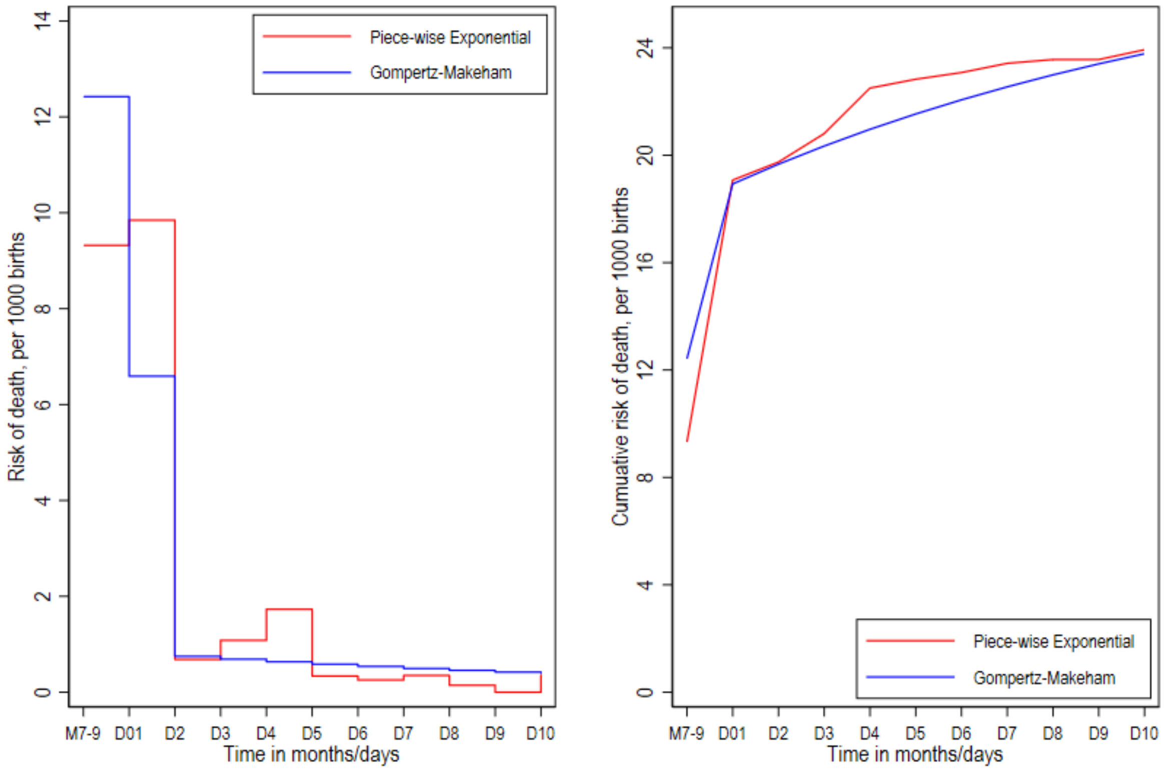

Figure S2: Colombia DHS-1995

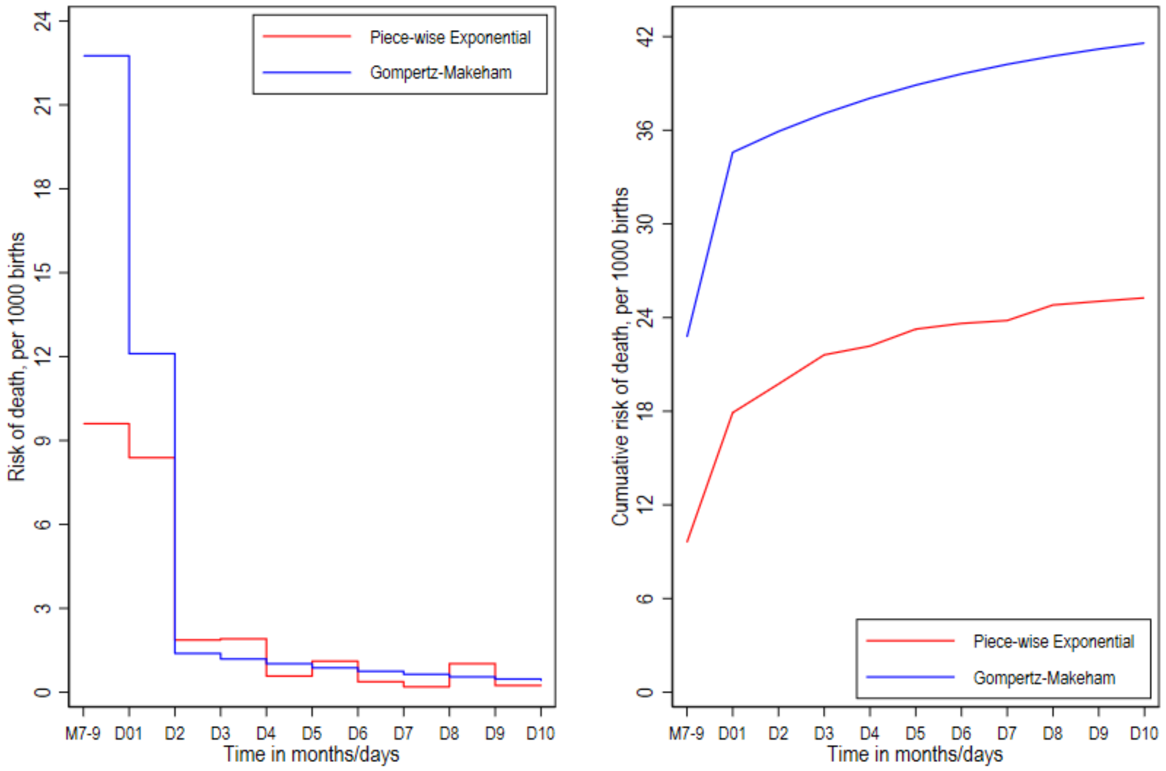

Figure S2: Colombia DHS-2000

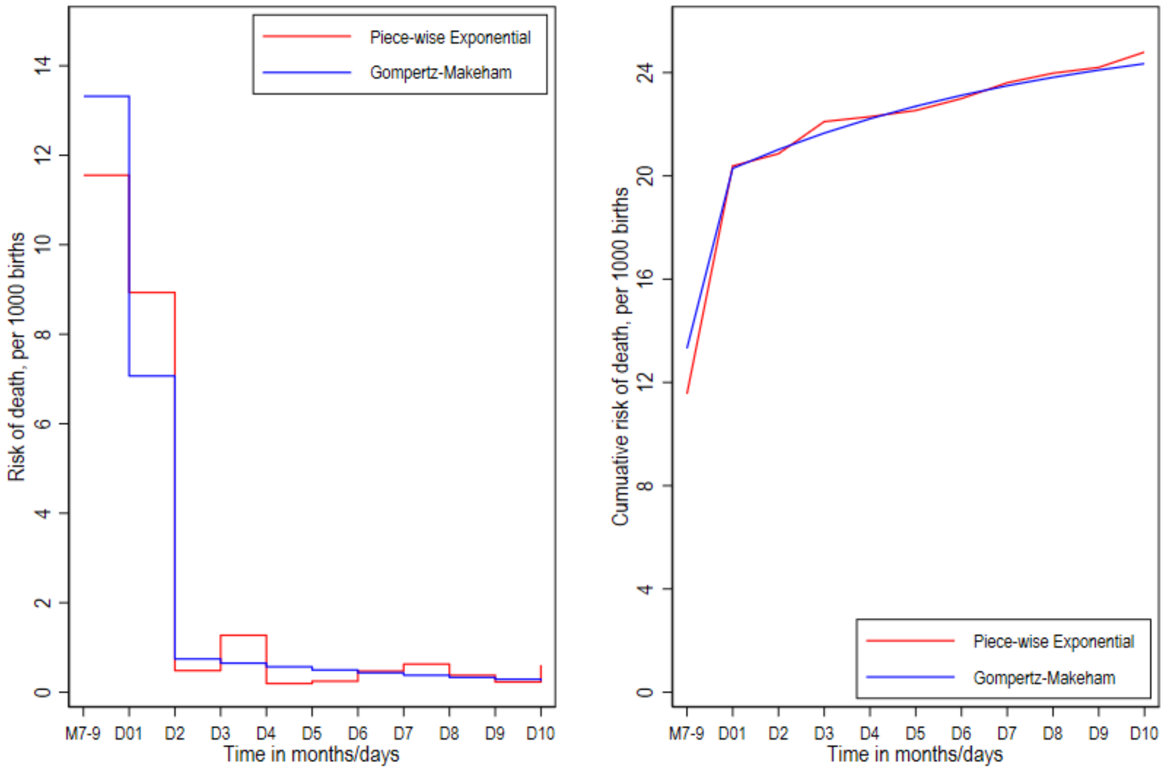

Figure S2: Colombia DHS-2004/5

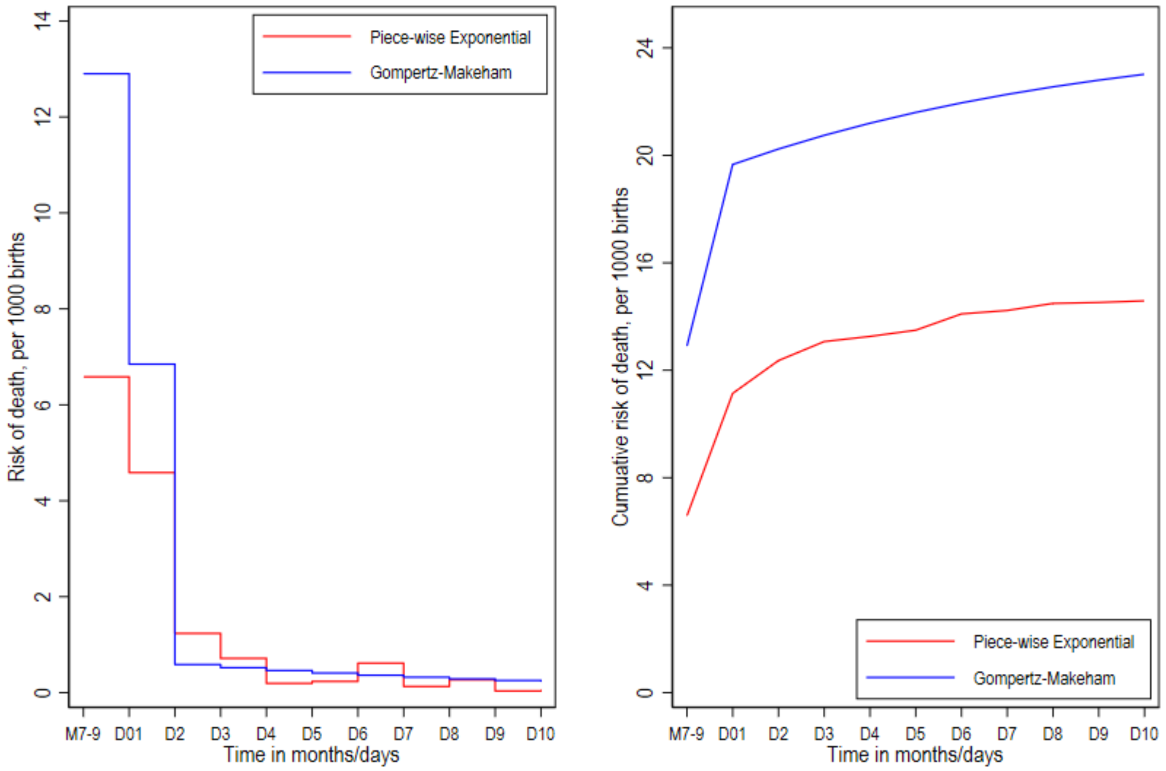

Figure S2: Colombia DHS-2009/10

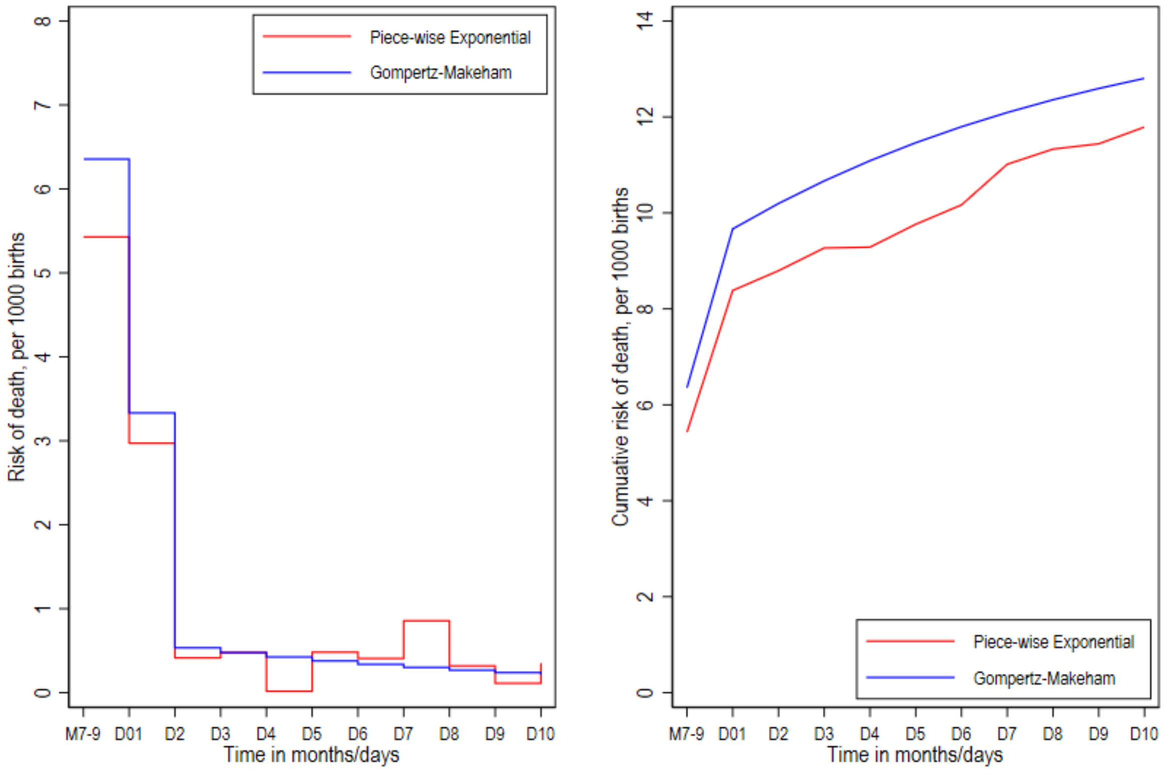

Figure S2: Colombia DHS-2015/16

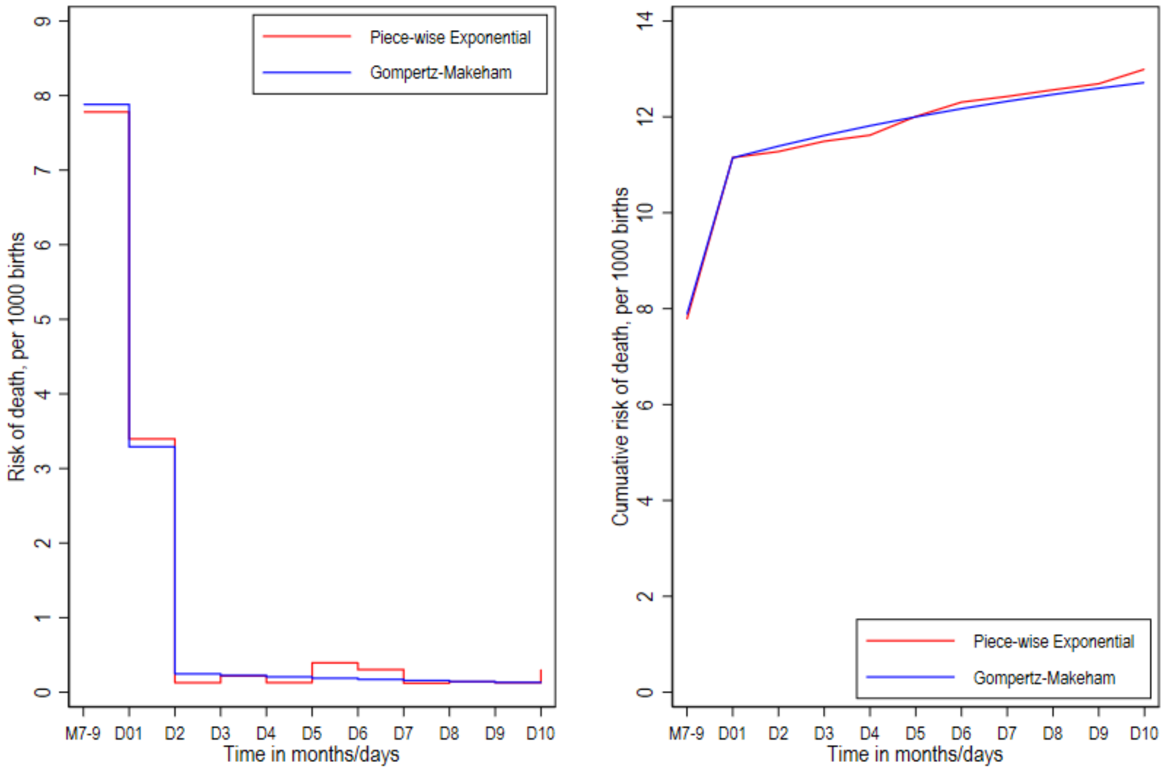

Figure S2: Dominican Republic DHS-1991

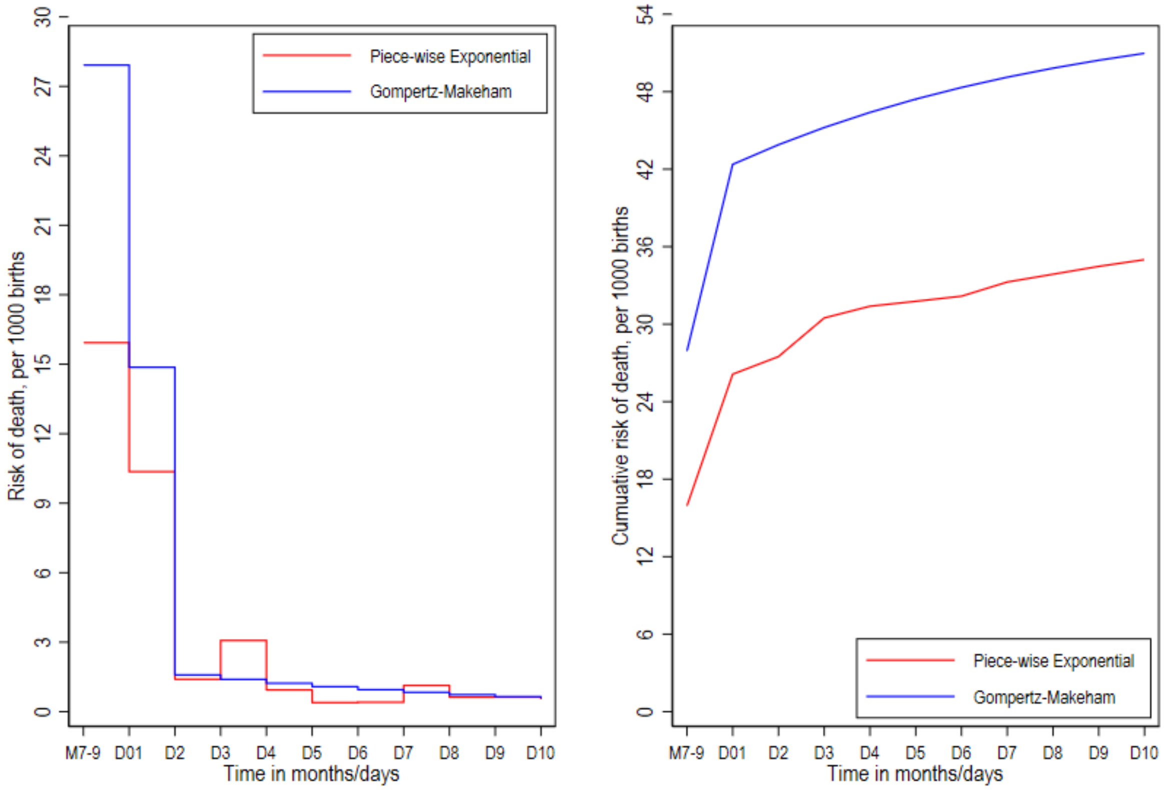

Figure S2: Dominican Republic DHS-1996

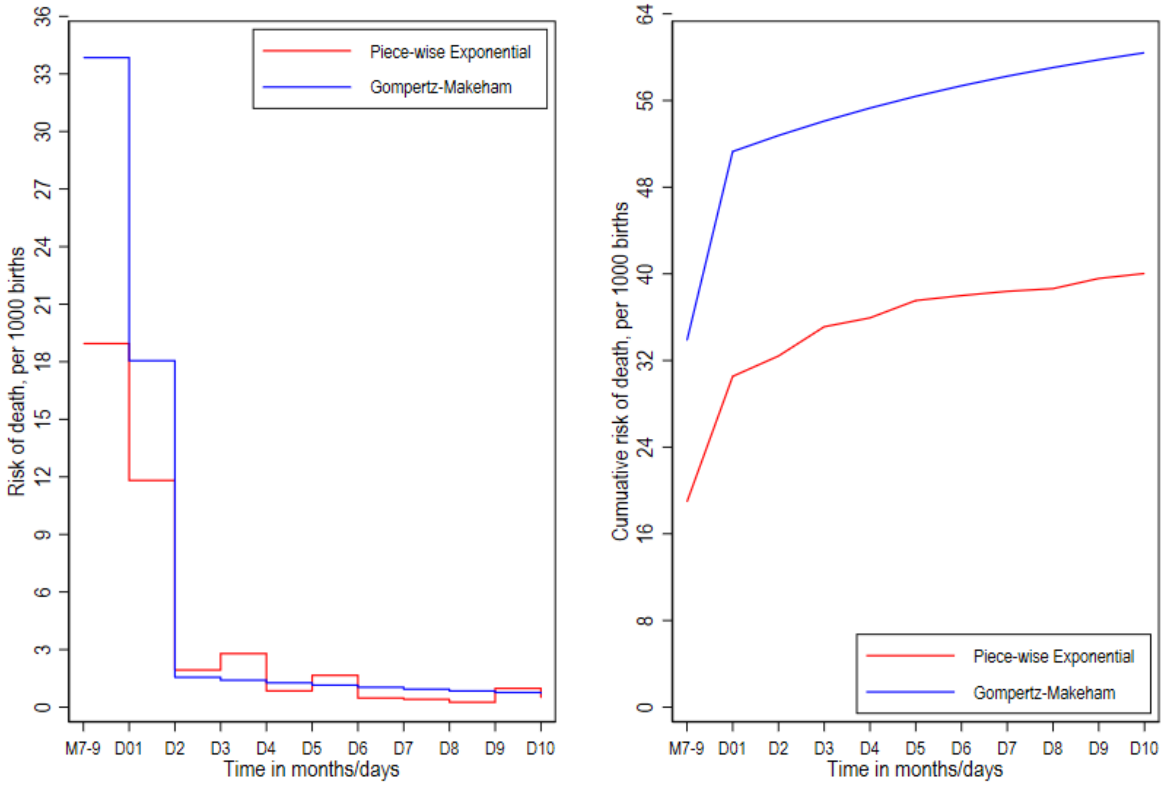

Figure S2: Dominican Republic DHS-2002

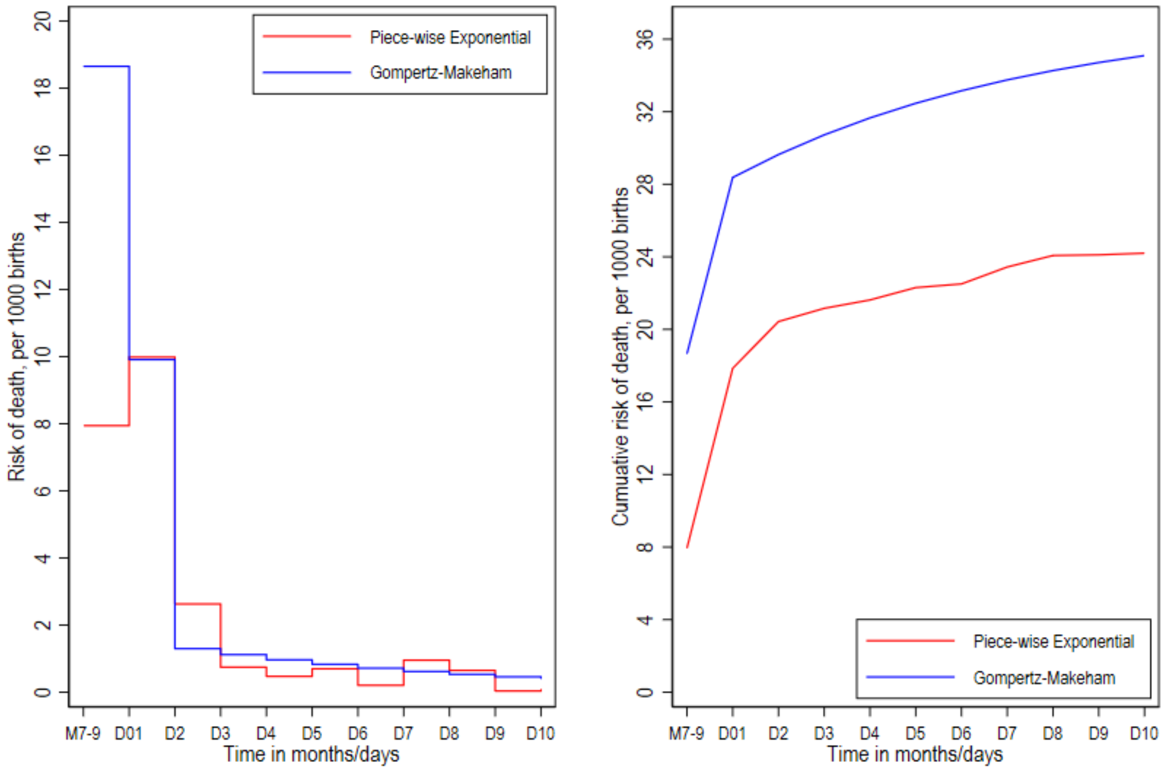

Figure S2: Guatemala DHS-1995

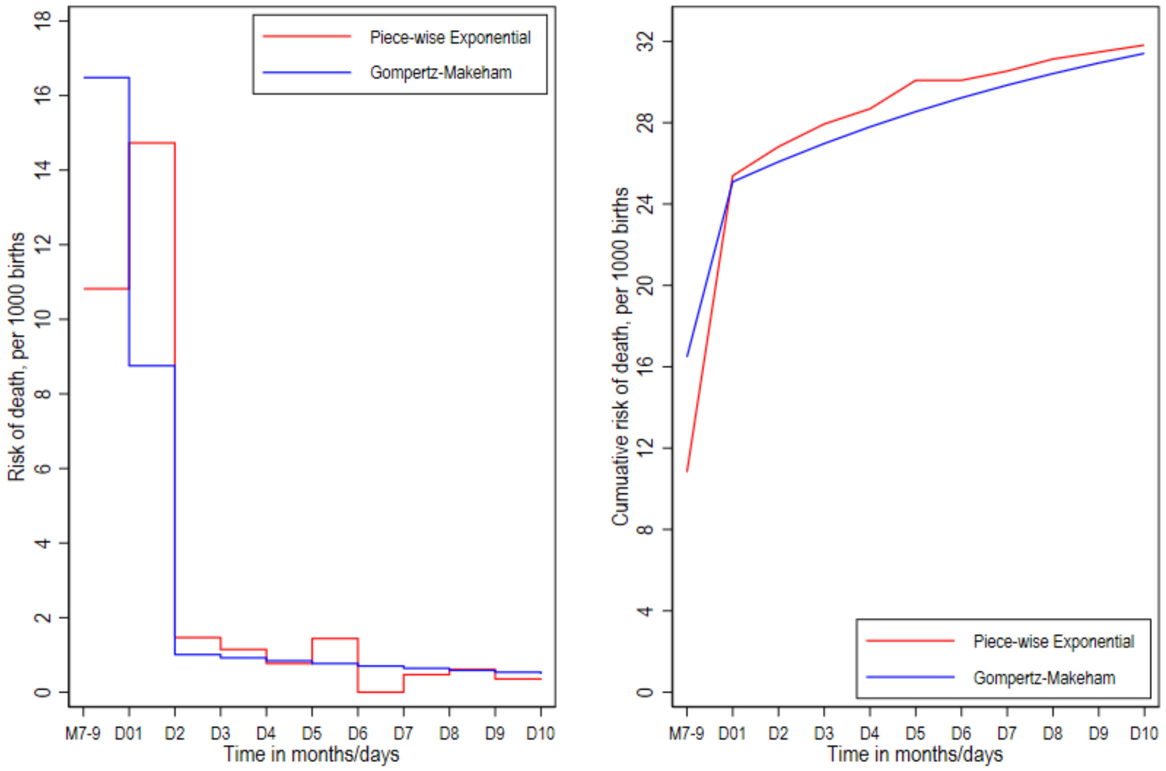

Figure S2: Guatemala DHS-1998/99

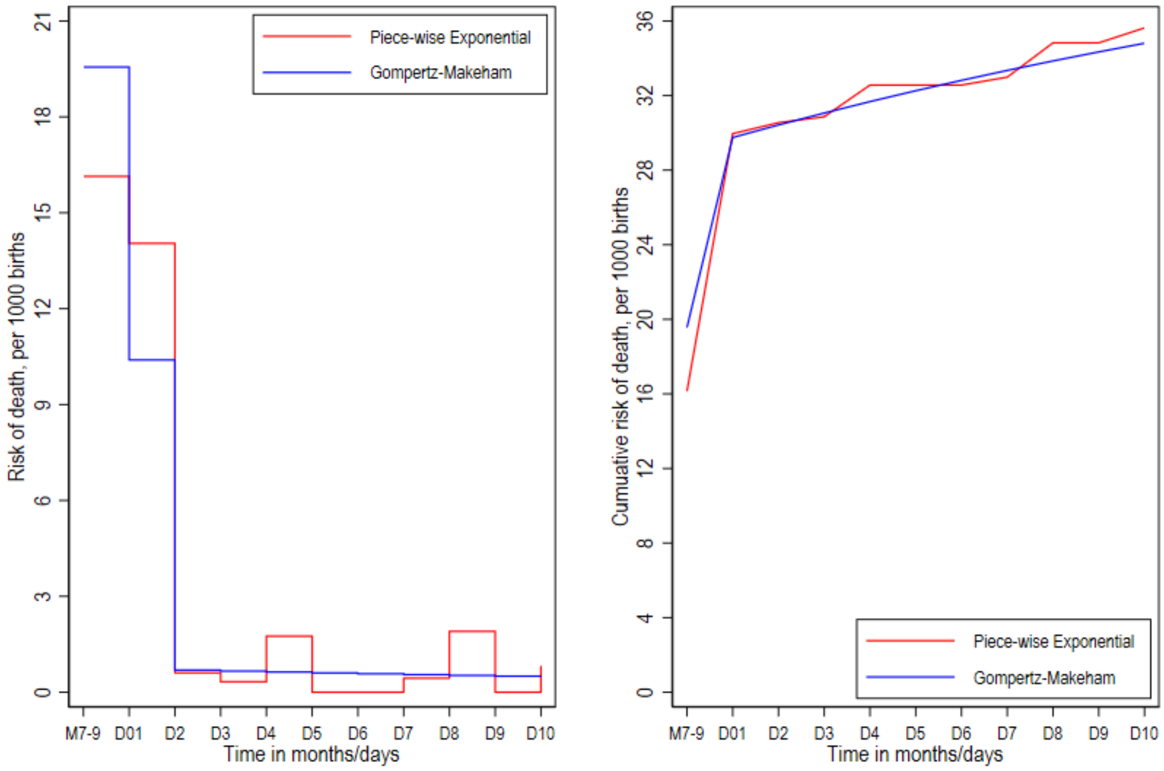

Figure S2: Guatemala DHS-2014/15

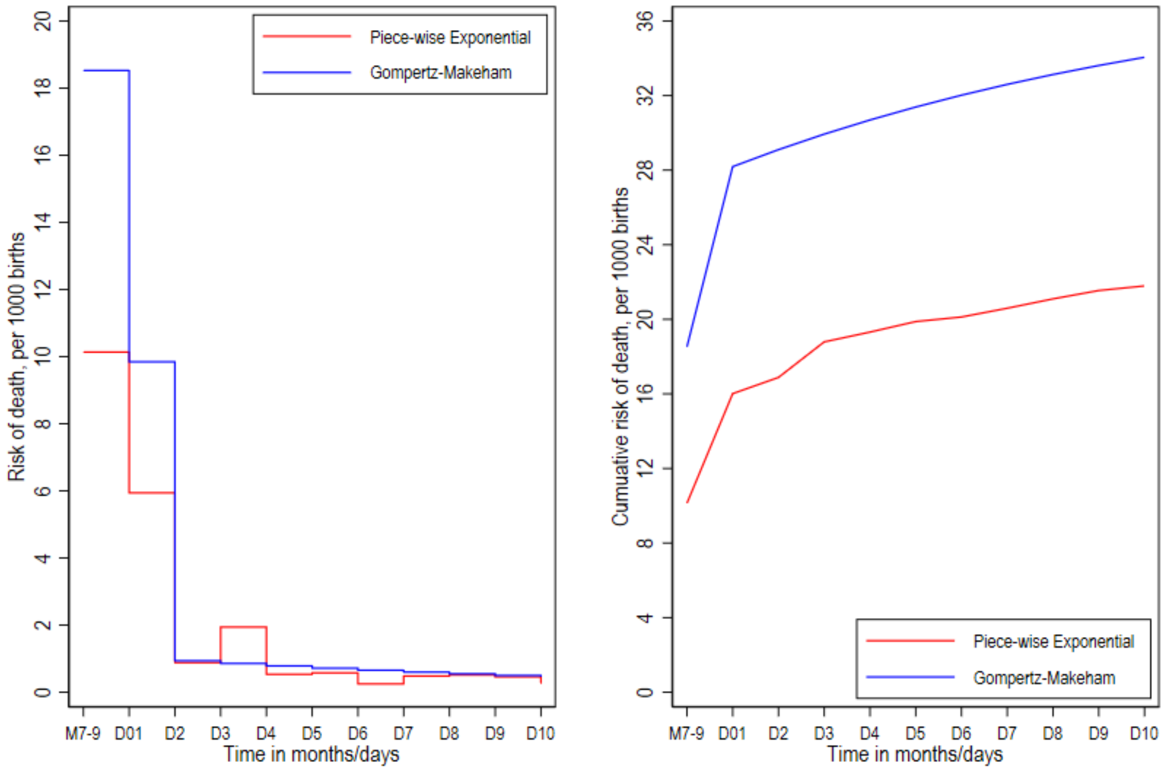

Figure S2: Honduras DHS-2005/6

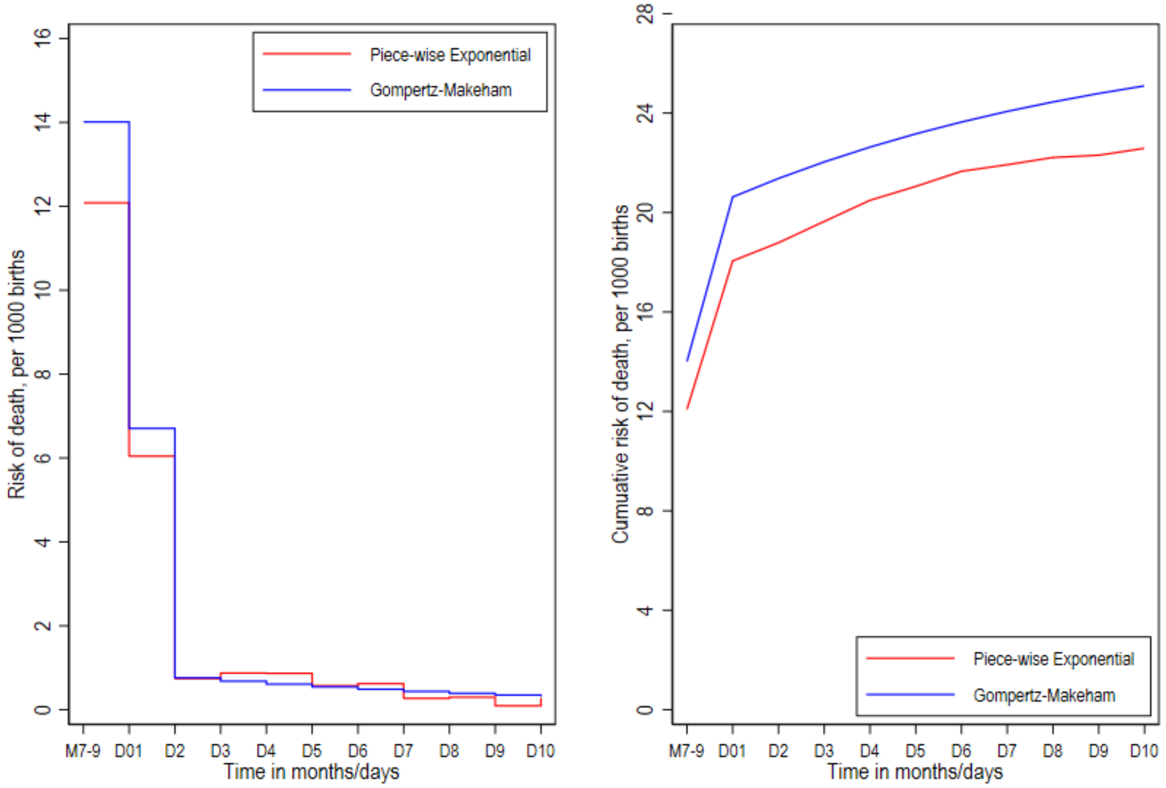

Figure S2: Honduras DHS-2011/12

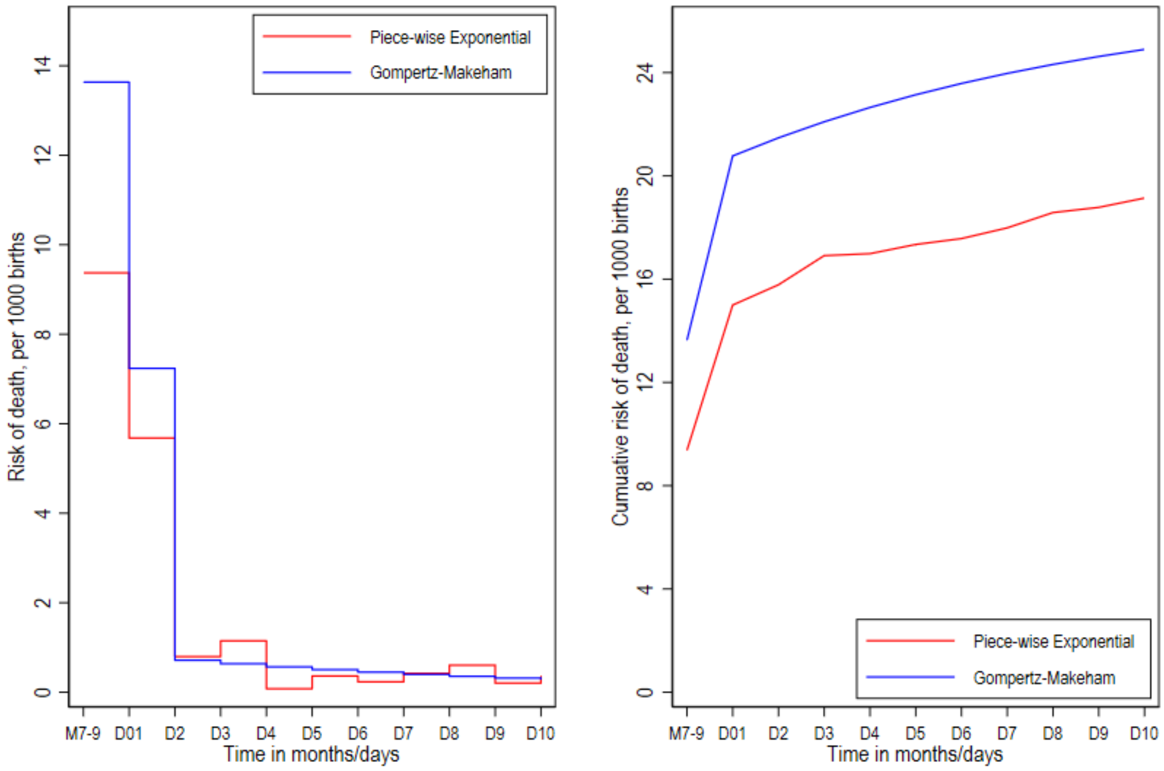

Figure S2: Nicaragua DHS-1997/98

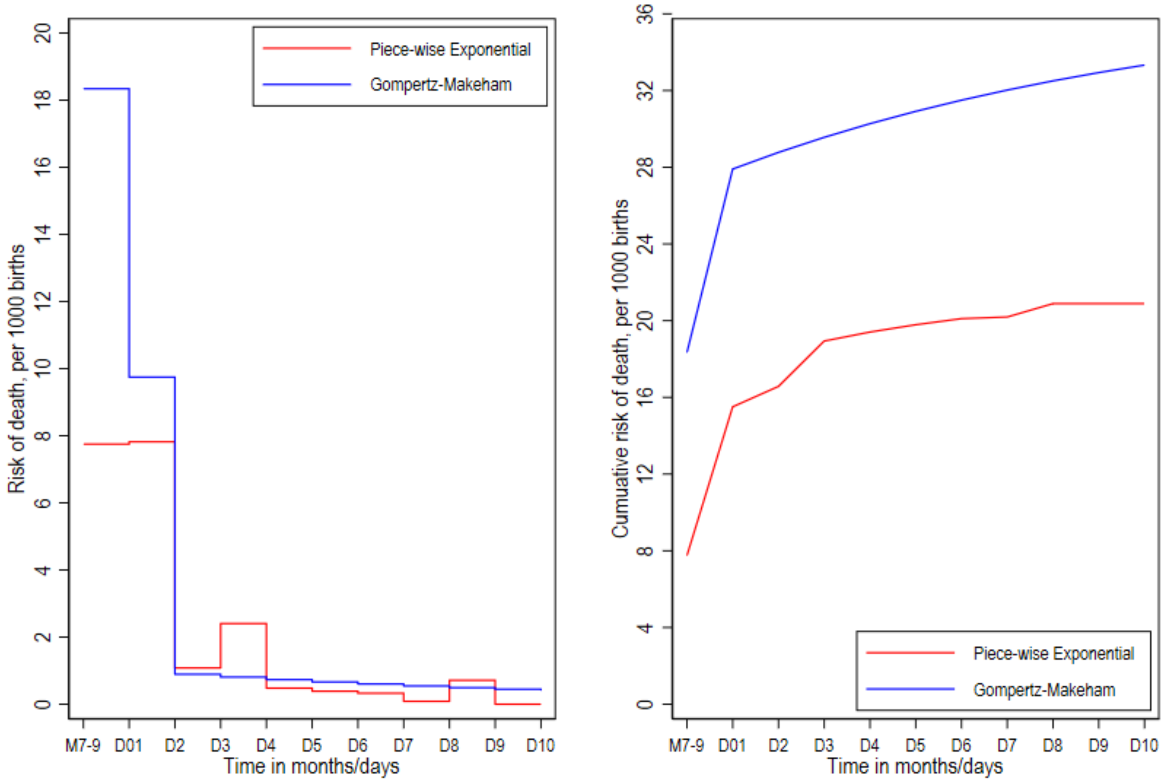

Figure S2: Nicaragua DHS-2001

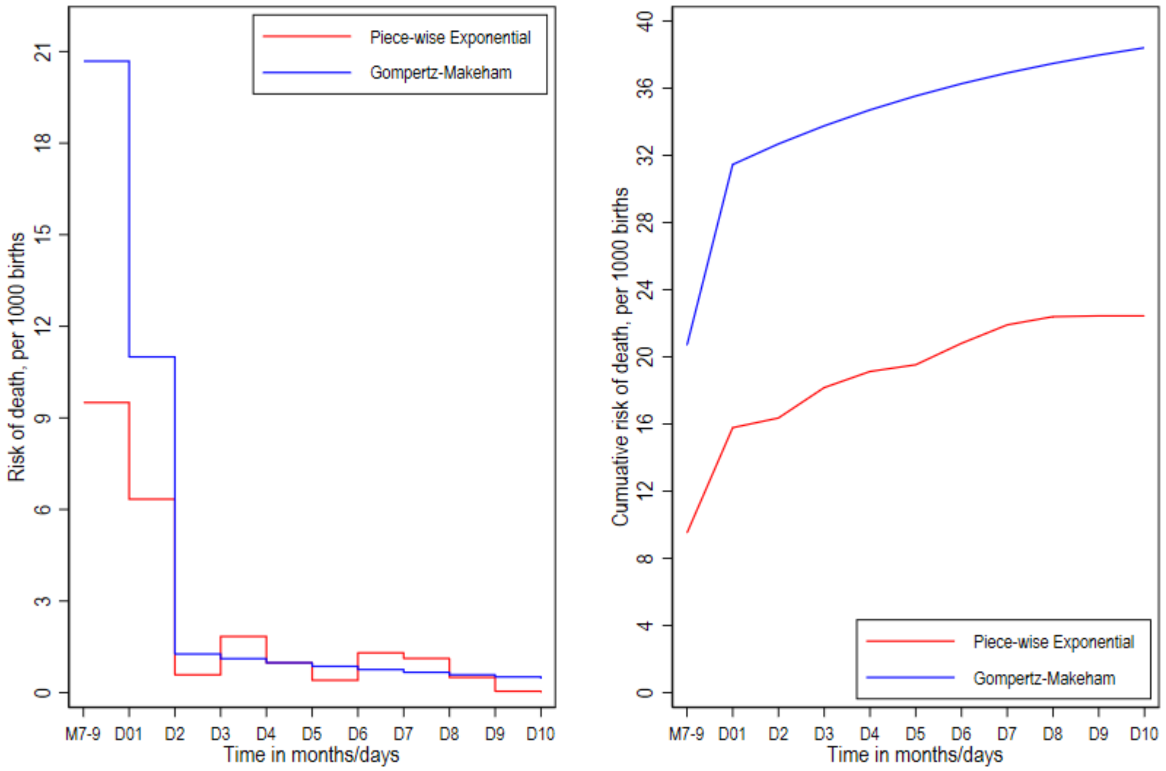

Figure S2: Paraguay DHS-1990

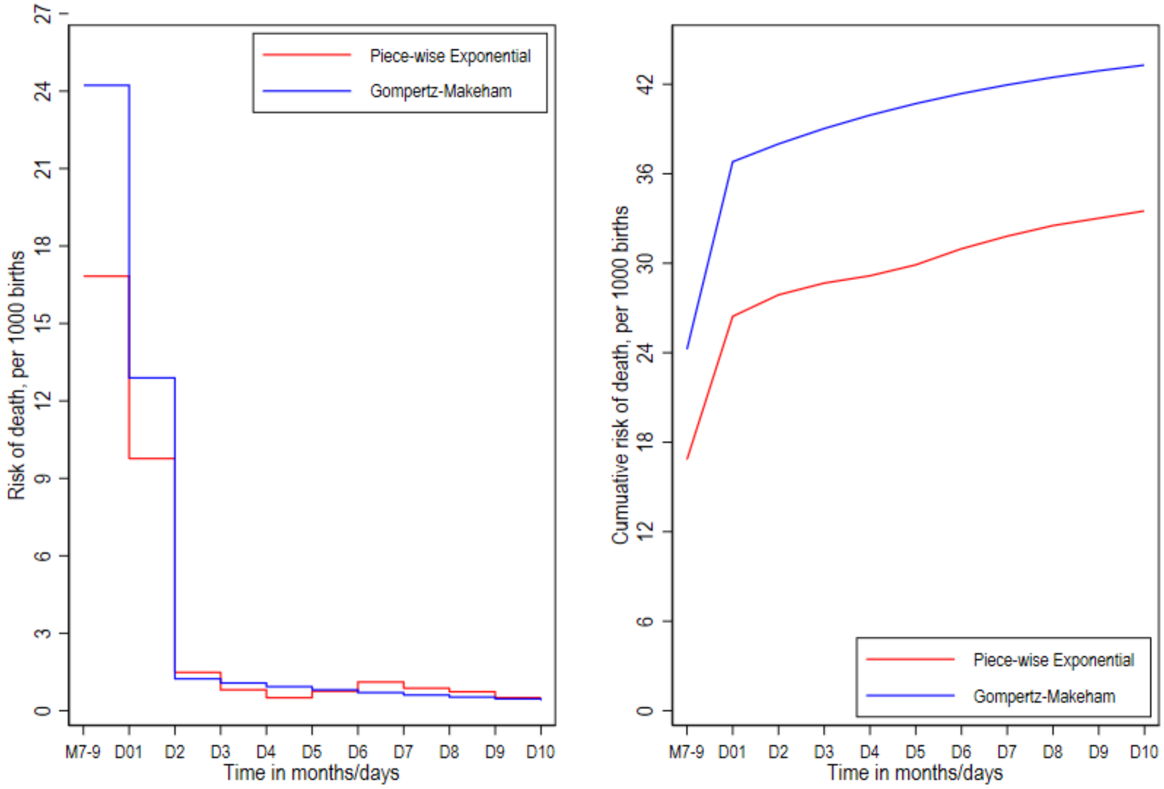

Figure S2: Peru DHS-1991/92

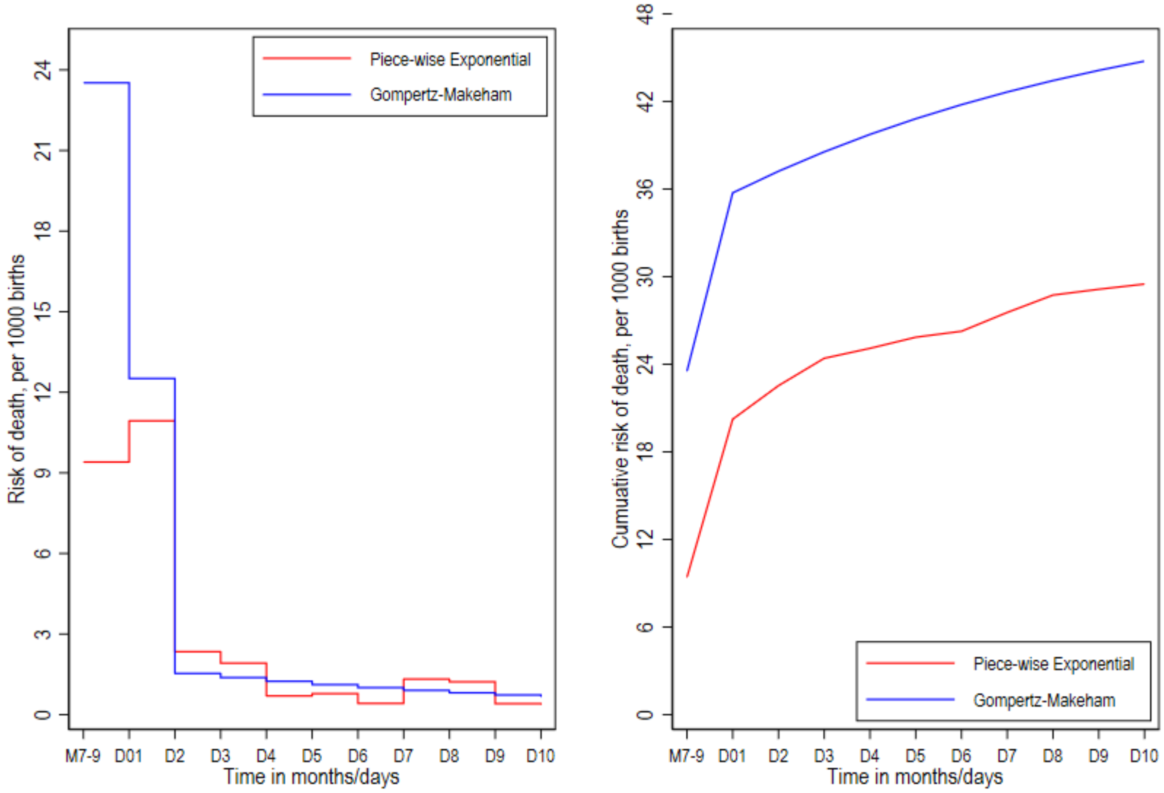

Figure S2: Peru DHS-1996

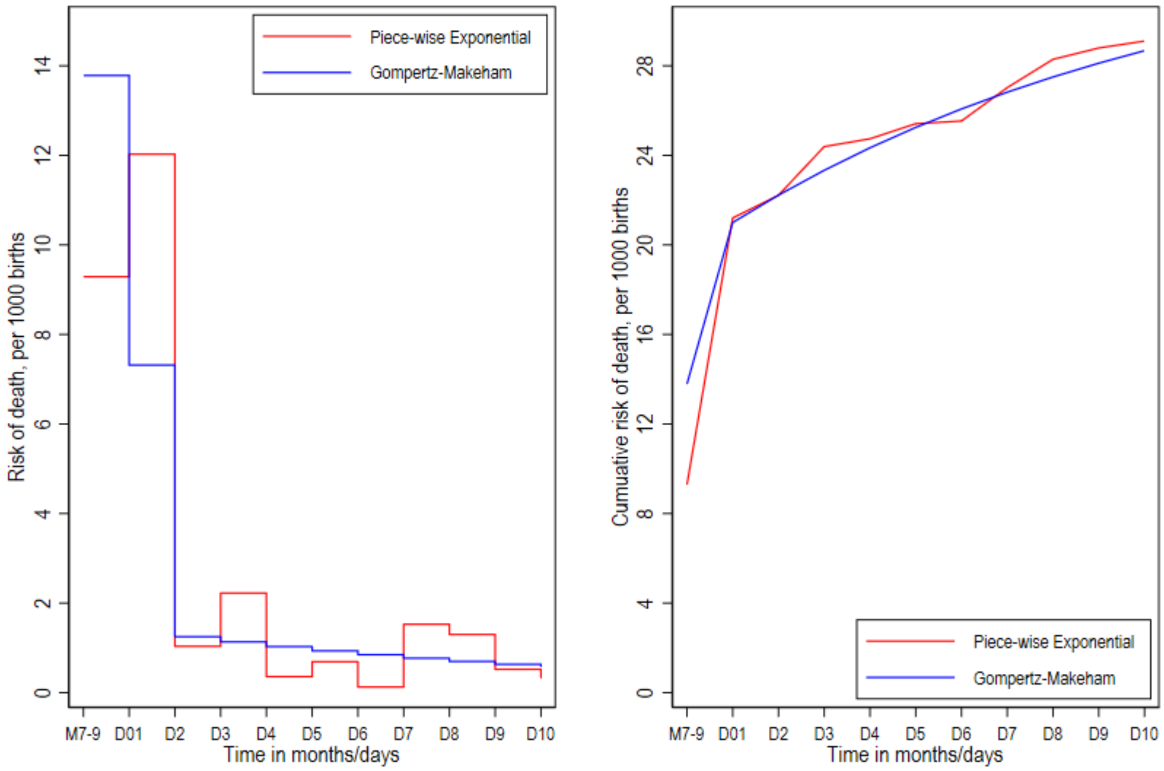

Figure S2: Peru DHS-2000

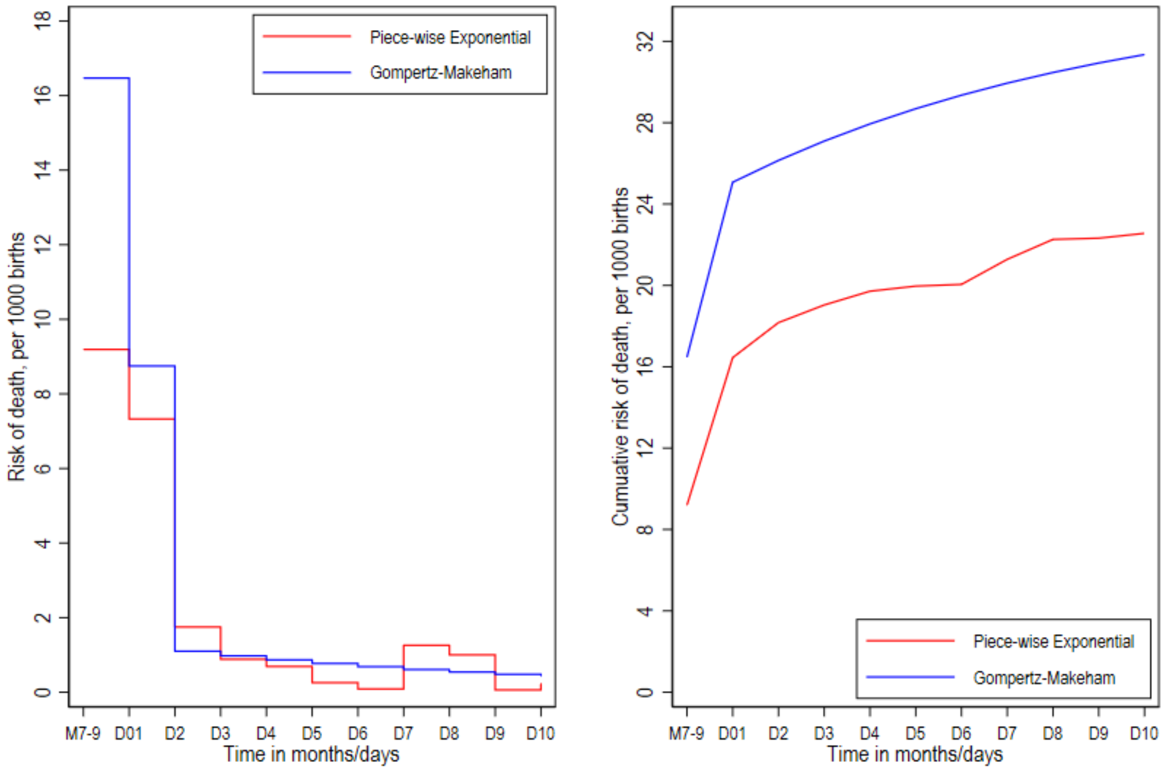

Figure S2: Peru DHS-2004/6

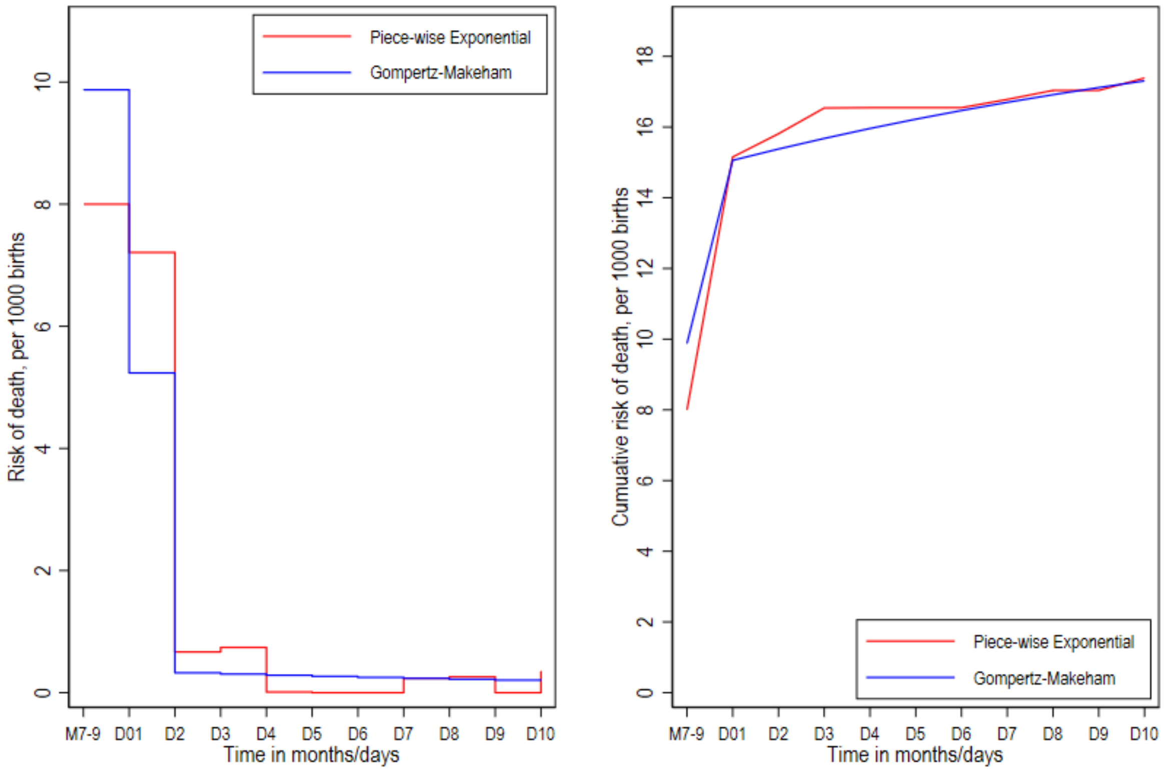

Figure S2: Peru DHS-2007/8

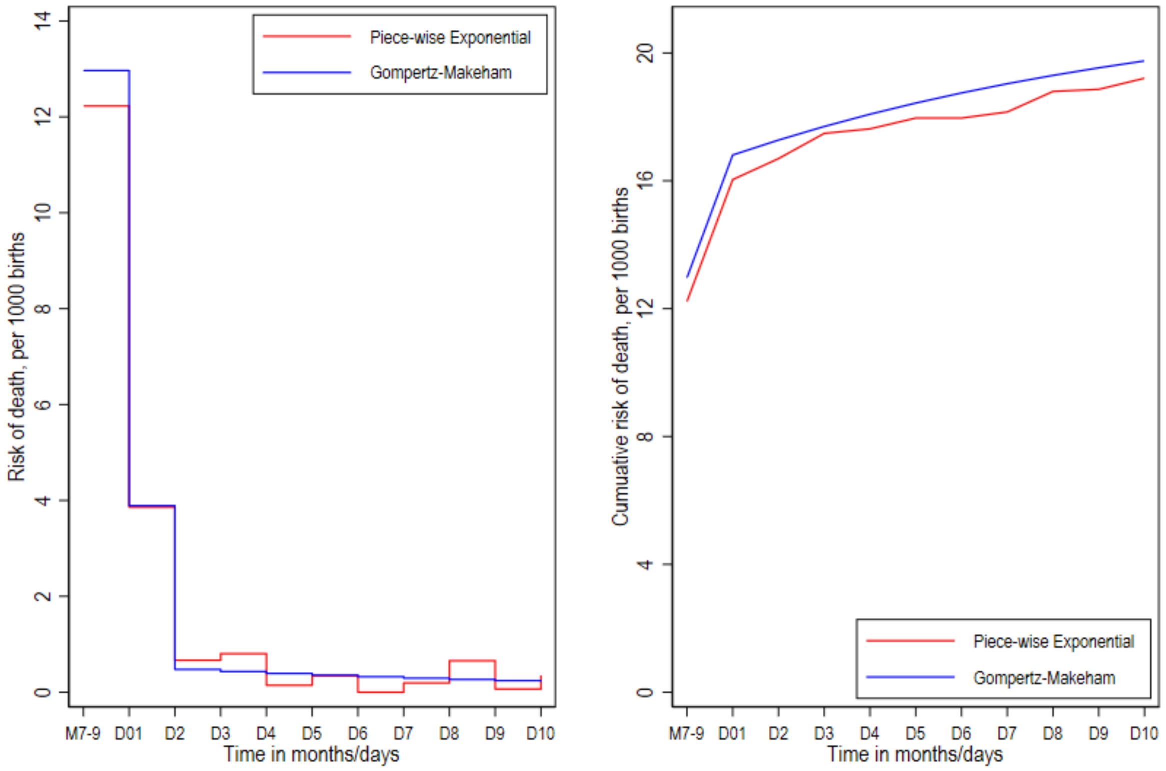

Figure S2: Peru DHS-2009

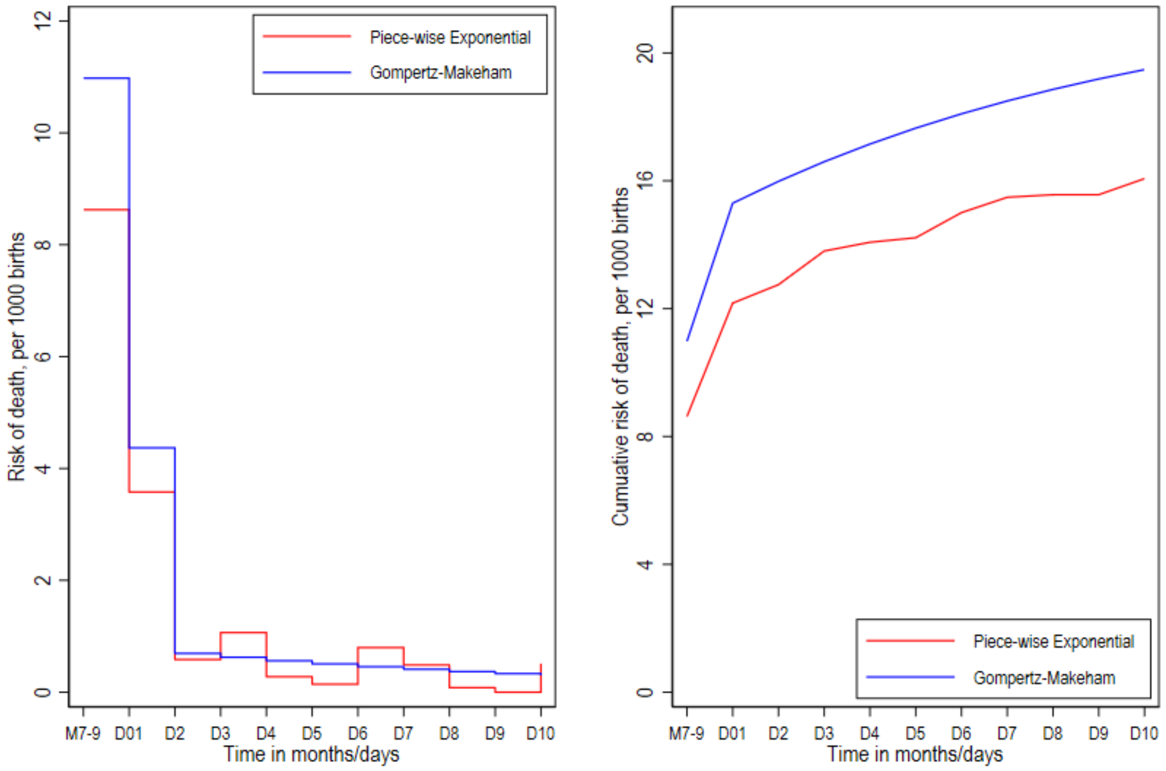

Figure S2: Peru DHS-2010

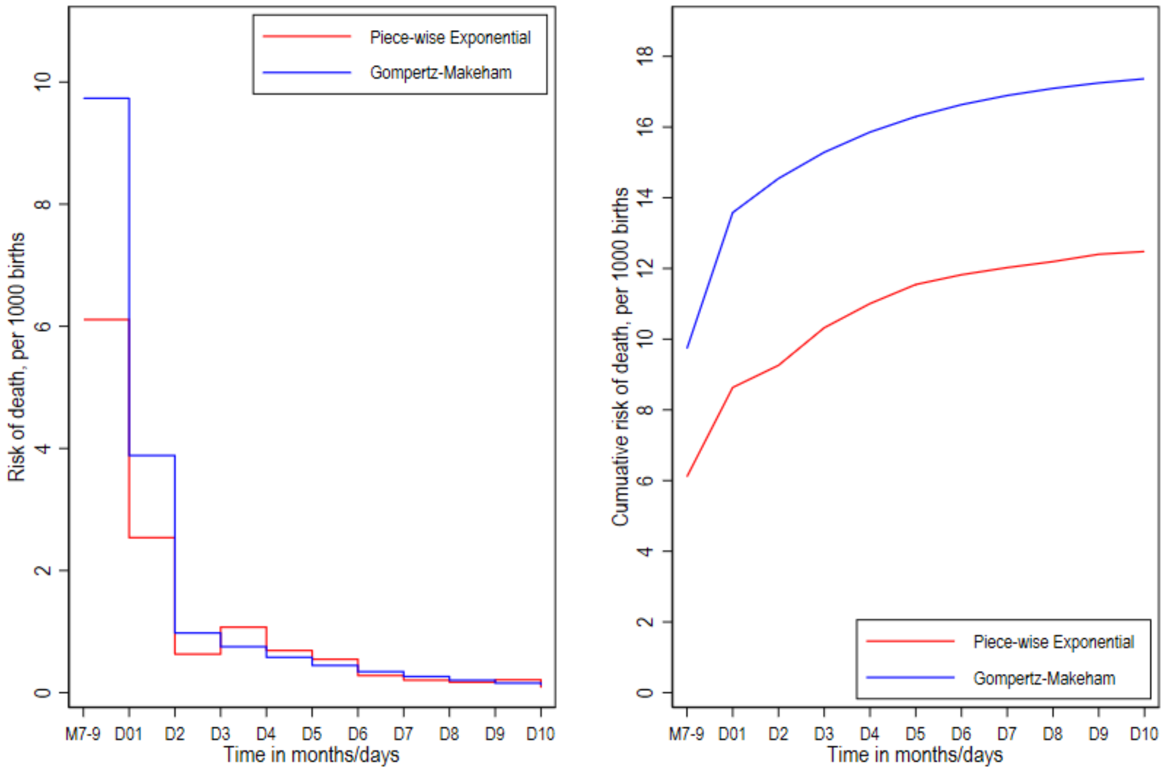

Figure S2: Peru DHS-2011

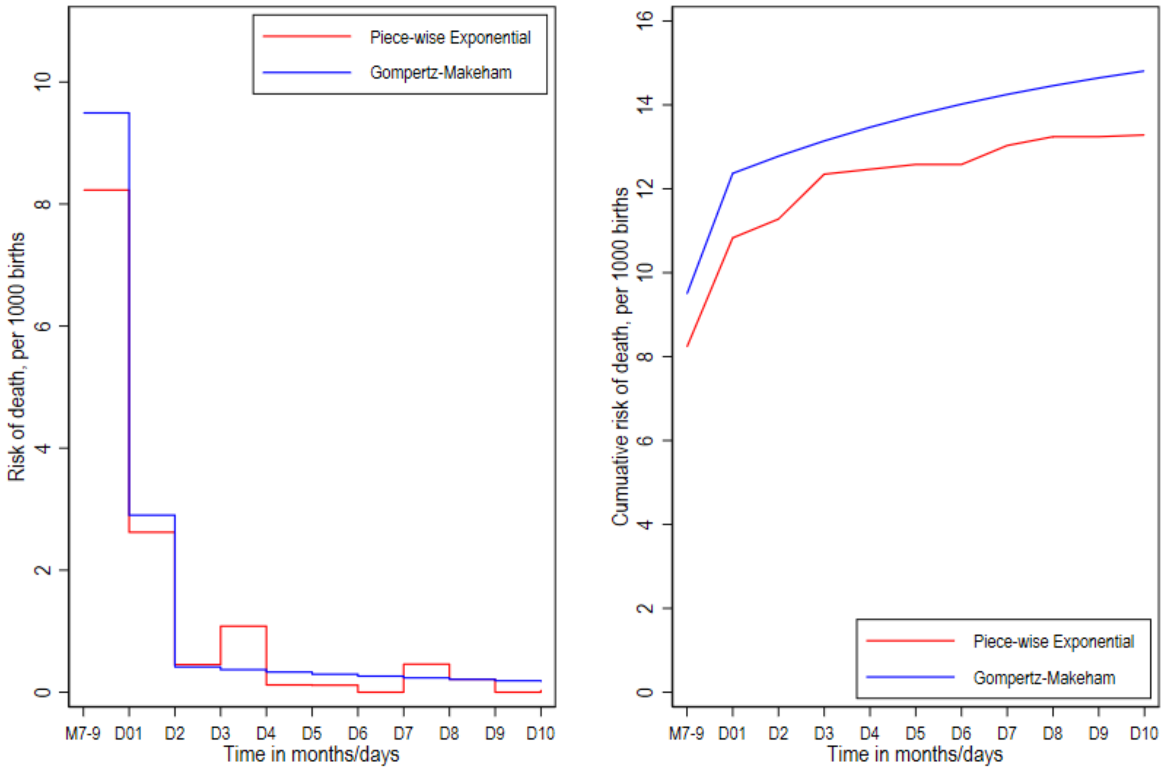

Figure S2: Peru DHS-2012

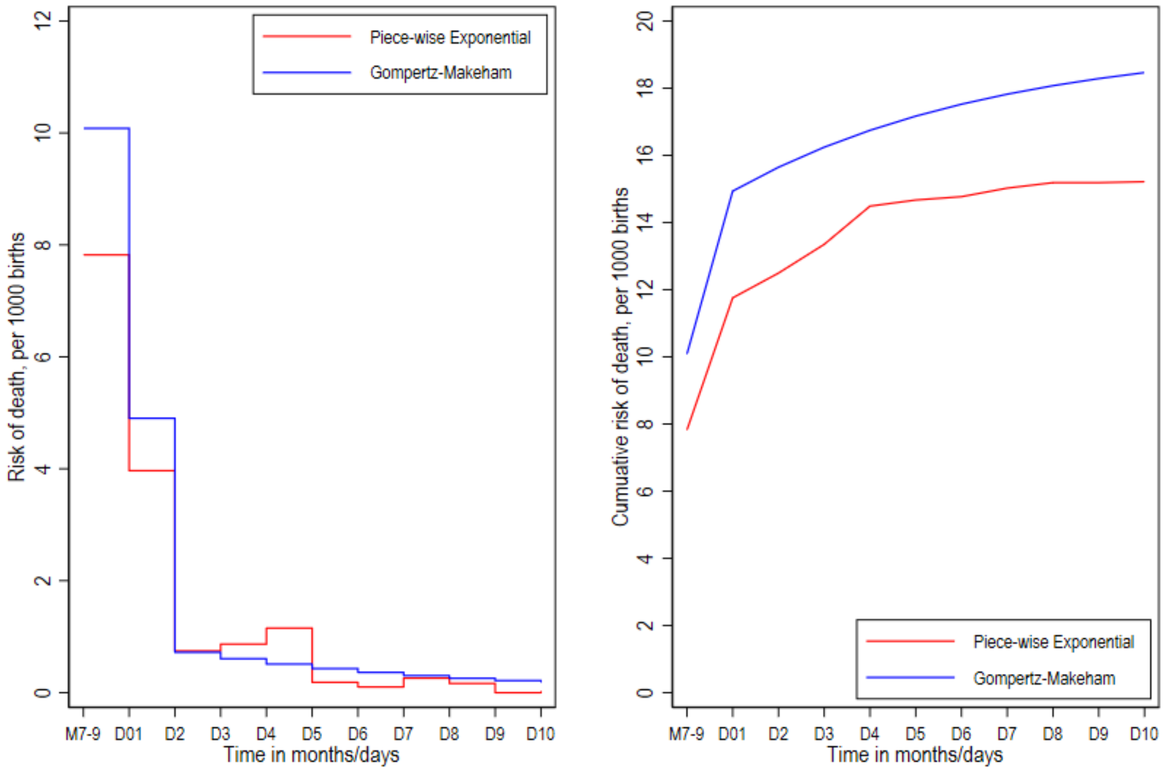

Supplement: Supplementary appendix [file mmc1.pdf]
